# Structure-guided design of a methyltransferase-like 3 (METTL3) proteolysis targeting chimera (PROTAC) incorporating an indole–nicotinamide chemotype†

**DOI:** 10.1039/d5md00359h

**Published:** 2025-06-19

**Authors:** Annabelle C. Weldert, Ariane F. Frey, Mackenzie W. Krone, Franziska Krähe, Hannah Kuhn, Christian Kersten, Fabian Barthels

**Affiliations:** a Institute of Pharmaceutical and Biomedical Sciences, Johannes Gutenberg-University Staudingerweg 5 55128 Mainz Germany barthels@uni-mainz.de; b Department of Molecular, Cellular, and Developmental Biology, Yale University 260 Whitney Ave CT 06520-8103 New Haven USA; c Institute for Quantitative and Computational Biosciences, Johannes Gutenberg-University BioZentrum I, Hanns-Dieter-Hüsch Weg 15 55128 Mainz Germany

## Abstract

Methyltransferase-like 3 (METTL3) is the main catalytic subunit of the m^6^A methyltransferase complex (MTC) and plays an essential role in various disease indications, including acute myeloid leukemia (AML). Here, we describe the structure-guided design and evaluation of METTL3 proteolysis-targeting chimeras (PROTACs), starting from the potent small-molecule inhibitor STM2457. Across four design generations, we highlight key considerations, particularly regarding the exit vector, linker mechanics, and METTL3-binding chemotype composition. Our most effective PROTAC, AF151, forms a stable complex between the E3 ligase von Hippel–Lindau (VHL) and the target-of-interest METTL3, demonstrating efficient METTL3 degradation (DC_50_ = 430 nM) in the AML cell line MOLM-13. This molecule candidate exhibits more pronounced effects on viability inhibition (IC_50_ = 0.45 μM) and more significant m^6^A level reduction in cancer cells than its non-PRTOAC parent compounds. By incorporating the indole-nicotinamide chemotype as the METTL3-binding recruiter, this PROTAC is structurally distinct from recently published METTL3 PROTACs, expanding the design options for future METTL3 degrader development.

## Abstract 2

Iterative PROTAC design meets molecular fine-tuning—a medicinal chemistry journey to targeted METTL3 degradation.

## Graphical abstract

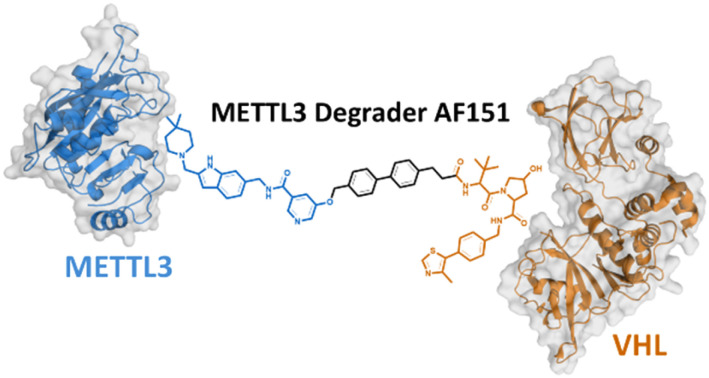

## Introduction

RNA modifications are critical for cellular activities, both at the transcriptional and post-transcriptional levels. Among the various RNA modifications, *N*^6^-methyladenosine (m^6^A) is the most prevalent in mammalian cells,^1^ influencing gene expression and cellular processes such as differentiation and stress adaptation.^2–4^ m^6^A is regulated by three key protein groups: writers (methyltransferases), erasers (demethylases), and readers (m^6^A-binding proteins).^5^ The primary writer of m^6^A in mRNA is the m^6^A methyltransferase complex (MTC), consisting of methyltransferase-like 3 and 14 (METTL3 and METTL14) and their cofactors, like the Wilms' tumor 1-associating protein (WTAP).^6^ As the enzymatic core of the MTC, METTL3 transfers methyl groups from its co-substrate *S*-adenosylmethionine (SAM) to target mRNA, while METTL14 facilitates RNA substrate recognition.^7^

The MTC plays a critical role in numerous diseases, including cardiovascular diseases,^8^ viral infections,^9,10^ and neurological disorders.^11^ Additionally, METTL3 is overexpressed in various cancers, including hematological,^12^ lung,^13^ and liver cancer.^14^ In acute myeloid leukemia (AML), METTL3 enhances the translation of oncogenes like Bcl2, promoting cell survival.^12^ Moreover, its depletion leads to cell cycle arrest, differentiation, and apoptosis, delaying leukemia progression.^12,15^ Similarly, small-molecule inhibition of METTL3 has been shown to reduce AML growth and increase differentiation and apoptosis, highlighting the pharmacological potential to target METTL3.^16^ Several small-molecule METTL3 targeting SAM-competitive inhibitors have been reported, including the selective and highly potent UZH2,^17^ EP652,^18^ STM2457,^16^ and STM3006.^19^ The latter two compounds were developed by STORM Therapeutics, whose lead candidate, STC-15, recently entered clinical trials, being the first RNA methyltransferase inhibitor in this class.^20,21^

Despite their low nanomolar potency in biochemical assays, most METTL3 inhibitors require micromolar concentrations to reduce m^6^A mRNA modification levels in cells,^16,17,19^ likely due to competition with the high intracellular concentration of SAM and other pharmacokinetic effects.^22^ Furthermore, m^6^A-independent mechanisms for METTL3, which cannot be captured by competitive SAM inhibition, have been demonstrated to promote tumorigenesis and translation as well.^13,23^ Hence, an alternative approach to target catalytic and non-catalytic functions of METTL3 at the same time is selective degradation using proteolysis-targeting chimeras (PROTACs). PROTACs are an emerging class of new modalities in drug discovery, with recent candidates entering late-stage clinical trials.^24,25^ These heterobifunctional small molecules contain a protein of interest (POI) ligand, an E3 ligase ligand, and a linker connecting both. PROTACs form ternary complexes with the E3 ligase and POI, leading to ubiquitination and subsequent degradation of the POI *via* the ubiquitin–proteasome system (UPS). This targeted protein degradation impairs both enzymatic and structural functions, thereby expanding therapeutic applications.^26–29^

During the preparation of this manuscript (2024–2025), the first reports of heterobifunctional degraders targeting METTL3 have been published, highlighting the current interest in this specific target class.^30–33^ Yet, all reported METTL3 PROTACs are based on the METTL3 recruiting chemotype of UZH2, limiting their structural diversity. Errani *et al.* developed degrader molecules that achieved approximately 50% degradation of METTL3 in multiple AML cell lines.^30^ Almost simultaneously, Du *et al.* published the potent (*D*_max_ ∼ 91%) METTL3 degrader WD6305.^31^ Soon after, Hwang *et al.* further highlighted the therapeutic potential of METTL3 degradation in gastric cancer cells by targeting its non-catalytic function.^32^ Meanwhile, Nar *et al.* demonstrated the synergistic effects of their METTL3 degraders with other anti-cancer agents.^33^ While all these studies confirm the therapeutic potential of METTL3 degraders, particularly against AML, the METTL3 PROTACs developed so far lack structural diversity, as all of them use UZH2 (ref. 17) as the METTL3-recruiting chemotype.

In this study, we report the development of **AF151** (compound 41), a potent METTL3 degrader with an indole-nicotinamide METTL3 binding chemotype based on a series of METTL3 ligands developed by STORM Therapeutics^16^ and EPICS Therapeutics.^18^**AF151** exhibits efficient METTL3 degradation and reduces viability in AML cells, offering a structurally distinct alternative to previously reported UZH2-based PROTACs. Here, our synthesis and testing of over 40 PROTAC candidates will support the continued development of METTL3 PROTACs.

## Experimental section

### Synthesis

The synthetic procedures of all compounds, including NMR and LC-MS data, are detailed in the ESI.†

### Reference compounds

WD6305, MLN4924, and elvitegravir were purchased from MedChemExpress. Venetoclax, STM3006, and bortezomib were purchased from BLDpharm. STM2457 was purchased from Cayman Chemicals. VH032 (ref. 34) was synthesized according to reported procedures. Crystal structures of ligand complexes were taken from the Protein Data Bank (PDB).^35^

### Recombinant protein expression and purification

#### METTL3/14

A METTL3/14 expression plasmid was used as described previously.^36^ In brief, a pETDuet-1 vector containing the MTase domains of human METTL3 (UniProt: Q86U44, aa 252–580) and METTL14 (UniProt: Q9HCE5, aa 108–444) was introduced into *E. coli* BL21 (DE3) cells. The METTL3 construct carried a C-terminal Strep-tag II, while METTL14 was fused to an N-terminal hexahistidine (His_6_) tag. The cells were grown in LB medium containing 100 μg mL^−1^ ampicillin at 37 °C and 160 rpm until they reached an optical density (OD_600_) of ∼0.6. Protein overexpression was induced by adding 0.1 mM isopropyl-β-d-1-thiogalactopyranoside (IPTG) and 0.1 mM ZnCl_2_, followed by incubation for ∼16 h at 18 °C. Cells were harvested by centrifugation and suspended in cold lysis buffer (10 mM potassium phosphate, pH 7.5, 300 mM KCl, 20 mM imidazole, 10% glycerol, 0.1 mM TCEP). Lysozyme, one tablet of protease inhibitor (cOMPLETE), and DNAse I were added. After a 30 minute incubation on ice, the cells were lysed by sonication, and the cell debris was removed by centrifugation. The resulting clear supernatant was kept on ice. The pellet was resuspended in lysis buffer, subjected to a second round of sonication, and centrifuged again to remove insoluble components. The clarified lysates were applied to a HisTrap HP column for affinity purification, pre-equilibrated in binding buffer (10 mM potassium phosphate, pH 7.5, 300 mM KCl, 20 mM imidazole). After washing with several column volumes of wash buffer (10 mM potassium phosphate, pH 7.5, 500 mM KCl, 20 mM imidazole), the protein was eluted with elution buffer (10 mM potassium phosphate, pH 7.5, 300 mM KCl, 250 mM imidazole). Finally, METTL3/14 was purified *via* size-exclusion (SEC), on a Superdex 16/60075 pg SEC column equilibrated with SEC buffer (25 mM Tris HCl, pH 7.5, 100 mM NaCl). METTL3/14 was flash-frozen in liquid nitrogen and stored at −80 °C.

### VHL, elongin B, and elongin C (VCB)

VHL (UniProt: P40337, aa 1–213), elongin B (UniProt: Q15370, aa 1–118), and elongin C (UniProt: Q15369, aa 17–112) were expressed as a complex (VCB) as described previously with slight modifications.^37^ Briefly, a pCDFDuet-1 vector containing elongin B and C and a pET28a vector containing VHL, harboring a hexahistidine (His_6_) tag at the N-terminus, were co-transformed in *E. coli* BL21 (DE3) cells. The cells were grown in LB medium containing 50 μg mL^−1^ kanamycin and 50 μg mL^−1^ streptomycin sulfate at 37 °C and 160 rpm until they reached an OD_600_ of ∼0.7. Overexpression was induced by adding 0.3 mM IPTG for ∼16 h at 24 °C. After harvesting the cells by centrifugation, the pellet was resuspended in cold lysis buffer (50 mM HEPES, pH 8.0, 500 mM NaCl, 20 mM imidazole, 5 mM mercaptoethanol). One tablet of protease inhibitor and DNAse I were added, and the cells were lysed by sonication. After removing the cell debris with centrifugation, the supernatant was filtered and applied to a HisTrap HP column for affinity purification, pre-equilibrated in lysis buffer. Unbound sample was washed with 10 CV lysis buffer, and the VCB complex was eluted in a linear gradient of elution buffer (50 mM HEPES, pH 8.0, 500 mM NaCl, 500 mM imidazole, 5 mM mercaptoethanol) from 0 to 100% over 20 CV. The pooled fractions were combined and dialyzed against 20 mM HEPES, pH 8.0, 20 mM NaCl, and 1 mM DTT at 4 °C overnight. Afterwards, the VCB complex was subjected to anion exchange chromatography (AEX) on a HiTrap HP column, preequilibrated in dialysis buffer. The protein complex was eluted in a linear gradient of elution buffer 2 (20 mM HEPES, pH 8.0, 500 mM NaCl, 1 mM DTT) from 0 to 100% over 20 CV. Lastly, the VCB complex was purified by SEC on a Superdex 16/600 75 pg SEC column equilibrated with SEC buffer (20 mM HEPES, pH 7.5, 100 mM NaCl, 1 mM TCEP). The VCB complex was flash-frozen in liquid nitrogen and stored at −80 °C.

### Fluorescence polarization (FP) assays

Fluorescence polarization (FP) displacement assays with METTL3/14 (ref. 36) and VCB^38–40^ were performed following previous protocols with slight adaptations. All experiments were conducted in black Greiner 96-well half-area plates using a Tecan Spark 10 M plate reader, equipped with polarization filters coupled to a monochromator setup (*λ*_ex_ = 480 nm, *λ*_em_ = 530 nm). Reaction mixtures for METTL3/14 contained 300 nM recombinant enzyme, 20 nM FAM-labelled displacement tracer (STM-FL^36^), and the test compounds at varying concentrations in 25 mM Tris HCl, pH 7.5, 100 mM NaCl, and 10% DMSO. For VCB, the mixtures contained 40 nM recombinant enzyme, 10 nM FAM-labelled probe (FAM-DEALAHypYIPMDDDFQLRSF, Tocris Bioscience), and the tested compounds, at varying concentrations, in 50 mM Tris, pH 7.5, 200 mM NaCl, 2 mM DTT, and 10% DMSO. For the ternary *K*_D_ determinations, METTL3/14 was added at 12 μM, corresponding to approximately a 30- to 100-fold excess of binary *K*_D_. All measurements were performed at least as technical triplicates. Polarization values (mP) were determined from polarization-specific parallel and orthogonal fluorescence intensities using the Tecan in-built calculation routine. Using GraphPad Prism 8.0.1, the FP values were normalized, and *K*_D_ values were calculated using a 4-parameter Hill equation: *y*(% probe bound) = bottom + ([ligand]slope) × (top–bottom)/([ligand]slope + *K*_D_ slope).

### Homogeneous time-resolved fluorescence (HTRF) assays

PROTACs binding to VHL or CRBN were measured using a homogeneous time-resolved fluorescence (HTRF) human VHL or CRBN binding kit (Revvity). Samples were prepared according to the manufacturer's protocol, with compounds diluted in the provided diluent while maintaining a constant DMSO concentration with a final concentration of 1.25%. For the ternary *K*_D_ determinations, METTL3/14 was added at 4 μM, corresponding to approximately a 20- to 40-fold excess of binary *K*_D_, while all other component concentrations were kept as specified in the protocol. Measurements were performed in triplicate using a Tecan Spark 10 M plate reader, recording donor and acceptor emissions at *λ*_em_donor_ = 620 nm and *λ*_em_acceptor_ = 665 nm, respectively, following excitation of the HTRF donor at *λ*_ex_ = 320 nm. The acceptor-to-donor emission ratio was calculated as ratio = signal 665 nm/signal 620 nm. Using GraphPad Prism 8.0.1, the fluorescence values were normalized, and *K*_D_ values were calculated as specified above.

### Computational analysis

#### Docking

Ternary PROTAC docking was performed using method 4B of the degrader modeling tool in the Molecular Operating Environment (MOE) 2022.02.^41–43^ Briefly, in this method, two protein–ligand complexes were provided, one for the E3 ligase and one for the target. Additionally, the structure of the entire degrader was provided. Protein–protein docking was conducted between the E3 ligase and target complex, and subsequently, different conformations for the PROTAC compound were generated. Lastly, the protein–protein docked ensemble and the PROTAC conformational ensemble were combined. A cluster number was generated for the protein and PROTAC conformations found across all poses, and a double cluster number was assigned by concatenating the two individual cluster numbers. This double cluster population can then be used to prioritize hit selection.^42^ The following specifications were applied: PROTAC structures were protonated using Protonate3D^44^ and energy-minimized using the MMFF94x^45^ force field, while all other calculations employed the Amber10:EHT force field. For WD6305 (ref. 31) and KH12,^32^ METTL3/14 (PDB: 7O2F^17^) and VCB (PDB: 4W9H^34^), crystallographic binding modes of the MTase and VHL ligands were directly used as templates in the docking protocol. For **AF151**, VCB (PDB: 4W9H^34^) was also used directly, whereas for METTL3/14 (PDB: 8BN8 (ref. 19)) a template docking with the METTL3/14-binding moiety of **AF151** was performed before ternary complex docking. Missing residues and loops were reconstructed before docking using MOE's *de novo* loop modeler. For MD simulations, for each ternary complex (VCB–**AF151**–METTL3/14, VCB–KH12–METTL3/14, and VCB–WD6305–METTL3/14), the best-scoring representative structures of the three largest double clusters were selected. All structures were protonated using Protonate3D within MOE to account for changes from the ternary complex formation.

### Molecular dynamics (MD) simulations

PROTACS were parameterized using antechamber^46^ and parmchk2 from the AmberTools24 (ref. 47) with the generalized amber force field (GAFF2 (ref. 48)) and AM1-BCC charges.^49^ Ternary complex structures were built with tleap and minimized using sander over 200 time steps. Complexes were then neutralized using sodium ions (Na^+^) and solvated using a TIP3P^50^ water box exceeding the complexes by 10.0 Å in every dimension. Equilibration and production were performed with NAMD2.14 (ref. 51) and the AMBER (ff19SB^52^) force field. Equilibration was performed over 1 ns with gradually releasing harmonic constraints on the PROTAC and protein atoms and heating from 100 to 300 K. Production MD simulations were performed over 20 ns (all) and prolonged to 100 ns for selected structures (see below) using time steps of 2 fs with rigid bond lengths and a van-der-Waals cut-off of 14.0 Å using periodic boundary conditions in an NPT ensemble. Simulations were visually inspected and analyzed using VMD-1.9.3.^53^ Molecular mechanic-generalized born surface area (MM-GBSA) analysis was performed using the 1-trajectory (1A) method with the ante-MMPBSA.py and MMPBSA.py^54^ implementation of AmberTools24 with GB model igb5.^55^ Simulations of 20 ns or 100 ns, respectively, were analyzed using an interval of 0.1 ns and a salt concentration of 0.1 M. As described previously,^56^ the energetic contributions were dissected into their respective parts from the individual binary and ternary complexes from target (T, here METTL3/14), E3 ligase (E, here VCB), and PROTAC (P) (Tables S2 and S3†).

### Cell culture

Human acute myeloid leukemia MOLM-13 cells were cultured in Roswell Park Memorial Institute (RPMI 1640) medium with 10% fetal bovine serum (FBS) and 100 μg mL^−1^ penicillin–streptomycin mix at 5% CO_2_ and 37 °C. Cell densities were maintained at 0.3–2 × 10^6^ cells per mL. MOLM-13 cells were obtained from DSMZ-German Collection of Microorganisms and Cell Cultures GmbH. All reagents and tissue culture plastics were purchased from Thermo Fisher Scientific.

### Immunoblotting

MOLM-13 were seeded in 12-well cell culture plates at a density of 1 × 10^6^ cells per mL and treated with DMSO (final: 0.1%) or different concentrations of PROTAC degraders in DMSO at the indicated times. Treatments were conducted in biological triplicate with varying passages of MOLM-13 cells. The cells were harvested by centrifugation at 500*g* for 5 min and washed with PBS. The cells were lysed with RIPA lysis buffer with the addition of protease inhibitor (1× cOMPLETE), and insoluble cellular parts were separated by centrifugation (12 000*g* for 10 min), subsequently, the supernatant was boiled with 1× Laemmli sample buffer (Carl Roth) for 5 min at 95 °C. Proteins were separated by SDS–PAGE on 4–20% Criterion™ TGX™ Precast Protein Gels (Bio-Rad) and transferred to Amersham™ Protean® 0.45 μm nitrocellulose membrane (Cytiva) using semi-dry transfer (25 V, 1 A, Towbin buffer). The membrane was blocked with 5% milk (Carl Roth) in TBST buffer and incubated with the following primary antibodies: Anti-METTL3 (1 : 1000, Abcam, ab195352), anti-METTL14 (1 : 1000, Abcam, ab220030), anti-Bcl2 (1 : 1000, Cell Signaling Technology [CST], EPR16891), anti-Mcl-1 (1 : 1000, CST, 5453 T), anti-Bcl-XL (1 : 1000, CST, 2764 T), anti-vinculin (1 : 1000, CST, 13901) or anti-GAPDH (1 : 5000, CST, D17C4) overnight at 4 °C. Subsequently, the membrane was incubated for 1 h at RT with secondary antibodies: anti-rabbit (1 : 2000, VWR, NA934) or anti-mouse (1 : 2000, VWR, 102646-160). The protein bands were detected *via* chemiluminescence using Pierce™ ECL western blotting substrate and a Fusion FX imaging system (Vilber). The ImageLab software (Bio-Rad) was used for band quantification.

### Reverse transcription polymerase chain reaction (RT-qPCR)

To determine mRNA expression levels of METTL3 and METTL14, MOLM-13 cells were treated in triplicate with compound 41 (**AF151**, 2 μM), STM2457 (10 μM), and DMSO for 24 resp. 48 h. Afterward, total RNA was extracted using the TRIzol™ reagent (Thermo Fisher Scientific) and purified with the Monarch® Spin RNA Cleanup Kit (New England Biolabs). RT-qPCR was conducted using the two-step GoTaq® RT-qPCR system (Promega) following the manufacturer's protocol. The data were normalized to the DMSO control and GAPDH housekeeping gene (ΔΔCt) using GraphPad Prism 8.0.1. The primer sequences used for RT-qPCR are listed in Table S4.†

### Cell viability assays

To determine cell viability, MOLM-13 cells were treated with the indicated compounds in a 1 : 1 serial dilution, with pure DMSO (final: 0.1%) as the negative control. Briefly, 1000 cells were seeded in black Greiner half-area 96-well plates and incubated for 72 h with inhibitors from DMSO stocks under standard cultivation conditions. Then, Promega CellTiter Glo 2.0 reagent was added to each well according to the manufacturer's protocols, and luminescence was measured using a Tecan Spark 10 M. Experiments were conducted in technical triplicate. Data was normalized to the DMSO control and analyzed with GraphPad Prism 8.0.1 to determine EC_50_ values. Synergistic effects of venetoclax and **AF151** on cell MOLM-13 viability were conducted accordingly, with dilution series of both compounds analyzed using the SynergyFinder+ webtoolkit.^57^

### RNA m^6^A quantification

Quantification of m^6^A levels upon compound treatment was conducted in analogy to the study introducing the WD6305 PROTAC.^31^ MOLM-13 cells were treated with **AF151** (2 μM), STM2457 (10 μM), and DMSO in triplicate for 24 and 48 h. Total RNA was isolated as specified above. PolyA mRNA was then isolated using the PolyATtract® mRNA Isolation System (Promega). m^6^A levels were quantified using a colorimetric m^6^A RNA methylation Assay Kit (Abcam) as specified in the manufacturer's protocol. The data was normalized to the DMSO control and displayed using GraphPad Prism 8.0.1.

### Real-time apoptosis assay

MOLM-13 cells were treated with **AF151** at the indicated concentrations for 48 h, with DMSO (final: 0.1%) as the negative control. Apoptosis of technical triplicate samples was detected in real-time using the RealTime-Glo™ annexin V apoptosis assay kit (Promega), following the manufacturer's protocol. Data were plotted using GraphPad Prism 8.0.1.

## Results and discussion

### Development and initial evaluation of PROTAC candidates

**Fig. 1:**
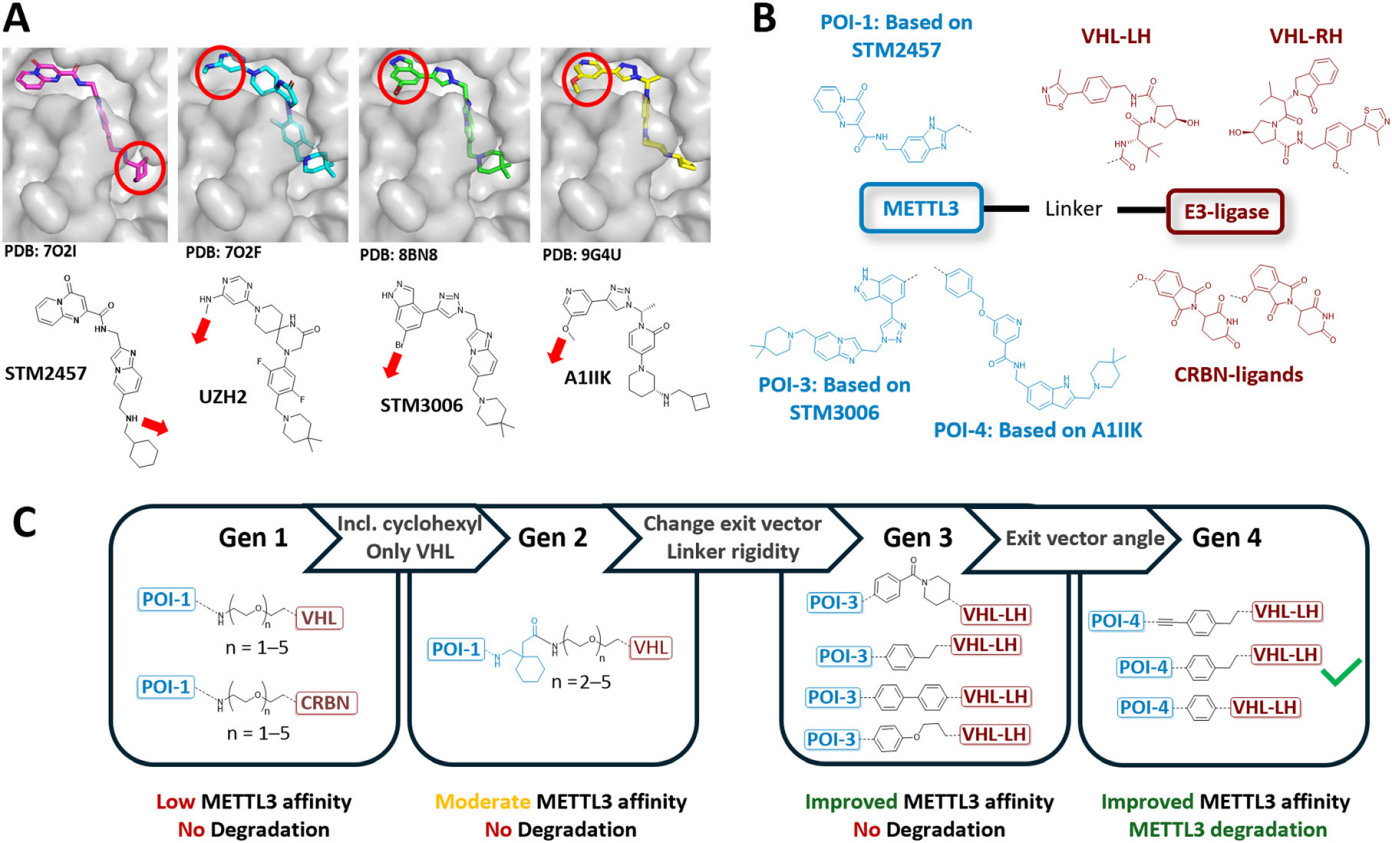
Development pipeline of PROTACs targeting METTL3. (A) Crystal structures of various METTL3-targeting small-molecule inhibitor complexes: STM2457 (PDB: 7O2I), UZH2 (PDB: 7O2F), STM3006 (PDB: 8BN8), and A1IIK (PDB: 9G4U), showing solvent-exposed regions and thereby possible exit vectors for linker attachment sides. (B) General design and selected structures of our METTL3 PROTAC strategy, including METTL3 and E3 ligase binding moieties used in this study. (C) Structural representation of METTL3 PROTAC development across four generations, specifying changes made between each generation.

The development pipeline of our METTL3 PROTACs, spanning four molecular generations, is shown in Fig. 1. In our heterobifunctional designs, we aimed to recruit the E3 ligases von Hippel–Lindau (VHL) or cereblon (CRBN) in a ternary complex with METTL3, since well-established ligands are available for all proteins.^58^ For VHL, we chose VH032 (ref. 34) and VL285 (ref. 59) (hereinafter referred to as VHL-LH and VHL-RH, respectively), and for CRBN, 4-hydroxy and 5-hydroxy thalidomide^60^ as E3 ligase ligands. For METTL3, we chose the small molecule inhibitor STM2457, developed by STORM Therapeutics, due to its high selectivity and potency.^16^ Compounds from the STM series show high *in vitro* potency, favourable ADME properties, and efficient m^6^A level reduction in cancer cells. Compared to the UZH2-based degraders, the STM-based parent compound has been characterized more extensively in clinically relevant AML models. In this regard, STC-15 has shown a robust average reduction of 63% in m^6^A levels in peripheral blood in humans in a phase 1 clinical trial.^20^

To identify a suitable attachment point for linker conjugation, we analyzed the X-ray structure of STM2457 bound to METTL3 (PDB: 7O2I^16^). Since the cyclohexyl moiety of STM2457 allows the attachment of a solvent-exposed exit vector (Fig. 1A), we selected this molecular segment for linker attachment. Based on the results of a SAR study reported by STORM Therapeutics,^61^ we attached a linker segment directly to the secondary amine instead of the cyclohexyl moiety. Further, the original imidazopyridine scaffold was replaced with a benzimidazole for synthetic reasons, yielding **POI-1** (Fig. 1B). To connect **POI-1** to the E3 ligase ligands, we opted for polyethylene glycol (PEG_*n*_, *n* = 1–5) linkers, yielding our first generation of METTL3 PROTACs (Fig. 1C). Synthesis and detailed structure of all generations are shown in the ESI.†

Next, we evaluate the affinity of the PROTAC candidates for METTL3 binding using a competitive fluorescence polarization (FP) assay (Table S1†). This revealed that VHL-based PROTACs exhibited an overall higher affinity for METTL3 compared to CRBN-based PROTACs. However, in general, the METTL3 affinities of our first-generation PROTACs were relatively low compared to the parent compound STM2457 (*K*_D_ = 1.4 nM^16^), with our best compounds showing affinities around *K*_D_ ∼ 4 μM (Table S1†). We concluded that the cyclohexyl moiety of STM2457 is necessary for high-affinity METTL3 binding. Furthermore, Hwang *et al.* and Errani *et al.* have reported that CRBN-based METTL3 PROTACs exhibit lower degradation activity compared to their VHL-based counterparts.^30,32^ Hence, we decided to focus only on the VHL-based PROTACs in the following generations of PROTACs.

**Fig. 2:**
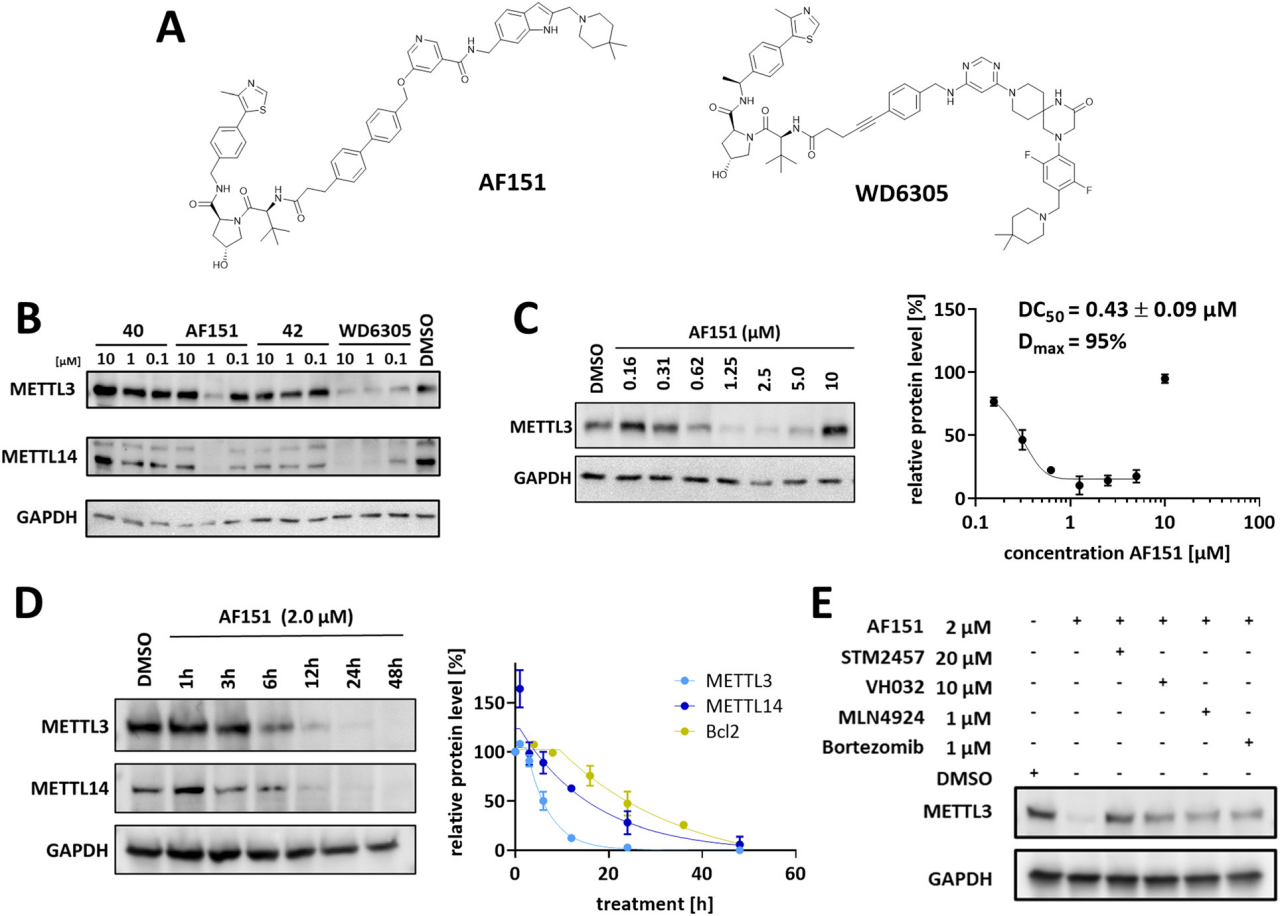
Cellular characterization of METTL3 PROTAC **AF151**. (A) Structures of **AF151** and reference degrader WD6305, developed by Du *et al.*^31^ (B) Western blots of PROTAC screening results analyzing the fourth generation of our PROTAC design. MOLM-13 cells were treated with the indicated compounds at different concentrations (10 μM, 1 μM, and 0.1 μM) for 16 h. WD6305 and DMSO were used as positive and negative controls. (C) Dose–response experiments and DC_50_ determination for **AF151** in MOLM-13 cells. Left: Western blot image after treatment at the indicated concentrations for 16 h. Right: Dose–response curve with different **AF151** concentrations plotted against METTL3 protein levels relative to the DMSO control. (D) Time course treatment experiments. Left: Western blot image after treatment with **AF151** at 2 μM at the indicated time points. Right: Time-response curve with different time points plotted against METTL3, METTL14, and Bcl-2 protein levels relative to the DMSO control. (E) Cellular PROTAC competition assay. MOLM-13 cells were treated with **AF151** in combination with the METTL3 inhibitor STM2457, VH032, MLN4924, or bortezomib at the indicated concentrations. Uncropped Western blot images can be found in Fig. S8–S11.†

Subsequently, we synthesized our second generation of VHL-based PROTACs, including a cyclohexyl motif (Fig. 1C), which demonstrated improved METTL3 affinity (*K*_D_ ∼ 300 nM, Table S1†). Next, we screened the METTL3 degradation activities of our most promising 2nd-generation PROTACs in MOLM-13 cells. However, none of these molecule candidates showed significant METTL3 degradation activity after treatment (final: 0.1–10 μM) for 16 h (Fig. S4†). Apart from possessing nanomolar affinity to METTL3 and the E3 ligase (Table S1†), PROTACs, in general, need to facilitate the right spatial orientation and alignment of both proteins to achieve efficient target degradation. Linker geometry, length, and rigidity play a crucial role in this process.^28,62,63^ Errani *et al.* showed that moving from flexible PEG linkers to more rigid benzyl, piperidine, and piperazine linkers can improve METTL3 degradation; additionally, the increase in lipophilicity enhanced cellular permeability.^30^ Furthermore, WD6305 (Fig. 2A), the highly potent PROTAC developed by Du *et al.*, features a relatively rigid and short linker compared to the less potent ones in this study.^31^ Based on these findings, we selected a series of shorter and more rigid linkers for our third-generation design (Fig. 1C). Additionally, we theorized that the previously used exit vector of STM2457 (pointing to the RNA binding site) might not be optimal for METTL3–VCB complex formation in cellulo.^6,64,65^ All currently published METTL3 degraders base their METTL3-binding functionality on UZH2, with a distinct exit vector away from the RNA binding site (Fig. 1A).

Hence, we changed our METTL3 recruiting ligand from STM2457 to STM3006, which allows us to exit METTL3 at a similar position to UZH2 by linker attachment to the indazole ring of **POI-3** (Fig. 1B).^19^ Yet, despite these optimizations, cellular degradation analysis still showed little effect on METTL3 protein levels (Fig. S4†). Compared to the potent literature-known WD6305 METTL3 degrader, our **POI-3** geometry differs only slightly by the angle and the rigidity of the exit vector, while linker lengths and position of the linkage are very similar to UZH2-based PROTACs (Fig. 1A). Hence, to further refine the flexibility and the angle of the exit vector, we continued optimizing **POI-3**: the co-crystal structure of EPICS Therapeutics' A1IIK (PDB: 9G4U^18^) showed that replacing STM3006's indazole moiety with a pyridine scaffold will allow an STM-based METTL3 recruiting chemotype but with a UZH2-like exit vector (Fig. 1A),^18^ and thus, yielding our 4th generation METTL3 recruiter **POI-4** (Fig. 1B).

Cell-based METTL3 degradation analysis showed that compound 41 (**AF151**, Fig. 2A), a **POI-4**-based PROTAC, led to significant degradation of METTL3 and its heterodimerized partner METTL14 after treatment for 16 h with 1 μM (Fig. 2B). To summarize, while our initial designs showed no METTL3 degradation, optimization efforts, including replacing flexible PEG linkers with more rigid linkers and refining the METTL3 exit vector, led to a functional METTL3 degrader in MOLM-13 cells.

### Analysis of **AF151**'s degradation efficiency and mechanism

Encouraged by the initial screening results, we further analyzed compound 41's (**AF151**) efficiency in degrading METTL3 in MOLM-13 cells and investigated the underlying molecular mechanisms. The half-degrading concentration (DC_50_) after 16 h of treatment was determined to be 430 nM (Fig. 2C). Maximal degradation (*D*_max_ ∼ 95%) occurred at 2.5 μM, while we noticed a characteristic “hook effect” >5 μM, where METTL3 degradation decreased. The high concentration of PROTAC leads to saturation of binding sites on both METTL3 and VHL, forming predominantly binary complexes and preventing the formation of ternary complexes and thereby degradation. However, at compound concentrations >5 μM, AF151 still acts as an inhibitor of METTL3, since the catalytic activity of the enzyme is still blocked by the binary complexes.

Time-course analysis revealed that **AF151** induces rapid METTL3 degradation, achieving over 50% METTL3 reduction within the first 6 h of treatment, reaching maximal effect at 24 h, and maintaining it for at least 48 h (Fig. 2D). METTL14 was reduced alongside METTL3, in agreement with prior METTL3 PROTAC studies.^30–33^ Of note, treatment with **AF151** led to an initial but transient increase of METTL14 (∼150%) after 1 h before inducing METTL14 degradation. Similarly, Errani *et al.* reported elevated levels of METTL3/14 after treatment with the small-molecule inhibitor UZH2. They proposed an unknown cellular compensatory mechanism, which might have led to an increase of METTL14.^30,66^ Half-lives for the degradation kinetics of METTL3 and METTL14 were determined at *t*_1/2_ = 4 h and 11 h, respectively. Thus, the degradation of METTL14 is significantly slower than that of METTL3, which can be explained by the fact that METTL3 occurs not only in complexes with METTL14 but also in a monomeric state and other protein complexes in cells.^67^

Next, we investigated the functional mechanism of **AF151** by confirming the involvement of the UPS. Co-treatment of the degrader with the proteasome inhibitor bortezomib (1 μM),^68^ neddylation inhibitor MLN4924 (1 μM),^69^ and VH032 (10 μM)^34^ suppressed METTL3 degradation, indicating that the process is both proteasome and VHL-dependent. Furthermore, adding a competitive METTL3 ligand STM2457 (20 μM) also inhibited degradation, confirming that **AF151** binds to the SAM-binding site of METTL3 (Fig. 2E).

Interestingly, the dose–response evaluation revealed that **AF151** and WD6305 share DC_50_ values (430 nM for **AF151***vs.* 140 nM for WD6305 (ref. 31)) in the same order of magnitude, but only **AF151** displays a characteristic hook effect at concentrations up to 10 μM. The relative ternary complex population, and thereby the hook effect, is known to be mainly influenced by the cooperativity of ternary complex formation.^70,71^ PROTACs can introduce *de novo* stabilizing or destabilizing protein–protein interactions during ternary complex formation. Hence, the binary binding affinity to METTL3 or the E3 ligase may differ from the ternary affinity in the presence of the second binding partner. This effect is quantified by the cooperativity factor (*α* = *K*_D_^binary^/*K*_D_^ternary^). Positive cooperativity (*α* > 1) indicates stabilizing *de novo* effects during ternary complex formation, while negative cooperativity (*α* < 1) signifies destabilizing *de novo* interactions.^70,72^

**Fig. 3:**
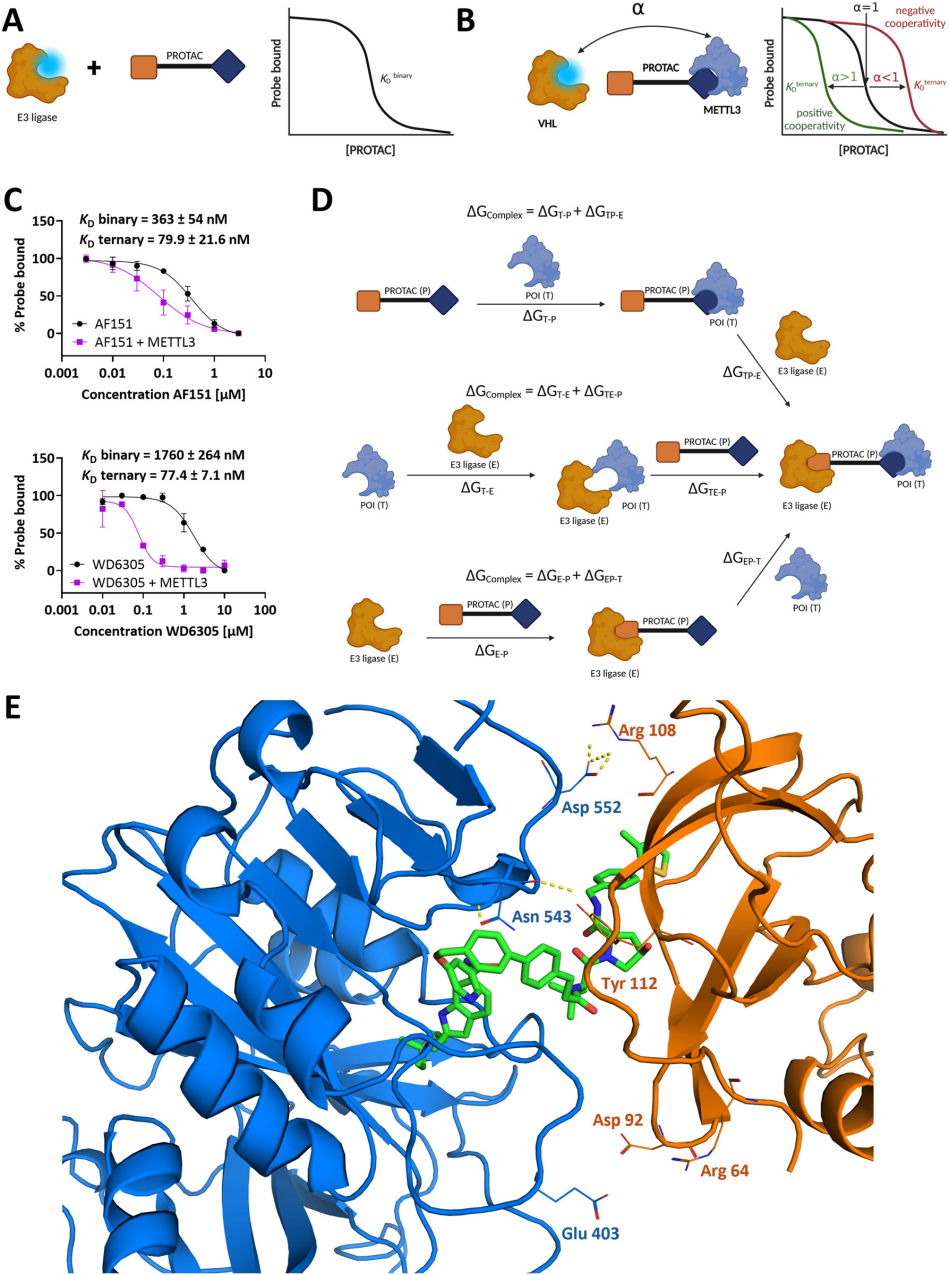
Investigation of ternary complex formation and cooperativity for **AF151** and WD6305. (A and B) A fluorescent displacement probe binds to the E3 ligase and is displaced upon PROTAC molecule binding. The FP level of the displaced probe was used to determine the dissociation constant of PROTAC binding. Cooperativity is evaluated by comparing the PROTAC's affinity for the E3 ligase in the presence and the absence of METTL3. (C) Cooperativity factor (α) determination for **AF151** and WD6305 binding in the presence (purple) and absence (black) of METTL3 (12 μM). Each data point represents the mean ± SEM from triplicates repeated in two independent experiments. (D) Possible binary and ternary interactions between PROTAC (P), E3-ligase (E), and the protein of interest (target, T) can be described using Gibbs free energy (Δ*G*) for each interaction. The overall Gibbs free energy change for the formation of the ternary complex can be determined in three equivalent ways: Δ*G*_complex_ = Δ*G*_T–P_ + Δ*G*_TP–E_ = Δ*G*_E–P_ + Δ*G*_EP–T_ = Δ*G*_T–E_ + Δ*G*_TE–P_. (E) Most stable predicted binding mode for **AF151** in a ternary METTL3-VCB complex. The proteins are portrayed as cartoons, with the amino acids commonly found in protein–protein interactions across all simulations labeled as lines. Polar protein–protein interactions are shown as dashed yellow lines, and **AF151** is shown as green sticks. METTL3: blue, VHL: orange.

To assess the cooperativity of **AF151** and WD6305, we employed a displacement FP assay, previously described to assess PROTAC cooperativity.^38,39^ In this assay, the VHL dissociation constants of both METTL3 PROTACs **AF151** and WD6305, with and without near-saturating METTL3 concentrations, were compared (Fig. 3A and B). Here, both PROTACs exhibited overall positive cooperativity (Fig. 3C), facilitating the interaction between METTL3 and VHL. Interestingly, the cooperativity factor of WD6305 (*α* = 23) is approximately five times higher than that of **AF151** (*α* = 4.5), which could possibly explain the observed differences in their hook effect characteristics (*i.e.*, lower binary affinity of WD6305 disfavors VHL-PROTAC complex formation in the absence of METTL3).

To validate these findings, we used a homogeneous time-resolved fluorescence (HTRF) assay to assess VHL affinity in the presence and absence of METTL3, confirming positive cooperativity for both PROTACs, with WD6305 displaying a higher α than **AF151** (Fig. S5†). Interestingly, the strong positive cooperativity for WD6305 is not due to an increased ternary affinity. The ternary *K*_D_ for **AF151** and WD6305 is very similar at 77 nM and 79 nM, respectively. Instead, the binary affinity of WD6305 for VHL (1.7 μM) is significantly reduced compared to **AF151** (363 nM) or parent compound VH032 (210 nM).

### Computational analysis of METTL3 PROTAC complexes

For further investigation of the ternary complex formed by **AF151** and WD6305, the small molecule-induced protein–protein interface was evaluated using *in silico* PROTAC-docking with subsequent molecular dynamics (MD) simulations. Additionally, KH12, the METTL3 PROTAC from Hwang *et al.*, was included in this analysis, since this PROTAC has a comparable structure to WD6305 but exhibits a hook effect similar to **AF151**.^32^ While PROTAC-docking provides possible geometries of ternary complexes, this coarse-grained method is not capable of discriminating the correct complex structure from incorrect ones, since the scoring function has insufficient accuracy.

Therefore, for each of the three PROTACs, three ternary complex structures were selected based on clustering and score for subsequent MD simulations. After short MD simulations of 20 ns, complex structures were evaluated based on their overall stability by root-mean-square-deviation (RMSD) analysis and complex stability using MM-GBSA calculations (Fig. S6, Table S2†). MM-GBSA was previously shown to be a good predictor for characterizing cooperativity, including stabilization and hook effects.^56^ MD simulations of the most stable complexes per PROTAC, presumably representing the correct complex orientation, were prolonged to 100 ns each prior to MM-GBSA analysis of these effects (Fig. S7, Table S3†). As described previously, simulation times of 50–100 ns were found to be suitable for this analysis.^56^ In these ternary complexes, there were only minor differences in MM-GBSA results between 50 ns and 100 ns, indicating sufficient sampling (Table S3†). Only the complex structure of WD6305 diverged from the starting structure after around 53 ns (Fig. S7C†). As the modeling of ternary PROTAC complexes is still in its infancy, some caveats must be raised. While MM-GBSA provides absolute binding free energy values, it should still be considered a coarse-grained method with limited accuracy. Therefore, instead of interpreting absolute Δ*G*-values, we performed only a qualitative, relative comparison between the ternary complexes. For higher accuracy, computationally more expensive absolute or relative binding free energy calculations (ABFE/RBFE) like free energy perturbations (FEP) or thermodynamic integration (TI) must be performed.^73–76^ Likewise, the 1-trajectory (1A) MM-GBSA approach is a simplification assuming the different affinity contributions can be obtained from a single simulation of the ternary complex instead of seven simulations covering ternary and binary complexes and target, ligase, and PROTAC alone (Fig. 3D). Despite these limitations, some observations from MD simulations reveal trends for the molecules under discussion.

Within the 20 ns of simulation, the structurally most stable complexes (Fig. S6†) also showed stronger favorable binding free energies for the ternary complex (Δ*G*_complex_, Table S2†). METTL3/14 and VCB were internally very stable, while structural changes mostly occurred in the whole complex, hence the orientation between target and ligase.

The similar geometry of the complexes with the highest stability used for prolonged MD simulations of 100 ns hints towards the identification of the putatively correct geometry of the complexes (Fig. S7†). MM-GBSA analysis (Table S3†) showed a higher affinity of the PROTAC towards METTL3/14 (Δ*G*_T–P_) than to VCB (Δ*G*_E–P_) in line with FP binding assays. Further, all compounds show a stabilizing effect (positive cooperativity) caused by favorable METTL3/14-VCB (target–E3 ligase, Δ*G*_T–E_) interactions. Without PROTAC, Δ*G*_T–E_ ∼ 0 kcal mol^−1^, strong protein–protein interactions should have stabilizing effects for ternary complex formation.

This is reflected in the predicted cooperativity (Δ*G*_TP–E_ – Δ*G*_E–P_, Table S3†). Based on the predicted METTL3/14–**AF151**–VCB complex (Fig. 3E) and Δ*G*_T–E_ contribution from MM-GBSA, mainly hydrophobic contacts contribute to these direct target–ligase interactions. Additionally, some polar interactions are commonly found in the simulations, *e.g.*, Asp552 (METTL3) + Arg108 (VHL), Asn543 (METTL3) + Tyr112 (VHL), and Glu403 (METTL3) + Arg64 (VHL) (Fig. 3E). Hook effects can occur for PROTACs with high affinity to the target or ligase while having low or negative cooperativity. For KH12, the predicted affinity to both METTL3/14 (Δ*G*_T–P_) and VCB (Δ*G*_E–P_) is higher than for **AF151** and WD6305, which might explain the observed hook effect for this compound.^32^

However, this cannot explain the difference between **AF151** (hook effect) and WD6305 (no hook effect at given concentrations), as predicted affinities for METTL3/14 and VCB (Δ*G*_T–P_ and Δ*G*_E–P_, respectively) are similar, while the predicted stabilizing effect for **AF151** (Δ*G*_T–E_) is even higher. Notably, the simulations were performed considering only one possible geometry of the ternary complex, whereas *in vivo* and *in vitro*, different conformations of the complex might form, some of which could lead to less stable interactions.^77^ Yet, ternary complex formation is crucial but insufficient by itself for protein degradation since ubiquitination and other cellular mechanisms also play an important role. Some of these alternative conformations possibly result in unproductive complexes.^78,79^ Ultimately, the MD simulations reason that the differing degradation profile of **AF151** and WD6305 is not due to overall cooperativity differences and binding mode variation, but raises the hypothesis that one of the other effects influencing cellular degradation might be the reason for **AF151**'s hook effect.

### Investigation of cellular response after **AF151** treatment

**Fig. 4:**
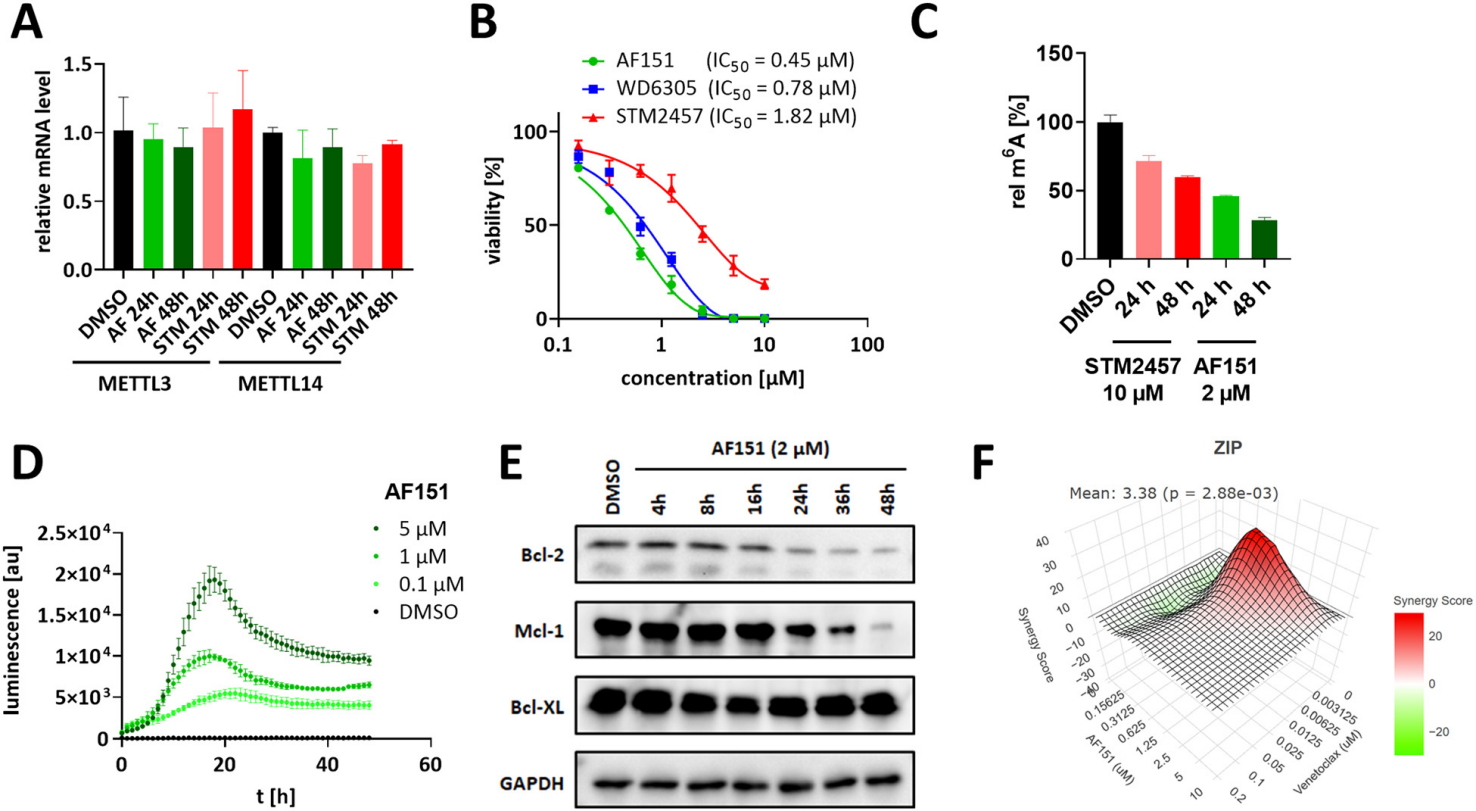
Evaluation of **AF151** in MOLM-13 cells. (A) Relative METTL3 and METTL14 mRNA levels (ΔΔCt) after treatment with **AF151** (2 μM) or STM2457 (10 μM) for 24 h resp. 48 h. (B) Cell viability inhibition after treatment with varying concentrations of **AF151**, WD6305, and STM2457. (C) m^6^A quantification of total mRNA isolated from MOLM-13 cells after treatment with **AF151** (2 μM) or STM2457 (10 μM) for the indicated durations. (D) Real-time apoptosis assay (homogeneous luminogenic annexin V binding assay). MOLM-13 cells were treated with **AF151** at the indicated concentrations, and apoptosis was monitored *via* luminescence over 48 h. (E) Time course treatment experiment of Bcl-like proteins performed analogously to Fig. 2D. Western blot image after treatment with **AF151** at 2 μM at the indicated time points. The corresponding time-response plot can be found in Fig. 2D. (F) Zero interaction potency (ZIP) analysis of co-treatment (72 h) with **AF151** and venetoclax in MOLM-13 cells. Quantification and illustration were generated with the SynergyFinder+ webserver.

Overall, **AF151** is able to form a stable ternary complex between VHL and METTL3, exhibiting positive cooperativity and effectively reducing METTL3 levels in MOLM-13 cells in a concentration-, time-, and UPS-dependent manner. To assess the cell biological effects of **AF151**, we investigated the cellular downstream responses of MOLM-13 cells following treatment with **AF151**. First, we evaluated METTL3 and METTL14 mRNA levels *via* RT-qPCR and found no significant changes in gene expression following **AF151** or STM2457 treatment after 24 h and 48 h (Fig. 4A), thereby verifying that the alteration of METTL3 and METTL14 protein levels by **AF151** is due to degradation rather than inhibited protein expression or feedback stimulation of RNA synthesis. This is in agreement with literature experiments conducted for the WD6305 degrader.^31^

Parent compound STM2457 has been shown to suppress the methyltransferase activity of METTL3, leading to reduced m^6^A levels and antiproliferative effects on AML cells.^16^ Thus, we examined whether the compound-induced degradation of METTL3 similarly resulted in reduced cell viability. For comparison, we determined the reduction in MOLM-13 cell viability caused by **AF151**, WD6305, and STM2457. **AF151** (IC_50_ = 0.45 μM) exhibited a similar reduction in cell viability as WD6305 (IC_50_ = 0.78 μM) and fourfold more efficient cellular inhibition than parent compound STM2457 (IC_50_ = 1.82 μM) (Fig. 4B). This agrees with previous reports that the degradation of METTL3 provides a greater benefit in suppressing cell viability, and METTL3 degraders effectively promote apoptosis in cancer cell lines.^31–33^ Next, we compared the effect of **AF151** and STM2457 on global m^6^A levels in MOLM-13 cell total mRNA, finding that **AF151** caused a more pronounced m^6^A reduction than the competitive inhibitor STM2457 alone (Fig. 4C).

METTL3 has been shown to regulate the translation of the anti-apoptotic protein Bcl-2 in cancer cells, and METTL3 inhibition using STM2457 leads to a reduction in Bcl-2 protein levels in AML.^16,80^ Thus, we evaluated whether the degradation of METTL3 by **AF151** would lead to a decrease in Bcl-2 protein levels. Analysis of the MOLM-13 treatment time course showed that Bcl-2 protein levels decreased alongside METTL3 (Fig. 2D), albeit with slower kinetics (*t*_1/2_ = 18 h), likely due to a delay in the reduction of METTL3-mediated RNA modifications on Bcl-2 mRNA (Fig. 4E). Recently, Jiao *et al.* demonstrated that inhibition of METTL3 by STM2457 leads to a decrease in Mcl-1 levels in AML cells, another antiapoptotic protein and member of the Bcl-2 family.^81^ Thus, we evaluated the impact of METTL3 degradation by **AF151** on Mcl-1 levels. Additionally, we tested the effect on Bcl-XL, another Bcl-2 family member, whose protein levels, however, were not affected by METTL3 inhibition in the study by Jiao *et al.* In summary, we were able to reproduce these results: **AF151** leads to a time-dependent reduction of Bcl-2 and Mcl-1 but not Bcl-XL (Fig. 4E).

Next, we used a homogeneous luminogenic annexin V binding assay to follow MOLM-13 apoptosis levels and progressions in real time, revealing that **AF151** induces apoptosis after approximately 10 h in a dose-dependent manner (Fig. 4D), which is consistent with both the reduction in cell viability and the kinetics of Bcl-2 level reduction. Jiao *et al.* showed that STM2457 synergistically enhances the antileukemic efficacy of the Bcl-2 inhibitor venetoclax and can overcome venetoclax resistance in *in vivo* experiments and resistance models. Hence, we hypothesized that co-treatment with a METTL3 degrader and venetoclax could equally lead to synergistic effects on cell viability reduction.

To test this hypothesis, we treated MOLM-13 cells with varying concentrations of **AF151** and venetoclax (Fig. 4F). The matrix of cell viability reduction was analyzed using the zero interaction potency (ZIP) model and quantified with the SynergyFinder method.^57,82^ Co-treatment with **AF151** and venetoclax exhibited a slight synergistic reduction in cell viability, predominantly at lower concentrations of venetoclax (mean ZIP = 3.38, maximum ZIP = 28.87; Fig. 4F), *i.e.*, **AF151** is sensitizing MOLM-13 cells for treatment with venetoclax as the apoptosis-inducing effector, possibly by downregulation of Bcl-2 family proteins. Altogether, targeted degradation of METTL3 seems to be an effective strategy against AML and exhibits enhanced efficacy when combined with other agents.

## Conclusions

In this work, we developed and characterized **AF151**, a functional METTL3 degrader, in MOLM-13 cells. In-depth characterization proved that **AF151** is an effective and rapid degrader of METTL3, utilizing the UPS. Our PROTAC candidates were developed across four design generations, including the evaluation of target affinities (*via* FP assays) and degradation potency (*via* immunoblotting). In this regard, we explored new chemotypes for METTL3 PROTAC development, offering a structurally distinct alternative to current UZH2-based PROTACs.^30–33^ During our development process, we demonstrated that targeting the E3 ligase VHL is preferable to CRBN. Moreover, strategic improvements in METTL3 affinity, exit vector orientation, and linker flexibility were found to be essential. These insights and design guidelines will be a useful resource for following METTL3 PROTAC discovery campaigns.

In the course of this study, we have established an FP-based assay to assess binary METTL3 PROTAC affinities and utilized a literature-reported FP assay for binary and ternary VHL PROTAC interactions. Additionally, we performed MD simulations to analyze the ternary complex formation and cooperativity between METTL3 PROTACs and VHL. In this regard, we noticed the interesting finding that the potent published METTL3 PROTAC WD6305 has a reduced VHL affinity compared to the parent compound VH032, and this generates its strong positive cooperativity and efficient degradation profile.

Overall, we highlighted key design considerations when developing PROTACs targeting METTL3 and proved that an alternative METTL3-binding chemotype, distinct from previously published structures, can lead to the efficient degradation of METTL3. These degradation results, in conjunction with biophysical characterization of the PROTAC-mediated complexes, expand the toolbox for the development of future targeted METTL3 degraders.

## Author contributions

A. C. W.: data curation, formal analysis, investigation, validation, visualization, writing – original draft, A. F. F.: formal analysis, investigation, writing – original draft, M. W. K.: methodology, resources. FK: investigation. HK: investigation. CK: formal analysis, investigation, writing – original draft. FB: conceptualization, methodology, project administration, writing – review & editing.

## Conflicts of interest

There are no conflicts of interest to declare.

## Supplementary Material

MD-OLF-D5MD00359H-s001

## Data Availability

The data supporting this article have been included as part of the ESI.†
