# Supplementary material for: Structure-guided design of a methyltransferase-like 3 (METTL3) proteolysis targeting chimera (PROTAC) incorporating an indole–nicotinamide chemotype
Source: RSC Med Chem. 2025 Jun 19;16(9):4194–207. doi: 10.1039/d5md00359h (PMC12207533; doi:10.1039/d5md00359h)
Supplement: MD-016-D5MD00359H-s001 [file MD-016-D5MD00359H-s001.pdf]

Supplementary Information (SI) for RSC Medicinal Chemistry.
This journal is © The Royal Society of Chemistry 2025

Structure-guided Design of a Methyltransferase-like 3 (METTL3) Proteolysis Targeting Chimera (PROTAC) Incorporating an Indole-Nicotinamide Chemotype

Annabelle C. Weldert[a], Ariane F. Frey[a], Mackenzie W. Krone[b], Franziska Krähe[a], Hannah Kuhn[a], Christian Kersten[a,c], Fabian Barthels[a,b]*

[a]Institute of Pharmaceutical and Biomedical Sciences, Johannes Gutenberg-University, Staudingerweg 5, 55128 Mainz, Germany.

[b]Department of Molecular, Cellular, and Developmental Biology, Yale University, 260 Whitney Ave, CT 06520-8103 New Haven, USA.

[c]Institute for Quantitative and Computational Biosciences, Johannes Gutenberg-University, BioZentrum I, Hanns-Dieter-Hüsch Weg 15, 55128 Mainz, Germany.

* E-Mail: barthels@uni-mainz.de

Table of contents

Biophysical investigation of PROTAC candidates 2

Supplementary FP and HTRF assay dose-response curves 7

Supplementary Western blots (screening results) 9

Supplementary data on biophysical investigation of ternary complexes 10

Molecular dynamics simulations 11

Primer sequences used for RT-qPCR 13

Uncropped Western blot images 14

References 15

Synthesis of E3 ligase recruiters with linker 16

VHL-RH-based recruiters 16

VHL-LH-based recruiters 18

4-Thalidomide-based recruiters 21

5-Thalidomid-based recruiters 23

VHL-LH building blocks for Suzuki and Sonogashira reactions 26

1st and 2nd generation METTL3 recruiter synthesis 28

Synthesis of 1st generation PROTACs 31

Synthesis of 2nd generation PROTACs 38

Synthesis of CRBN-based PROTACs 47

Synthesis of 3rd generation PROTACs 54

Synthesis of 4th generation PROTACs 57

Spectral appendix 62

Supporting results & discussion

Biophysical investigation of PROTAC candidates

Table S1: Binary affinities to METTL3 were determined for all PROTACs synthesized in this work via FP assays. Selected affinities to the respective E3 ligase were determined via HTRF and FP assays. All K_D values include the mean value and standard deviations from at least technical triplicate measurements. Dose-response curves are depicted in Figures S1–S3 and S5.

Cpd No.	METTL3 recruiter	Linker	E3 ligase recruiter	K_D METTL3 [µM]	K_D E3-ligases [µM]
**5**	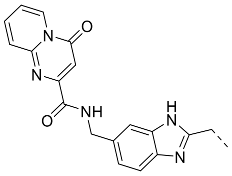 **POI-1**	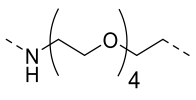	none	10.38 ± 0.98	n.d.
**6**	**POI-1**	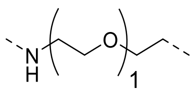	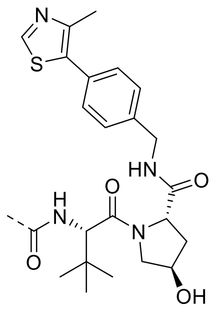 VHL-LH	>100	n.d.
**7**	**POI-1**	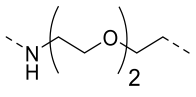	VHL-LH	12.47 ± 1.34	n.d.
**8**	**POI-1**	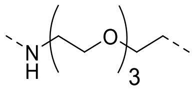	VHL-LH	16.07 ± 1.55	n.d.
**9**	**POI-1**	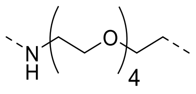	VHL-LH	37.59 ± 8.73	n.d.
**10**	**POI-1**	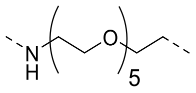	VHL-LH	16.33 ± 1.44	n.d.
**11**	**POI-1**	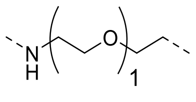	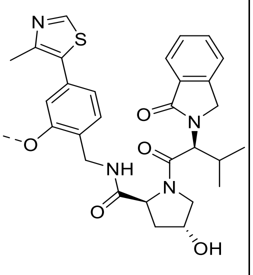 VHL-RH	>100	n.d.

**12**	**POI-1**	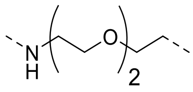	VHL-RH	4.41 ± 1.25	n.d.
**13**	**POI-1**	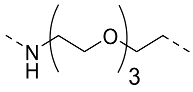	VHL-RH	3.94 ± 0.53	n.d.
**14**	**POI-1**	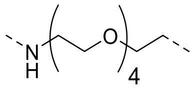	VHL-RH	7.50 ± 1.07	n.d.
**15**	**POI-1**	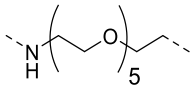	VHL-RH	5.15 ± 0.59	n.d.
**16**	**POI-1**	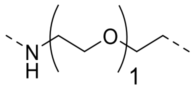	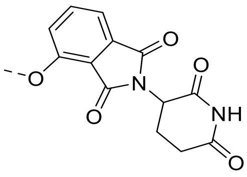 4-Thalidomide	8.06 ± 1.72	n.d.
**17**	**POI-1**	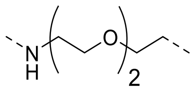	4-Thalidomide	10.58 ± 1.17	n.d.
**18**	**POI-1**	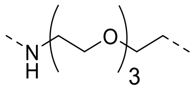	4-Thalidomide	26.51 ± 2.05	n.d.
**19**	**POI-1**	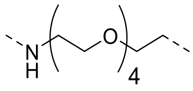	4-Thalidomide	10.58 ± 3.00	n.d.
**20**	**POI-1**	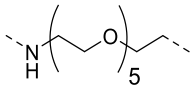	4-Thalidomide	6.13 ± 1.36	2.98 ± 0.23
**21**	**POI-1**	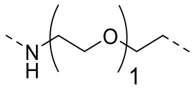	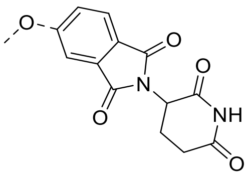 5-Thalidomide	>100	n.d.
**22**	**POI-1**	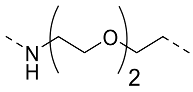	5-Thalidomide	6.44 ± 0.54	2.20 ± 0.14

**23**	**POI-1**	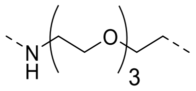	5-Thalidomide	7.27 ± 0.89	n.d.
**24**	**POI-1**	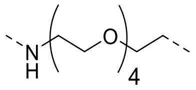	5-Thalidomide	19.55 ± 2.85	n.d.
**25**	**POI-1**	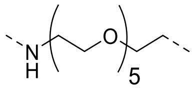	5-Thalidomide	31.87 ± 9.06	n.d.
**26**	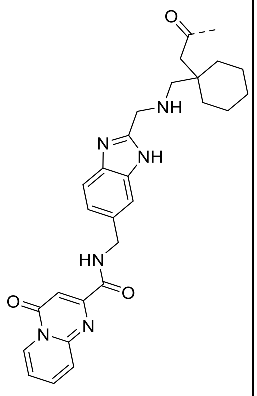 **POI-2**	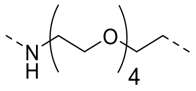	none	0.433 ± 0.056	n.d.
**27**	**POI-2**	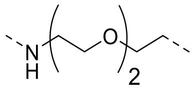	VHL-LH	0.429 ± 0.046	n.d.
**28**	**POI-2**	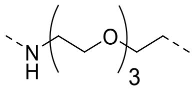	VHL-LH	0.309 ± 0.022	0.145 ± 0.04
**29**	**POI-2**	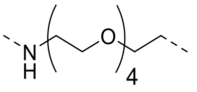	VHL-LH	1.53 ± 0.10	n.d.
**30**	**POI-2**	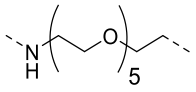	VHL-LH	0.725 ± 0.079	n.d.
**31**	**POI-2**	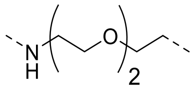	VHL-RH	1.15 ± 0.10	n.d.
**32**	**POI-2**	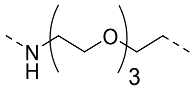	VHL-RH	2.05 ± 0.32	0.222 ± 0.05
**33**	**POI-2**	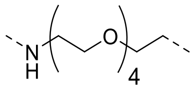	VHL-RH	2.04 ± 0.46	n.d.
**34**	**POI-2**	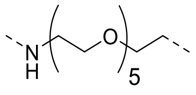	VHL-RH	2.49 ± 0.55	n.d.

**35**	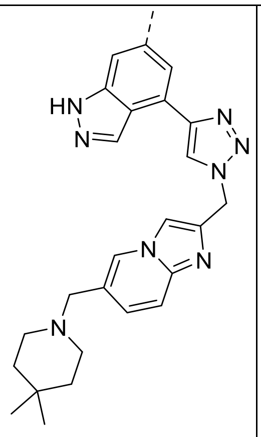 **POI-3**	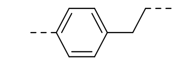	VHL-LH	0.263 ± 0.015	n.d.
**36**	**POI-3**	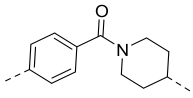	VHL-LH	0.223 ± 0.045	n.d.
**37**	**POI-3**	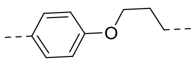	VHL-LH	2.48 ± 0.392	n.d.
**38**	**POI-3**	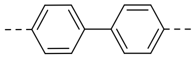	VHL-LH	n.d.*	n.d.
**39**	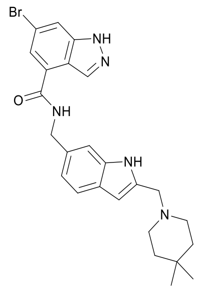	none	none	0.0542 ± 0.0066	n.d.
**40**	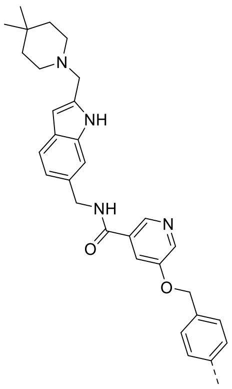 **POI-4**	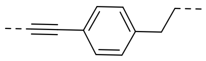	VHL-LH	0.158 ± 0.019	n.d.
**41 (AF151)**	**POI-4**	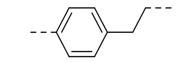	VHL-LH	0.159 ± 0.026	0.363 ± 0.054 (FP) 0.740 ± 0.113 (HTRF)

**42**	**POI-4**	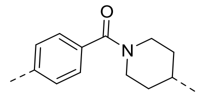	VHL-LH	0.295 ± 0.050	n.d.
WD6305[1]	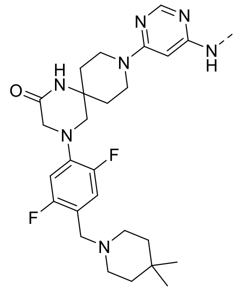	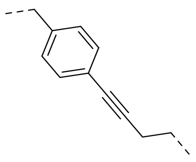	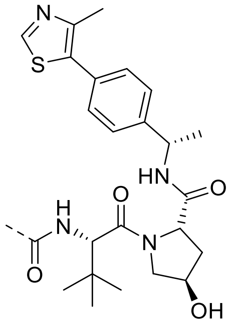	0.096 ± 0.001	1.76 ± 0.26 (FP) 2.17 ± 0.28 (HTRF)

*The affinity could not be determined due to fluorescence interference of the biphenyl moiety with FP setup.

Supplementary FP and HTRF assay dose-response curves

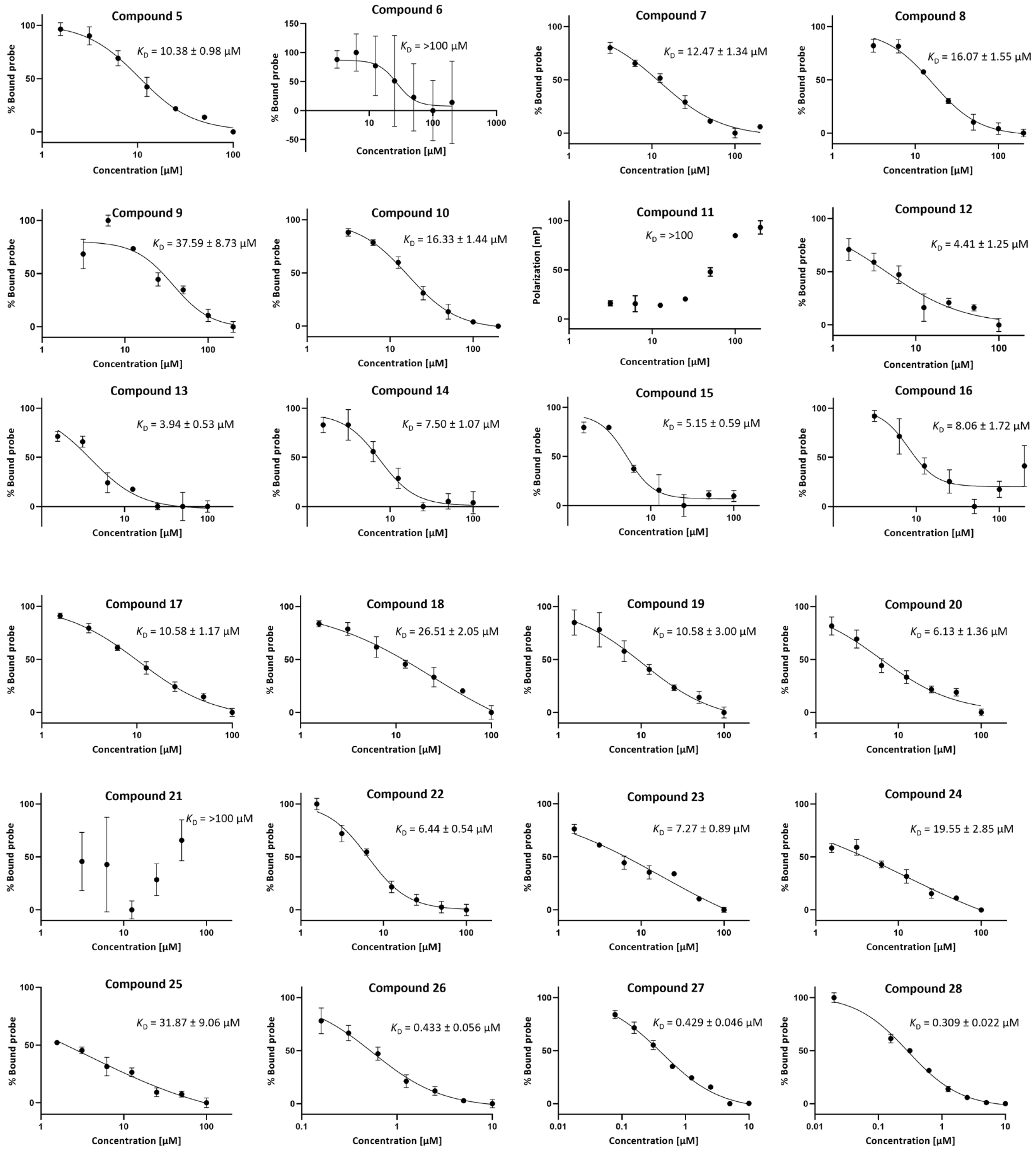

Figure S1: Supplementary FP assay dose-response curves of compounds **5**–**28**. Each experiment was carried out in triplicate. The ligand was titrated to 300 nM METTL3/14 and 20 nM of the displacement probe (STM-FL[2]) to determine the apparent dissociation constant, as specified in the experimental section. Compound **11** led to the visible formation of aggregates; thus, the distorted dose-response curve was not evaluated by non-linear regression.

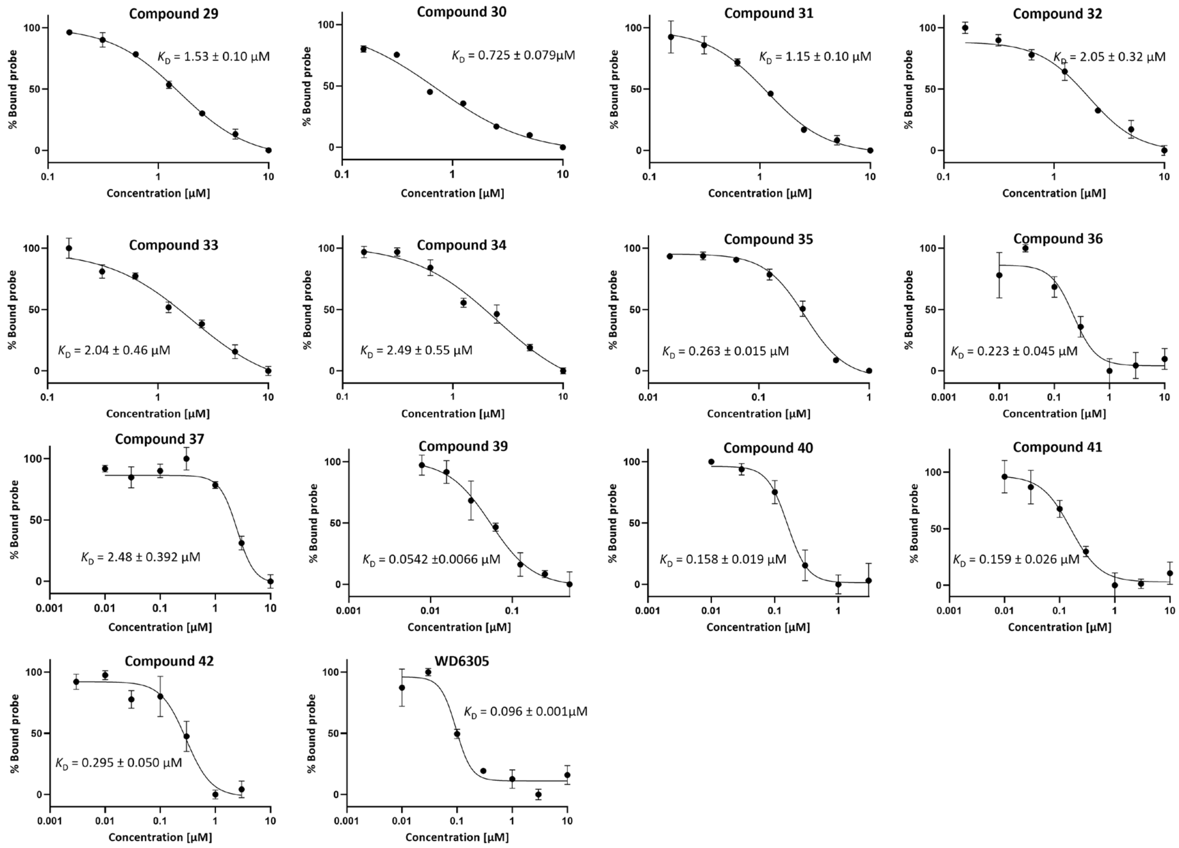

Figure S2: Supplementary FP assay dose-response curves of compounds **29**–**42** and WD6305[1]. Each experiment was carried out in triplicate. The ligand was titrated to 300 nM METTL3/14 and 20 nM of the displacement probe (STM-FL[2]) to determine the apparent dissociation constant, as specified in the experimental section.

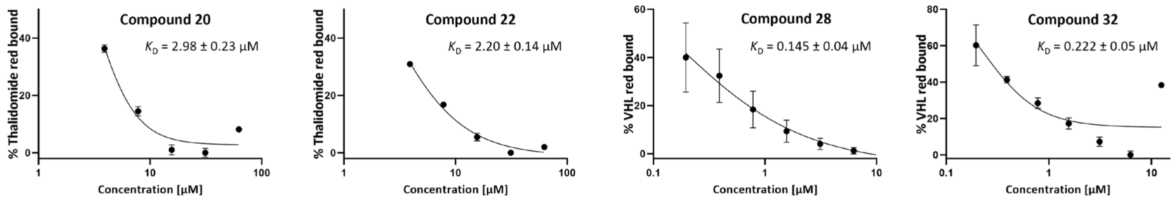

Figure S3: Supplementary Time-Resolved Fluorescence (HTRF) assay results for compounds **20**, **22**, **28**, and **32.** Each experiment was carried out in triplicate. The ligand PROTACs were titrated using an HTRF Human VHL Binding Kit (Revvity) or an HTRF Human Cereblon Binding Kit (Revvity) to determine the apparent dissociation constant, as specified in the experimental section.

Supplementary Western blots (screening results)

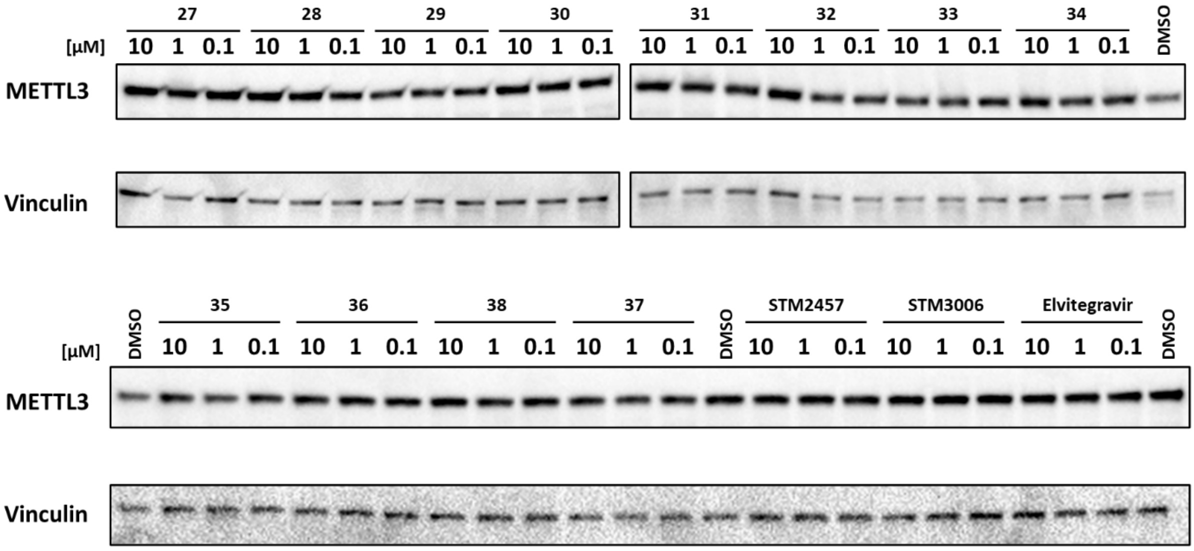

Figure S4: Western blot screening results of PROTAC candidates from the 2nd and the 3rd generation designs. MOLM-13 cells were treated with the indicated compounds at varying concentrations (10 µM, 1 µM, and 0.1 µM) for 16 h. DMSO was used as a negative control. None of the compounds showed significant degradation of METTL3. The METTL3 inhibitors STM2457[3] (Cayman Chemicals), STM3006[4] (BLDpharm), and Elvitegravir[5] (MedChemExpress), were used as conditional controls to prove that competitive METTL3 inhibitors do not function as METTL3 degraders. Shown is a representative Western blot image of two independent experiments.

Supplementary data on biophysical investigation of ternary complexes

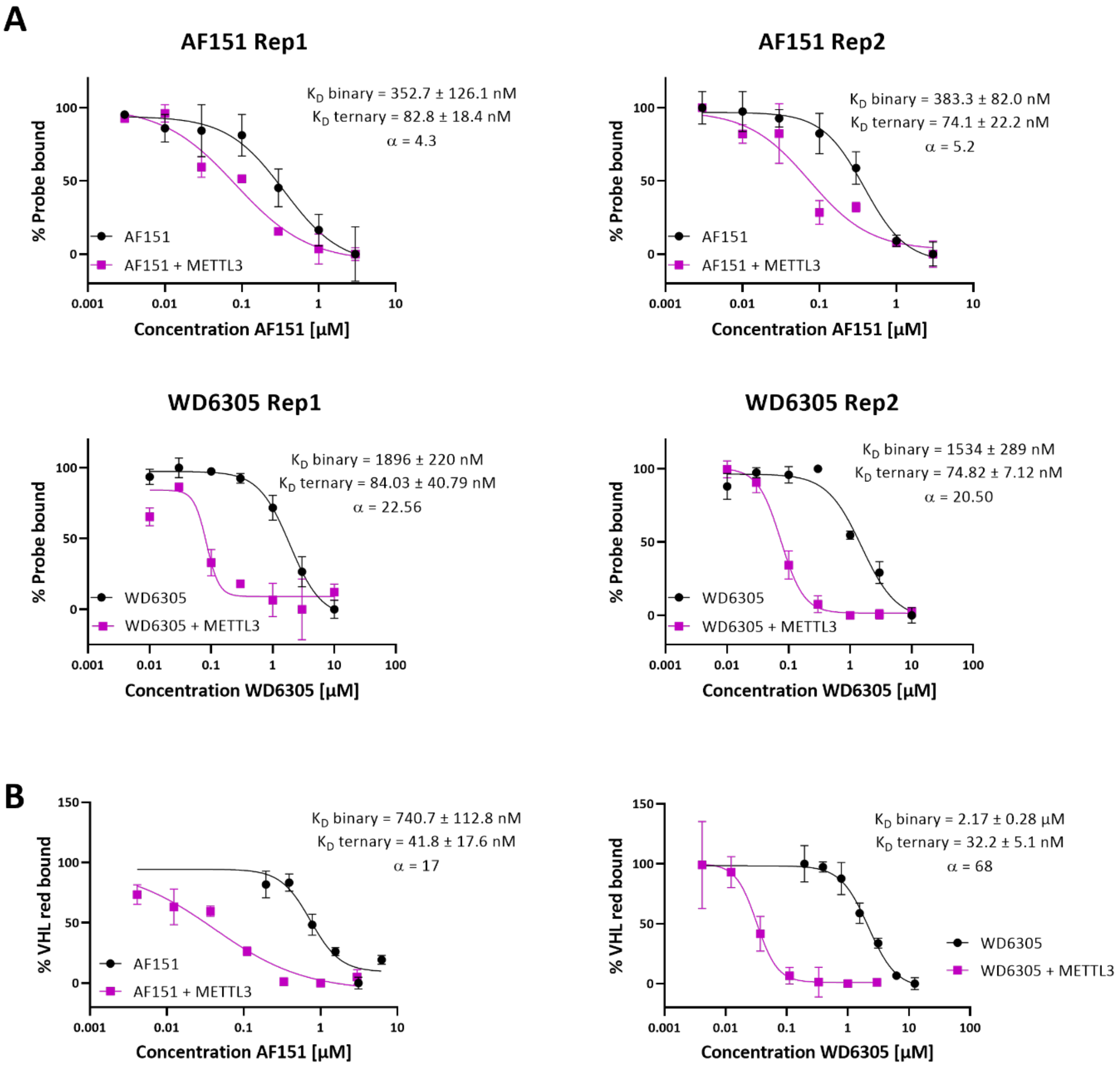

Figure S5. Supplementary data for ternary complex investigation. (**A**) FP dose-response curves for ternary affinity determination of **AF151** and WD6305. Each experiment was carried out in triplicate on two non-consecutive days (Rep1 & Rep2). PROTACs were titrated to 40 nM VCB complex and 10 nM displacement probe to determine the apparent dissociation constant, as specified in the experimental section. For ternary K_D determinations, METTL3/14 was added in near-saturating concentrations relative to the highest PROTAC concentration. (**B**) Homogeneous Time-Resolved Fluorescence (HTRF) assay results for **AF151** and WD6305. Each experiment was carried out in triplicate. The ligand PROTACs were titrated using an HTRF Human VHL Binding Kit (Revvity) to determine the apparent dissociation constant, as specified in the experimental section. For ternary K_D determinations, METTL3/14 was added in near-saturating concentrations relative to the highest PROTAC concentration.

Molecular dynamics simulations

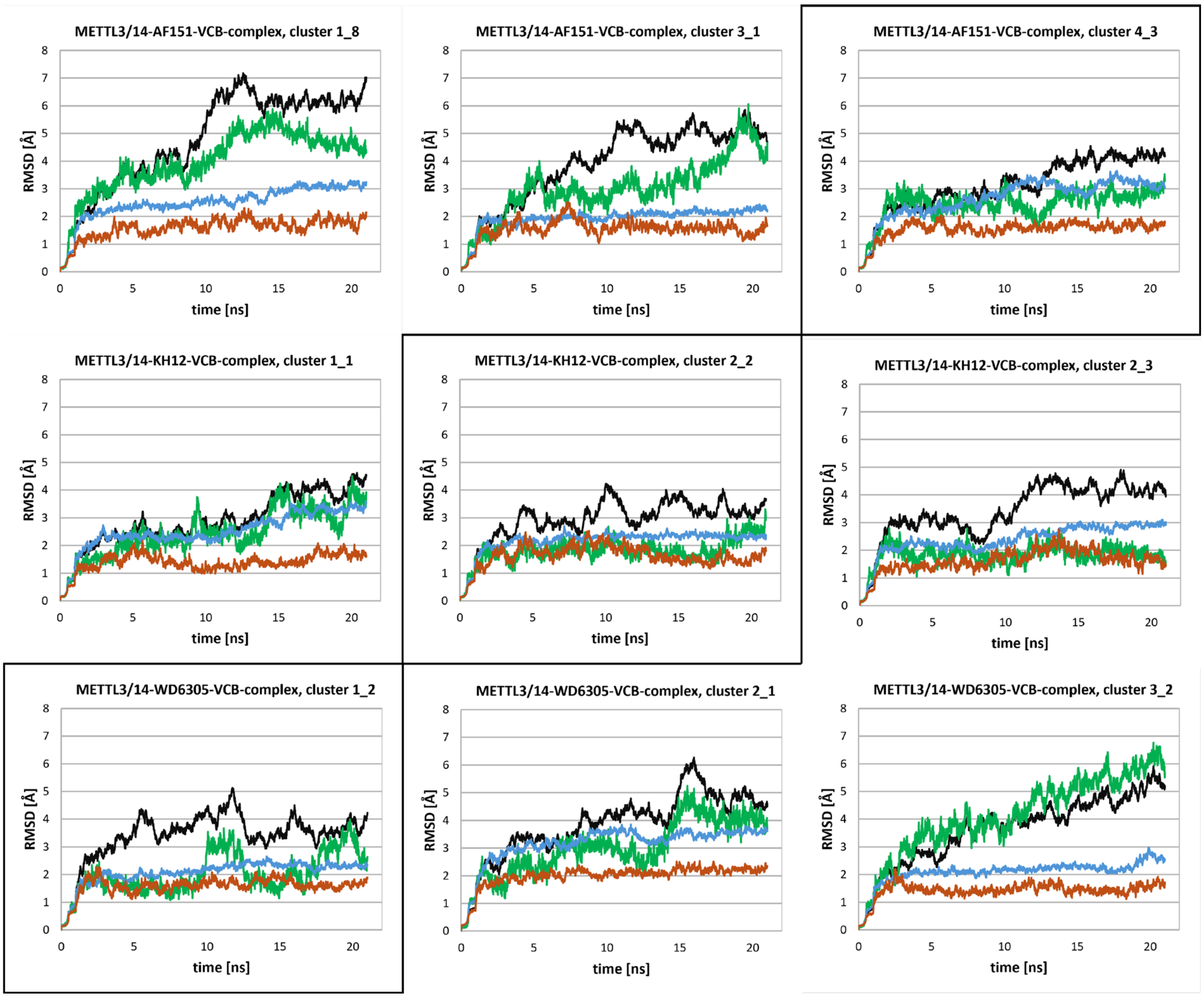

Figure S6: MD simulation RMSD-analysis of ternary METTL3/14-PROTAC-VCB complexes over 1 ns equilibration and 20 ns production. PROTACs **41** (**AF151**), KH12[6] and WD6305[1] were investigated. Complex protein backbone RMSD in Å is depicted in black, METTL3/14 in blue, VCB in orange, and ligand heavy atoms (non-hydrogen) in green. For complex and ligand RMSD, aligned complexes were evaluated, while for internal stability of METTL3/14 and VCB, alignment was performed on the respective target or VCB part. The best-scoring cluster representatives of ternary structures from PROTAC-docking were used as starting structures for MD simulations. In WD6305-complex cluster 1_2, the increased ligand RMSD values are caused by a flip of the phenyl linker. Structures for which MD simulations were prolonged to 100 ns production (Figure S7) are highlighted by a black border.

Table S2: 1A-MM-GBSA analysis of PROTAC-docking generated ternary complexes from 20 ns MD simulations. T: target (METTL3/14), P: PROTAC, E: E3 ligase (VCB). Binding free energy of ternary complex ΔGcomplex = $\Delta G_{T\text{-}P} + \Delta G_{TP\text{-}E} = \Delta G_{E\text{-}P} + \Delta G_{EP\text{-}T} = \Delta G_{T\text{-}E} + \Delta G_{TE\text{-}P}$ according to main manuscript Figure 3D. MM-GBSA scores are in kcal/mol.

	**AF151 1_8**	**AF151 3_1**	**AF151 4_3**	**KH12 1_1**	**KH12 2_2**	**KH12 2_3**	**WD6305 1_2**	**WD6305 2_1**	**WD6305 3_2**
**$\Delta G_{TE\text{-}P}$**	-129.2	-136.2	-143.1	-150.5	-170.1	-154.7	-152.5	-161.1	-167.1
**$\Delta G_{TP\text{-}E}$**	-68.3	-62.9	-72.3	-66.6	-67.5	-71.9	-70.3	-47.9	-61.6
**$\Delta G_{EP\text{-}T}$**	-89.7	-92.0	-120.0	-123.2	-121.4	-111.6	-116.1	-100.9	-124.9
**$\Delta G_{T\text{-}E}$**	-9.6	-5.5	-18.8	-13.6	-3.6	-9.2	-12.5	+9.7	-6.1
**$\Delta G_{T\text{-}P}$**	-70.5	-62.9	-89.6	-97.5	-106.1	-91.9	-94.7	-103.6	-111.6
**$\Delta G_{E\text{-}P}$**	-49.1	-49.6	-41.9	-41.0	-52.2	-52.2	-48.9	-50.5	-48.3
**$\Delta G_{complex}$**	-138.8	-141.7	-161.9	-164.1	-173.6	-163.9	-165.0	-151.4	-173.2

Table S3: 1A-MM-GBSA analysis of ternary complexes from 50 ns and 100 ns MD simulations, respectively. T: target (METTL3/14), P: PROTAC, E: E3 ligase (VCB). Binding free energy of ternary complex ΔGcomplex = $\Delta G_{T\text{-}P} + \Delta G_{TP\text{-}E} = \Delta G_{E\text{-}P} + \Delta G_{EP\text{-}T} = \Delta G_{T\text{-}E} + \Delta G_{TE\text{-}P}$ according to main manuscript Figure 3D. MM-GBSA scores are in kcal/mol.

	**50 ns**			**100 ns**		
	**AF151 4_3**	**KH12 2_2**	**WD6305 1_2**	**AF151 4_3**	**KH12 2_2**	**WD6305 1_2**
**$\Delta G_{TE\text{-}P}$**	-146.4	-167.1	-146.1	-147.1	-166.6	-147.9
**$\Delta G_{TP\text{-}E}$**	-77.8	-67.9	-64.4	-77.3	-67.5	-70.3
**$\Delta G_{EP\text{-}T}$**	-123.4	-119.7	-105.7	-122.2	-118.1	-111.3
**$\Delta G_{T\text{-}E}$**	-21.5	-4.7	-8.2	-20.3	-4.3	-13.1
**$\Delta G_{T\text{-}P}$**	-90.1	-103.9	-90.0	-90.1	-103.3	-90.7
**$\Delta G_{E\text{-}P}$**	-44.5	-52.1	-48.7	-45.3	-52.7	-49.7
**$\Delta G_{TP\text{-}E} - \Delta G_{E\text{-}P}$**	-33.3	-15.8	-15.7	-32.1	-14.8	-20.6
**$\Delta G_{complex}$**	-167.9	-171.8	-154.4	-167.4	-170.8	-161.0

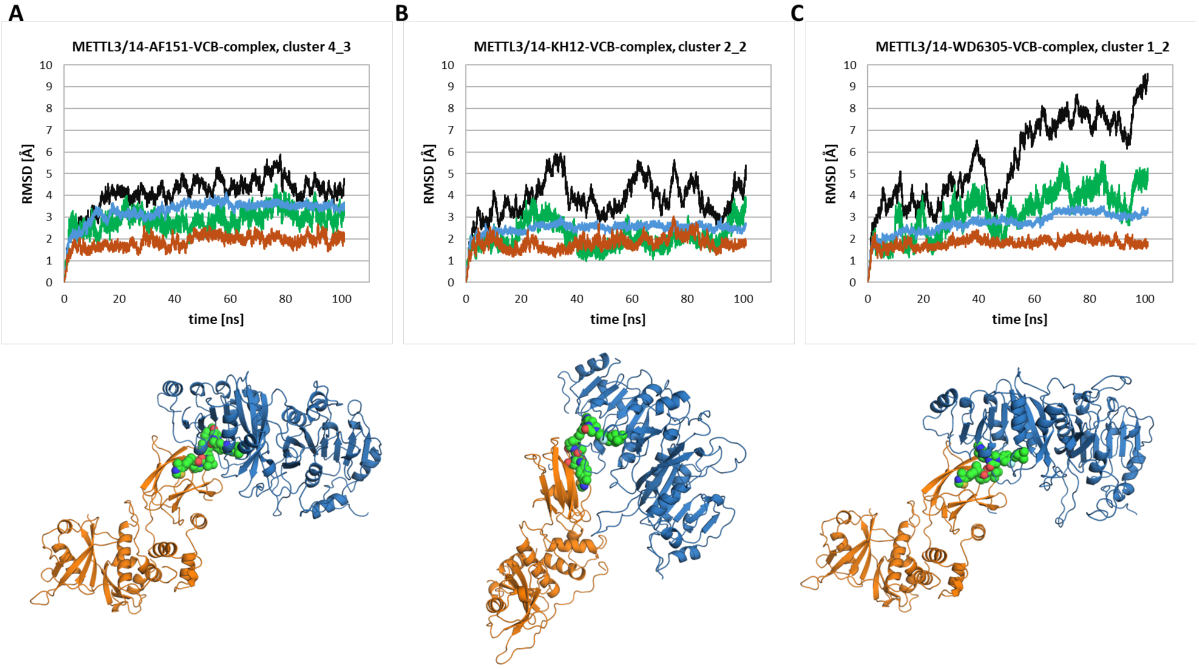

Figure S7: MD-based RMSD-analysis of ternary METTL3/14-PROTAC-VCB complexes over 1 ns equilibration and 100 ns production. Complex protein backbone RMSD in Å is depicted in black, METTL3/14 in blue, VCB in orange, and ligand heavy atoms (non-hydrogen) in green. For complex and ligand RMSD in Å, aligned complexes were evaluated, while for internal stability of METTL3/14 and VCB, alignment was performed on the respective target or VCB part. (**A**) prolonged simulation of **AF151-**complex, cluster 4_3, (**B**) prolonged simulation of KH12-complex, cluster 2_2, (**C**) prolonged simulation of WD6305-complex, cluster 1_2. The starting structures are depicted below the RMSD traces. METTL3/14 blue, VCB orange, and PROTAC carbon atoms green.

Primer sequences used for RT-qPCR

Table S4. Primers used for quantitative reverse transcription polymerase chain reaction.

Gene	Forward Primer	Reverse Primer
METTL3	TTGTCTCCAACCTTCCGTAGT	CCAGATCAGAGAGGTGGTGTAG
METTL14	GAACACAGAGCTTAAATCCCCA	TGTCAGCTAAACCTACATCCCTG
GAPDH	GGAGCGAGATCCCTCCAAAAT	GGCTGTTGTCATACTTCTCATGG

Uncropped Western blot images

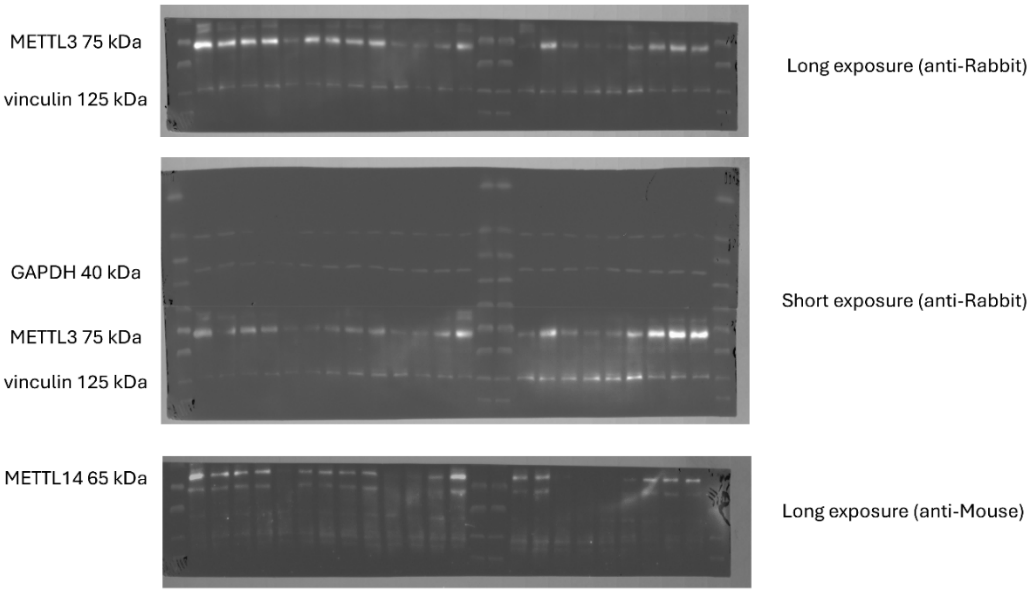

Figure S8: Uncropped images of Figure 2B,C: All data originates from a single blot cut between GAPDH (40 kDa) and METTL3 (75 kDa). For METTL14 ECL detection, the METTL3-detected slice was incubated with mouse anti-METTL14 antibody and anti-Mouse-HRP after the inactivation of anti-Rabbit-HRP with 0.05% sodium azide.

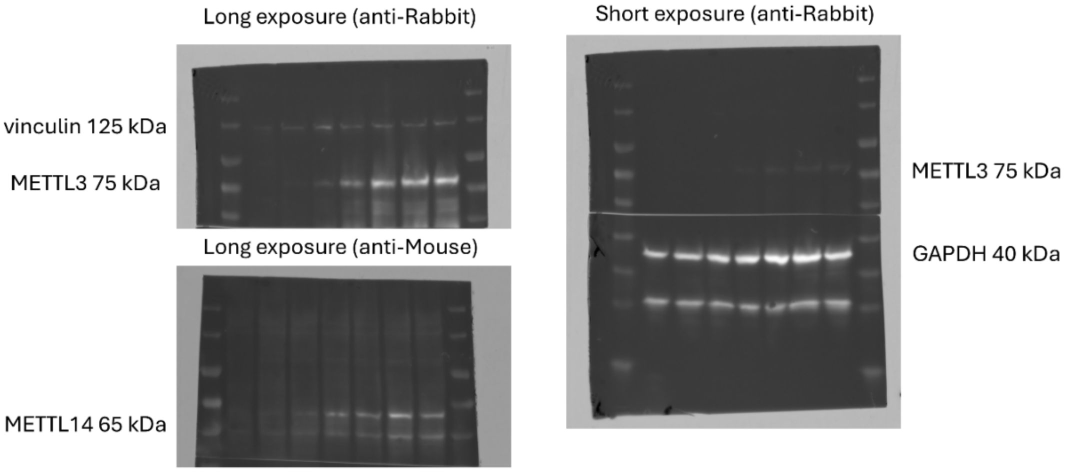

Figure S9: Uncropped images of Figure 2D: All data originates from a single blot cut between GAPDH (40 kDa) and METTL3 (75 kDa). For METTL14 ECL detection, the METTL3-detected slice was incubated with mouse anti-METTL14 antibody and anti-Mouse-HRP after the inactivation of anti-Rabbit-HRP with 0.05% sodium azide.

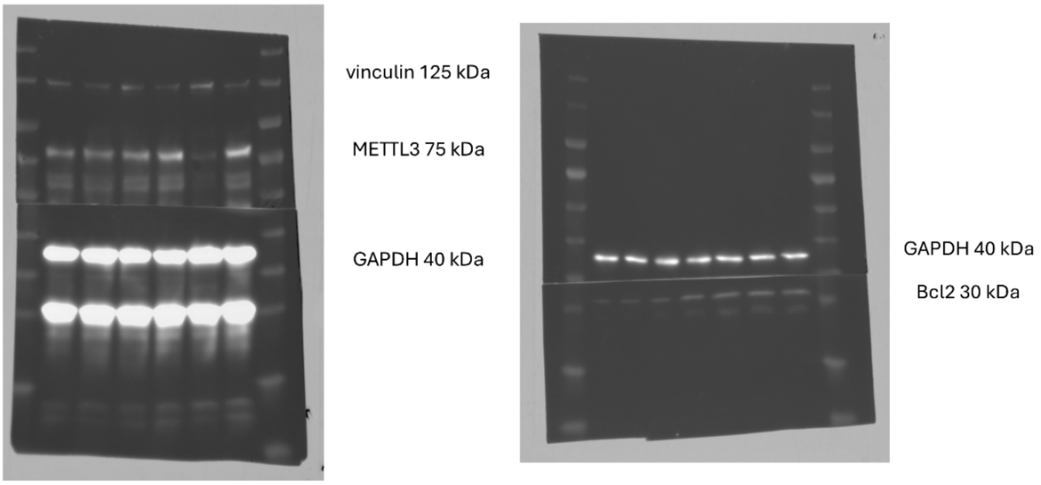

Figure S10: Uncropped images of Figures 2E&4E: Data originates from single blots cut between GAPDH (40 kDa) and METTL3 (75 kDa) for Figure 2E and GAPDH (40 kDa) and Bcl-2 (30 kDa) for Figure 4E.

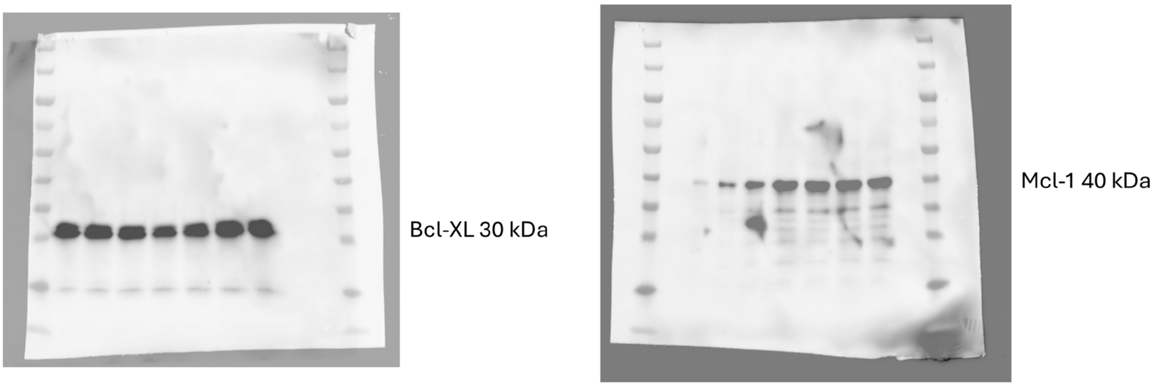

Figure S11: Uncropped images of Figure 4E: Data originates from Bcl-XL (30 kDa) and Mcl-1 (40 kDa) for Figure 4E.

References

1 W. Du, Y. Huang, X. Chen, Y. Deng, Y. Sun, H. Yang, Q. Shi, F. Wu, G. Liu, H. Huang, J. Ding, X. Huang and S. Xu, *Cell chemical biology*, 2024, **31**, 177-183.

2 J. L. Meidner, A. F. Frey, R. A. Zimmermann, M. O. Sabin, Z. Nidoieva, A. C. Weldert, S. N. Hoba, M. W. Krone and F. Barthels, *Angewandte Chemie (International ed. in English)*, 2024, **63**, e202403792.

3 E. Yankova, W. Blackaby, M. Albertella, J. Rak, E. de Braekeleer, G. Tsagkogeorga, E. S. Pilka, D. Aspris, D. Leggate, A. G. Hendrick, N. A. Webster, B. Andrews, R. Fosbeary, P. Guest, N. Irigoyen, M. Eleftheriou, M. Gozdecka, J. M. L. Dias, A. J. Bannister, B. Vick, I. Jeremias, G. S. Vassiliou, O. Rausch, K. Tzelepis and T. Kouzarides, *Nature*, 2021, **593**, 597–601.

4 A. A. Guirguis, Y. Ofir-Rosenfeld, K. Knezevic, W. Blackaby, D. Hardick, Y.-C. Chan, A. Motazedian, A. Gillespie, D. Vassiliadis, E. Y. N. Lam, K. Tran, B. Andrews, M. E. Harbour, L. Vasiliauskaite, C. J. Saunders, G. Tsagkogeorga, A. Azevedo, J. Obacz, E. S. Pilka, M. Carkill, L. MacPherson, E. N. Wainwright, B. Liddicoat, B. J. Blyth, M. R. Albertella, O. Rausch and M. A. Dawson, *Cancer discovery*, 2023, **13**, 2228–2247.

5 L. Liao, Y. He, S.-J. Li, G.-G. Zhang, W. Yu, J. Yang, Z.-J. Huang, C.-C. Zheng, Q.-Y. He, Y. Li and B. Li, *Cancer research*, 2022, **82**, 2444–2457.

6 K. Hwang, J. Bae, Y.-L. Jhe, J. Kim, J.-H. Cheong, H.-S. Choi and T. Sim, *European journal of medicinal chemistry*, 2024, **279**, 116843.

Synthesis of E3 ligase recruiters with linker

VHL-RH-based recruiters

General procedure: A mixture of VL-285 phenol (0.1 mmol, 1.0 eq.), Boc-NH-PEG$_n$-OTs (0.1 mmol, 1.0 eq.), and K_2CO_3 (1.0 mmol, 10.0 eq.) in DMF (3 mL) was stirred at 80 °C for 16 h. The solvent was evaporated, and the mixture was purified by RP flash chromatography to afford the desired intermediate. The residue was then resolved in DCM/TFA (2:1) and stirred at RT for 1 h. Evaporation and lyophilization gave the product.

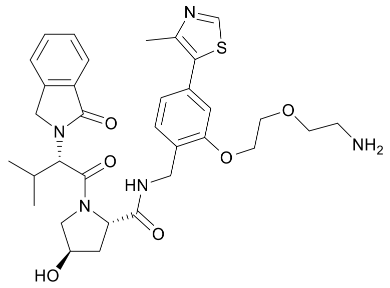

Compound **43**: Yield: 70 mg (94% of th.).

^{1}H NMR: (300 MHz, MeO*D*) δ [ppm] = 9.07 (s, 1H), 7.65 (d, *J* = 7.5 Hz, 1H), 7.51 – 7.31 (m, 5H), 6.98 – 6.92 (m, 2H), 4.72 (d, *J* = 10.9 Hz, 1H), 4.51 – 4.27 (m, 7H), 4.16 – 4.12 (m, 2H), 3.87 – 3.66 (m, 7H), 3.05 (t, *J* = 5.1 Hz, 2H), 2.42 – 2.26 (m, 5H), 2.15 – 2.06 (m, 1H), 1.95 (ddd, *J* = 19.2, 9.7, 5.2 Hz, 1H), 0.91 (d, *J* = 6.4 Hz, 3H), 0.70 (d, *J* = 6.7 Hz, 3H).

^{13}C NMR: (75 MHz, MeOD) δ [ppm] = 174.2, 170.9, 170.7, 157.9, 143.7, 133.2, 132.4, 132.1, 130.0, 129.2, 128.6, 124.4, 123.0, 113.4, 70.8, 70.8, 69.3, 68.1, 60.7, 60.1, 57.1, 40.7, 39.3, 39.1, 30.1, 19.7, 19.0, 15.2.

LC/MS: m/z calculated for $C_{33}H_{41}N_5O_6S$ $[M+H]^+$: 636.3, found: 635.9.

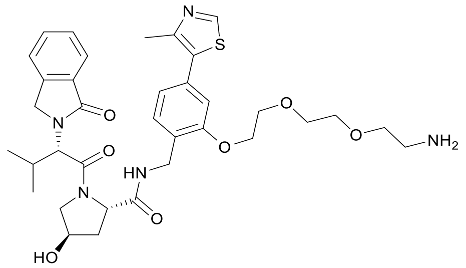

Compound **44**: Yield: 62 mg (78% of th.).

^{1}H NMR: (300 MHz, MeO*D*) δ [ppm] = 9.06 (s, 1H), 7.60 (dt, *J* = 7.6, 1.0 Hz, 1H), 7.46 – 7.36 (m, 2H), 7.35 – 7.25 (m, 2H), 6.90 (dq, *J* = 3.4, 1.6 Hz, 2H), 4.67 (d, *J* = 10.9 Hz, 1H), 4.49 – 4.21 (m, 6H), 4.13 – 3.92 (m, 2H), 3.83 (d, *J* = 11.1 Hz, 1H), 3.79 – 3.67 (m, 3H), 3.63 – 3.57 (m, 2H), 3.56 – 3.48 (m, 4H), 2.93 (t, *J* = 5.1 Hz, 2H), 2.35 (s, 3H), 2.26 (dt, *J* = 10.8, 6.5 Hz, 1H), 2.05 (dd, *J* = 12.9, 8.0 Hz, 1H), 1.88 (ddd, *J* = 13.3, 9.1, 4.4 Hz, 1H), 0.87 (d, *J* = 6.5 Hz, 3H), 0.65 (d, *J* = 6.6 Hz, 3H).

^{13}C NMR: (75 MHz, MeOD) δ [ppm] = 174.2, 171.0, 170.7, 161.1, 160.6, 158.1, 154.1, 147.0, 143.8, 134.8, 133.3, 132.4, 131.8, 130.0, 129.2, 128.9, 124.4, 123.0, 113.6, 71.7, 71.3, 69.3, 67.9, 60.7, 60.1, 57.2, 40.7, 39.4, 39.1, 30.1, 19.7, 19.0, 15.0.

LC/MS: m/z calculated for $C_{35}H_{45}N_5O_7S$ $[M+H]^+$: 680.3, found: 680.1.

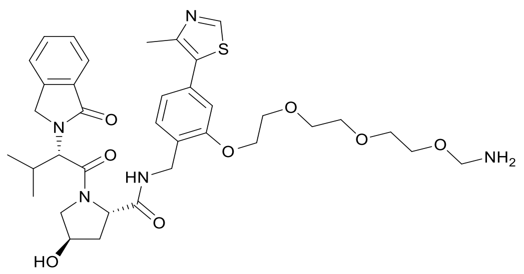

Compound **45**: Yield: 66 mg (79% of th.).

^{1}H NMR: (300 MHz, MeO*D*) δ [ppm] = 9.09 (s, 1H), 7.53 (d, *J* = 7.6 Hz, 1H), 7.40 – 7.29 (m, 2H), 7.24 (dd, *J* = 8.5, 6.3 Hz, 2H), 6.88 – 6.74 (m, 2H), 4.60 (d, *J* = 10.9 Hz, 1H), 4.42 – 4.15 (m, 6H), 4.00 (dd, *J* = 5.8, 3.3 Hz, 2H), 3.79 – 3.60 (m, 4H), 3.51 (dd, *J* = 6.2, 3.1 Hz, 2H), 3.47 – 3.31 (m, 9H), 2.85 (t, *J* = 4.9 Hz, 2H), 2.30 (s, 4H), 1.99 (dd, *J* = 13.4, 7.9 Hz, 1H), 1.82 (ddd, *J* = 13.3, 9.0, 4.5 Hz, 1H), 0.80 (d, *J* = 6.5 Hz, 3H), 0.58 (d, *J* = 6.5 Hz, 3H).

^{13}C NMR: (75 MHz, MeOD) δ [ppm] = 174.3, 171.0, 170.6, 158.1, 154.4, 146.4, 143.8, 135.1, 133.3, 132.4, 131.4, 130.1, 129.2, 129.1, 124.4, 122.9, 118.9, 117.9, 113.6, 71.7, 71.5, 71.2, 70.8, 70.8, 69.4, 67.8, 60.7, 60.1, 57.2, 40.6, 39.4, 39.1, 31.1, 30.1, 19.7, 19.0, 14.7.

LC/MS: m/z calculated for $C_{37}H_{49}N_5O_8S$ $[M+H]^+$: 724.3, found: 724.0.

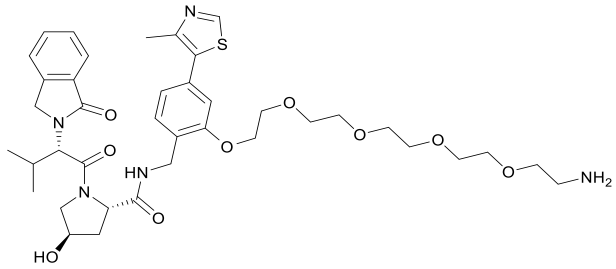

Compound **46**: Yield: 67 mg (76% of th.).

^{1}H NMR: (300 MHz, MeO*D*) δ [ppm] = 9.07 (s, 1H), 7.58 – 7.48 (m, 1H), 7.40 – 7.28 (m, 2H), 7.24 (ddd, *J* = 7.1, 4.7, 2.0 Hz, 2H), 6.87 – 6.75 (m, 2H), 4.60 (d, *J* = 10.9 Hz, 1H), 4.43 – 4.16 (m, 6H), 4.04 – 3.93 (m, 2H), 3.76 (d, *J* = 11.3 Hz, 1H), 3.71 – 3.59 (m, 3H), 3.51 (dt, *J* = 3.8, 2.3 Hz, 2H), 3.46 – 3.31 (m, 13H), 2.85 (t, *J* = 5.1 Hz, 2H), 2.30 (s, 3H), 2.19 (dt, *J* = 10.9, 6.5 Hz, 1H), 2.03 – 1.92 (m, 1H), 1.82 (ddd, *J* = 13.3, 9.0, 4.5 Hz, 1H), 0.80 (d, *J* = 6.5 Hz, 3H), 0.58 (d, *J* = 6.6 Hz, 3H).

^{13}C NMR: (75 MHz, MeOD) δ [ppm] = 174.2, 170.9, 170.6, 161.1, 160.6, 158.1, 154.4, 146.5, 143.7, 135.0, 133.3, 132.4, 131.5, 130.1, 129.2, 129.1, 124.4, 123.0, 118.9, 115.1, 113.7, 71.7, 71.4, 71.3, 71.1, 70.8, 70.8, 69.4, 67.8, 60.7, 60.1, 57.1, 40.6, 39.4, 39.1, 30.1, 19.7, 19.0, 14.8.

LC/MS: m/z calculated for $C_{39}H_{53}N_5O_9S$ $[M+H]^+$: 768.4, found: 768.0.

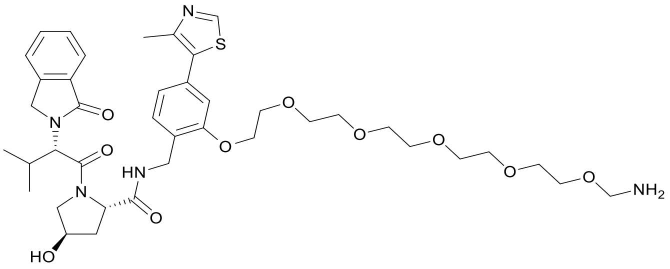

Compound **47**: Yield: 62 mg (67% of th.).

^{1}H NMR: (300 MHz, MeO*D*) δ [ppm] = 9.06 (s, 1H), 7.57 (d, *J* = 7.7 Hz, 1H), 7.44 – 7.33 (m, 2H), 7.28 (dt, *J* = 7.3, 3.7 Hz, 2H), 6.92 – 6.79 (m, 2H), 4.64 (d, *J* = 10.9 Hz, 1H), 4.47 – 4.20 (m, 6H), 4.05 (t, *J* = 4.3 Hz, 2H), 3.87 – 3.64 (m, 4H), 3.59 – 3.52 (m, 2H), 3.50 – 3.35 (m, 17H), 2.90 (t, *J* = 5.1 Hz, 2H), 2.33 (s, 3H), 2.28 – 2.17 (m, 1H), 2.06 – 1.81 (m, 2H), 0.84 (d, *J* = 6.5 Hz, 3H), 0.62 (d, *J* = 6.7 Hz, 3H).

^{13}C NMR: (75 MHz, MeOD) δ [ppm] = 174.2, 170.9, 170.6, 161.1, 160.6, 158.1, 154.2, 146.9, 143.8, 134.8, 133.3, 132.4, 131.7, 130.1, 129.2, 129.0, 124.4, 124.4, 123.0, 113.7, 71.6, 71.4, 71.3, 71.3, 71.2, 71.1, 70.8, 70.7, 69.4, 67.8, 60.7, 60.1, 57.1, 40.5, 39.4, 39.1, 30.1, 19.7, 19.0, 14.9.

LC/MS: m/z calculated for $C_{41}H_{57}N_5O_{10}S$ $[M+H]^+$: 812.4, found: 812.2.

VHL-LH-based recruiters

General procedure: VH032-amine (0.05 mmol, 1.0 eq.), TBTU (0.05 mmol, 1.0 eq.), and Boc-NH-PEG$_n$-COOH (0.05 mmol, 1.0 eq.) were solved in DMF (1 mL), and DIPEA (0.15 mmol, 3.0 eq.) was added. The suspension was stirred at RT for 16 h. The suspension was then concentrated under reduced pressure, purified by RP flash chromatography, and lyophilized. The resulting solid was resolved in dichloromethane (1 ml) and TFA (1 ml) and stirred at RT for 1 h. Evaporation and lyophilization gave the product.

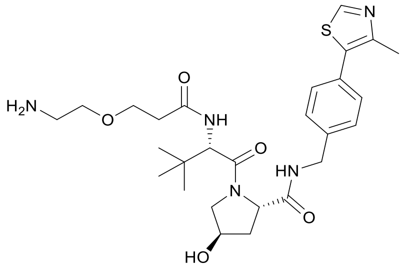

Compound **48**: Yield: 47 mg (quant.).

^{1}H NMR: (300 MHz, MeO*D*) δ [ppm] = 9.08 (s, 1H), 7.56 – 7.41 (m, 1H), 7.34 – 7.22 (m, 5H), 4.44 – 4.29 (m, 4H), 4.21 (d, *J* = 15.6 Hz, 1H), 3.65 – 3.43 (m, 6H), 2.94 (t, *J* = 5.1 Hz, 2H), 2.33 (s, 3H), 2.06 (ddt, *J* = 11.3, 7.6, 1.9 Hz, 1H), 1.91 (ddd, *J* = 13.3, 9.2, 4.3 Hz, 1H), 1.29 – 1.12 (m, 3H), 0.86 (s, 9H), 0.79 – 0.70 (m, 2H).

^{13}C NMR: (75 MHz, MeOD) δ [ppm] = 174.4, 173.9, 172.4, 154.2, 141.0, 132.4, 130.4, 130.3, 129.1, 71.1, 69.1, 67.9, 67.4, 60.9, 59.4, 58.0, 49.9, 49.6, 49.3, 49.0, 48.7, 48.4, 48.2, 43.7, 40.4, 40.2, 39.0, 36.7, 36.5, 31.6, 30.1, 27.0, 24.9, 24.0, 14.8, 14.4, 11.4.

LC/MS: m/z calculated for $C_{27}H_{39}N_5O_5S$ $[M+H]^+$: 546.3, found: 546.4.

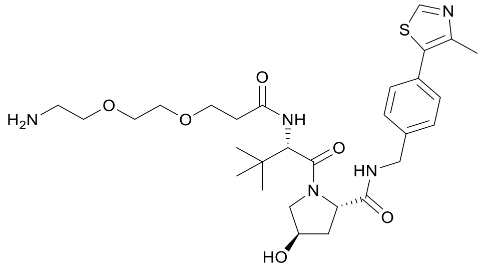

Compound **49**: Yield: 36 mg (quant.).

^{1}H NMR: (300 MHz, MeO*D*) δ [ppm] = 9.12 (s, 1H), 7.55 – 7.40 (m, 1H), 7.34 – 7.23 (m, 4H), 4.48 – 4.28 (m, 4H), 4.21 (d, *J* = 15.5 Hz, 1H), 3.65 – 3.42 (m, 9H), 2.92 (t, *J* = 5.0 Hz, 2H), 2.33 (s, 3H), 2.11 – 1.84 (m, 2H), 1.26 – 1.08 (m, 4H), 0.85 (s, 9H), 0.78 – 0.69 (m, 3H).

^{13}C NMR: (75 MHz, MeOD) δ [ppm] = 173.1, 172.3, 170.9, 152.9, 139.7, 132.2, 131.0, 129.0, 128.8, 127.8, 69.9, 69.9, 69.7, 67.7, 66.7, 66.4, 59.5, 57.7, 56.6, 48.5, 48.2, 47.9, 47.6, 47.3, 47.0, 46.8, 42.3, 39.2, 38.8, 37.7, 35.6, 35.3, 30.2, 28.7, 25.6, 23.5, 22.6, 13.3, 13.0, 10.0.

LC/MS: m/z calculated for $C_{29}H_{43}N_5O_6S$ $[M+H]^+$: 590.3, found: 589.9.

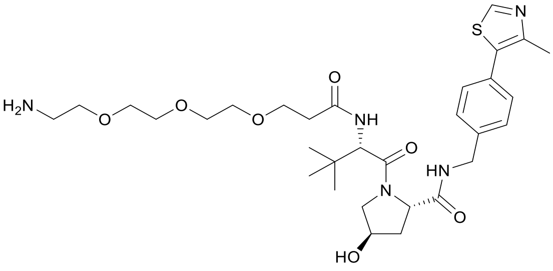

Compound **50**: Yield: 48 mg (quant.).

^{1}H NMR: (300 MHz, MeO*D*) δ [ppm] = 9.03 (s, 1H), 7.67 – 7.51 (m, 1H), 7.38 (q, *J* = 8.5 Hz, 4H), 4.56 (s, 1H), 4.52 – 4.48 (m, 1H), 4.43 (d, *J* = 4.2 Hz, 1H), 4.14 (dd, *J* = 5.7, 1.3 Hz, 1H), 3.83 (d, *J* = 11.1 Hz, 1H), 3.68 – 3.51 (m, 12H), 3.05 (t, *J* = 5.0 Hz, 2H), 2.42 (s, 3H), 2.21 – 2.10 (m, 1H), 2.01 (ddd, *J* = 13.3, 9.2, 4.4 Hz, 1H), 1.31 – 1.22 (m, 4H), 0.97 (s, 9H), 0.90 – 0.81 (m, 4H).

^{13}C NMR: (75 MHz, MeOD) δ [ppm] = 174.4, 173.8, 172.2, 169.3, 153.6, 140.7, 133.6, 132.4, 130.9, 130.4, 129.9, 129.1, 117.9, 114.1, 71.5, 71.4, 71.2, 71.1, 69.1, 68.3, 67.8, 60.9, 59.1, 58.0, 43.7, 40.6, 40.2, 39.0, 37.1, 36.7, 31.6, 30.1, 27.0, 24.9, 24.0, 15.3, 14.4, 11.4.

LC/MS: m/z calculated for $C_{31}H_{47}N_5O_7S$ $[M+H]^+$: 634.3, found: 634.0.

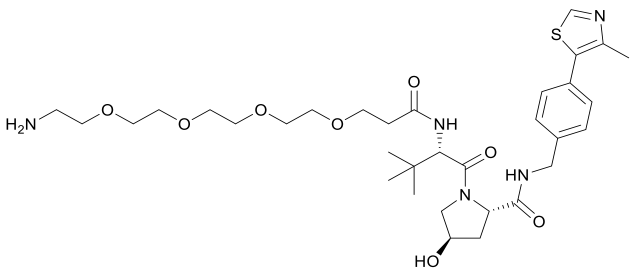

Compound **51**: Yield: 36 mg (91% of th.).

^{1}H NMR: (300 MHz, MeO*D*) δ [ppm] = 9.78 (s, 1H), 8.50 – 8.42 (m, 1H), 8.21 – 8.13 (m, 4H), 4.45 – 4.24 (m, 19H), 3.94 (s, 2H), 3.75 (h, *J* = 4.9, 4.3 Hz, 2H), 3.22 (s, 3H), 2.88 – 2.78 (m, 1H), 2.68 (ddd, *J* = 12.9, 8.6, 4.5 Hz, 1H), 2.17 – 1.97 (m, 3H), 1.71 (s, 9H), 1.63 (td, *J* = 7.2, 4.3 Hz, 3H).

^{13}C NMR: (75 MHz, MeOD) δ [ppm] = 181.7, 179.9, 179.2, 176.7, 161.3, 149.2, 141.4, 141.3, 139.2, 138.3, 137.1, 79.4, 79.3, 79.1, 78.6, 77.1, 76.6, 76.3, 68.4, 66.1, 58.3, 51.3, 47.8, 47.6, 45.3, 45.1, 39.5, 38.0, 32.9, 32.1, 25.5, 23.5, 20.4.

LC/MS: m/z calculated for $C_{33}H_{51}N_5O_8S$ [M+H]$^+$: 678.4, found: 678.2.

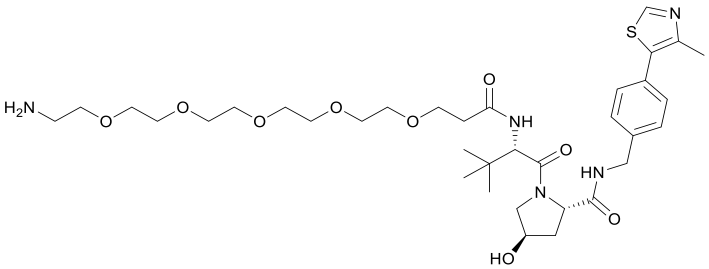

Compound **52**: Yield: 40 mg (96% of th.).

^{1}H NMR (300 MHz, MeO*D*) δ [ppm] = 9.09 (s, 1H), 7.67 – 7.59 (m, 1H), 7.57 – 7.49 (m, 1H), 7.37 (q, *J* = 8.4 Hz, 4H), 4.56 – 4.39 (m, 4H), 4.28 (d, *J* = 15.6 Hz, 1H), 3.71 – 3.47 (m, 22H), 2.41 (s, 3H), 2.15 (dd, *J* = 13.4, 7.6 Hz, 1H), 1.99 (ddd, *J* = 13.3, 9.3, 4.3 Hz, 1H), 1.24 (tt, *J* = 9.0, 3.9 Hz, 3H), 0.95 (s, 9H), 0.82 (dd, *J* = 8.1, 6.6 Hz, 3H).

^{13}C NMR (75 MHz, MeOD) δ [ppm] = 174.4, 173.8, 172.2, 169.3, 140.8, 133.5, 132.4, 130.6, 130.4, 129.8, 129.1, 71.4, 71.4, 71.3, 71.2, 71.2, 71.1, 71.1, 70.9, 69.1, 68.2, 67.9, 60.8, 43.7, 40.1, 39.0, 36.9, 36.6, 31.6, 30.1, 27.0, 24.9, 24.0, 15.1, 14.4, 11.4.

LC/MS: m/z calculated for $C_{35}H_{55}N_5O_9S$ [M+H]$^+$: 722.4, found: 722.2.

4-Thalidomide-based recruiters

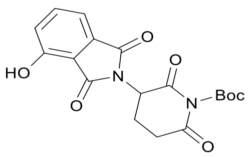

Compound **53**: 4-Hydroxythalidomide (600 mg, 2.19 mmol, 1.0 eq.) was combined with DMAP (268 mg, 2.19 mmol, 1.0 eq.) in 1,4-Dioxane (10 mL), followed by the addition of Boc-anhydride (960 mg, 4.4 mmol, 2.0 eq.). The reaction mixture was stirred at RT for 1 h. Piperidine (0.22 mL, 2.19 mmol, 1.0 eq.) was added, and the mixture was stirred for 1 h. Afterward, the reaction mixture was partitioned between 1 M HCl and EtOAc. The organic phase was filtered over silica, followed by column chromatography (CH/EA 1:1) purification to give the product as a colorless solid.

Yield: 510 mg (62% of th.).

^{1}H NMR: (300 MHz, MeO*D*) δ [ppm] = 7.90 (s, 1H), 7.65 (td, *J* = 7.8, 7.2, 1.3 Hz, 1H), 7.37 (d, *J* = 7.1 Hz, 1H), 7.20 (d, *J* = 8.4 Hz, 1H), 5.22 (dd, *J* = 12.7, 5.4 Hz, 1H), 3.07 – 2.68 (m, 3H), 2.20 – 2.08 (m, 1H), 1.55 (s, 9H).

^{13}C NMR: (75 MHz, MeOD) δ [ppm] = 171.3, 168.8, 168.6, 156.9, 137.6, 134.5, 124.6, 116.0, 87.5, 79.5, 50.3, 32.2, 27.7, 22.9.

LC/MS: m/z calculated for $C_{18}H_{18}N_2O_7$ [M - Boc+H]$^+$: 275.1, found: 274.9.

General Procedure: A mixture of **53** (0.1 mmol, 1.0 eq.), Boc-NH-PEG$_n$-OTs (0.1 mmol, 1.0 eq.), and K_2CO_3 (0.3 mmol, 3.0 eq.) in DMF (1 mL) was agitated at 80 °C for 3 h. The solvent was evaporated, and the mixture was purified by RP flash chromatography to afford the desired intermediate. The intermediate was then resolved in DCM/TFA (2:1) and stirred at RT for 1 h. Evaporation and lyophilization gave the product.

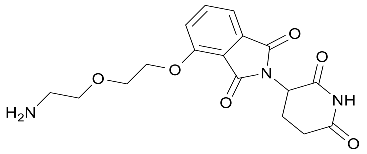

Compound **54**: Yield: 3 mg (6% of th.).

^{1}H NMR: (300 MHz, MeO*D*) δ [ppm] = 7.73 (dd, *J* = 8.5, 7.4 Hz, 1H), 7.44 – 7.37 (m, 2H), 5.05 (dd, *J* = 12.4, 5.4 Hz, 1H), 4.36 – 4.30 (m, 2H), 3.94 – 3.86 (m, 2H), 3.84 – 3.78 (m, 2H), 3.12 (t, *J* = 5.0 Hz, 2H), 2.89 – 2.57 (m, 3H), 2.11 – 2.02 (m, 1H), 1.32 – 0.73 (m, 2H).

^{13}C NMR: (75 MHz, MeOD) δ [ppm] = 174.6, 171.5, 168.5, 167.9, 157.5, 138.2, 135.0, 120.6, 118.2, 116.9, 70.2, 68.2, 50.5, 40.6, 32.2, 23.6.

LC/MS: m/z calculated for $C_{17}H_{19}N_3O_6$ [M+H]$^+$: 362.1, found: 362.0.

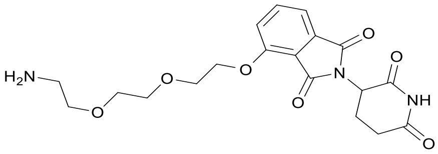

Compound **55**: Yield: 17 mg (33% of th.).

^{1}H NMR: (300 MHz, MeO*D*) δ [ppm] = 7.81 – 7.71 (m, 1H), 7.49 – 7.40 (m, 2H), 5.08 (dd, *J* = 12.4, 5.4 Hz, 1H), 4.43 – 4.32 (m, 2H), 3.95 – 3.87 (m, 2H), 3.77 (dt, *J* = 4.6, 3.1 Hz, 2H), 3.73 – 3.63 (m, 4H), 3.08 (t, *J* = 5.0 Hz, 2H), 2.92 – 2.59 (m, 3H), 2.15 – 2.04 (m, 1H), 1.44 – 0.77 (m, 1H).

^{13}C NMR: (75 MHz, MeOD) δ [ppm] = 174.5, 171.6, 168.5, 167.6, 157.6, 138.1, 135.1, 120.7, 118.3, 116.8, 72.0, 71.2, 70.3, 70.2, 67.9, 50.4, 40.7, 32.2, 23.7.

LC/MS: m/z calculated for $C_{19}H_{23}N_3O_7$ $[M+H]^+$: 406.2, found: 406.0.

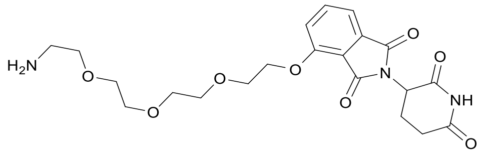

Compound **56**: Yield: 31 mg (55% of th.).

^{1}H NMR: (300 MHz, MeO*D*) δ [ppm] = 7.79 – 7.69 (m, 1H), 7.47 – 7.38 (m, 2H), 5.13 – 5.00 (m, 1H), 4.43 – 4.29 (m, 2H), 3.89 (p, *J* = 2.7 Hz, 2H), 3.76 – 3.70 (m, 2H), 3.69 – 3.57 (m, 8H), 3.07 (t, *J* = 5.1 Hz, 2H), 2.92 – 2.56 (m, 3H), 2.09 (dtd, *J* = 13.3, 5.4, 2.5 Hz, 1H), 1.37 – 0.79 (m, 1H).

^{13}C NMR: (75 MHz, MeOD) δ [ppm] = 174.6, 171.6, 168.4, 168.1, 157.6, 138.2, 135.0, 120.7, 118.2, 116.9, 71.6, 71.4, 71.3, 70.7, 70.3, 67.7, 50.5, 40.5, 32.1, 23.7.

LC/MS: m/z calculated for $C_{21}H_{27}N_3O_8$ $[M+H]^+$: 450.2, found: 450.1.

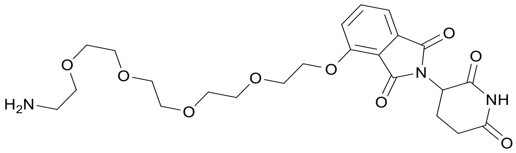

Compound **57**: Yield: 29 mg (48% of th.).

^{1}H NMR: (300 MHz, MeO*D*) δ [ppm] = 7.72 (dd, *J* = 8.6, 7.2 Hz, 1H), 7.45 – 7.38 (m, 2H), 5.04 (dd, *J* = 12.4, 5.5 Hz, 1H), 4.34 (t, *J* = 4.3 Hz, 2H), 4.19 – 4.09 (m, 2H), 3.91 – 3.83 (m, 2H), 3.74 – 3.69 (m, 2H), 3.67 – 3.53 (m, 12H), 3.07 (t, *J* = 5.0 Hz, 2H), 2.87 – 2.54 (m, 3H), 2.14 – 2.00 (m, 1H), 1.60 (q, *J* = 6.0 Hz, 1H).

^{13}C NMR: (75 MHz, MeOD) δ [ppm] = 173.2, 170.1, 167.9, 167.0, 156.2, 136.8, 133.7, 132.2, 131.0, 128.5, 119.5, 116.9, 115.6, 70.4, 70.0, 69.8, 69.5, 69.0, 67.7, 66.5, 49.1, 39.2, 38.8.

LC/MS: m/z calculated for $C_{23}H_{31}N_3O_9$ $[M+H]^+$: 494.2, found: 494.1.

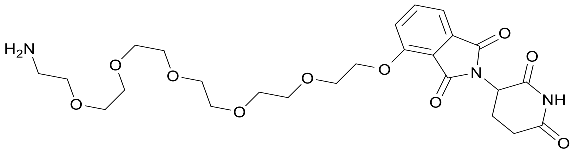

Compound **58**: Yield: 31 mg (48% of th.).

^{1}H NMR: (300 MHz, MeO*D*) δ [ppm] = 7.73 (dd, *J* = 8.6, 7.2 Hz, 1H), 7.41 (d, *J* = 7.7 Hz, 2H), 5.04 (dd, *J* = 12.3, 5.5 Hz, 1H), 4.34 (t, *J* = 4.3 Hz, 2H), 3.91 – 3.85 (m, 2H), 3.76 – 3.70 (m, 2H), 3.66 – 3.51 (m, 16H), 3.10 (t, *J* = 5.2 Hz, 2H), 2.89 – 2.58 (m, 3H), 2.11 – 2.01 (m, 1H).

^{13}C NMR: (75 MHz, MeOD) δ [ppm] = 174.6, 171.4, 168.4, 167.8, 157.5, 138.2, 135.1, 120.8, 118.3, 117.0, 71.5, 71.3, 71.3, 71.2, 71.2, 71.1, 71.0, 70.6, 70.3, 70.1, 67.8, 50.5, 40.7, 32.2, 23.6.

LC/MS: m/z calculated for $C_{25}H_{35}N_3O_{10}$ [M+H]$^+$: 538.2, found: 538.0.

5-Thalidomid-based recruiters

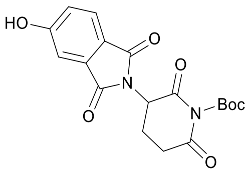

Compound **59**: 5-Hydroxythalidomide (600 mg, 2.19 mmol, 1.0 eq.) was combined with DMAP (268 mg, 2.19 mmol, 1.0 eq.) in 1,4-Dioxane (10 mL), followed by the addition of Boc-anhydride (960 mg, 4.4 mmol, 2.0 eq.). The reaction mixture was then stirred at RT for 1 h. Piperidine (0.22 mL, 2.19 mmol) was added, and the mixture was stirred for 1 h. Afterward, the mixture was partitioned between 1 M HCl and EtOAc, and the organic phase was filtered over silica. Subsequently, purification by column chromatography (CH/EA 1:1) afforded the product as a colorless solid.

Yield: 531 mg (65% of th.).

^{1}H NMR (300 MHz, MeO*D*) δ [ppm] = 7.73 (d, *J* = 8.2 Hz, 1H), 7.22 (d, *J* = 2.2 Hz, 1H), 7.15 (dd, *J* = 8.2, 2.2 Hz, 1H), 5.24 (dd, *J* = 12.7, 5.3 Hz, 1H), 3.08 – 2.70 (m, 3H), 2.16 (dtd, *J* = 13.1, 5.1, 2.6 Hz, 1H), 2.03 (d, *J* = 4.8 Hz, 1H), 1.54 (s, 9H).

^{13}C NMR: (75 MHz, MeOD) δ [ppm] = 171.3, 168.7, 168.5, 165.1, 149.6, 135.6, 126.6, 123.2, 121.8, 111.2, 87.4, 50.4, 32.2, 28.7, 27.6, 22.8.

LC/MS: m/z calculated for $C_{18}H_{18}N_2O_7$ [M - Boc+H]$^+$: 275.1, found: 274.9.

General Procedure: A mixture of **59** (0.1 mmol, 1.0 eq.), Boc-NH-PEG$_n$-OTs (0.1 mmol, 1.0 eq.), and K_2CO_3 (0.3 mmol, 3.0 eq.) in DMF (1 mL) was agitated at 80 °C for 3 h. The solvent was evaporated, and the mixture was purified by RP flash chromatography to afford the desired intermediate. The intermediate residue was then resolved in DCM/TFA (2:1) and stirred at RT for 1 h. Evaporation and lyophilization gave the product.

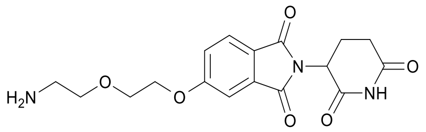

Compound **60**: Yield: 21 mg (44% of th.).

^{1}H NMR: (300 MHz, MeO*D*) δ [ppm] = 7.79 (d, *J* = 8.3 Hz, 1H), 7.40 (d, *J* = 2.3 Hz, 1H), 7.31 (dd, *J* = 8.3, 2.3 Hz, 1H), 5.09 (dd, *J* = 12.4, 5.4 Hz, 1H), 4.35 – 4.29 (m, 2H), 3.95 – 3.88 (m, 2H), 3.77 (dd, *J* = 5.6, 4.5 Hz, 2H), 3.14 (t, *J* = 5.0 Hz, 2H), 2.91 – 2.62 (m, 3H), 2.16 – 2.05 (m, 1H).

^{13}C NMR: (75 MHz, MeOD) δ [ppm] = 174.6, 171.5, 168.6, 168.5, 165.6, 135.7, 126.4, 125.1, 121.5, 110.2, 70.5, 69.6, 68.1, 50.6, 40.6, 32.2, 23.7.

LC/MS: m/z calculated for $C_{17}H_{19}N_3O_6$ [M+H]$^+$: 362.1, found: 362.0.

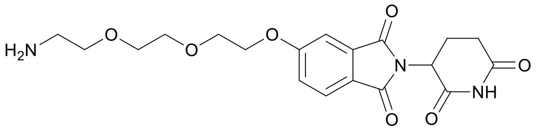

Compound **61**: Yield: 43 mg (83% of th.).

^{1}H NMR: (300 MHz, MeO*D*) δ [ppm] = 7.69 (d, *J* = 8.3 Hz, 1H), 7.32 (d, *J* = 2.3 Hz, 1H), 7.22 (dd, *J* = 8.3, 2.3 Hz, 1H), 4.99 (dd, *J* = 12.4, 5.4 Hz, 1H), 4.23 – 4.17 (m, 2H), 3.84 – 3.76 (m, 2H), 3.67 – 3.56 (m, 6H), 3.00 (t, *J* = 5.0 Hz, 2H), 2.84 – 2.52 (m, 3H), 2.04 (s, 1H), 1.92 (s, 1H), 1.18 (s, 1H).

^{13}C NMR: (75 MHz, MeOD) δ [ppm] = 174.6, 171.5, 168.6, 165.7, 135.7, 126.4, 125.0, 121.6, 110.2, 71.3, 70.6, 69.6, 68.2, 67.9, 50.6, 40.6, 32.2, 24.2, 23.7.

LC/MS: m/z calculated for $C_{19}H_{23}N_3O_7$ [M+H]$^+$: 406.2, found: 406.0.

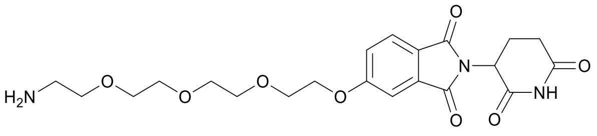

Compound **62**: Yield: 40 mg (71% of th.).

^{1}H NMR: (300 MHz, MeO*D*) δ [ppm] = 7.70 (d, *J* = 8.3 Hz, 1H), 7.33 (d, *J* = 2.2 Hz, 1H), 7.24 (dd, *J* = 8.3, 2.3 Hz, 1H), 5.01 (dd, *J* = 12.4, 5.4 Hz, 1H), 4.25 – 4.19 (m, 2H), 3.84 – 3.76 (m, 2H), 3.66 – 3.53 (m, 10H), 3.02 (t, *J* = 5.0 Hz, 2H), 2.86 – 2.54 (m, 3H), 2.09 – 1.99 (m, 1H).

^{13}C NMR: (75 MHz, MeOD) δ [ppm] = 174.6, 171.5, 168.6, 168.5, 165.8, 135.6, 126.4, 124.9, 121.6, 110.3, 71.6, 71.5, 71.4, 71.2, 70.5, 69.7, 67.8, 50.6, 40.6, 32.2, 23.7.

LC/MS: m/z calculated for $C_{21}H_{27}N_3O_8$ [M+H]$^+$: 450.2, found: 450.0.

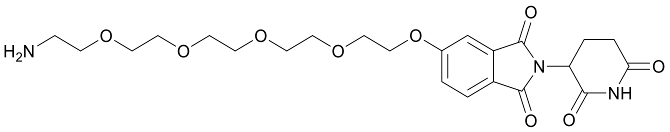

Compound **63**: Yield: 45 mg (74% of th.).

^{1}H NMR: (300 MHz, MeO*D*) δ [ppm] = 7.73 (d, *J* = 8.3 Hz, 1H), 7.35 (d, *J* = 2.2 Hz, 1H), 7.26 (dd, *J* = 8.3, 2.2 Hz, 1H), 5.03 (dd, *J* = 12.4, 5.4 Hz, 1H), 4.27 – 4.21 (m, 2H), 3.86 – 3.79 (m, 2H), 3.68 – 3.55 (m, 14H), 3.04 (t, *J* = 5.0 Hz, 2H), 2.89 – 2.55 (m, 3H), 2.06 (dtd, *J* = 12.7, 4.9, 2.1 Hz, 1H).

^{13}C NMR: (75 MHz, MeOD) δ [ppm] = 174.6, 171.5, 168.6, 168.5, 165.8, 135.7, 126.4, 125.0, 121.6, 110.3, 71.7, 71.4, 71.4, 71.3, 71.1, 70.5, 69.7, 67.8, 50.6, 40.6, 32.2, 23.7.

LC/MS: m/z calculated for $C_{23}H_{31}N_3O_9$ [M+H]$^+$: 494.2, found: 493.9.

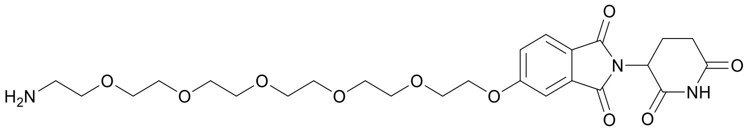

Compound **64**: Yield: 53 mg (81% of th.).

^{1}H NMR: (300 MHz, MeO*D*) δ [ppm] = 7.74 (d, *J* = 8.3 Hz, 1H), 7.38 (d, *J* = 2.2 Hz, 1H), 7.28 (dd, *J* = 8.3, 2.3 Hz, 1H), 5.04 (dd, *J* = 12.4, 5.4 Hz, 1H), 4.29 – 4.22 (m, 2H), 3.88 – 3.81 (m, 2H), 3.70 – 3.50 (m, 19H), 3.06 (t, *J* = 5.0 Hz, 2H), 2.89 – 2.57 (m, 3H), 2.11 – 2.02 (m, 1H).

^{13}C NMR: (75 MHz, MeOD) δ [ppm] = 174.6, 171.5, 168.6, 168.5, 165.7, 135.7, 126.4, 125.0, 121.5, 110.4, 71.6, 71.4, 71.3, 71.2, 70.8, 70.4, 69.6, 67.8, 50.6, 40.5, 32.2, 23.7.

LC/MS: m/z calculated for $C_{25}H_{35}N_3O_{10}$ [M+H]$^+$: 538.2, found: 538.0.

VHL-LH building blocks for Suzuki and Sonogashira reactions

General Procedure: VH032-amine (0.046–0.058 mmol, 1.0 eq.), TBTU (0.046–0.058 mmol, 1.0 eq.), and the respective carboxylic acid (0.046–0.058 mmol, 1.0 eq.) were solved in DMF (1 mL), and then DIPEA (0.14–0.17 mmol, 3.0 eq.) was added. The resulting suspension was stirred at RT for 16 h. The suspension was then concentrated under reduced pressure, purified by RP flash chromatography, and subsequently lyophilized.

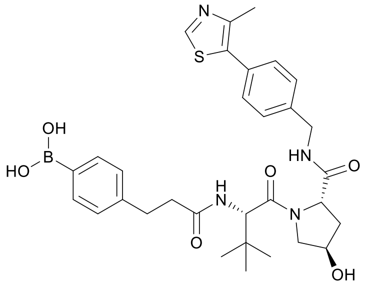

Compound **65**: Yield: 25 mg (71% of th.).

^{1}H NMR: (300 MHz, MeO*D*) δ [ppm] = 9.04 (s, 1H), 7.64 (d, *J* = 7.8 Hz, 1H), 7.44 (q, *J* = 8.3 Hz, 7H), 7.20 (d, *J* = 7.6 Hz, 2H), 4.63 – 4.44 (m, 5H), 4.34 (d, *J* = 15.6 Hz, 1H), 3.88 (d, *J* = 11.0 Hz, 1H), 3.76 (dd, *J* = 11.0, 3.9 Hz, 1H), 2.90 (t, *J* = 7.6 Hz, 2H), 2.59 (dp, *J* = 21.6, 7.6, 7.1 Hz, 2H), 2.48 (s, 4H), 2.28 – 1.95 (m, 3H), 0.94 (s, 9H).

^{13}C NMR: (75 MHz, MeOD) δ [ppm] = 178.6, 174.9, 174.5, 172.3, 153.5, 140.7, 130.9, 130.4, 129.1, 128.7, 71.1, 60.8, 59.1, 58.0, 48.6, 43.7, 38.9, 38.1, 36.5, 32.8, 32.8, 27.0, 15.4, 8.8.

LC/MS: m/z calculated for $C_{31}H_{39}BN_4O_6S$ [M+H]$^+$: 607.3, found: 607.0.

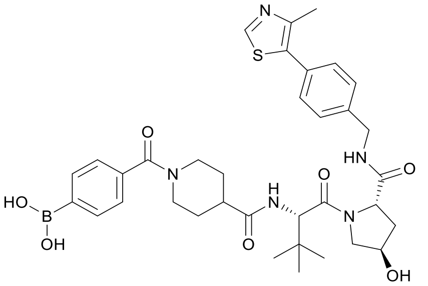

Compound **66**: Yield: 17 mg (43% of th.).

^{1}H NMR: (400 MHz, MeO*D*) δ [ppm] = 8.87 (d, *J* = 2.2 Hz, 1H), 7.82 (d, *J* = 7.7 Hz, 2H), 7.48 – 7.31 (m, 6H), 4.52 (s, 2H), 4.37 (s, 1H), 3.88 (d, *J* = 11.1 Hz, 1H), 3.84 – 3.67 (m, 2H), 2.91 (d, *J* = 12.5 Hz, 1H), 2.72 – 2.64 (m, 1H), 2.47 (d, *J* = 2.3 Hz, 3H), 2.22 (t, *J* = 10.4 Hz, 1H), 2.13 – 2.02 (m, 2H), 1.88 (d, *J* = 21.7 Hz, 1H), 1.70 (d, *J* = 21.3 Hz, 3H), 1.35 – 1.28 (m, 1H), 1.03 (s, 9H).

^{13}C NMR: (101 MHz, MeOD) δ [ppm] = 174.5, 172.7, 172.2, 152.9, 149.0, 140.3, 135.2, 133.4, 131.5, 130.4, 129.0, 126.7, 71.1, 60.8, 58.9, 58.0, 49.6, 49.3, 49.1, 48.9, 48.7, 43.7, 43.4, 38.9, 36.7, 27.0, 15.8.

LC/MS: m/z calculated for $C_{35}H_{44}BN_5O_7S$ [M+H]$^+$: 690.3, found: 690.3.

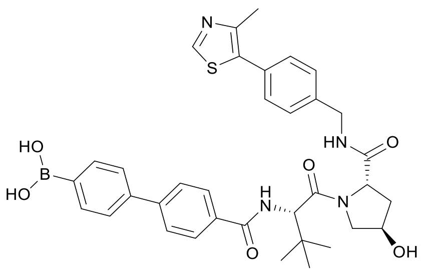

Compound **67**: Yield: 32 mg (quant.).

^{1}H NMR: (300 MHz, MeO*D*) δ [ppm] = 9.04 (s, 1H), 7.98 – 7.82 (m, 3H), 7.81 – 7.62 (m, 6H), 7.54 – 7.40 (m, 5H), 4.62 (dd, *J* = 16.4, 9.1 Hz, 3H), 4.37 (d, *J* = 15.6 Hz, 1H), 4.02 (d, *J* = 11.1 Hz, 1H), 3.90 (dd, *J* = 11.0, 3.8 Hz, 1H), 2.51 (s, 3H), 2.32 – 2.07 (m, 2H), 1.37 – 1.28 (m, 1H), 1.14 (s, 10H), 1.07 – 0.89 (m, 1H).

^{13}C NMR: (75 MHz, MeOD) δ [ppm] = 174.4, 172.4, 169.6, 153.2, 140.5, 131.2, 130.4, 129.1, 129.0, 128.1, 127.3, 71.1, 60.9, 59.4, 58.2, 57.5, 49.4, 49.1, 48.9, 43.7, 39.0, 37.3, 27.1, 15.6.

LC/MS: m/z calculated for $C_{35}H_{39}BN_4O_6S$ [M+H]$^+$: 655.3, found: 654.4.

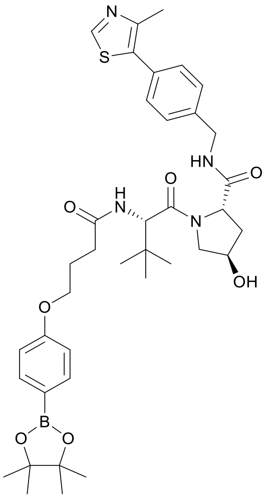

Compound **68**: Yield: 27 mg (65% of th.).

^{1}H NMR: (300 MHz, C*D*Cl$_3$) δ [ppm] = 7.67 – 7.60 (m, 3H), 7.24 – 7.16 (m, 2H), 6.82 – 6.73 (m, 3H), 6.25 (d, *J* = 8.7 Hz, 1H), 4.58 (t, *J* = 7.9 Hz, 1H), 4.51 – 4.41 (m, 3H), 4.23 (dd, *J* = 15.0, 5.2 Hz, 1H), 3.98 (d, *J* = 11.4 Hz, 1H), 3.89 (t, *J* = 6.0 Hz, 2H), 3.52 (dd, *J* = 11.4, 3.6 Hz, 1H), 2.46 – 2.39 (m, 4H), 2.32 (td, *J* = 7.5, 7.0, 4.9 Hz, 2H), 1.98 (p, *J* = 6.8 Hz, 3H), 1.23 (d, *J* = 1.6 Hz, 15H), 0.82 (s, 9H).

^{13}C NMR: (75 MHz, C*D*Cl$_3$) δ [ppm] = 173.3, 171.9, 170.9, 161.5, 138.5, 136.7, 129.7, 128.3, 114.0, 83.7, 77.4, 70.2, 66.6, 58.7, 57.8, 56.9, 43.4, 36.1, 35.0, 32.8, 26.5, 25.2, 25.0, 15.9.

LC/MS: m/z calculated for $C_{38}H_{51}BN_4O_7S$ [M+H]$^+$: 719.4, found: 719.2.

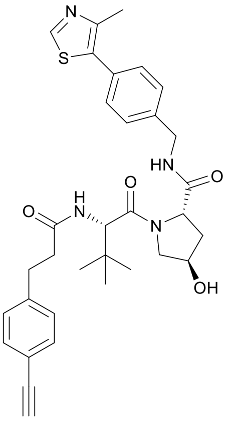

Compound **69**: Yield: 30 mg (88% of th.).

^{1}H NMR: (400 MHz, C*D*Cl$_3$) δ [ppm] = 9.37 (s, 1H), 7.38 (ddd, *J* = 16.8, 9.6, 5.7 Hz, 7H), 7.11 (d, *J* = 7.8 Hz, 2H), 6.35 (d, *J* = 8.7 Hz, 1H), 4.69 – 4.56 (m, 6H), 4.35 (dd, *J* = 15.4, 4.7 Hz, 1H), 4.10 (d, *J* = 11.2 Hz, 1H), 3.65 (dd, *J* = 11.4, 3.4 Hz, 1H), 3.03 (s, 1H), 2.92 (t, *J* = 7.2 Hz, 2H), 2.63 (s, 3H), 2.56 – 2.52 (m, 1H), 2.49 – 2.42 (m, 1H), 2.22 (dd, *J* = 13.6, 7.8 Hz, 1H), 0.88 (s, 9H).

^{13}C NMR: (101 MHz, *C*DCl$_3$) δ [ppm] = 173.2, 171.9, 171.1, 160.1, 159.7, 152.5, 143.8, 141.2, 140.2, 134.9, 132.6, 129.6, 128.8, 128.7, 128.5, 127.9, 127.0, 120.2, 116.6, 115.8, 113.8, 83.8, 70.3, 59.1, 57.9, 57.1, 43.3, 37.8, 36.4, 35.0, 31.6, 26.4, 14.0.

LC/MS: m/z calculated for $C_{33}H_{38}N_4O_4S$ $[M+H]^+$: 587.3, found: 586.3.

1st and 2nd generation METTL3 recruiter synthesis

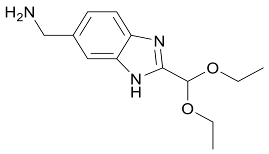

Compound **70**: To a solution of sodium ethoxide (437 mg, equals 19 mmol Na) in 10 mL of dry ethanol (1.9 eq.) *o*-phenylenediamine-5-carbonitrile (1.3 g, 10 mmol, 1.0 eq.) and ethyl diethoxyacetate (2.1 mL, 12 mmol, 1.2 eq.) were added. The mixture was heated in a sealed tube to 120 °C for 16 h, cooled to RT, and the solvent was removed in vacuo. The resulting residue was dissolved in water, neutralized with acetic acid, and extracted with ethyl acetate. Then, the combined organic phases were purified over a short silica column (CH/EA 1:1 +1% TEA) and concentrated under reduced pressure. This intermediate was converted without further characterization. To a solution of the carbonitrile intermediate (1.6 g) in methanol (10 mL), Raney Ni (200 mg, 3.4 mmol, 0.3 eq.) was added, followed by ammonia in methanol (7 M, 3 mL). The reaction solution was hydrogenated at 4 bar at RT for 4 h. The resulting mixture was filtered through a plug of silica. The raw product was purified by column chromatography (EA/MeOH 1:1 + 1% TEA).

Yield: 1.16 g (47% of th.).

^{1}H NMR: (300 MHz, C*D*Cl$_3$) δ [ppm] = 7.52 – 7.43 (m, 2H), 7.09 (dd, *J* = 8.3, 1.6 Hz, 1H), 5.68 (s, 1H), 3.87 (s, 2H), 3.72 – 3.53 (m, 4H), 3.39 (s, 3H), 1.16 (t, *J* = 7.1 Hz, 6H).

^{13}C NMR: (75 MHz, *C*DCl$_3$) δ = 151.8, 137.7, 137.3, 122.3, 97.2, 62.3, 49.8, 46.4, 15.0.

LC/MS: m/z calculated for $C_{13}H_{19}N_3O_2$ $[M+H]^+$: 250.2, found: 250.0.

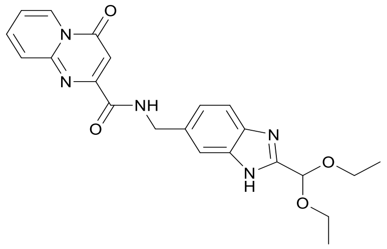

Compound **71**: A solution of 4-oxopyrido[1,2-a]pyrimidine-2-carboxylic acid hydrochloride (452 mg, 2 mmol, 1.0 eq.), TBTU (642 mg, 2 mmol, 1.0 eq.) and **70** (500 mg, 2 mmol, 1.0 eq.) in dry DMF (10 mL) was treated with DIPEA (1 mL, 6 mmol, 3.0 eq.). The suspension was stirred at RT for 16 h and concentrated in vacuo. The resulting residue was diluted with water and dichloromethane, and the organic layer was extracted with saturated $NaHCO_3$ solution and purified via column chromatography (DCM/MeOH 10:1 + 1% TEA). Evaporation gave the product as an off-white solid.

Yield: 472 mg (56% of th.).

^{1}H NMR: (300 MHz, C*D*Cl$_3$) δ [ppm] = 9.09 – 9.01 (m, 1H), 8.37 (s, 1H), 7.81 – 7.70 (m, 2H), 7.64 – 7.57 (m, 1H), 7.45 (d, *J* = 21.8 Hz, 1H), 7.31 (s, 1H), 7.21 – 7.14 (m, 1H), 5.74 (s, 1H), 4.75 (d, *J* = 6.0 Hz, 2H), 3.78 – 3.61 (m, 4H), 1.24 (t, *J* = 7.1 Hz, 6H).

^{13}C NMR: (75 MHz, *C*DCl$_3$) δ [ppm] = 163.0, 158.7, 153.6, 152.0, 150.6, 137.0, 127.7, 126.6, 116.3, 102.9, 97.0, 62.4, 46.1, 44.2, 15.2, 10.4.

LC/MS: m/z calculated for $C_{22}H_{23}N_5O_4$ [M+H]$^+$: 422.2, found: 422.1.

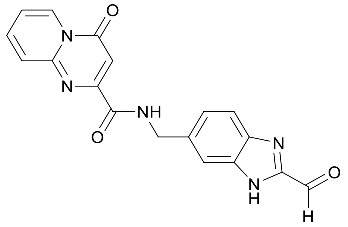

Compound **72**: **71** (230 mg, 0.55 mmol, 1.0 eq.) was solved in THF/water/conc. HCl (3+3+2 mL) and stirred at 80 °C for 3 h under an argon atmosphere. Evaporation and lyophilization afforded the dihydrochloride, which was suspended in DCM/MeOH/TEA (10:1:1), filtered over silica, and evaporated. The residue was washed with distilled water and lyophilized to yield the product as an off-white solid

Yield: 192 mg (quant.).

^{1}H NMR: (300 MHz, DMSO-d_6) δ [ppm] = 9.65 – 9.52 (m, 1H), 9.05 – 8.99 (m, 1H), 8.09 (ddd, *J* = 8.6, 6.7, 1.6 Hz, 1H), 7.79 (dt, *J* = 8.9, 1.2 Hz, 1H), 7.75 – 7.69 (m, 2H), 7.57 (dd, *J* = 8.7, 1.4 Hz, 1H), 7.47 (td, *J* = 6.9, 1.3 Hz, 1H), 6.90 (d, *J* = 4.2 Hz, 1H), 6.25 (s, 1H), 4.66 (s, 2H).

^{13}C NMR: (75 MHz, DMSO) δ [ppm] = 163.1, 157.9, 154.5, 153.5, 150.5, 138.8, 137.7, 130.9, 129.9, 127.5, 126.1, 126.0, 117.5, 114.1, 113.0, 101.0, 83.6, 8.4.

LC/MS: m/z calculated for $C_{18}H_{13}N_5O_3$ [M+H]$^+$: 348.1, found: 348.1.

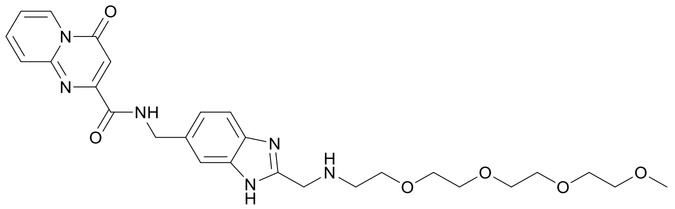

Compound **5**: OMe-PEG4-NH_2 (10.4 mg, 0.05 mmol, 1.0 eq.) and **72** (17.3 mg, 0.05 mmol, 1.0 eq.) were dissolved in dichloroethane (DCE, 1 mL), and AcOH (6 µL, 0.1 mmol, 2.0 eq.) was added to the reaction mixture. $NaBH(OAc)_3$ (21 mg, 0.1 mmol, 2.0 eq.) was added, and the mixture was incubated at 60 °C for 2 h. The reaction was quenched by the addition of MeOH (1 mL). Evaporation, purification by RP flash chromatography, and lyophilization afforded the product as a yellowish oil.

Yield: 26 mg (97% of th.).

LC/MS: m/z calculated for $C_{27}H_{34}N_6O_6$ $[M+H]^+$: 539.3, found: 538.9.

Purity (HPLC): 99%.

Synthesis of 1st generation PROTACs

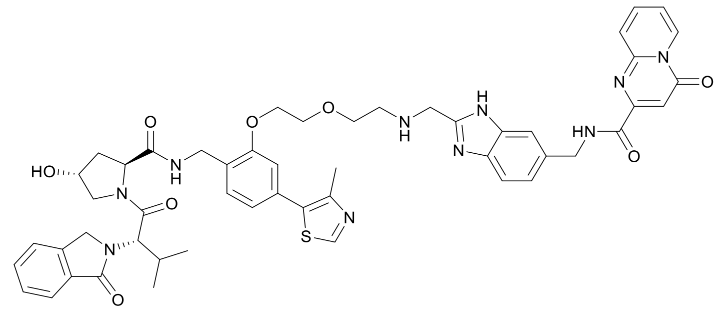

Compound **11**: **43** (12.7 mg, 0.02 mmol, 1.0 eq.) and **72** (9 mg, 0.025 mmol, 1.3 eq.) were dissolved in dichloroethane (DCE, 1 mL), and AcOH (6 µL, 0.1 mmol, 2.0 eq.) was added to the reaction mixture. Then NaBH(OAc)$_3$ (21 mg, 0.1 mmol, 2.0 eq.) was added, and the mixture was incubated at 60 °C for 2 h. The reaction was quenched by the addition of MeOH (1 mL). Evaporation, purification by RP flash chromatography, and lyophilization afforded the product as a yellowish solid.

Yield: 10 mg (51% of th.).

^{1}H NMR: (600 MHz, MeO*D*) δ [ppm] = 9.00 (d, *J* = 7.2 Hz, 1H), 8.87 – 8.79 (m, 1H), 7.92 (ddd, *J* = 8.6, 6.8, 1.4 Hz, 1H), 7.74 (t, *J* = 9.0 Hz, 1H), 7.66 (dd, *J* = 24.8, 7.5 Hz, 1H), 7.57 (s, 1H), 7.52 (h, *J* = 7.7 Hz, 2H), 7.48 – 7.44 (m, 1H), 7.44 – 7.39 (m, 1H), 7.39 – 7.30 (m, 2H), 7.30 – 7.25 (m, 1H), 7.02 – 6.99 (m, 1H), 6.99 – 6.87 (m, 2H), 4.74 (d, *J* = 11.0 Hz, 1H), 4.66 (s, 2H), 4.57 – 4.41 (m, 5H), 4.41 – 4.25 (m, 3H), 4.23 – 4.10 (m, 2H), 3.94 – 3.82 (m, 4H), 3.79 (dd, *J* = 11.1, 3.9 Hz, 1H), 3.64 – 3.49 (m, 1H), 3.44 – 3.41 (m, 1H), 3.19 – 3.00 (m, 1H), 2.45 – 2.34 (m, 3H), 2.30 (dp, *J* = 17.6, 6.6 Hz, 1H), 2.15 – 2.10 (m, 1H), 1.96 (ddt, *J* = 13.5, 8.7, 4.3 Hz, 1H), 1.30 – 1.22 (m, 1H), 1.00 – 0.84 (m, 3H), 0.80 – 0.65 (m, 3H).

^{13}C NMR: (151 MHz, MeOD) δ [ppm] = 174.2, 170.9, 170.7, 165.4, 160.6, 157.8, 155.4, 153.0, 152.2, 149.1, 147.3, 143.7, 139.2, 135.4, 133.2, 133.0, 132.4, 129.9, 129.2, 128.5, 128.0, 127.7, 124.6, 124.6, 124.4, 124.4, 122.9, 118.4, 113.5, 102.5, 70.9, 70.8, 69.2, 67.4, 60.8, 60.1, 57.2, 45.1, 44.5, 39.4, 39.1, 30.1, 19.6, 19.0, 17.4, 17.3, 15.8.

LC/MS: m/z calculated for $C_{51}H_{54}N_{10}O_8S$ $[M+2H]^{2+}$: 484.2, found: 484.1.

Purity (HPLC): 97%.

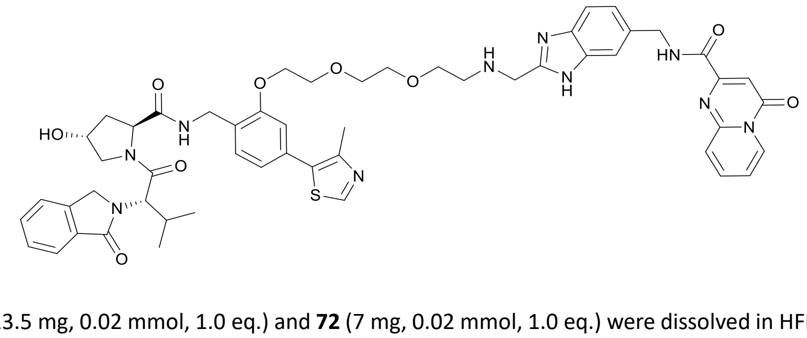

Compound **12**: **44** (13.5 mg, 0.02 mmol, 1.0 eq.) and **72** (7 mg, 0.02 mmol, 1.0 eq.) were dissolved in HFIP (200 µL). One pearl of mol-sieve was added, and the mixture was stirred at 20 °C for 16 h. The reaction was diluted with DCE (1 mL), and NaBH(OAc)$_3$ (10 mg, 0.05 mmol, 2.5 eq.) was added and incubated at 20 °C for 3 h. The reaction was terminated by the addition of MeOH (1 mL). The evaporation of solvent, purification by RP flash chromatography, and lyophilization afforded the product as a yellowish solid

Yield: 6 mg (30% of th.).

^{1}H NMR (600 MHz, MeO*D*) δ [ppm] = 9.02 – 8.81 (m, 2H), 8.76 (d, *J* = 32.5 Hz, 1H), 7.89 (s, 1H), 7.71 (dd, *J* = 43.1, 7.6 Hz, 2H), 7.60 – 7.13 (m, 9H), 7.03 – 6.96 (m, 1H), 6.88 (s, 1H), 4.79 (dd, *J* = 19.5, 10.9 Hz, 1H), 4.69 – 4.30 (m, 8H), 4.28 – 3.99 (m, 5H), 3.98 – 3.93 (m, 1H), 3.93 – 3.77 (m, 3H), 3.76 – 3.55 (m, 5H), 3.16 – 2.95 (m, 2H), 2.44 (d, *J* = 17.6 Hz, 1H), 2.33 (d, *J* = 43.7 Hz, 3H), 2.20 – 2.12 (m, 1H), 2.06 – 1.89 (m, 2H), 1.32 – 1.21 (m, 1H), 0.99 (dt, *J* = 20.6, 10.9 Hz, 3H), 0.77 (dd, *J* = 37.2, 6.4 Hz, 3H).

^{13}C NMR (151 MHz, MeOD) δ [ppm] = 174.2, 174.1, 171.1, 170.9, 170.8, 165.3, 160.5, 157.9, 155.4, 152.9, 152.2, 149.2, 149.1, 143.8, 143.7, 139.1, 133.3, 133.2, 133.1, 133.0, 132.4, 130.0, 129.9, 129.3, 129.2, 128.5, 128.2, 128.1, 127.7, 124.4, 123.0, 122.8, 118.4, 113.7, 102.5, 71.8, 71.3, 70.8, 69.2, 67.9, 60.7, 60.1, 60.1, 57.3, 57.2, 40.7, 39.4, 39.2, 30.2, 19.7, 19.0, 16.0, 15.9.

LC/MS: m/z calculated for $C_{53}H_{58}N_{10}O_{9}S$ $[M+2H]^{2+}$: 506.2, found: 506.1.

Purity (HPLC): 91%.

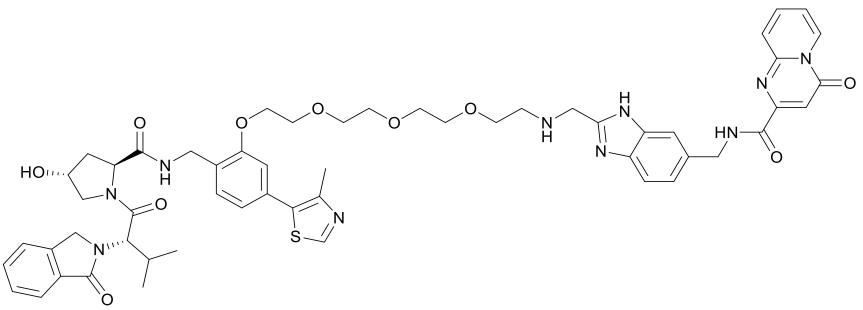

Compound **13**: **45** (14.5 mg, 0.02 mmol, 1.0 eq.) and **72** (7 mg, 0.02 mmol, 1.0 eq.) were solved in HFIP (200 µL). One pearl of mol-sieve was added, and the mixture was stirred at 20 °C for 16 h and diluted with DCE (1 mL). To this reaction, $NaBH(OAc)_3$ (10 mg, 0.05 mmol, 2.5 eq.) was added, and the mixture was incubated at 20 °C for 3 h. The reaction was subsequently quenched by the addition of MeOH (1 mL). The evaporation of solvent, purification by RP flash chromatography, and lyophilization afforded the product as a yellowish solid.

Yield: 2 mg (10% of th.).

^{1}H NMR: (600 MHz, MeO*D*) δ [ppm] = 8.98 (s, 1H), 8.82 (d, *J* = 32.5 Hz, 1H), 7.90 (d, *J* = 7.2 Hz, 1H), 7.76 – 7.67 (m, 2H), 7.59 – 7.37 (m, 5H), 7.36 – 7.27 (m, 2H), 7.20 (d, *J* = 26.1 Hz, 1H), 7.01 (d, *J* = 5.7 Hz, 1H), 6.90 (d, *J* = 7.4 Hz, 1H), 6.86 (s, 1H), 4.70 (s, 2H), 4.69 – 4.28 (m, 9H), 4.24 – 4.17 (m, 1H), 4.11 – 3.98 (m, 3H), 3.94 (d, *J* = 10.6 Hz, 1H), 3.89 – 3.69 (m, 4H), 3.67 – 3.45 (m, 7H), 3.14 – 3.03 (m, 1H), 2.87 (s, 1H), 2.46 – 2.28 (m, 4H), 2.15 (d, *J* = 7.3 Hz, 1H), 2.00 (d, *J* = 17.3 Hz, 1H), 1.36 – 1.22 (m, 2H), 1.00 (dd, *J* = 12.9, 6.2 Hz, 3H), 0.78 (dd, *J* = 22.7, 6.5 Hz, 3H).

^{13}C NMR: (151 MHz, MeOD) δ [ppm] = 170.9, 170.7, 170.7, 160.6, 158.0, 152.9, 152.2, 149.1, 143.8, 139.2, 133.3, 133.2, 132.4, 129.9, 129.9, 129.3, 129.2, 128.5, 128.2, 128.1, 127.8, 127.8, 124.5, 124.4, 122.8, 118.4, 113.7, 113.5, 102.5, 71.8, 71.7, 71.5, 71.5, 71.2, 70.8, 69.3, 67.8, 60.7, 60.1, 57.2, 44.6, 39.2, 30.2, 19.7, 19.0, 16.0, 15.9.

LC/MS: m/z calculated for $C_{55}H_{62}N_{10}O_{10}S$ $[M+2H]^{2+}$: 528.2, found: 528.1.

Purity (HPLC): 93%.

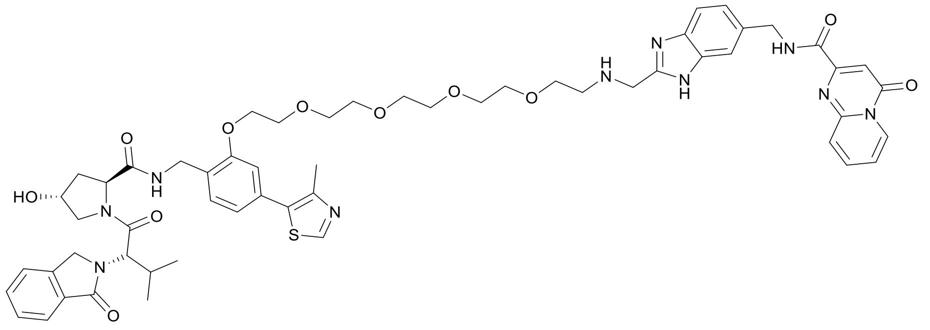

Compound **14**: **46** (15.3 mg, 0.02 mmol, 1.0 eq.) and **72** (7 mg, 0.02 mmol, 1.0 eq.) were solved in HFIP (200 µL). One pearl of mol sieve was added, and the mixture was stirred at 20 °C for 16 h and diluted with DCE (1 mL). To this reaction, $NaBH(OAc)_3$ (10 mg, 0.05 mmol, 2.5 eq.) was added, and the mixture was incubated at 20 °C for 3 h. The reaction was quenched by the addition of MeOH (1 mL). The evaporation of solvent, purification by RP flash chromatography, and lyophilization afforded the product as a yellowish solid.

Yield: 4 mg (18% of th.).

^{1}H NMR: (600 MHz, MeO*D*) δ [ppm] = 8.97 (s, 1H), 8.85 – 8.75 (m, 1H), 7.89 (d, *J* = 6.3 Hz, 1H), 7.69 (td, *J* = 27.1, 6.3 Hz, 2H), 7.60 – 7.35 (m, 5H), 7.35 – 7.14 (m, 3H), 7.03 – 6.97 (m, 1H), 6.95 – 6.75 (m, 2H), 4.78 (d, *J* = 10.3 Hz, 1H), 4.70 – 4.14 (m, 9H), 4.05 (d, *J* = 19.3 Hz, 3H), 3.94 (d, *J* = 10.6 Hz, 1H), 3.88 – 3.70 (m, 3H), 3.65 – 3.42 (m, 9H), 3.39 (s, 1H), 3.30 (s, 1H), 3.15 – 3.00 (m, 1H), 2.77 (d, *J* = 68.3 Hz, 2H), 2.47 – 2.26 (m, 4H), 2.15 (s, 1H), 2.04 – 1.95 (m, 1H), 1.33 – 1.19 (m, 1H), 1.04 – 0.89 (m, 3H), 0.85 – 0.71 (m, 3H).

^{13}C NMR: (151 MHz, MeOD) δ [ppm] = 172.8, 169.5, 169.2, 163.9, 159.2, 156.6, 154.0, 151.5, 150.8, 147.7, 142.4, 137.8, 132.0, 131.9, 131.8, 131.5, 131.0, 128.6, 127.8, 127.1, 126.8, 126.4, 123.0, 123.0, 121.6, 121.4, 117.0, 112.4, 112.2, 101.1, 70.3, 70.1, 70.0, 69.9, 69.7, 69.4, 69.4, 68.0, 67.9, 66.5, 59.3, 58.7, 55.8, 43.2, 39.2, 38.1, 38.0, 37.8, 28.8, 24.7, 18.3, 18.3, 17.6, 14.6, 14.5.

LC/MS: m/z calculated for $C_{57}H_{66}N_{10}O_{11}S$ $[M+2H]^{2+}$: 550.2, found: 550.1.

Purity (HPLC): 95%.

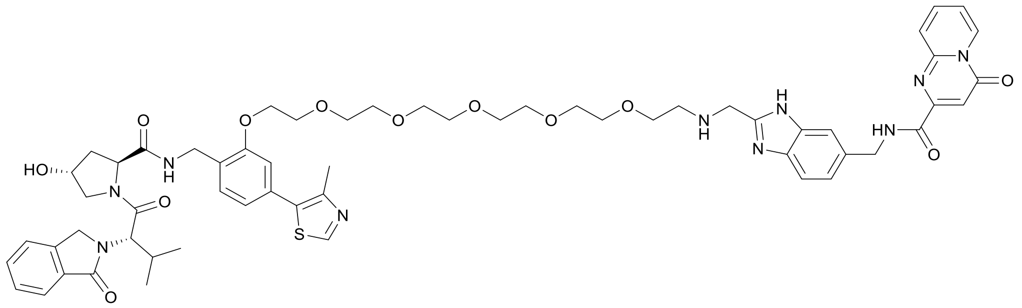

Compound **15**: **47** (16.2 mg, 0.02 mmol, 1.0 eq.) and **72** (7 mg, 0.02 mmol, 1.0 eq.) were solved in HFIP (200 µL). One pearl of mol-sieve was added, and the mixture was stirred at 20 °C for 16 h and diluted with DCE (1 mL). Then $NaBH(OAc)_3$ (10 mg, 0.05 mmol, 2.5 eq.) was added, and the mixture was incubated at 20 °C for 3 h. The reaction was quenched by the addition of MeOH (1 mL). The evaporation of solvent, purification by RP flash chromatography, and lyophilization afforded the product as a yellowish solid.

Yield: 4 mg (18% of th.).

^{1}H NMR: (600 MHz, MeO*D*) δ [ppm] = 8.98 (s, 1H), 8.82 (d, *J* = 28.3 Hz, 1H), 7.90 (s, 1H), 7.76 – 7.65 (m, 2H), 7.59 – 7.38 (m, 5H), 7.30 (dd, *J* = 33.1, 7.5 Hz, 2H), 7.22 (s, 1H), 7.02 (s, 1H), 6.96 – 6.83 (m, 2H), 4.78 (d, *J* = 10.6 Hz, 1H), 4.68 – 4.27 (m, 9H), 4.22 – 3.98 (m, 4H), 3.94 (d, *J* = 10.9 Hz, 1H), 3.90 – 3.69 (m, 4H), 3.67 – 3.41 (m, 14H), 3.29 (s, 2H), 2.79 (d, *J* = 77.2 Hz, 2H), 2.47 – 2.31 (m, 4H), 2.14 (d, *J* = 8.2 Hz, 1H), 2.04 – 1.97 (m, 1H), 1.32 – 1.22 (m, 1H), 1.00 (t, *J* = 6.5 Hz, 3H), 0.78 (dd, *J* = 15.8, 6.6 Hz, 3H).

^{13}C NMR: (151 MHz, MeOD) δ [ppm] = 174.2, 174.2, 170.9, 170.6, 165.3, 160.6, 158.0, 157.9, 155.4, 152.9, 152.2, 149.2, 149.1, 143.8, 139.2, 133.4, 133.3, 133.2, 132.9, 132.5, 130.0, 129.2, 128.5, 128.2, 127.8, 124.4, 122.8, 118.4, 113.6, 102.5, 71.7, 71.6, 71.5, 71.4, 71.3, 71.3, 71.2, 70.9, 70.8, 69.3, 67.9, 60.7, 60.1, 57.2, 49.6, 44.6, 40.5, 39.5, 39.4, 39.2, 30.2, 19.7, 19.0, 16.0, 15.9.

LC/MS: m/z calculated for $C_{59}H_{70}N_{10}O_{12}S$ $[M+2H]^{2+}$: 572.2, found: 572.2.

Purity (HPLC): 95%.

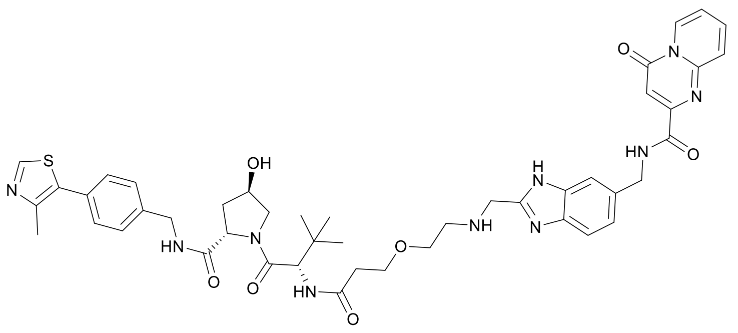

Compound **6**: **48** (22 mg, 0.04 mmol, 1.0 eq.), **72** (14 mg, 0.04 mmol, 1.0 eq.), and acetic acid (6 µL, 0.1 mmol, 2.5 eq.) were solved in HFIP (200 µL). One pearl of mol-sieve was added, and the mixture was stirred at 60 °C for 3 h and diluted with DCE (1 mL). $NaBH(OAc)_3$ (21 mg, 0.1 mmol, 2.5 eq.) was added, and the mixture was incubated for 24 h. The reaction was quenched by the addition of MeOH (1 mL). The evaporation of solvent, purification by RP flash chromatography, and lyophilization afforded the product.

Yield: 8 mg (23% of th.).

^{1}H NMR: (600 MHz, DMSO-*d*$_6$) δ [ppm] = 9.42 (t, *J* = 6.5 Hz, 1H), 9.01 (dd, *J* = 7.2, 1.6 Hz, 1H), 8.98 (s, 1H), 8.59 (t, *J* = 6.1 Hz, 1H), 8.10 – 8.04 (m, 2H), 7.76 (d, *J* = 8.9 Hz, 1H), 7.60 – 7.55 (m, 2H), 7.45 (td, *J* = 6.9, 1.4 Hz, 1H), 7.39 (d, *J* = 8.1 Hz, 2H), 7.35 (d, *J* = 8.2 Hz, 2H), 7.27 (dd, *J* = 8.3, 1.6 Hz, 1H), 6.89 (s, 1H), 4.60 (d, *J* = 6.4 Hz, 2H), 4.50 (d, *J* = 9.3 Hz, 1H), 4.47 (s, 2H), 4.41 (dt, *J* = 16.3, 7.3 Hz, 2H), 4.33 (tt, *J* = 4.2, 2.3 Hz, 1H), 4.19 (dd, *J* = 15.9, 5.5 Hz, 1H), 2.61 – 2.55 (m, 1H), 2.42 (s, 4H), 2.03 (dd, *J* = 13.0, 8.0 Hz, 1H), 1.88 (ddd, *J* = 13.0, 8.9, 4.5 Hz, 1H), 0.89 (s, 9H).

^{13}C NMR: (151 MHz, DMSO) δ [ppm] = 172.0, 170.2, 169.6, 163.0, 158.4, 158.2, 158.0, 153.9, 151.6, 150.6, 147.8, 146.8, 139.5, 138.6, 133.8, 131.2, 129.7, 128.7, 127.5, 127.5, 126.2, 122.7, 117.4, 117.3, 115.3, 100.9, 69.0, 66.6, 65.2, 62.0, 58.8, 56.5, 56.5, 46.3, 43.7, 43.0, 41.7, 38.1, 35.4, 35.2, 26.4, 16.0, 15.1.

LC/MS: m/z calculated for $C_{45}H_{52}N_{10}O_7S$ $[M+2H]^{2+}$: 439.2, found: 439.1.

Purity (HPLC): 99%.

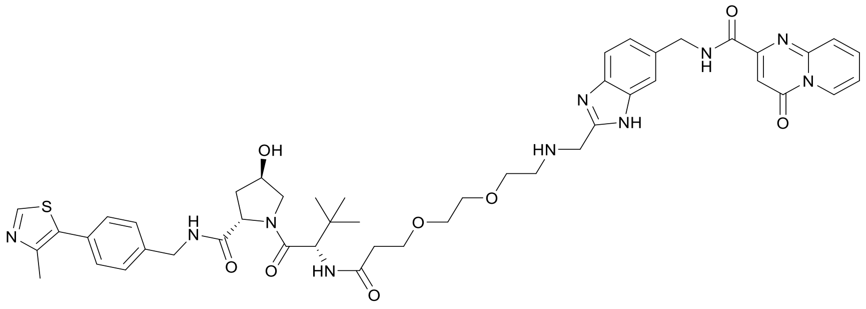

Compound **7**: **49** (11 mg, 0.02 mmol, 1.0 eq.) was solved in 0.5 mL DCE and 0.2 mL HFIP, then **72** (7 mg, 0.02 mmol, 1.0 eq.) was added and mixed until everything was solved. Ti(O$_i$Pr)$_4$ (50 µL, 0.165 mmol, 8.0 eq.) was added and incubated at 30 °C for 8 h. After that, another portion Ti(O$_i$Pr)$_4$ (50 µL, 0.165 mmol, 8.0 eq.) was added, and the mixture was incubated at 30 °C for 8 h. After the addition of 200 µL MeOH, the resulting solution was added to NaBH$_4$ (1 mg, 0.02 mmol, 1.0 eq.) and vortexed immediately. The solution was left at RT for 15 min, and then 10 µL TFA was added. Purification was done by flash chromatography and lyophilization to afford the product as an off-white solid.

Yield: 9 mg (49% of th.).

^{1}H NMR: (600 MHz, MeO*D*) δ [ppm] = 8.99 (d, *J* = 7.0 Hz, 1H), 8.78 (s, 1H), 7.90 (t, *J* = 7.6 Hz, 1H), 7.73 (d, *J* = 8.8 Hz, 1H), 7.53 (s, 1H), 7.49 – 7.43 (m, 1H), 7.32 (t, *J* = 6.7 Hz, 3H), 7.27 (d, *J* = 8.1 Hz, 2H), 7.23 (d, *J* = 6.9 Hz, 1H), 7.00 (d, *J* = 12.9 Hz, 1H), 4.64 (s, 2H), 4.57 (s, 1H), 4.50 (t, *J* = 8.3 Hz, 1H), 4.43 – 4.36 (m, 2H), 4.26 (d, *J* = 15.4 Hz, 1H), 4.15 (s, 2H), 3.80 (d, *J* = 10.8 Hz, 1H), 3.71 – 3.63 (m, 3H), 3.57 (d, *J* = 35.0 Hz, 6H), 2.95 (s, 2H), 2.49 – 2.42 (m, 2H), 2.36 (s, 3H), 2.15 (dd, *J* = 12.8, 7.5 Hz, 1H), 1.99 (ddd, *J* = 13.2, 9.5, 3.7 Hz, 1H), 0.93 (s, 9H).

^{13}C NMR: (151 MHz, MeOD) δ [ppm] = 174.4, 173.8, 172.2, 165.3, 160.6, 155.4, 152.9, 152.2, 149.0, 140.2, 139.1, 134.6, 133.3, 131.5, 130.3, 128.9, 128.5, 127.8, 124.0, 118.4, 102.5, 71.3, 71.1, 68.3, 60.9, 59.0, 58.1, 44.6, 43.7, 39.0, 37.2, 36.8, 27.0, 21.9, 15.8.

LC/MS: m/z calculated for $C_{47}H_{56}N_{10}O_8S$ $[M+2H]^{2+}$: 461.2, found: 461.1.

Purity (HPLC): 95%.

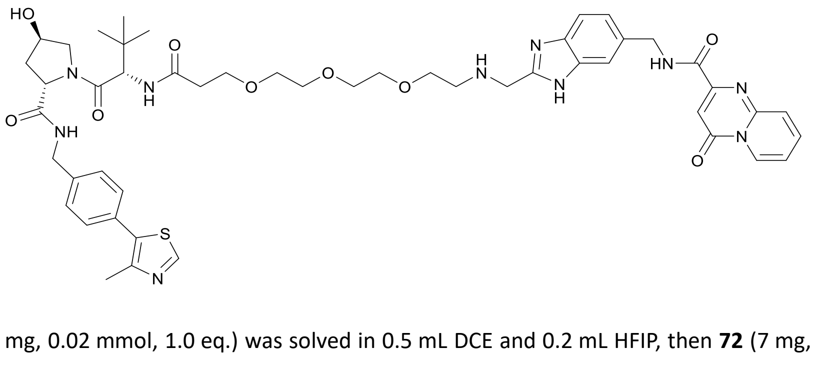

Compound **8**: **50** (12 mg, 0.02 mmol, 1.0 eq.) was solved in 0.5 mL DCE and 0.2 mL HFIP, then **72** (7 mg, 0.02 mmol, 1.0 eq.) was added and mixed until everything was solved. Ti(O$_i$Pr)$_4$ (50 µL, 0.165 mmol, 8.0 eq.) was added and incubated at 30 °C for 8 h. After that, another portion Ti(O$_i$Pr)$_4$ (50 µL, 0.165 mmol, 8.0 eq.) was added and incubated at 30 °C for 8 h. After the addition of 200 µL MeOH, the resulting solution was added to NaBH$_4$ (1 mg, 0.02 mmol, 1.0 eq.) and vortexed immediately. The solution was left at RT for 15 min, and then 10 µL TFA was added. Purification was done by flash chromatography and subsequent lyophilization to afford the product as an off-white solid.

Yield: 8 mg (41% of th.).

^{1}H NMR: (600 MHz, MeO*D*) δ [ppm] = 9.00 (d, *J* = 7.0 Hz, 1H), 8.80 (s, 1H), 7.94 – 7.88 (m, 1H), 7.75 (d, *J* = 8.9 Hz, 1H), 7.53 (s, 1H), 7.47 (d, *J* = 7.8 Hz, 1H), 7.41 – 7.29 (m, 5H), 7.24 (d, *J* = 7.8 Hz, 1H), 7.03 (s, 1H), 4.65 (s, 2H), 4.56 (s, 1H), 4.53 – 4.48 (m, 1H), 4.45 (d, *J* = 15.7 Hz, 2H), 4.27 (d, *J* = 15.5 Hz, 1H), 4.14 (s, 2H), 3.81 (d, *J* = 10.9 Hz, 1H), 3.72 (dd, *J* = 10.9, 3.6 Hz, 1H), 3.68 – 3.47 (m, 12H), 2.94 (s, 2H), 2.45 (td, *J* = 13.4, 11.6, 5.5 Hz, 1H), 2.38 (s, 4H), 2.16 (dd, *J* = 12.9, 7.6 Hz, 1H), 2.01 (ddd, *J* = 16.2, 8.3, 3.6 Hz, 1H), 0.97 (d, *J* = 19.2 Hz, 9H).

^{13}C NMR: (151 MHz, MeOD) δ [ppm] = 174.5, 173.7, 172.1, 165.3, 160.6, 155.4, 152.9, 152.2, 149.0, 140.2, 139.1, 134.6, 133.4, 131.4, 130.3, 128.9, 128.5, 127.8, 124.0, 118.4, 102.5, 71.5, 71.4, 71.3, 71.2, 71.1, 68.2, 60.9, 59.0, 58.0, 44.6, 43.7, 39.0, 37.2, 36.8, 27.0, 21.9, 19.2, 15.8.

LC/MS: m/z calculated for $C_{49}H_{60}N_{10}O_{9}S$ $[M+2H]^{2+}$: 483.2, found: 483.1.

Purity (HPLC): 97%.

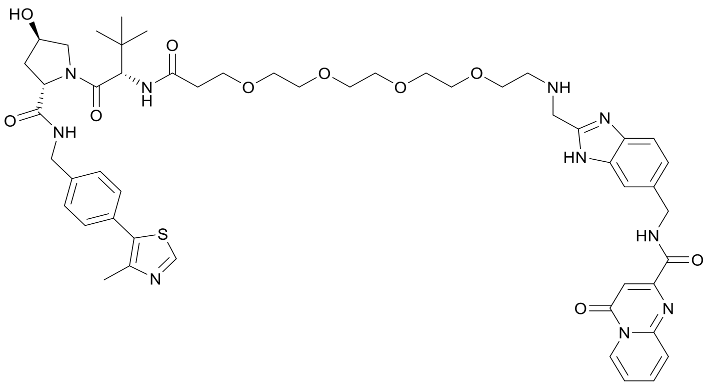

Compound **9**: **51** (13 mg, 0.02 mmol, 1.0 eq.) was solved in 0.5 mL DCE and 0.2 mL HFIP, then **72** (7 mg, 0.02 mmol, 1.0 eq.) was added and mixed until everything was solved. $Ti(O_iPr)_4$ (50 µL, 0.165 mmol, 8.0 eq.) was added, and the mixture was incubated at 30 °C for 8 h. After that, another portion $Ti(O_iPr)_4$ (50 µL, 0.165 mmol, 8.0 eq.) was added, and the mixture was incubated at 30 °C for 8 h. After the addition of 200 µL MeOH, the resulting solution was added to $NaBH_4$ (1 mg, 0.02 mmol, 1.0 eq.) and vortexed immediately. The solution was left at RT for 15 min, and then 10 µL TFA was added. Purification was done by flash chromatography and lyophilization to afford the product as an off-white solid.

Yield: 6 mg (30% of th.).

^{1}H NMR: (600 MHz, MeO*D*) δ [ppm] = 9.00 (d, *J* = 7.0 Hz, 1H), 8.80 (s, 1H), 7.94 – 7.89 (m, 1H), 7.75 (d, *J* = 8.9 Hz, 1H), 7.54 (s, 1H), 7.47 (d, *J* = 7.7 Hz, 1H), 7.40 – 7.30 (m, 5H), 7.24 (d, *J* = 8.0 Hz, 1H), 7.03 (d, *J* = 8.7 Hz, 1H), 4.66 (s, 2H), 4.56 (s, 1H), 4.53 – 4.41 (m, 4H), 4.30 – 4.25 (m, 1H), 4.15 (s, 2H), 3.81 (d, *J* = 11.1 Hz, 1H), 3.74 – 3.66 (m, 2H), 3.63 – 3.45 (m, 18H), 2.95 (s, 2H), 2.49 – 2.41 (m, 2H), 2.40 – 2.34 (m, 4H), 2.16 (dd, *J* = 12.9, 7.6 Hz, 1H), 2.01 (ddd, *J* = 16.9, 8.6, 4.0 Hz, 1H), 0.96 (s, 9H).

^{13}C NMR: (151 MHz, MeOD) δ [ppm] = 174.5, 173.7, 172.1, 165.3, 160.6, 155.4, 152.9, 152.2, 149.0, 140.2, 139.1, 134.6, 133.4, 131.4, 130.4, 130.3, 128.9, 128.5, 127.8, 123.9, 118.4, 102.5, 71.4, 71.3, 71.1, 70.8, 69.7, 68.2, 67.1, 60.9, 59.0, 58.0, 45.7, 44.6, 43.7, 40.5, 39.0, 37.2, 36.8, 27.0, 19.2, 15.9.

LC/MS: $C_{51}H_{64}N_{10}O_{10}S$ $[M+2H]^{2+}$: 502.1, found: 502.2.

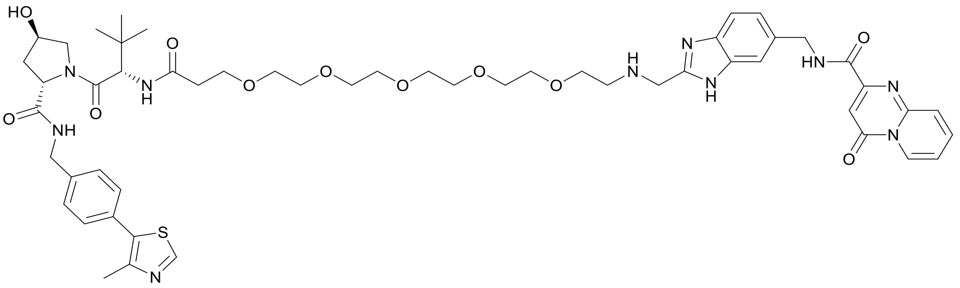

Compound **10**: **52** (15 mg, 0.02 mmol, 1.0 eq.) was solved in 0.5 mL DCE and 0.2 mL HFIP, then **72** (7 mg, 0.02 mmol, 1.0 eq.) was added and mixed until everything was solved. $Ti(O_iPr)_4$ (50 µL, 0.165 mmol, 8.0 eq.) was added and incubated at 30 °C for 8 h. After that, another portion $Ti(O_iPr)_4$ (50 µL, 0.165 mmol, 8.0 eq.) was added, and the mixture was incubated at 30 °C for 8 h. After adding 200 µL MeOH, the resulting solution was added to $NaBH_4$ (1 mg, 0.02 mmol, 1.0 eq.) and vortexed immediately. The solution was left at RT for 15 min, and then 10 µL TFA was added. Purification was done by flash chromatography and lyophilization to afford the product as an off-white solid.

Yield: 3 mg (14% of th.).

^{1}H NMR: (600 MHz, MeO*D*) δ [ppm] = 9.03 (d, *J* = 7.1 Hz, 1H), 8.81 (s, 1H), 7.95 – 7.91 (m, 1H), 7.77 (d, *J* = 8.9 Hz, 1H), 7.54 (s, 1H), 7.47 (d, *J* = 8.1 Hz, 1H), 7.42 – 7.30 (m, 5H), 7.24 (d, *J* = 8.2 Hz, 1H), 7.06 (s, 1H), 4.69 (d, *J* = 20.7 Hz, 2H), 4.59 (d, *J* = 10.1 Hz, 1H), 4.55 – 4.40 (m, 4H), 4.28 (d, *J* = 15.6 Hz, 1H), 4.05 (s, 1H), 3.85 – 3.81 (m, 1H), 3.78 – 3.45 (m, 20H), 2.83 (s, 1H), 2.51 – 2.45 (m, 1H), 2.41 – 2.36 (m, 3H), 2.16 (dd, *J* = 13.1, 7.4 Hz, 1H), 2.02 (ddd, *J* = 13.3, 9.3, 4.5 Hz, 1H), 1.31 – 1.22 (m, 2H), 0.98 (d, *J* = 13.0 Hz, 9H).

^{13}C NMR: (151 MHz, MeOD) δ [ppm] = 174.5, 173.7, 172.1, 165.3, 160.6, 155.5, 152.9, 140.3, 139.1, 133.4, 131.4, 130.4, 130.3, 128.9, 128.5, 127.8, 118.4, 102.6, 71.5, 71.3, 71.2, 71.1, 68.3, 60.8, 58.9, 58.0, 49.6, 44.6, 43.7, 39.0, 37.3, 36.8, 27.0, 15.9.

LC/MS: m/z calculated for $C_{53}H_{68}N_{10}O_{11}S$ $[M+2H]^{2+}$: 527.2, found: 527.1.

Purity (HPLC): 98%.

Synthesis of 2nd generation PROTACs

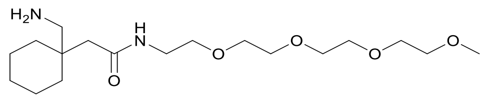

Compound **73**: A solution of OMe-PEG4-NH_2 (21 mg, 0.1 mmol, 1.0 eq.), TBTU (32 mg, 0.1 mmol, 1.0 eq.) and Boc-gabapentin (27 mg, 0.1 mmol, 1.0 eq.) in dry DMF (1 mL) was treated with DIPEA (52 µL, 0.3 mmol, 3.0 eq.), and the suspension was stirred at RT for 16 h. The suspension was concentrated in vacuo, and the resulting residue was purified by RP flash chromatography and lyophilized. The intermediate was resolved in DCM/TFA (1+1 mL) and stirred at RT for 30 min. Evaporation and lyophilization afforded the product as a colorless oil.

Yield: 31 mg (86% of th.).

^{1}H NMR: (300 MHz, MeO*D*) δ [ppm] = 3.62 – 3.45 (m, 15H), 3.35 (d, *J* = 5.3 Hz, 2H), 2.91 (s, 2H), 2.44 (s, 2H), 1.58 – 1.23 (m, 12H).

^{13}C NMR: (75 MHz, MeOD) δ [ppm] = 174.2, 72.9, 71.6, 71.5, 71.5, 71.3, 71.2, 70.4, 59.1, 44.1, 40.5, 36.5, 34.6, 26.7, 22.2.

LC/MS: m/z calculated for $C_{18}H_{36}N_2O_5$ $[M+H]^+$: 361.3, found: 361.1.

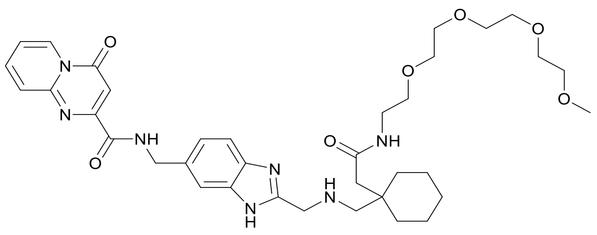

Compound **26**: **73** (7.2 mg, 0.02 mmol, 1.0 eq.) and **72** (7 mg, 0.02 mmol, 1.0 eq.) were dissolved in DCE/HFIP (500+100 µL) and $Ti(O_iPr)_4$ (50 µL, 0.165 mmol, 8.0 eq). The solution was stirred at RT for 16 h. Subsequently, $Ti(O_iPr)_4$ (50 µL, 0.165 mmol, 8.0 eq), MeOH (200 µL), and $NaBH_4$ (1 mg, 0.02 mmol, 1.0 eq.) were added. The reaction was incubated at RT for 30 min and quenched with TFA (10 µL). After evaporation and purification by RP flash chromatography, lyophilization afforded the product as a colorless solid.

Yield: 7 mg (51% of th.).

^{1}H NMR: (600 MHz, MeO*D*) δ [ppm] = 9.01 (d, *J* = 7.0 Hz, 1H), 7.93 (ddd, *J* = 8.4, 6.8, 1.3 Hz, 1H), 7.76 (d, *J* = 8.9 Hz, 1H), 7.53 (s, 1H), 7.48 (d, *J* = 7.2 Hz, 1H), 7.36 – 7.32 (m, 1H), 7.24 (d, *J* = 8.1 Hz, 1H), 7.03 (s, 1H), 4.65 (d, *J* = 15.8 Hz, 2H), 4.21 (s, 2H), 3.68 – 3.54 (m, 1H), 3.54 – 3.44 (m, 10H), 3.44 – 3.38 (m, 4H), 3.30 (d, *J* = 20.4 Hz, 3H), 2.83 (s, 2H), 2.38 (s, 2H), 1.40 (d, *J* = 51.7 Hz, 11H).

^{13}C NMR: (151 MHz, MeOD) δ [ppm] = 174.6, 165.3, 160.6, 155.4, 152.3, 139.2, 134.6, 128.5, 127.8, 124.0, 118.4, 102.5, 72.9, 71.5, 71.5, 71.5, 71.3, 71.2, 70.5, 59.1, 44.6, 40.4, 37.6, 35.3, 27.0, 23.8, 22.4.

LC/MS: m/z calculated for $C_{36}H_{49}N_7O_7$ $[M+H]^+$: 692.4, found: 692.2.

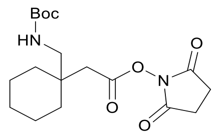

Compound **74**: Boc-gabapentin (700 mg, 2.6 mmol, 1.0 eq.) was dissolved in DCM (20 mL), and *N*-hydroxysuccinimide (300 mg, 2.6 mmol, 1.0 eq.), DMAP (5 mg, cat.), and DCC (500 mg, 2.45 mmol, 0.9 eq.) were added. The reaction was stirred at RT for 16 h, filtered over silica, and evaporated to give the product as a colorless oil.

Yield: 451 mg (47% of th.).

^{1}H NMR: (300 MHz, C*D*Cl$_3$) δ [ppm] = 5.24 (s, 1H), 3.15 (d, *J* = 6.8 Hz, 2H), 2.76 (s, 4H), 2.49 (s, 2H), 1.36 (s, 19H).

^{13}C NMR: (75 MHz, *C*DCl$_3$) δ [ppm] = 169.2, 167.1, 156.4, 79.0, 53.5, 46.6, 38.7, 37.4, 33.6, 28.3, 25.7, 25.6, 21.4.

LC/MS: m/z calculated for $C_{18}H_{28}N_2O_6$ [M - Boc+H]$^+$: 269.2, found: 269.0.

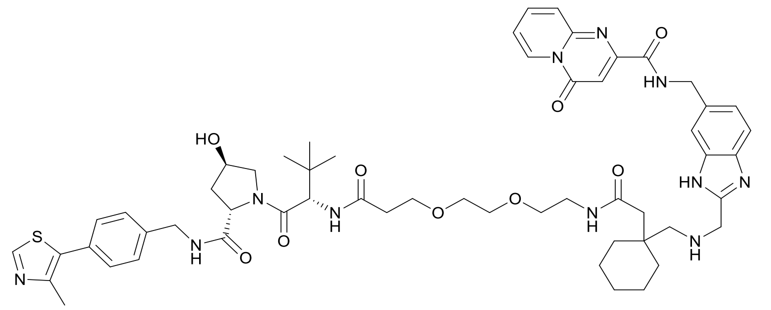

Compound **27**: **49** (11.8 mg, 0.02 mmol, 1.0 eq.) was dissolved in DMF (100 µL), DIPEA (10 µL, 0.06 mmol, 3.0 eq.), and **74** (7.3 mg, 0.02 mmol, 1.0 eq.) were added, and the mixture was stirred for at RT for 3 h. The solvent was evaporated, and the residue resolved in DCM/TFA (200+200 µL). The reaction was then stirred at RT for 30 min, the solvent evaporated, and the mix was purified by RP flash chromatography. The resulting intermediate (11.5 mg, 0.02 mmol, 1.0 eq.) was dissolved in DCE/HFIP (500+100 µL), and **72** (7 mg, 0.02 mmol, 1.0 eq.) and Ti(O$_i$Pr)$_4$ (50 µL, 0.165 mmol, 8.0 eq.) were added, and incubated at RT for 16 h. Subsequently, MeOH (200 µL) and NaBH$_4$ (1 mg, 0.02 mol, 1.0 eq.) were added at 0 °C, the reaction was incubated at RT for 30 min and quenched by the addition of 0.1% TFA (10 µL). The organic solvents were evaporated, and the residue was purified by RP flash chromatography.

Yield: 4 mg (19% of th.).

^{1}H NMR: (400 MHz, MeO*D*) δ [ppm] = 9.09 (d, *J* = 7.2 Hz, 1H), 8.87 (s, 1H), 8.03 – 7.97 (m, 1H), 7.86 (dd, *J* = 20.5, 8.9 Hz, 2H), 7.72 – 7.57 (m, 2H), 7.51 – 7.34 (m, 6H), 7.09 (d, *J* = 17.4 Hz, 1H), 4.75 (s, 2H), 4.66 (d, *J* = 8.7 Hz, 1H), 4.60 – 4.49 (m, 5H), 4.35 (dd, *J* = 15.2, 4.9 Hz, 1H), 3.89 (d, *J* = 10.8 Hz, 1H), 3.80 (dd, *J* = 11.2, 3.8 Hz, 1H), 3.75 – 3.67 (m, 2H), 3.63 – 3.54 (m, 5H), 3.49 (t, *J* = 5.4 Hz, 2H), 3.26 – 3.19 (m, 3H), 2.56 (d, *J* = 11.8 Hz, 3H), 2.50 – 2.44 (m, 4H), 2.26 – 2.19 (m, 1H), 2.12 – 2.04 (m, 1H), 1.55 (s, 7H), 1.48 – 1.39 (m, 4H), 1.33 (t, *J* = 7.3 Hz, 3H), 1.04 (d, *J* = 9.5 Hz, 9H).

^{13}C NMR: (101 MHz, MeOD) δ [ppm] = 174.7, 174.4, 173.6, 172.2, 165.5, 160.6, 155.5, 152.9, 152.3, 140.2, 139.2, 131.5, 130.4, 130.3, 129.4, 129.0, 128.5, 127.8, 124.6, 118.7, 118.4, 102.6, 71.4, 71.2, 71.1, 70.4, 68.3, 60.9, 58.9, 58.0, 47.9, 45.8, 44.6, 43.7, 40.7, 39.0, 37.3, 37.0, 36.8, 34.8, 27.0, 26.7, 22.3, 21.9, 17.7, 17.5, 17.3, 17.1, 15.8, 9.2.

LC/MS: m/z calculated for $C_{56}H_{71}N_{11}O_9S$ [M+2H]$^{2+}$: 537.8, found: 537.7.

Purity (HPLC): 95%.

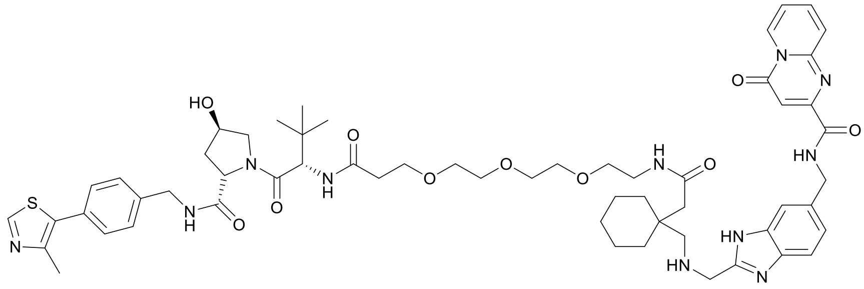

Compound **28**: **50** (15.8 mg, 0.025 mmol, 1.0 eq.) was dissolved in DMF (100 µL), DIPEA (10 µL, 0.06 mmol, 2.4 eq.), and **74** (9.1 mg, 0.025 mmol, 1.0 eq.) were added and the mixture was stirred at RT for 3 h. The solvent was evaporated, and the mixture was resolved in DCM/TFA (200+200 µL). The reaction was stirred at RT for 30 min, the solvent evaporated, and the mixture was purified by RP flash chromatography. The intermediate (12 mg, 0.02 mmol, 1.0 eq.) was dissolved in DCE/HFIP (500+100 µL), and **72** (7 mg, 0.02 mmol, 1.0 eq.) and $Ti(O_iPr)_4$ (50 µL, 0.165 mmol, 8.0 eq.) were added. This mixture was incubated at RT for 16 h. Subsequently, MeOH (200 µL) and $NaBH_4$ (1 mg, 0.02 mmol, 1.0 eq.) were added at 0 °C, the reaction was incubated at RT for 30 min and quenched by the addition of 0.1% TFA (10 µL). The organic solvents were evaporated, and the residue was purified by RP flash chromatography.

Yield: 3 mg (11% of th.).

^{1}H NMR: (400 MHz, MeO*D*) δ [ppm] = 9.10 (d, *J* = 6.6 Hz, 1H), 8.04 – 7.98 (m, 1H), 7.87 (dd, *J* = 18.4, 8.7 Hz, 2H), 7.68 (s, 1H), 7.62 (d, *J* = 8.2 Hz, 1H), 7.50 – 7.35 (m, 7H), 7.12 (s, 1H), 4.75 (s, 2H), 4.66 (d, *J* = 5.7 Hz, 1H), 4.56 (dd, *J* = 18.1, 9.7 Hz, 6H), 4.35 (d, *J* = 15.5 Hz, 1H), 3.89 (d, *J* = 11.2 Hz, 1H), 3.83 – 3.67 (m, 4H), 3.58 (dt, *J* = 13.7, 5.8 Hz, 12H), 3.48 (dt, *J* = 10.1, 5.4 Hz, 4H), 3.26 – 3.13 (m, 4H), 2.58 (q, *J* = 11.1, 9.1 Hz, 3H), 2.48 (d, *J* = 11.7 Hz, 4H), 2.26 – 2.17 (m, 2H), 2.13 – 2.05 (m, 1H), 1.88 – 1.81 (m, 1H), 1.75 – 1.69 (m, 1H), 1.61 (d, *J* = 5.7 Hz, 2H), 1.53 (d, *J* = 12.3 Hz, 9H), 1.44 (d, *J* = 6.3 Hz, 3H), 1.33 (t, *J* = 7.3 Hz, 3H), 1.04 (d, *J* = 6.7 Hz, 9H).

^{13}C NMR: (101 MHz, MeOD) δ [ppm] = 174.7, 174.5, 173.7, 172.2, 160.6, 140.3, 139.2, 135.5, 130.3, 129.0, 128.6, 127.8, 124.7, 118.4, 71.8, 71.6, 71.5, 71.3, 71.2, 71.0, 70.3, 68.3, 62.8, 60.9, 59.0, 58.0, 57.7, 57.5, 45.7, 45.6, 44.6, 43.7, 40.7, 39.0, 37.7, 37.3, 37.0, 36.8, 34.8, 30.8, 30.4, 28.1, 27.3, 27.0, 26.8, 26.7, 23.9, 17.5, 17.3, 17.1, 15.8, 9.2.

LC/MS: m/z calculated for $C_{58}H_{75}N_{11}O_{10}S$ $[M+2H]^{2+}$: 559.8, found: 559.7.

Purity (HPLC): 99%.

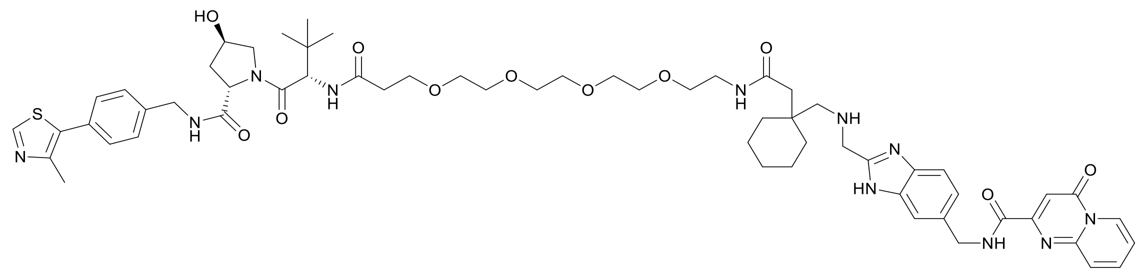

Compound **29**: **51** (24 mg, 0.035 mmol, 1.0 eq.) was dissolved in DMF (100 µL), DIPEA (10 µL, 0.06 mmol, 1.7 eq.), and **74** (13 mg, 0.035 mmol, 1.0 eq.) were added and the reaction was stirred at RT for 3 h. The solvent was evaporated, and the resulting residue was resolved in DCM/TFA (200+200 µL). The reaction was stirred at RT for 30 min, the solvent evaporated, and the residue purified by RP flash chromatography. The intermediate (21 mg, 0.03 mmol, 1.5 eq.) was dissolved in DCE/HFIP (500+100 µL), and **72** (7 mg, 0.02 mmol, 1.0 eq.) and $Ti(O_iPr)_4$ (50 µL, 0.165 mmol, 5.5 eq.) were added, and the mixture was incubated at RT for 16 h. Subsequently, MeOH (200 µL) and $NaBH_4$ (1 mg, 0.02 mmol, 1.0 eq.) were added at 0 °C, subsequently, the reaction was incubated at RT for 30 min and quenched by the addition of 0.1% TFA (10 µL). The organic solvents were evaporated, and the residue was purified by RP flash chromatography.

Yield: 4 mg (10% of th.).

^{1}H NMR: (400 MHz, MeO*D*) δ [ppm] = 9.10 (t, *J* = 6.8 Hz, 1H), 8.93 (s, 1H), 8.04 (dt, *J* = 24.3, 7.6 Hz, 1H), 7.87 (dd, *J* = 24.4, 8.8 Hz, 1H), 7.76 – 7.58 (m, 2H), 7.49 – 7.36 (m, 6H), 7.09 (d, *J* = 16.8 Hz, 1H), 4.75 (s, 2H), 4.65 (s, 1H), 4.60 – 4.47 (m, 5H), 4.35 (d, *J* = 15.5 Hz, 1H), 3.89 (d, *J* = 10.9 Hz, 1H), 3.79 (dd, *J* = 11.0, 3.7 Hz, 1H), 3.70 (dt, *J* = 9.7, 5.4 Hz, 2H), 3.64 – 3.55 (m, 13H), 3.48 (dq, *J* = 10.0, 5.7 Hz, 4H), 3.22 (d, *J* = 7.5 Hz, 2H), 2.58 (d, *J* = 9.2 Hz, 3H), 2.48 (d, *J* = 9.5 Hz, 3H), 2.21 (q, *J* = 10.1, 7.1 Hz, 1H), 2.08 (ddd, *J* = 13.1, 9.1, 4.3 Hz, 1H), 1.84 (dt, *J* = 14.3, 6.6 Hz, 1H), 1.71 (dt, *J* = 13.2, 6.4 Hz, 1H), 1.66 – 1.48 (m, 9H), 1.43 (d, *J* = 6.2 Hz, 3H), 1.32 (t, *J* = 7.3 Hz, 1H), 1.04 (d, *J* = 6.4 Hz, 9H).

^{13}C NMR: (101 MHz, MeOD) δ [ppm] = 174.5, 173.7, 172.2, 160.6, 140.3, 139.2, 130.3, 129.0, 128.6, 127.8, 124.8, 118.8, 118.4, 71.7, 71.6, 71.5, 71.4, 71.2, 71.1, 71.0, 70.3, 68.3, 62.8, 60.9, 59.0, 58.0, 45.6, 44.5, 43.7, 40.7, 39.0, 37.3, 37.0, 36.8, 34.8, 34.8, 30.8, 30.4, 28.1, 27.3, 27.1, 26.7, 22.2, 21.9, 15.8, 9.2.

LC/MS: m/z calculated for $C_{60}H_{79}N_{11}O_{11}S$ $[M+2H]^{2+}$: 581.8, found: 581.7.

Purity (HPLC): 95%.

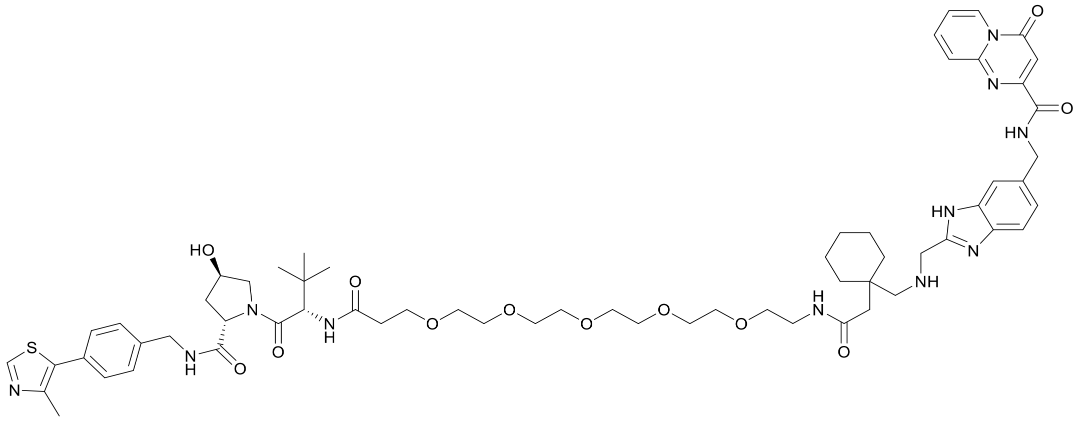

Compound **30**: **52** (21 mg, 0.03 mmol, 1.0 eq.) was dissolved in DMF (100 µL), DIPEA (10 µL, 0.06 mmol, 2.0 eq.), and **74** (11 mg, 0.03 mmol, 1.0 eq.) were added and the mixture was stirred at RT for 3 h. The solvent was evaporated, and the resulting residue was resolved in DCM/TFA (200+200 µL). The reaction was stirred at RT for 30 min, evaporated, and purified by RP flash chromatography. The product (18 mg, 0.02 mmol, 1.0 eq.) was dissolved in DCE/HFIP (500+100 µL), and **72** (7 mg, 0.02 mmol, 1.0 eq.) and $Ti(O_iPr)_4$ (50 µL, 0.165 mmol, 8.0 eq.) were added, and incubated at RT for 16 h. Subsequently, MeOH (200 µL) and $NaBH_4$ (1 mg, 0.02 mmol, 1.0 eq.) were added at 0 °C, the reaction was incubated at RT for 30 min and quenched by the addition of 0.1% TFA (10 µL). The organic solvents were evaporated, and the residue was purified by RP flash chromatography.

Yield: 4 mg (11% of th.).

^{1}H NMR: (400 MHz, MeO*D*) δ [ppm] = 9.16 – 8.82 (m, 2H), 8.01 (t, *J* = 7.3 Hz, 1H), 7.85 (d, *J* = 8.4 Hz, 1H), 7.74 – 7.59 (m, 2H), 7.43 (dt, *J* = 14.8, 7.8 Hz, 6H), 7.12 (s, 1H), 4.75 (s, 2H), 4.65 (s, 1H), 4.60 – 4.48 (m, 5H), 4.35 (d, *J* = 15.5 Hz, 1H), 3.89 (d, *J* = 10.7 Hz, 1H), 3.80 (dd, *J* = 11.0, 3.6 Hz, 1H), 3.70 (dt, *J* = 10.0, 5.8 Hz, 2H), 3.59 (dd, *J* = 11.6, 4.1 Hz, 17H), 3.48 (dq, *J* = 10.1, 5.3 Hz, 3H), 3.21 (s, 1H), 2.62 – 2.52 (m, 3H), 2.48 (d, *J* = 7.4 Hz, 3H), 2.21 (dd, *J* = 17.6, 10.2 Hz, 1H), 2.09 (td, *J* = 13.1, 11.0, 4.4 Hz, 1H), 1.85 (dt, *J* = 14.3, 6.7 Hz, 1H), 1.75 – 1.69 (m, 1H), 1.61 (s, 1H), 1.55 (s, 7H), 1.47 – 1.38 (m, 3H), 1.33 (t, *J* = 7.3 Hz, 1H), 1.04 (d, *J* = 6.1 Hz, 9H).

^{13}C NMR: (101 MHz, MeOD) δ [ppm] = 174.7, 174.5, 174.3, 173.7, 172.2, 160.6, 140.3, 139.2, 131.5, 130.4, 129.0, 128.6, 127.8, 124.7, 118.4, 71.7, 71.6, 71.6, 71.5, 71.4, 71.2, 71.1, 71.0, 70.3, 68.3, 62.8, 60.9, 59.0, 58.0, 50.0, 45.8, 45.7, 45.6, 44.6, 43.7, 40.7, 39.0, 37.7, 37.4, 37.0, 36.8, 36.5, 34.8, 34.8, 30.8, 30.4, 28.1, 27.3, 27.1, 26.8, 26.7, 23.9, 22.3.

LC/MS: m/z calculated for $C_{62}H_{83}N_{11}O_{12}S$ $[M+2H]^{2+}$: 603.8, found: 603.7.

Purity (HPLC): 96%.

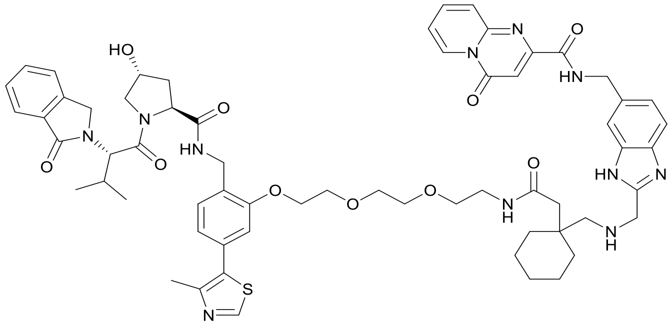

Compound **31**: **44** (17.7 mg, 0.025 mmol, 1.0 eq.) was dissolved in DMF (500 µL), DIPEA (20 µL, 0.12 mmol, 4.8 eq.), and **74** (9.3 mg, 0.025 mmol, 1.0 eq.) were added and the mixture was stirred at RT for 3 h. The solvent was evaporated, and the resulting residue was resolved in DCM/TFA (200+200 µL). The reaction was stirred at RT for 30 min, the solvent evaporated, and the mixture was purified by RP flash chromatography. The residue (13 mg, 0.02 mmol, 1.0 eq.) was dissolved in DCE/HFIP (500+100 µL), and **72** (7 mg, 0.02 mmol, 1.0 eq.) and $Ti(O_iPr)_4$ (50 µL, 0.165 mmol, 8.0 eq.) were added. The reaction was incubated at RT for 16 h. Subsequently, MeOH (200 µL) and $NaBH_4$ (1 mg, 0.02 mmol, 1.0 eq.) were added at 0 °C. The reaction was incubated at RT for 30 min and quenched by the addition of 0.1% TFA (10 µL). The organic solvents were evaporated, and the residue was purified by RP flash chromatography.

Yield: 5 mg (17% of th.).

^{1}H NMR: (400 MHz, MeO*D*) δ [ppm] = 9.06 (d, *J* = 7.1 Hz, 1H), 8.91 (s, 1H), 7.98 (t, *J* = 7.8 Hz, 1H), 7.78 (dd, *J* = 15.1, 8.1 Hz, 2H), 7.68 (s, 1H), 7.60 (t, *J* = 7.5 Hz, 2H), 7.54 (d, *J* = 7.5 Hz, 1H), 7.48 (t, *J* = 7.6 Hz, 1H), 7.40 (dt, *J* = 11.9, 8.1 Hz, 3H), 7.06 (d, *J* = 10.5 Hz, 1H), 7.02 (d, *J* = 3.9 Hz, 2H), 4.73 (s, 2H), 4.61 – 4.54 (m, 2H), 4.52 (d, *J* = 7.6 Hz, 3H), 4.46 (d, *J* = 3.4 Hz, 2H), 4.40 (d, *J* = 15.6 Hz, 1H), 4.19 (q, *J* = 4.3 Hz, 2H), 4.00 (d, *J* = 11.1 Hz, 1H), 3.88 (p, *J* = 4.4 Hz, 3H), 3.70 (t, *J* = 4.5 Hz, 2H), 3.62 – 3.55 (m, 3H), 3.48 (dd, *J* = 6.7, 4.2 Hz, 3H), 3.17 (s, 2H), 2.49 (d, *J* = 12.4 Hz, 5H), 2.24 – 2.18 (m, 1H), 2.05 (ddd, *J* = 13.3, 9.0, 4.5 Hz, 1H), 1.85 (p, *J* = 6.7 Hz, 1H), 1.75 – 1.64 (m, 1H), 1.62 (dd, *J* = 6.6, 4.0 Hz, 1H), 1.55 – 1.44 (m, 8H), 1.43 – 1.28 (m, 4H), 1.05 (d, *J* = 6.6 Hz, 3H), 0.83 (d, *J* = 6.6 Hz, 3H).

^{13}C NMR: (101 MHz, MeOD) δ [ppm] = 174.6, 174.1, 170.9, 170.7, 165.4, 160.6, 158.0, 143.8, 139.2, 135.6, 133.2, 132.9, 132.5, 130.0, 129.2, 128.5, 128.3, 127.7, 124.8, 124.4, 124.4, 122.9, 118.4, 116.5, 115.5, 113.7, 102.5, 71.8, 71.7, 71.2, 71.0, 70.8, 70.3, 69.3, 62.8, 60.7, 60.1, 58.7, 57.2, 45.6, 44.5, 40.7, 39.5, 39.1, 37.7, 36.9, 34.8, 34.7, 30.8, 30.4, 30.2, 28.1, 27.3, 26.7, 23.9, 22.2, 19.7, 19.0, 15.9.

LC/MS: m/z calculated for $C_{62}H_{73}N_{11}O_{10}S$ $[M+2H]^{2+}$: 582.8, found: 582.6.

Purity (HPLC): 95%.

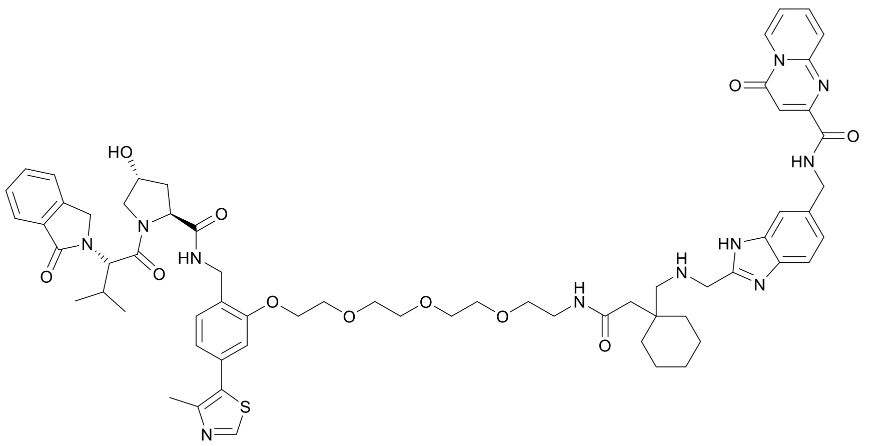

Compound **32**: **45** (17 mg, 0.023 mmol, 1.0 eq.) was dissolved in DMF (500 µL), DIPEA (20 µL, 0.12 mmol, 5.2 eq.), and **74** (8.4 mg, 0.023 mmol, 1.0 eq.) were added and stirred at RT for 3 h. The solvent was evaporated, and the resulting residue resolved in DCM/TFA (200+200 µL). The reaction was stirred at RT for 30 min, the solvent evaporated, and the mix was purified by RP flash chromatography. The residue (11 mg, 0.013 mmol, 1.0 eq.) was dissolved in DCE/HFIP (500+100 µL), and **72** (7 mg, 0.02 mmol, 1.5 eq.) and $Ti(O_iPr)_4$ (50 µL, 0.165 mmol, 12.7 eq.) were added. The reaction was incubated at RT for 16 h. Subsequently, MeOH (200 µL) and $NaBH_4$ (1 mg, 0.02 mol, 1.5 eq.) were added at 0 °C. The reaction was incubated at RT for 30 min and quenched by the addition of 0.1% TFA (10 µL). The organic solvents were evaporated, and the residue was purified by RP flash chromatography.

Yield: 4 mg (14% of th.).

^{1}H NMR: (400 MHz, MeO*D*) δ [ppm] = 9.06 (d, *J* = 7.1 Hz, 1H), 8.90 (s, 1H), 7.98 (t, *J* = 7.8 Hz, 1H), 7.79 (dd, *J* = 15.5, 8.3 Hz, 2H), 7.67 (s, 1H), 7.60 (dd, *J* = 8.1, 5.6 Hz, 2H), 7.55 (d, *J* = 7.6 Hz, 1H), 7.49 (t, *J* = 7.4 Hz, 1H), 7.39 (td, *J* = 9.3, 8.1, 4.3 Hz, 3H), 7.08 (s, 1H), 7.01 (d, *J* = 7.7 Hz, 2H), 4.74 (s, 2H), 4.62 (d, *J* = 18.0 Hz, 1H), 4.56 (d, *J* = 8.5 Hz, 1H), 4.52 (d, *J* = 5.6 Hz, 3H), 4.48 (d, *J* = 5.6 Hz, 2H), 4.41 (d, *J* = 15.6 Hz, 1H), 4.21 (q, *J* = 3.6 Hz, 2H), 3.99 (d, *J* = 11.0 Hz, 1H), 3.94 – 3.86 (m, 3H), 3.72 (dd, *J* = 5.6, 3.3 Hz, 2H), 3.64 (dd, *J* = 5.6, 3.5 Hz, 2H), 3.58 (dt, *J* = 9.5, 2.8 Hz, 3H), 3.51 (dd, *J* = 6.6, 3.3 Hz, 2H), 3.46 (dt, *J* = 10.4, 5.5 Hz, 3H), 3.18 (d, *J* = 4.9 Hz, 2H), 2.52 (d, *J* = 5.7 Hz, 2H), 2.48 (s, 3H), 2.25 – 2.18 (m, 1H), 2.07 (ddd, *J* = 13.5, 9.1, 4.6 Hz, 1H), 1.85 (p, *J* = 6.7 Hz, 1H), 1.75 – 1.67 (m, 1H), 1.61 (td, *J* = 6.7, 5.6, 3.4 Hz, 1H), 1.50 (dd, *J* = 17.8, 5.6 Hz, 8H), 1.43 – 1.32 (m, 3H), 1.05 (d, *J* = 6.5 Hz, 3H), 0.83 (d, *J* = 6.7 Hz, 3H).

^{13}C NMR: (101 MHz, MeOD) δ [ppm] = 174.6, 174.1, 170.9, 170.7, 165.4, 160.6, 158.0, 155.4, 143.8, 139.2, 135.6, 133.2, 132.9, 132.5, 130.0, 129.2, 128.5, 128.3, 127.8, 124.8, 124.4, 124.4, 122.8, 118.4, 116.5, 115.4, 113.7, 102.5, 71.8, 71.7, 71.6, 71.6, 71.2, 71.0, 70.8, 70.2, 69.4, 62.8, 60.7, 60.1, 57.2, 46.5, 45.6, 44.5, 40.7, 39.5, 39.1, 37.7, 37.0, 34.8, 30.8, 30.4, 30.2, 28.1, 27.3, 26.7, 22.2, 19.7, 19.0, 15.9.

LC/MS: m/z calculated for $C_{64}H_{77}N_{11}O_{11}S$ $[M+2H]^{2+}$: 604.8, found: 604.7.

Purity (HPLC): 95%.

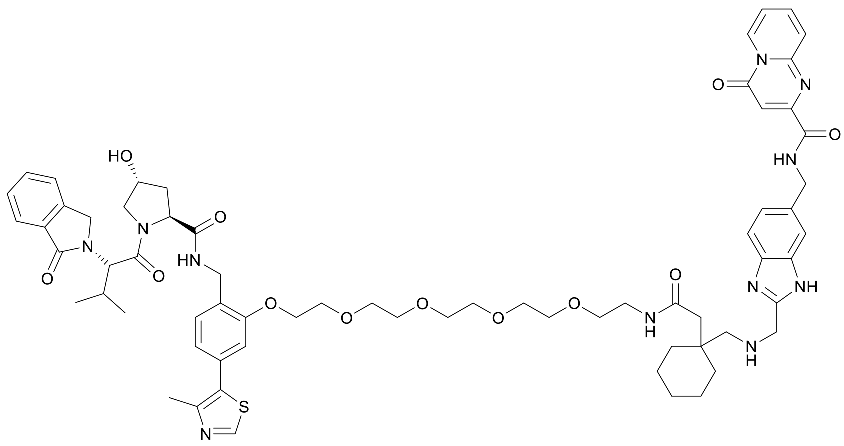

Compound **33**: **46** (18.2 mg, 0.023 mmol, 1.0 eq.) was dissolved in DMF (500 µL), DIPEA (20 µL, 0.06 mmol, 2.6 eq.), and **74** (8.4 mg, 0.023 mmol, 1.0 eq.) were added and stirred at RT for 3 h. The solvent was evaporated, and the resulting residue was resolved in DCM/TFA (200+200 µL). The reaction was stirred at RT for 30 min, the solvent was evaporated, and the mixture was purified by RP flash chromatography. The residue (18 mg, 0.02 mmol, 1.0 eq.) was dissolved in DCE/HFIP (500+100 µL), and **72** (7 mg, 0.02 mmol, 1.0 eq.) and $Ti(O_iPr)_4$ (50 µL, 0.165 mmol, 8.0 eq.) were added. The reaction was incubated at RT for 16 h. Subsequently, MeOH (200 µL) and $NaBH_4$ (1 mg, 0.02 mmol, 1.0 eq.) were added at 0 °C. The reaction was incubated at RT for 30 min and quenched by the addition of 0.1% TFA (10 µL). The organic solvents were evaporated, and the residue was purified by RP flash chromatography.

Yield: 3 mg (10% of th.).

^{1}H NMR: (400 MHz, MeO*D*) δ [ppm] = 9.07 (d, *J* = 7.2 Hz, 1H), 8.90 (s, 1H), 7.99 (t, *J* = 7.9 Hz, 1H), 7.79 (dd, *J* = 15.6, 8.2 Hz, 2H), 7.67 (s, 1H), 7.60 (dd, *J* = 7.9, 4.1 Hz, 2H), 7.55 (d, *J* = 7.6 Hz, 1H), 7.49 (t, *J* = 7.4 Hz, 1H), 7.45 – 7.35 (m, 3H), 7.08 (s, 1H), 7.01 (d, *J* = 7.4 Hz, 2H), 4.74 (s, 2H), 4.62 (d, *J* = 18.1 Hz, 1H), 4.56 (d, *J* = 8.5 Hz, 1H), 4.52 (d, *J* = 3.7 Hz, 3H), 4.48 (d, *J* = 5.8 Hz, 2H), 4.41 (d, *J* = 15.5 Hz, 1H), 4.21 (q, *J* = 3.8 Hz, 2H), 3.99 (d, *J* = 11.1 Hz, 1H), 3.89 (h, *J* = 4.4, 4.0 Hz, 3H), 3.75 – 3.70 (m, 2H), 3.65 (dd, *J* = 5.7, 3.2 Hz, 2H), 3.57 (ddd, *J* = 13.8, 9.0, 4.7 Hz, 7H), 3.53 – 3.44 (m, 5H), 3.19 (s, 2H), 2.53 (d, *J* = 9.5 Hz, 2H), 2.48 (s, 3H), 2.25 – 2.17 (m, 1H), 2.07 (ddd, *J* = 13.2, 8.9, 4.5 Hz, 1H), 1.85 (p, *J* = 6.7 Hz, 1H), 1.75 – 1.67 (m, 1H), 1.65 – 1.59 (m, 1H), 1.58 – 1.46 (m, 8H), 1.40 (d, *J* = 12.6 Hz, 3H), 1.05 (d, *J* = 6.4 Hz, 3H), 0.83 (d, *J* = 6.7 Hz, 3H).

^{13}C NMR: (101 MHz, MeOD) δ [ppm] = 174.6, 174.1, 170.9, 170.7, 165.4, 160.6, 158.1, 155.4, 143.8, 139.2, 135.6, 133.2, 132.9, 132.5, 130.0, 129.2, 128.5, 128.3, 127.8, 124.7, 124.4, 124.4, 122.8, 118.4, 116.5, 115.4, 113.7, 102.5, 71.8, 71.6, 71.5, 71.2, 71.0, 70.8, 70.3, 69.4, 62.8, 60.7, 60.1, 57.2, 45.7, 44.6, 40.7, 39.5, 39.1, 37.7, 37.0, 34.8, 30.8, 30.4, 30.2, 28.1, 27.3, 26.7, 22.2, 19.7, 19.0, 17.3, 15.9.

LC/MS: m/z calculated for $C_{66}H_{81}N_{11}O_{12}S$ $[M+2H]^{2+}$: 626.8, found: 626.6.

Purity (HPLC): 96%.

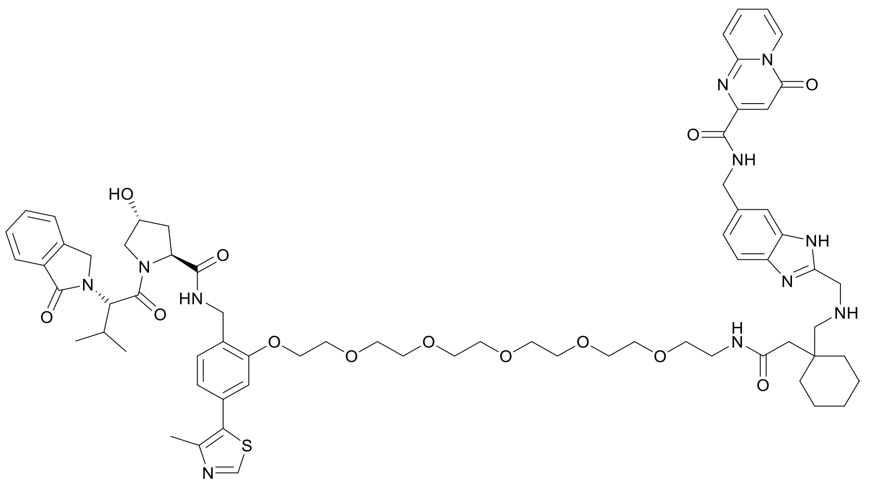

Compound **34**: **47** (25 mg, 0.040 mmol, 1.3 eq.) was dissolved in DMF (500 µL), DIPEA (20 µL, 0.06 mmol, 2.0 eq.), and **74** (11 mg, 0.030 mmol, 1.0 eq.) were added and stirred at RT for 3 h. The solvent was evaporated, and the resulting residue resolved in DCM/TFA (200+200 µL). The reaction was stirred at RT for 30 min. The solvent was evaporated and the mixture was purified by RP flash chromatography. The residue (14 mg, 0.02 mmol, 1.0 eq.) was dissolved in DCE/HFIP (500+100 µL), and **72** (7 mg, 0.02 mmol, 1.0 eq.) and $Ti(O_iPr)_4$ (50 µL, 0.165 mmol, 8.0 eq.) were added. The reaction was incubated at RT for 16 h. Subsequently, MeOH (200 µL) and $NaBH_4$ (1 mg, 0.02 mmol, 1.0 eq.) were added at 0 °C. The reaction was incubated at RT for 30 min and quenched by the addition of 0.1% TFA (10 µL). The organic solvents were evaporated, and the residue was purified by RP flash chromatography.

Yield: 3 mg (8% of th.).

^{1}H NMR: (400 MHz, MeO*D*) δ [ppm] = 9.07 (d, *J* = 7.2 Hz, 1H), 8.91 (s, 1H), 7.99 (t, *J* = 7.7 Hz, 1H), 7.80 (dd, *J* = 16.6, 8.2 Hz, 2H), 7.68 (s, 1H), 7.64 – 7.54 (m, 3H), 7.49 (t, *J* = 7.4 Hz, 1H), 7.41 (dt, *J* = 12.7, 8.4 Hz, 3H), 7.09 (s, 1H), 7.07 – 6.96 (m, 2H), 4.74 (s, 2H), 4.63 (d, *J* = 18.0 Hz, 1H), 4.56 (d, *J* = 8.6 Hz, 1H), 4.52 (d, *J* = 3.5 Hz, 3H), 4.48 (d, *J* = 6.3 Hz, 2H), 4.42 (d, *J* = 15.5 Hz, 1H), 4.21 (q, *J* = 4.0 Hz, 2H), 3.99 (d, *J* = 10.9 Hz, 1H), 3.89 (q, *J* = 3.9 Hz, 3H), 3.72 (t, *J* = 4.2 Hz, 2H), 3.65 (p, *J* = 4.4 Hz, 3H), 3.57 (td, *J* = 13.0, 11.5, 4.7 Hz, 13H), 3.46 (d, *J* = 5.3 Hz, 2H), 3.19 (d, *J* = 10.7 Hz, 2H), 2.53 (d, *J* = 9.9 Hz, 2H), 2.47 (d, *J* = 10.9 Hz, 4H), 2.25 – 2.18 (m, 1H), 2.07 (ddd, *J* = 13.3, 8.9, 4.5 Hz, 1H), 1.78 (dq, *J* = 47.3, 7.2 Hz, 1H), 1.54 (s, 9H), 1.40 (dd, *J* = 14.9, 6.9 Hz, 3H), 1.06 (t, *J* = 5.8 Hz, 3H), 0.84 (d, *J* = 6.7 Hz, 3H).

^{13}C NMR: (101 MHz, MeOD) δ [ppm] = 174.6, 174.1, 170.9, 170.7, 165.4, 160.6, 158.1, 143.8, 139.2, 135.6, 133.2, 132.9, 132.5, 130.1, 129.2, 128.5, 128.3, 127.8, 124.7, 124.4, 124.4, 122.8, 118.4, 113.8, 102.5, 71.8, 71.6, 71.5, 71.5, 71.5, 71.2, 70.8, 70.3, 69.4, 60.7, 60.1, 57.2, 45.7, 44.6, 40.7, 39.5, 39.1, 37.7, 37.0, 34.8, 30.2, 26.7, 23.9, 22.2, 19.7, 19.0, 15.9.

LC/MS: m/z calculated for $C_{68}H_{85}N_{11}O_{13}S$ $[M+3H]^{3+}$: 432.9, found: 432.9.

Purity (HPLC): 95%.

Synthesis of CRBN-based PROTACs

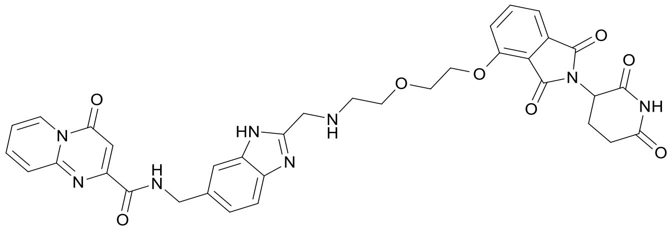

Compound **16**: **54** (10.8 mg, 0.03 mmol, 1.0 eq.) and **72** (13.8 mg, 0.04 mmol, 1.3 eq.) were dissolved in DCE/ACN (1+0.2 mL), and AcOH (6 µL, 0.1 mmol, 3.3 eq.) was added. Subsequently, $NaBH(OAc)_3$ (21 mg, 0.1 mmol, 3.3 eq.) was added, and the mixture was incubated at 30 °C for 5 h. The reaction was quenched by the addition of MeOH (1 mL). The solvent was evaporated. Purification by RP flash chromatography and lyophilization afforded the product as a colorless solid.

Yield: 7 mg (34% of th.).

^{1}H NMR: (600 MHz, MeO*D*) δ [ppm] = 9.03 (d, *J* = 7.0 Hz, 1H), 7.95 (ddd, *J* = 8.5, 6.7, 1.4 Hz, 1H), 7.79 (t, *J* = 8.2 Hz, 1H), 7.70 – 7.65 (m, 1H), 7.47 (s, 1H), 7.43 (d, *J* = 8.4 Hz, 1H), 7.40 – 7.30 (m, 3H), 7.24 (d, *J* = 8.3 Hz, 1H), 7.04 (s, 1H), 4.65 (s, 2H), 4.60 – 4.50 (m, 2H), 4.33 (t, *J* = 4.1 Hz, 2H), 4.03 – 3.89 (m, 4H), 3.50 – 3.37 (m, 2H), 2.60 (ddd, *J* = 18.5, 14.2, 5.1 Hz, 1H), 2.51 (d, *J* = 16.2 Hz, 1H), 2.42 – 2.32 (m, 1H), 1.80 – 1.72 (m, 1H), 1.33 – 1.22 (m, 1H).

^{13}C NMR: (151 MHz, MeOD) δ [ppm] = 174.4, 170.8, 168.3, 168.0, 165.4, 165.0, 160.7, 157.4, 155.4, 152.3, 139.2, 138.1, 134.8, 128.5, 127.8, 124.5, 120.3, 118.5, 116.8, 103.0, 102.5, 85.6, 70.3, 70.0, 67.6, 57.5, 56.2, 50.3, 45.2, 44.5, 32.1, 23.4.

LC/MS: m/z calculated for $C_{35}H_{32}N_8O_8$ $[M+H]^+$: 693.3, found: 693.1.

Purity (HPLC): 99%.

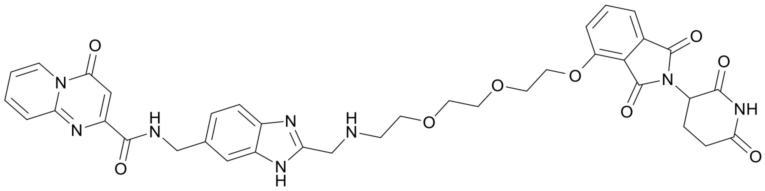

Compound **17**: **55** (8.0 mg, 0.02 mmol, 1.0 eq.) and **72** (7 mg, 0.02 mmol, 1.0 eq.) were dissolved in DCE/HFIP (500+100 µL) and $Ti(O_iPr)_4$ (50 µL, 0.165 mmol, 8.0 eq) and stirred at RT for 16 h. Subsequently, $Ti(O_iPr)_4$ (50 µL, 0.165 mmol, 8.0 eq.), MeOH (200 µL), and $NaBH_4$ (1 mg, 0.02 mmol, 1.0 eq.) were added, and the mixture was incubated at RT for 30 min. The reaction was quenched with TFA (10 µL), the solvent was evaporated, and the product was purified by RP flash chromatography. Lyophilization afforded the product as a colorless solid.

Yield: 7 mg (48% of th.).

^{1}H NMR: (600 MHz, MeO*D*) δ [ppm] = 9.00 (d, *J* = 7.0 Hz, 1H), 7.95 – 7.90 (m, 1H), 7.76 (d, *J* = 8.9 Hz, 1H), 7.54 (t, *J* = 7.9 Hz, 1H), 7.47 (s, 1H), 7.40 (d, *J* = 8.3 Hz, 1H), 7.34 (t, *J* = 6.9 Hz, 1H), 7.21 (t, *J* = 8.1 Hz, 3H), 7.03 (d, *J* = 5.7 Hz, 1H), 4.96 (d, *J* = 5.1 Hz, 1H), 4.64 (s, 2H), 4.25 (d, *J* = 21.4 Hz, 3H), 3.84 (s, 1H), 3.74 – 3.59 (m, 6H), 3.09 (s, 2H), 2.72 (td, *J* = 16.2, 14.0, 5.3 Hz, 1H), 2.64 – 2.52 (m, 2H), 2.00 (dd, *J* = 11.3, 6.2 Hz, 1H), 1.32 – 1.17 (m, 3H).

^{13}C NMR: (151 MHz, MeOD) δ [ppm] = 174.5, 171.7, 168.4, 167.5, 165.4, 160.6, 157.4, 155.4, 152.3, 139.2, 137.8, 134.8, 128.5, 127.8, 124.1, 120.4, 118.4, 117.8, 116.6, 102.5, 97.8, 71.9, 71.2, 70.3, 70.2, 68.7, 63.3, 50.4, 46.1, 44.5, 32.1, 23.6, 15.4.

LC/MS: m/z calculated for $C_{37}H_{36}N_8O_9$ $[M+H]^+$: 737.3, found: 737.1.

Purity (HPLC): 98%.

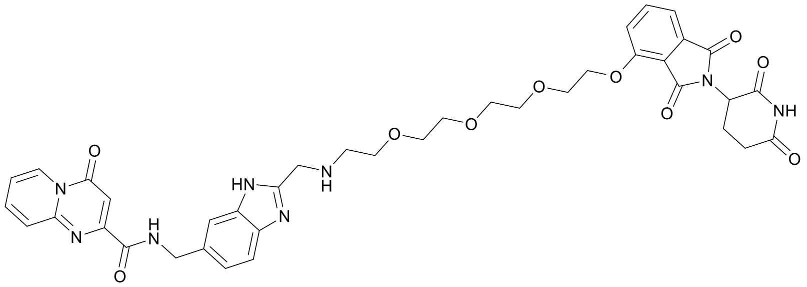

Compound **18**: **56** (8.8 mg, 0.02 mmol, 1.0 eq.) and **72** (7 mg, 0.02 mmol, 1.0 eq.) were dissolved in DCE/HFIP (500+100 µL) and $Ti(O_iPr)_4$ (50 µL, 0.165 mmol, 8.0 eq.) and stirred at RT for 16 h. Subsequently, $Ti(O_iPr)_4$ (50 µL, 0.165 mmol, 8.0 eq.), MeOH (200 µL), and $NaBH_4$ (1 mg, 0.02 mol, 1.0 eq.) were added, and the mixture was incubated at RT for 30 min. The reaction was quenched with TFA (10 µL), and the product was purified by RP flash chromatography. Lyophilization afforded the product as a colorless solid.

Yield: 4 mg (27% of th.).

^1H NMR: (600 MHz, MeO*D*) δ [ppm] = 9.01 (d, *J* = 7.0 Hz, 1H), 7.95 – 7.91 (m, 1H), 7.76 (d, *J* = 8.9 Hz, 1H), 7.56 – 7.48 (m, 2H), 7.43 (d, *J* = 8.4 Hz, 1H), 7.37 – 7.33 (m, 1H), 7.23 (d, *J* = 7.3 Hz, 2H), 7.16 (d, *J* = 8.5 Hz, 1H), 7.04 (s, 1H), 4.63 (d, *J* = 31.8 Hz, 3H), 4.29 (s, 1H), 4.17 (s, 2H), 3.78 (s, 1H), 3.70 – 3.55 (m, 7H), 3.08 (s, 2H), 2.80 – 2.69 (m, 2H), 2.67 – 2.51 (m, 3H), 2.05 – 1.93 (m, 2H), 1.24 (s, 1H).

^{13}C NMR: (151 MHz, MeOD) δ [ppm] = 184.3, 174.6, 171.6, 168.4, 167.5, 165.4, 160.6, 157.4, 155.5, 152.3, 139.2, 137.8, 134.8, 128.5, 127.8, 124.1, 120.4, 118.4, 117.9, 116.6, 102.5, 71.8, 71.5, 71.4, 71.0, 70.4, 70.2, 50.4, 44.5, 32.1, 23.7.

LC/MS: m/z calculated for $C_{39}H_{40}N_8O_{10}$ $[M+H]^+$: 781.3, found: 781.1.

Purity (HPLC): 95%.

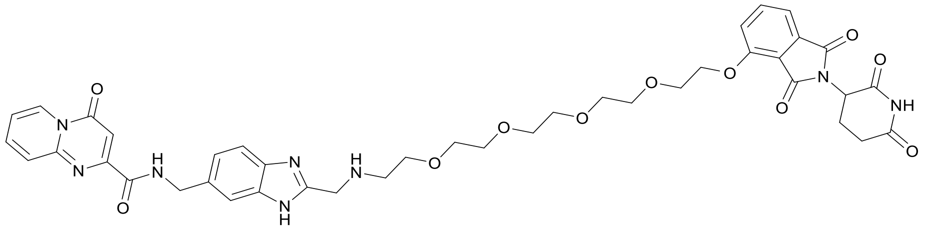

Compound **19**: **57** (9.8 mg, 0.02 mmol, 1.0 eq.) and **72** (7 mg, 0.02 mmol, 1.0 eq.) were dissolved in DCE/HFIP (500+100 µL) and $Ti(O_iPr)_4$ (50 µL, 0.165 mmol, 8.0 eq.) and stirred at RT for 16 h. Subsequently, $Ti(O_iPr)_4$ (50 µL, 0.165 mmol, 8.0 eq.), MeOH (200 µL), and $NaBH_4$ (1 mg, 0.02 mmol, 1.0 eq.) were added, and the mixture was incubated at RT for 30 min. The reaction was quenched with TFA (10 µL), and the product was purified by RP flash chromatography. Lyophilization afforded the product as a colorless solid.

Yield: 6 mg (36% of th.).

^{1}H NMR: (600 MHz, MeO*D*) δ [ppm] = 8.98 (d, *J* = 7.0 Hz, 1H), 7.93 – 7.89 (m, 1H), 7.74 (d, *J* = 8.9 Hz, 1H), 7.58 – 7.54 (m, 1H), 7.52 (s, 1H), 7.44 (d, *J* = 8.2 Hz, 1H), 7.33 (t, *J* = 6.9 Hz, 1H), 7.24 (dd, *J* = 14.3, 7.8 Hz, 2H), 7.19 (d, *J* = 8.5 Hz, 1H), 7.01 (s, 1H), 4.98 (dd, *J* = 12.7, 5.4 Hz, 1H), 4.64 (s, 2H), 4.31 (s, 2H), 4.19 – 4.13 (m, 2H), 3.76 – 3.71 (m, 2H), 3.65 (s, 2H), 3.64 – 3.61 (m, 2H), 3.55 (s, 8H), 3.10 (s, 2H), 2.75 (ddd, *J* = 18.7, 13.9, 5.2 Hz, 1H), 2.67 – 2.55 (m, 2H), 2.06 – 2.00 (m, 1H), 1.32 – 1.20 (m, 1H).

^{13}C NMR: (151 MHz, MeOD) δ [ppm] = 174.6, 171.6, 168.4, 167.3, 165.3, 160.6, 157.5, 155.4, 152.2, 139.2, 137.8, 134.9, 134.8, 128.5, 127.8, 124.1, 120.5, 118.4, 118.0, 116.6, 102.5, 71.9, 71.5, 71.3, 71.3, 71.0, 70.4, 70.2, 68.5, 50.4, 46.0, 44.6, 32.1, 23.6.

LC/MS: m/z calculated for $C_{41}H_{44}N_8O_{11}$ $[M+H]^+$: 825.3, found: 825.1.

Purity (HPLC): 98%.

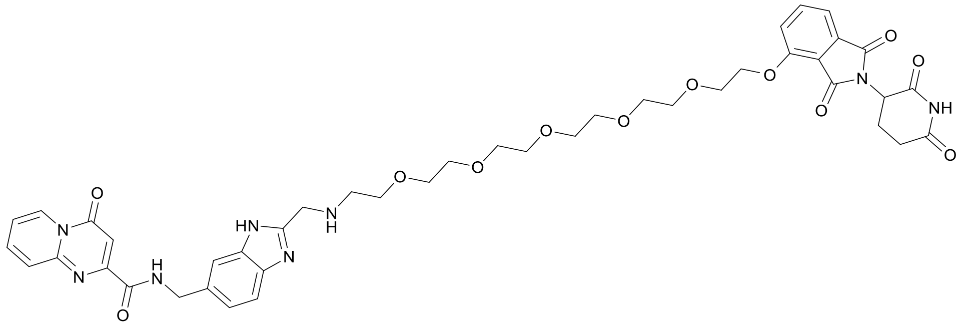

Compound **20**: **58** (10.6 mg, 0.02 mmol, 1.0 eq.) and **72** (7 mg, 0.02 mmol, 1.0 eq.) were dissolved in DCE/HFIP (500+100 µL) and $Ti(O_iPr)_4$ (50 µL, 0.165 mmol, 8.0 eq.) and stirred at RT for 16 h. Subsequently, $Ti(O_iPr)_4$ (50 µL, 0.165 mmol, 8.0 eq.), MeOH (200 µL), and $NaBH_4$ (1 mg, 0.02 mmol, 1.0 eq.) were added, and the mixture was incubated at RT for 30 min. The reaction was quenched with TFA (10 µL), the solvent was evaporated, and the product was purified by RP flash chromatography. Lyophilization afforded the product as a colorless solid.

Yield: 5 mg (29% of th.).

^{1}H NMR: (600 MHz, MeO*D*) δ [ppm] = 8.98 (d, *J* = 7.0 Hz, 1H), 7.95 – 7.89 (m, 1H), 7.74 (d, *J* = 8.6 Hz, 1H), 7.61 – 7.56 (m, 1H), 7.54 (d, *J* = 12.8 Hz, 1H), 7.44 (dd, *J* = 14.2, 5.6 Hz, 1H), 7.33 (t, *J* = 6.9 Hz, 1H), 7.23 (td, *J* = 21.2, 20.7, 7.3 Hz, 3H), 7.01 (s, 1H),

4.97 (dd, J = 12.7, 5.4 Hz, 1H), 4.65 (s, 2H), 4.36 – 4.24 (m, 2H), 4.15 (d, J = 37.7 Hz, 2H), 3.79 – 3.70 (m, 2H), 3.68 – 3.61 (m, 3H), 3.59 – 3.43 (m, 11H), 3.16 – 3.03 (m, 2H), 2.78 – 2.69 (m, 1H), 2.68 – 2.54 (m, 2H), 2.04 – 1.97 (m, 1H), 1.32 – 1.20 (m, 1H).

^{13}C NMR: (151 MHz, MeOD) δ [ppm] = 174.6, 171.5, 168.4, 167.3, 165.3, 160.6, 157.5, 155.4, 152.2, 139.2, 137.9, 134.9, 134.8, 128.5, 127.8, 124.1, 120.5, 118.4, 118.0, 116.6, 102.5, 71.9, 71.5, 71.4, 71.3, 71.2, 71.0, 70.4, 70.3, 68.6, 50.4, 46.1, 44.6, 32.1, 23.6, 19.2.

LC/MS: m/z calculated for $C_{43}H_{48}N_8O_{12}$ $[M+2H]^{2+}$: 435.2, found: 435.1.

Purity (HPLC): 94%.

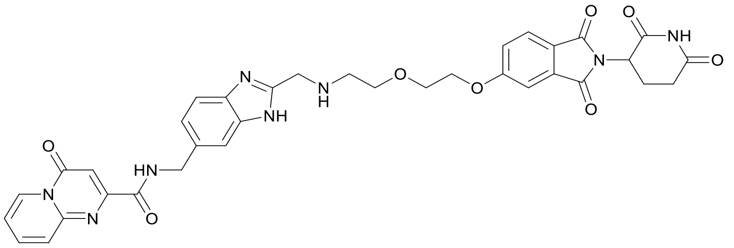

Compound **21**: **60** (10.8 mg, 0.03 mmol, 1.0 eq.) and **72** (13.8 mg, 0.04 mmol, 1.3 eq.) were dissolved in DCE/ACN (1+0.2 mL), AcOH (6 µL, 0.1 mmol, 3.3 eq.) and $NaBH(OAc)_3$ (21 mg, 0.1 mmol, 3.3 eq.) were added and the reaction was incubated at 30 °C for 5 h. The reaction was quenched by the addition of MeOH (1 mL), the solvent was evaporated, and the product was purified by RP flash chromatography. Lyophilization afforded the product as a yellowish solid.

Yield: 9 mg (43% of th.).

^{1}H NMR: (600 MHz, MeO*D*) δ [ppm] = 8.98 (d, J = 7.0 Hz, 1H), 7.94 – 7.89 (m, 1H), 7.73 (d, J = 8.9 Hz, 1H), 7.62 (d, J = 8.3 Hz, 1H), 7.52 (s, 1H), 7.46 (d, J = 8.3 Hz, 1H), 7.33 (td, J = 7.0, 1.2 Hz, 1H), 7.29 – 7.22 (m, 2H), 7.16 (d, J = 7.4 Hz, 1H), 7.02 – 6.97 (m, 1H), 5.02 (dd, J = 12.8, 5.5 Hz, 1H), 4.63 (s, 2H), 4.48 (d, J = 16.4 Hz, 2H), 4.23 (s, 2H), 3.87 (dt, J = 17.0, 4.6 Hz, 3H), 3.29 (s, 9H), 2.79 (ddd, J = 17.3, 13.9, 5.2 Hz, 1H), 2.70 – 2.58 (m, 2H), 2.04 (dtd, J = 12.9, 6.1, 5.4, 3.1 Hz, 1H).

^{13}C NMR: (151 MHz, MeOD) δ [ppm] = 174.6, 171.5, 168.5, 168.4, 165.5, 165.3, 160.6, 155.3, 152.2, 139.3, 135.6, 135.2, 128.5, 127.7, 126.3, 125.0, 124.6, 124.2, 121.3, 118.5, 110.0, 102.5, 70.6, 69.5, 67.4, 57.5, 50.6, 49.9, 45.1, 44.6, 32.2, 23.7, 17.2.

LC/MS: m/z calculated for $C_{35}H_{32}N_8O_8$ $[M+H]^{+}$: 693.2, found: 693.1.

Purity (HPLC): 99%.

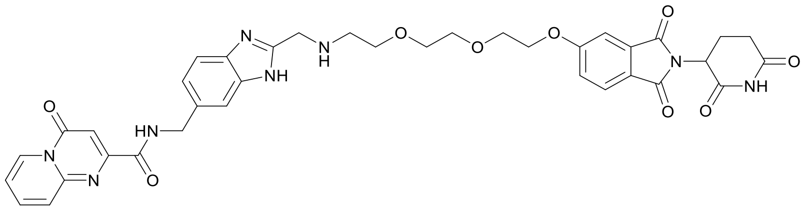

Compound **22**: **61** (8.0 mg, 0.02 mmol, 1.0 eq.) and **72** (7 mg, 0.02 mmol, 1.0 eq.) were dissolved in DCE/HFIP (500+100 µL) and $Ti(O_iPr)_4$ (50 µL, 0.165 mmol, 8.0 eq.) and the reaction was stirred at RT for 16 h. Subsequently, $Ti(O_iPr)_4$ (50 µL, 0.165 mmol, 8.0 eq.), MeOH (200 µL), and $NaBH_4$ (1 mg, 0.02 mmol, 1.0 eq.) were added, and the mixture was incubated at RT for 30 min. The reaction was quenched with TFA (10 µL), the solvent was evaporated, and the product was purified by RP flash chromatography. Lyophilization afforded the product as a colorless solid.

Yield: 13 mg (88% of th.).

^{1}H NMR: (600 MHz, MeO*D*) δ [ppm] = 9.00 (d, *J* = 7.0 Hz, 1H), 7.94 – 7.90 (m, 1H), 7.74 (d, *J* = 8.8 Hz, 1H), 7.55 – 7.50 (m, 2H), 7.43 (d, *J* = 8.0 Hz, 1H), 7.34 (t, *J* = 6.9 Hz, 1H), 7.23 (d, *J* = 8.1 Hz, 1H), 7.18 (s, 1H), 7.06 – 7.01 (m, 2H), 5.02 (dd, *J* = 12.9, 5.5 Hz, 1H), 4.63 (d, *J* = 30.9 Hz, 3H), 4.14 (d, *J* = 19.6 Hz, 3H), 3.79 (s, 2H), 3.67 – 3.59 (m, 5H), 2.99 (s, 2H), 2.80 (ddd, *J* = 19.3, 14.0, 5.2 Hz, 2H), 2.65 (dtd, *J* = 26.7, 13.3, 3.5 Hz, 3H), 2.08 – 2.03 (m, 1H), 1.30 – 1.23 (m, 2H).

^{13}C NMR: (151 MHz, MeOD) δ [ppm] = 174.6, 171.5, 168.5, 168.4, 165.5, 165.3, 160.6, 155.4, 152.2, 139.2, 135.5, 134.6, 128.5, 127.8, 126.1, 124.8, 124.0, 121.3, 118.4, 109.9, 102.5, 71.7, 71.3, 70.5, 69.6, 50.5, 44.6, 32.2, 23.7.

LC/MS: m/z calculated for $C_{37}H_{36}N_8O_9$ $[M+H]^+$: 737.3, found: 737.2.

Purity (HPLC): 99%.

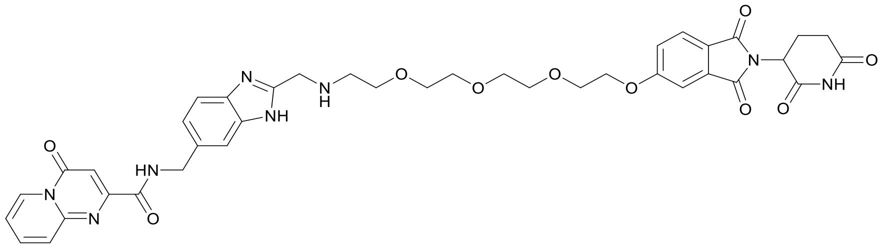

Compound **23**: **62** (8.8 mg, 0.02 mmol, 1.0 eq.) and **72** (7 mg, 0.02 mmol, 1.0 eq.) were dissolved in DCE/HFIP (500+100 µL) and $Ti(O_iPr)_4$ (50 µL, 0.165 mmol, 8.0 eq.) and stirred at RT for 16 h. Subsequently, $Ti(O_iPr)_4$ (50 µL, 0.165 mmol, 8.0 eq.), MeOH (200 µL), and $NaBH_4$ (1 mg, 0.02 mmol, 1.0 eq.) were added and incubated at RT for 30 min. The reaction was quenched with TFA (10 µL), the solvent was evaporated, and the product was purified by RP flash chromatography. Lyophilization afforded the product as a colorless solid.

Yield: 6 mg (38% of th.).

^{1}H NMR: (600 MHz, MeO*D*) δ [ppm] = 8.99 (d, *J* = 7.0 Hz, 1H), 7.94 – 7.90 (m, 1H), 7.74 (d, *J* = 8.9 Hz, 1H), 7.57 – 7.51 (m, 2H), 7.45 (d, *J* = 8.1 Hz, 1H), 7.34 (t, *J* = 6.4 Hz, 1H), 7.24 (d, *J* = 8.3 Hz, 1H), 7.14 (s, 1H), 7.06 – 7.00 (m, 2H), 5.02 (dd, *J* = 12.8, 5.5 Hz, 1H), 4.62 (d, *J* = 30.6 Hz, 6H), 4.17 (s, 1H), 4.04 (d, *J* = 4.2 Hz, 1H), 3.72 – 3.66 (m, 2H), 3.63 – 3.53 (m, 8H), 3.29 (s, 1H), 2.97 (s, 1H), 2.79 (ddd, *J* = 18.0, 14.0, 5.3 Hz, 2H), 2.72 – 2.59 (m, 3H), 2.08 – 2.03 (m, 1H), 1.26 – 1.22 (m, 1H).

^{13}C NMR: (151 MHz, MeOD) δ [ppm] = 174.6, 171.5, 168.5, 168.5, 165.5, 165.3, 160.6, 155.4, 152.2, 139.2, 135.5, 128.5, 127.8, 126.1, 124.8, 124.0, 121.2, 118.4, 110.0, 102.5, 72.8, 71.7, 71.5, 71.5, 71.1, 70.5, 69.6, 50.5, 44.6, 32.2, 23.7, 19.1, 0.8.

LC/MS: m/z calculated for $C_{39}H_{40}N_8O_{10}$ $[M+H]^+$: 781.3, found: 781.2.

Purity (HPLC): 99%.

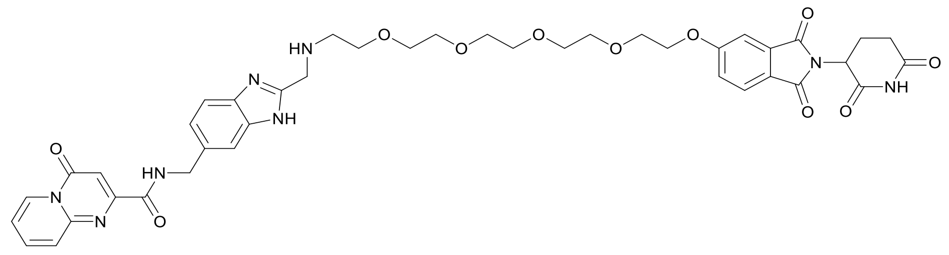

Compound **24**: **63** (9.8 mg, 0.02 mmol, 1.0 eq.) and **72** (7 mg, 0.02 mmol, 1.0 eq.) were dissolved in DCE/HFIP (500+100 µL) and $Ti(O_iPr)_4$ (50 µL, 0.165 mmol, 8.0 eq.) and stirred at RT for 16 h. Subsequently, $Ti(O_iPr)_4$ (50 µL, 0.165 mmol, 8.0 eq.), MeOH (200 µL), and $NaBH_4$ (1 mg, 0.02 mmol, 1.0 eq.) were added and incubated at RT for 30 min. The reaction was quenched with TFA (10 µL), the solvent was evaporated, and the product was purified by RP flash chromatography. Lyophilization afforded the product as a colorless solid.

Yield: 4 mg (24% of th.).

^1H NMR: (600 MHz, MeO*D*) δ [ppm] = 9.06 (d, *J* = 7.0 Hz, 1H), 8.01 – 7.97 (m, 1H), 7.81 (t, *J* = 7.8 Hz, 1H), 7.67 (d, *J* = 8.3 Hz, 1H), 7.60 (s, 1H), 7.53 (d, *J* = 8.3 Hz, 1H), 7.42 – 7.39 (m, 1H), 7.31 (d, *J* = 8.2 Hz, 1H), 7.25 (s, 1H), 7.18 – 7.14 (m, 1H), 7.09 (s, 1H), 5.09 (dd, *J* = 12.9, 5.4 Hz, 1H), 4.73 (s, 2H), 4.19 (s, 1H), 4.15 – 4.13 (m, 1H), 3.78 – 3.71 (m, 3H), 3.70 – 3.58 (m, 14H), 2.97 (s, 2H), 2.91 – 2.82 (m, 2H), 2.79 – 2.67 (m, 3H), 2.16 – 2.10 (m, 1H), 1.35 – 1.30 (m, 1H).

^{13}C NMR: (151 MHz, MeOD) δ [ppm] = 174.6, 171.5, 168.5, 168.5, 165.6, 165.3, 160.6, 155.4, 152.2, 139.2, 135.5, 134.5, 128.5, 127.8, 126.2, 124.8, 123.9, 121.3, 118.4, 110.0, 102.5, 71.7, 71.5, 71.5, 71.5, 71.1, 70.4, 69.9, 69.6, 67.1, 50.5, 46.9, 44.6, 32.2, 23.7, 19.2.

LC/MS: m/z calculated for $C_{41}H_{44}N_8O_{11}$ $[M+H]^+$: 825.3, found: 825.1.

Purity (HPLC): 98%.

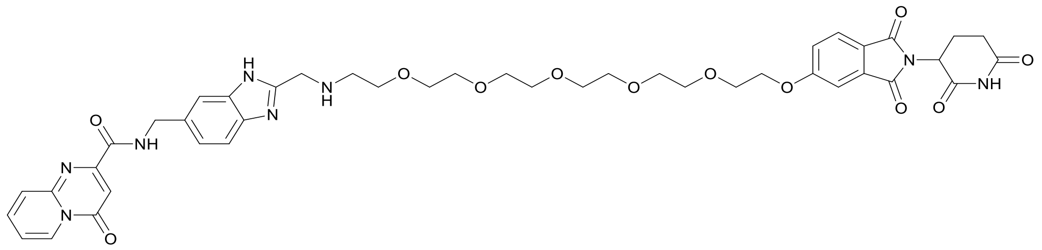

Compound **25**: **64** (10.6 mg, 0.02 mmol, 1.0 eq.) and **72** (7 mg, 0.02 mmol, 1.0 eq.) were dissolved in DCE/HFIP (500+ 100 µL) and $Ti(O_iPr)_4$ (50 µL, 0.165 mmol, 8.0 eq.) and stirred at RT for 16 h. Subsequently, $Ti(O_iPr)_4$ (50 µL, 0.165 mmol, 8.0 eq.), MeOH (200 µL), and $NaBH_4$ (1 mg, 0.02 mol, 1.0 eq.) were added and incubated at RT for 30 min. The reaction was quenched

with TFA (10 µL), the solvent was evaporated, and the product was purified by RP flash chromatography. Lyophilization afforded the product as a colorless solid.

Yield: 2 mg (12% of th.).

^{1}H NMR: (600 MHz, MeO*D*) δ [ppm] = 9.07 (d, *J* = 7.1 Hz, 1H), 8.02 – 7.98 (m, 1H), 7.82 (d, *J* = 9.4 Hz, 1H), 7.70 (d, *J* = 8.3 Hz, 1H), 7.62 (s, 1H), 7.55 (d, *J* = 8.3 Hz, 1H), 7.43 – 7.40 (m, 1H), 7.32 (d, *J* = 8.2 Hz, 1H), 7.29 (s, 1H), 7.20 (d, *J* = 7.3 Hz, 1H), 7.10 (s, 1H), 5.09 (dd, *J* = 12.8, 5.4 Hz, 1H), 4.74 (s, 1H), 4.67 (s, 1H), 4.36 – 4.32 (m, 1H), 4.24 (s, 1H), 4.18 (s, 1H), 3.95 – 3.90 (m, 1H), 3.80 – 3.78 (m, 1H), 3.74 – 3.56 (m, 14H), 3.37 (s, 3H), 3.25 – 3.20 (m, 1H), 3.03 (s, 1H), 2.92 – 2.83 (m, 2H), 2.80 – 2.67 (m, 3H), 2.16 – 2.11 (m, 1H), 2.06 (s, 1H), 1.34 (d, *J* = 6.6 Hz, 1H).

^{13}C NMR: (151 MHz, MeOD) δ [ppm] = 174.6, 171.5, 168.5, 168.5, 165.6, 160.6, 152.2, 139.2, 135.5, 128.5, 127.8, 126.2, 124.8, 121.4, 118.4, 110.1, 102.5, 71.7, 71.5, 71.5, 71.4, 71.4, 70.5, 69.6, 67.0, 50.6, 44.6, 32.2, 23.7, 19.2, 0.8.

LC/MS: m/z calculated for $C_{43}H_{48}N_8O_{12}$ $[M+2H]^{2+}$: 435.2, found: 435.1.

Purity (HPLC): 96%.

Synthesis of 3rd generation PROTACs

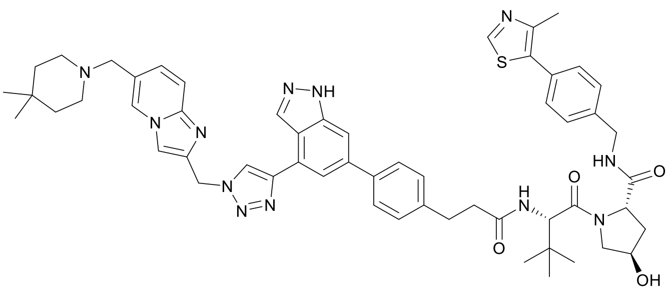

Compound **35**: **65** (6.7 mg, 0.011 mmol, 1.1 eq.), STM3006 (5 mg, 0.010 mmol, 1.0 eq.), potassium phosphate (4.3 mg, 0.02 mmol, 2.0 eq.), X-Phos (1 mg, 0.002 mmol, 0.2 eq.), and tris(dibenzylidenacetone)dipalladium (1 mg, 0.001 mmol, 0.1 eq.) were dissolved under argon atmosphere in 56 µL degassed water/ dioxane (1:4). The reaction was stirred at 110 °C for 1.5 h. The mixture was diluted with water, the solvents were evaporated, and the residue was purified with RP flash chromatography.

Yield: 4 mg (40% of th.).

^{1}H NMR: (600 MHz, DMSO-*d*$_6$) δ [ppm] = 8.98 (s, 2H), 8.59 (q, *J* = 6.2, 4.8 Hz, 2H), 8.44 (s, 1H), 8.03 – 7.97 (m, 2H), 7.90 (d, *J* = 1.4 Hz, 1H), 7.73 – 7.66 (m, 3H), 7.46 (d, *J* = 9.3 Hz, 1H), 7.42 (d, *J* = 8.1 Hz, 2H), 7.39 – 7.36 (m, 2H), 7.34 (d, *J* = 8.0 Hz, 2H), 7.20 (dd, *J* = 9.3, 1.7 Hz, 1H), 5.79 (d, *J* = 6.5 Hz, 2H), 5.18 (s, 2H), 4.56 (dd, *J* = 16.9, 9.4 Hz, 2H), 4.48 – 4.40 (m, 3H), 4.36 (s, 1H), 4.21 (dd, *J* = 15.9, 5.5 Hz, 2H), 3.69 – 3.65 (m, 2H), 3.43 (s, 2H), 2.90 – 2.83 (m, 2H), 2.67 – 2.60 (m, 2H), 2.44 (d, *J* = 3.5 Hz, 3H), 2.35 – 2.32 (m, 3H), 2.07 – 2.00 (m, 2H), 1.90 (ddd, *J* = 12.9, 8.7, 4.6 Hz, 2H), 1.30 (t, *J* = 5.5 Hz, 3H), 0.90 (d, *J* = 4.3 Hz, 7H), 0.87 (d, *J* = 4.7 Hz, 5H).

^{13}C NMR: (151 MHz, DMSO) δ [ppm] = 172.0, 171.3, 169.7, 163.7, 147.7, 145.8, 144.2, 141.4, 140.9, 140.5, 139.6, 138.6, 137.9, 131.2, 129.7, 129.1, 128.9, 128.7, 127.4, 127.3, 127.1, 125.6, 123.6, 123.2, 123.1, 118.7, 117.1, 116.2, 115.0, 111.6, 106.9, 68.9, 59.1, 58.8, 56.5, 56.4, 49.3, 48.0, 41.7, 40.1, 38.3, 38.0, 36.5, 35.3, 31.0, 28.3, 28.1, 26.4, 16.0.

LC/MS: m/z calculated for $C_{56}H_{64}N_{12}O_4S$ $[M+2H]^{2+}$: 501.2, found: 501.2.

Purity (HPLC): 97%.

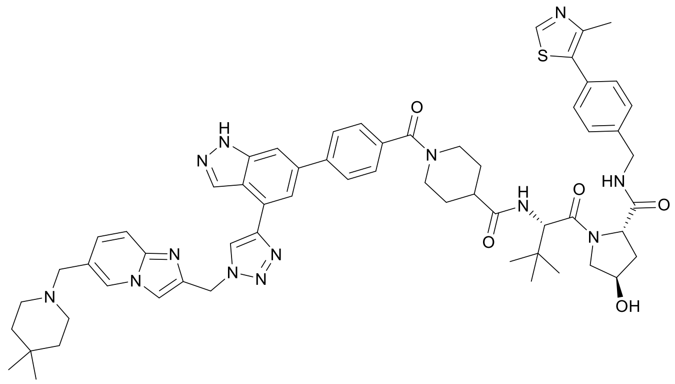

Compound **36**: **66** (9.5 mg, 0.013 mmol, 1.0 eq.), STM3006 (6.7 mg, 0.013 mmol, 1.0 eq.), potassium phosphate (5.5 mg, 0.026 mmol, 2.0 eq.), X-Phos (1.2 mg, 0.003 mmol, 0.2 eq.), and tris(dibenzylidenacetone)dipalladium (2.3 mg, 0.003 mmol, 0.2 eq.) were dissolved under argon atmosphere in 112 µL degassed water/ dioxane (1:4). The reaction was stirred at 110 °C

for 2 h. The mixture was diluted with water, the solvents were evaporated, and the residue was purified with RP flash chromatography.

Yield: 4.5 mg (32% of th.).

^{1}H NMR: (400 MHz, DMSO-d_6) δ [ppm] = 8.98 (d, *J* = 3.6 Hz, 2H), 8.63 (s, 1H), 8.57 (t, *J* = 6.1 Hz, 1H), 8.44 (s, 1H), 7.99 (s, 1H), 7.95 (d, *J* = 1.4 Hz, 1H), 7.91 (d, *J* = 9.3 Hz, 1H), 7.88 (d, *J* = 1.8 Hz, 1H), 7.86 (d, *J* = 1.9 Hz, 1H), 7.75 (s, 1H), 7.51 (s, 1H), 7.49 (s, 1H), 7.48 – 7.42 (m, 2H), 7.41 (s, 1H), 7.38 (d, *J* = 8.4 Hz, 2H), 7.20 (dd, *J* = 9.3, 1.7 Hz, 1H), 5.80 (s, 2H), 5.14 (s, 1H), 4.54 (d, *J* = 9.3 Hz, 1H), 4.46 – 4.33 (m, 4H), 4.22 (dd, *J* = 15.9, 5.5 Hz, 1H), 3.71 – 3.59 (m, 4H), 2.71 (s, 2H), 2.44 (s, 3H), 2.35 (d, *J* = 4.0 Hz, 5H), 2.07 (s, 2H), 2.02 (d, *J* = 8.7 Hz, 1H), 1.94 – 1.86 (m, 1H), 1.55 (d, *J* = 12.5 Hz, 3H), 1.30 (t, *J* = 5.6 Hz, 4H), 0.94 (s, 10H), 0.87 (s, 6H).

^{13}C NMR: (101 MHz, DMSO) δ [ppm] = 173.7, 172.0, 169.7, 169.6, 168.8, 151.5, 151.5, 147.7, 145.8, 144.1, 141.3, 141.2, 140.5, 139.5, 137.8, 135.3, 131.2, 129.6, 128.6, 127.4, 127.3, 127.3, 125.6, 123.8, 123.2, 116.1, 111.6, 68.9, 59.1, 56.2, 50.0, 49.2, 41.7, 38.3, 37.9, 35.4, 28.2, 26.4, 19.1, 16.0, 1.2.

LC/MS: m/z calculated for $C_{60}H_{69}N_{13}O_5S$ $[M+2H]^{2+}$: 542.8, found: 542.7.

Purity (HPLC): 97%.

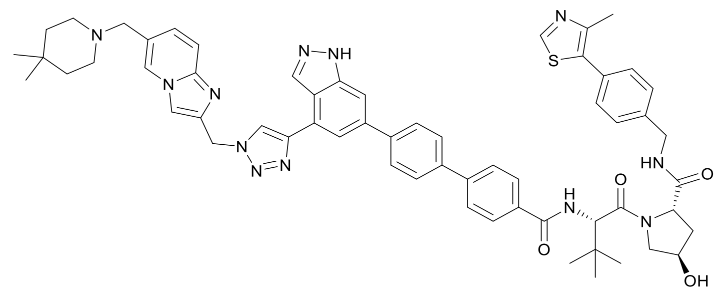

Compound **38**: **67** (7 mg, 0.011 mmol, 1.1 eq.), STM3006 (5 mg, 0.01 mmol, 1.0 eq.), potassium phosphate (4.3 mg, 0.02 mmol, 2.0 eq.), X-Phos (1 mg, 0.002 mmol, 0.2 eq.) and tris(dibenzylidenacetone)dipalladium (1 mg, 0.001 mmol, 0.1 eq.) were dissolved under argon atmosphere in 56 µL degassed water/ dioxane (1:4). The reaction was stirred at 110 °C for 1.5 h. The mixture was diluted with water, the solvents were evaporated, and the residue was purified with RP flash chromatography.

Yield: 3.7 mg (35% of th.).

^{1}H NMR: (400 MHz, DMSO-d_6) δ [ppm] = 9.00 (d, *J* = 6.7 Hz, 2H), 8.64 – 8.58 (m, 2H), 8.44 (s, 1H), 8.16 – 8.06 (m, 2H), 8.01 (s, 1H), 7.99 (s, 3H), 7.95 (s, 1H), 7.93 (s, 1H), 7.89 (s, 1H), 7.87 – 7.85 (m, 2H), 7.84 (s, 1H), 7.79 (s, 1H), 7.47 (d, *J* = 9.2 Hz, 1H), 7.42 (d, *J* = 2.9 Hz, 3H), 7.23 – 7.19 (m, 1H), 5.81 (s, 2H), 5.17 (d, *J* = 3.6 Hz, 1H), 4.81 (d, *J* = 9.1 Hz, 1H), 4.47 (q, *J* = 7.1, 6.1 Hz, 2H), 4.40 (dd, *J* = 8.3, 4.9 Hz, 2H), 4.25 (dd, *J* = 15.8, 5.6 Hz, 2H), 3.75 (d, *J* = 3.2 Hz, 2H), 3.45 (s, 3H), 2.45 (s, 2H), 2.07 (s, 3H), 1.31 (t, *J* = 5.6 Hz, 4H), 1.08 – 1.02 (m, 10H), 0.88 (s, 7H).

^{13}C NMR: (101 MHz, DMSO) δ [ppm] = 171.9, 169.5, 166.2, 158.4, 151.5, 147.8, 145.8, 142.3, 140.5, 139.9, 139.5, 133.0, 131.2, 129.7, 128.7, 128.5, 127.9, 127.5, 127.4, 126.3, 125.6, 123.7, 123.1, 116.1, 114.7, 68.9, 62.0, 59.1, 57.4, 49.2, 40.2, 38.2, 35.6, 28.2, 26.6, 25.5, 16.0, 1.2.

LC/MS: m/z calculated for $C_{60}H_{64}N_{12}O_4S$ $[M+2H]^{2+}$: 525.2, found: 525.2.

Purity (HPLC): 95%.

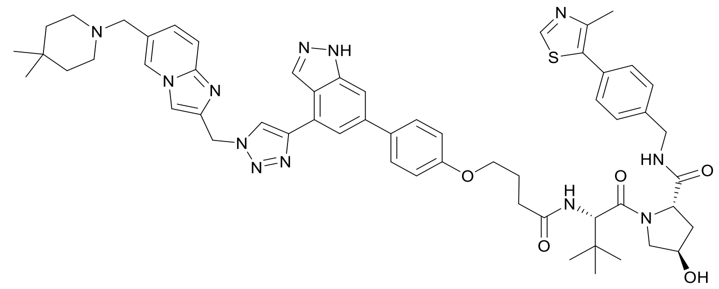

Compound **37**: **68** (15.8 mg, 0.022 mmol, 1.1 eq.), STM3006 (10 mg, 0.02 mmol, 1.0 eq.), potassium phosphate (8.4 mg, 0.04 mmol, 2.0 eq.), X-Phos (1 mg, 0.002 mmol, 0.1 eq.), and tris(dibenzylidenacetone)dipalladium (2 mg, 0.002 mmol, 0.1 eq.) were dissolved under argon atmosphere in 56 µL degassed water/ dioxane (1:4). The reaction was stirred at 110 °C for 1.5 h. The mixture was diluted with water, the solvents were evaporated, and the residue was purified with RP flash chromatography.

Yield: 5.6 mg (27% of th.).

^{1}H NMR: (400 MHz, DMSO-d_6) δ [ppm] = 8.97 (d, *J* = 4.0 Hz, 1H), 8.57 (d, *J* = 5.0 Hz, 2H), 8.43 (s, 2H), 7.99 (d, *J* = 9.5 Hz, 2H), 7.87 (s, 1H), 7.72 (d, *J* = 8.4 Hz, 2H), 7.64 (s, 1H), 7.41 (dp, *J* = 16.7, 8.9 Hz, 5H), 7.20 (d, *J* = 9.5 Hz, 1H), 7.04 (d, *J* = 8.4 Hz, 2H), 5.79 (s, 2H), 4.57 (d, *J* = 9.3 Hz, 1H), 4.47 – 4.33 (m, 3H), 4.21 (dd, *J* = 15.9, 5.5 Hz, 1H), 4.02 (dt, *J* = 7.0, 4.2 Hz, 2H), 3.68 (d, *J* = 3.7 Hz, 1H), 2.44 (s, 3H), 2.34 (d, *J* = 7.0 Hz, 4H), 2.06 – 1.87 (m, 4H), 1.56 (td, *J* = 11.8, 10.1, 5.9 Hz, 3H), 1.30 (t, *J* = 5.8 Hz, 6H), 1.04 (d, *J* = 6.1 Hz, 2H), 0.94 (d, *J* = 6.0 Hz, 10H), 0.87 (s, 5H).

^{13}C NMR: (101 MHz, DMSO) δ [ppm] = 68.9, 67.1, 62.0, 59.1, 57.5, 56.5, 49.2, 41.6, 38.3, 38.0, 35.2, 32.3, 31.3, 28.2, 26.4, 26.3, 25.5, 23.1, 19.2, 15.9, 13.5.

LC/MS: m/z calculated for $C_{57}H_{66}N_{12}O_5S$ [M+H]$^+$: 1031.5, found: 1031.3.

Purity (HPLC): 99%.

Synthesis of 4th generation PROTACs

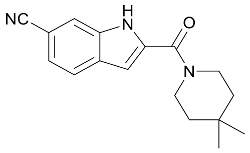

Compound **75**: Methyl 6-cyano-1*H*-indole-2-carboxylate (100 mg, 0.5 mmol, 1.0 eq.) was suspended in 1.5 mL methanol and 0.75 mL 1 M NaOH and stirred at RT for 4 h. The reaction mixture was acidified with 2 M HCl. Ethylacetat was added, and the solution was washed with water. The organic solvents were dried over Na_2SO_4 and evaporated. To this residue, TBTU (161 mg, 0.5 mmol, 1.0 eq.), DIPEA (219 µL, 1.250 mmol, 2.5 eq.), and 4,4-dimethylpiperidine hydrochloride (97 mg, 0.650 mmol, 1.3 eq.) were added in ethyl acetate (5 mL) and stirred at RT for 16 h. The organic solvents were evaporated, and the crude product was purified by RP flash chromatography.

Yield: 117 mg (83% of th.).

^{1}H NMR: (300 MHz, C*D*Cl$_3$) δ [ppm] = 11.14 (s, 1H), 7.78 – 7.76 (m, 1H), 7.70 – 7.66 (m, 1H), 7.30 (dd, *J* = 8.3, 1.4 Hz, 1H), 6.81 – 6.79 (m, 1H), 3.91 (s, 4H), 1.56 – 1.49 (m, 4H), 1.07 (s, 6H).

^{13}C NMR: (75 MHz, *C*DCl$_3$) δ [ppm] = 162.1, 134.9, 133.2, 130.3, 122.9, 122.6, 120.5, 117.4, 106.4, 105.0, 38.8, 29.5, 27.9.

LC/MS: m/z calculated for $C_{17}H_{19}N_3O$ [M+H]$^+$: 282.2, found: 282.1.

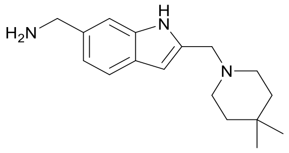

Compound **76**: **75** (195 mg, 0.695 mmol, 1.0 eq.) was dissolved in 1.5 mL anhydrous THF, and 1 M LAH (2.2 mL, 2.157 mmol, 3.1 eq.) in THF was added at 0 °C. The reaction was stirred at RT for 16 h before 1.5 mL of water and 1.5 mL of 1 M NaOH were added at 0 °C. The suspension was stirred for 10 min. The precipitate was removed by filtration and washed with THF. The organic solvents were evaporated, and the crude product was purified by RP flash chromatography.

Yield: 95 mg (50% of th.).

^{1}H NMR: (400 MHz, DMSO-*d$_6$*) δ [ppm] = 8.18 (s, 3H), 7.62 (d, *J* = 8.2 Hz, 1H), 7.54 (s, 1H), 7.13 (d, *J* = 8.2 Hz, 1H), 6.66 (d, *J* = 1.9 Hz, 1H), 4.51 (d, *J* = 4.0 Hz, 2H), 4.12 (q, *J* = 5.7 Hz, 2H), 3.25 (d, *J* = 12.6 Hz, 2H), 3.10 – 3.02 (m, 2H), 1.56 (q, *J* = 5.8, 3.9 Hz, 4H), 0.98 – 0.94 (m, 6H).

^{13}C NMR: (101 MHz, DMSO) δ [ppm] = 158.4, 158.1, 136.4, 128.3, 128.0, 127.5, 120.7, 120.6, 112.4, 105.3, 51.8, 48.0, 42.9, 34.8, 34.7, 30.9, 27.4, 25.5, 23.0.

LC/MS: m/z calculated for $C_{17}H_{25}N_3$[M+H]$^+$: 272.2, found:271.0.

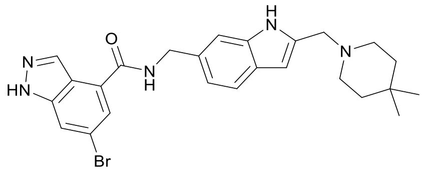

Compound **39**: **76** (40 mg, 0.147 mmol, 1.2 eq.), 6-bromo-1*H*-indazole-4-carboxylic acid (30 mg, 0.123 mmol, 1.0 eq.), TBTU (39 mg, 0.123 mmol, 1.0 eq.) and DIPEA (64 µL, 0.369 mmol, 3.0 eq.) were dissolved in ethyl acetate and stirred at RT for 16 h. The solvent was evaporated, and the crude product was purified with RP flash chromatography.

Yield: 8 mg (13% of th.).

^{1}H NMR: (400 MHz, MeO*D*) δ [ppm] = 8.54 (s, 1H), 8.37 (s, 1H), 7.91 (s, 1H), 7.88 – 7.81 (m, 1H), 7.71 (s, 1H), 7.56 (d, *J* = 8.2 Hz, 1H), 7.47 (s, 1H), 7.17 – 7.12 (m, 1H), 6.61 (s, 1H), 4.72 (s, 2H), 4.30 (s, 2H), 3.09 (dt, *J* = 7.9, 4.0 Hz, 4H), 1.61 (t, *J* = 5.9 Hz, 4H), 1.01 (s, 6H).

^{13}C NMR: (101 MHz, MeOD) δ [ppm] = 169.9, 168.3, 138.7, 134.7, 130.5, 129.3, 128.6, 127.2, 124.5, 121.8, 121.2, 121.1, 121.1, 120.9, 117.1, 111.5, 106.4, 54.7, 50.1, 45.0, 37.2, 28.8.

LC/MS: m/z calculated for $C_{25}H_{28}BrN_5O$ $[M+H]^+$: 494.2, found: 494.1.

Purity (HPLC): 99%.

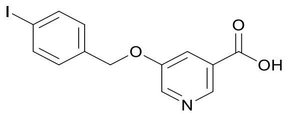

Compound **77**: Methyl 5-hydroxynicotinate (300 mg, 1.96 mmol, 1.0 eq.) was dissolved in 5 mL DMF, and NaH (156 mg, 6.5 mmol, 3.3 eq.) was added to the solution portion-wise at 0 °C. 1-(bromomethyl)-4-iodobenzene (582 mg, 1.96 mmol, 1.0 eq.) was dissolved in 1 mL DMF and added dropwise to the cooled solution. The reaction was stirred at RT for 16 h. Subsequently, the organic solvent was evaporated, and the residue was resolved in water and acidified with concentrated TFA. The precipitation was filtered and dried to afford the desired compound.

Yield: 485 mg (70% of th.).

^{1}H NMR: (300 MHz, DMSO-d_6) δ [ppm] = 8.68 (d, *J* = 1.4 Hz, 1H), 8.56 (d, *J* = 2.9 Hz, 1H), 7.83 – 7.74 (m, 4H), 7.27 (s, 3H), 5.23 (s, 2H).

LC/MS: m/z calculated for $C_{13}H_{10}INO_3$ $[M+H]^+$: 356.0, found: 356.0.

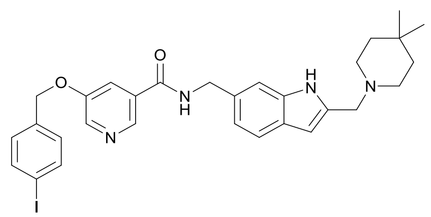

Compound **78**: **77** (7 mg, 0.02 mmol, 1.0 eq.), **76** (5.4 mg, 0.02 mmol, 1.0 eq.), HATU (7.6 mg, 0.02 mmol, 1.0 eq.), and DIPEA (14 µL, 0.08 mmol, 4.0 eq.) were dissolved in ethyl acetate and stirred at RT for 16 h. The solvent was evaporated, and the crude product was purified by RP flash chromatography.

Yield: 1.5 mg (12% of th.).

No NMR analytics available due to instability of this intermediate in NMR solvents.

LC/MS: m/z calculated for $C_{30}H_{33}IN_4O_2$ $[M+2H]^{2+}$: 305.1, found: 305.2.

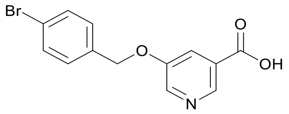

Compound **79**: Methyl 5-hydroxynicotinate (300 mg, 1.96 mmol, 1.0 eq.) was dissolved in 5 mL DMF, and NaH (156 mg, 6.5 mmol, 3.3 eq.) was added to the solution portion-wise at 0 °C. 1-(bromomethyl)-4-bromobenzene (488 mg, 1.96 mmol, 1.0 eq.) was dissolved in 1 mL DMF and added dropwise to the cooled solution. The reaction was stirred at RT for 16 h. Subsequently, the organic solvent was evaporated, and the crude product was purified via NP flash chromatography to afford the desired compound.

Yield: 200 mg (33% of th.).

^{1}H NMR: (300 MHz, MeO*D*) δ [ppm] = 8.81 (s, 1H), 8.60 (s, 1H), 8.16 (s, 1H), 7.62 – 7.57 (m, 2H), 7.44 (d, *J* = 8.0 Hz, 2H), 5.27 (s, 2H).

^{13}C NMR: (75 MHz, MeOD) δ [ppm] = 166.7, 141.8, 140.7, 136.4, 133.9, 132.9, 132.0, 130.8, 126.0, 123.3, 71.3.

LC/MS: m/z calculated for $C_{13}H_{10}BrNO_3$ $[M+H]^+$: 308.0, found: 307.9.

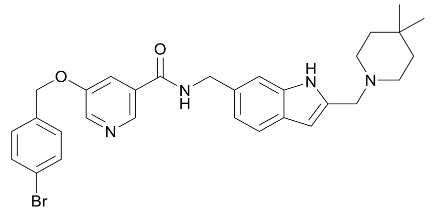

Compound **80**: **79** (40 mg, 0.13 mmol, 1.0 eq.) was dissolved in anhydrous DCM (4 mL). HATU (49 mg, 0.13 mmol, 1.0 eq.) and DIPEA (113 µL, 0.65 mmol, 5.0 eq.) were added at 0 °C. The reaction was stirred at 0 °C for 90 min before **76** (50 mg, 0.185 mmol, 1.4 eq.) dissolved in 2 mL DCM/DMF (1:1) was added at 0 °C. The reaction was stirred at RT for 16 h. The solvents were evaporated, and the crude product was purified with RP flash chromatography.

Yield: 42 mg (58% of th.).

^{1}H NMR: (400 MHz, DMSO-d_6) δ [ppm] = 9.18 (t, *J* = 5.9 Hz, 1H), 8.68 (s, 1H), 8.49 (d, *J* = 2.9 Hz, 1H), 7.88 – 7.85 (m, 1H), 7.64 – 7.58 (m, 3H), 7.46 – 7.42 (m, 3H), 7.31 (s, 1H), 7.00 – 6.94 (m, 1H), 6.30 (s, 1H), 5.23 (d, *J* = 11.8 Hz, 3H), 4.55 (d, *J* = 5.9 Hz, 2H), 3.77 (s, 2H), 2.05 – 1.89 (m, 1H), 1.37 (t, *J* = 5.6 Hz, 4H), 1.30 – 1.17 (m, 1H), 0.89 (s, 7H).

^{13}C NMR: (101 MHz, DMSO) δ [ppm] = 164.2, 163.1, 154.2, 142.6, 140.8, 140.6, 136.4, 135.7, 131.5, 130.6, 123.0, 129.9, 126.8, 121.3, 121.1, 119.8, 119.5, 119.0, 110.2, 69.0, 49.1, 43.3, 40.2, 28.0.

LC/MS: m/z calculated for $C_{30}H_{33}BrN_4O_2$ $[M+H]^+$: 561.2, found: 561.2.

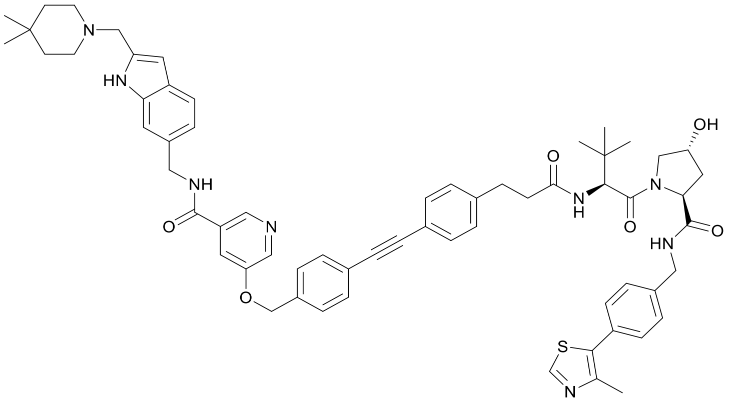

Compound **40**: **69** (4.8 mg, 0.008 mmol, 1.0 eq.), **78** (5.0 mg, 0.008 mmol, 1.0 eq.), Tris(dibenzylidenaceton)dipaladium(0) (0.8 mg, 0.0008 mmol, 0.1 eq.), copper(I)iodide (0.3 mg, 0.0016 mmol, 0.2 eq.), and X-Phos (0.8 mg, 0.0016 mmol, 0.2 eq.) were dissolved in 0.5 mL degassed DMF/THF (1:1) and triethylamine (3.2 µL, 0.0024 mmol, 3.0 eq.). The reaction was stirred at 80 °C for 3 h. Subsequently, the organic solvents were evaporated, and the crude product was purified with RP flash chromatography.

Yield: 0.6 mg (7% of th.).

^{1}H NMR: (600 MHz, DMSO-*d*$_6$) δ [ppm] = 10.91 (s, 1H), 9.21 – 9.18 (m, 1H), 8.98 (s, 1H), 8.69 (s, 1H), 8.61 – 8.58 (m, 1H), 8.51 (s, 1H), 8.00 (d, *J* = 9.5 Hz, 1H), 7.90 – 7.87 (m, 1H), 7.61 (d, *J* = 8.3 Hz, 1H), 7.57 (d, *J* = 8.2 Hz, 2H), 7.52 (d, *J* = 8.3 Hz, 2H), 7.46 – 7.37 (m, 6H), 7.28 (d, *J* = 7.9 Hz, 2H), 7.07 (d, *J* = 9.2 Hz, 1H), 6.95 – 6.92 (m, 1H), 6.21 (s, 1H), 5.27 (s, 2H), 5.18 – 5.16 (m, 1H), 4.55 (dd, *J* = 7.5, 4.1 Hz, 2H), 4.46 – 4.38 (m, 3H), 4.36 (s, 1H), 4.23 – 4.15 (m, 2H), 3.69 – 3.63 (m, 2H), 3.56 (s, 1H), 2.84 (ddt, *J* = 21.2, 14.6, 8.2 Hz, 3H), 2.61 (d, *J* = 22.3 Hz, 2H), 2.44 (s, 3H), 2.33 (s, 3H), 2.07 – 1.96 (m, 3H), 1.90 (ddd, *J* = 12.9, 9.5, 4.5 Hz, 2H), 1.33 – 1.29 (m, 3H), 1.27 – 1.22 (m, 2H), 0.89 (s, 5H), 0.87 (s, 6H).

^{13}C NMR: (151 MHz, DMSO) δ [ppm] = 172.0, 171.1, 169.6, 164.2, 154.3, 151.6, 147.8, 142.5, 140.8, 140.7, 139.6, 136.9, 136.6, 136.4, 131.5, 131.5, 131.3, 131.2, 129.7, 128.8, 128.7, 128.1, 127.4, 126.9, 119.8, 119.6, 119.3, 118.9, 110.1, 100.4, 69.3, 68.9, 68.8, 58.7, 56.5, 56.4, 55.6, 49.4, 43.3, 41.7, 40.1, 38.3, 38.0, 36.1, 35.3, 28.2, 26.4, 16.0.

LC/MS: m/z calculated for $C_{63}H_{70}N_8O_6S$ $[M+2H]^{2+}$: 534.3, found: 534.6.

Purity (HPLC): 97%.

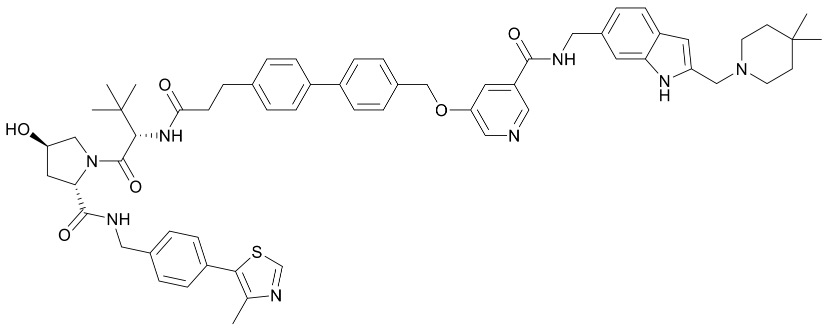

Compound **41**: **65** (5.4 mg, 0.009 mmol, 1.1 eq.), **80** (5 mg, 0.008 mmol, 1.0 eq.), potassium phosphate (3.4 mg, 0.016 mmol, 2.0 eq.), X-Phos (1 mg, 0.002 mmol, 0.2 eq.), and tris(dibenzylidenacetone)dipalladium (1 mg, 0.001 mmol, 0.1 eq.) were dissolved under argon atmosphere in 120 µL degassed water/ dioxane (1:4). The reaction was stirred at 70 °C for 1.5 h. The mixture was diluted with water, and the mixture was directly purified by RP flash chromatography.

Yield: 2.4 mg (25% of th.).

^{1}H NMR: (600 MHz, DMSO-d_6) δ [ppm] = 9.21 (t, *J* = 6.0 Hz, 1H), 8.98 (s, 1H), 8.68 (dd, *J* = 4.2, 1.7 Hz, 1H), 8.59 (t, *J* = 6.1 Hz, 1H), 8.51 (d, *J* = 2.8 Hz, 1H), 7.99 (d, *J* = 9.4 Hz, 1H), 7.92 – 7.89 (m, 1H), 7.68 – 7.65 (m, 2H), 7.59 – 7.51 (m, 4H), 7.45 – 7.35 (m, 5H), 7.31 (d, *J* = 8.2 Hz, 2H), 6.98 – 6.92 (m, 2H), 6.25 (s, 2H), 5.27 (s, 2H), 5.17 (d, *J* = 3.5 Hz, 1H), 4.55 (dd, *J* = 7.7, 5.1 Hz, 3H), 4.43 (td, *J* = 9.2, 8.4, 5.2 Hz, 2H), 4.35 (d, *J* = 4.4 Hz, 1H), 4.21 (dd, *J* = 15.9, 5.5 Hz, 2H), 3.67 (d, *J* = 3.8 Hz, 3H), 2.86 (dq, *J* = 14.0, 6.2 Hz, 3H), 2.67 – 2.59 (m, 3H), 2.44 (s, 3H), 2.07 – 1.99 (m, 2H), 1.90 (ddd, *J* = 12.9, 8.8, 4.6 Hz, 2H), 1.34 (s, 3H), 1.26 – 1.22 (m, 3H), 1.15 (t, *J* = 6.4 Hz, 2H), 0.88 (d, *J* = 6.1 Hz, 11H).

^{13}C NMR: (151 MHz, DMSO) δ [ppm] = 172.0, 171.3, 169.7, 164.3, 163.2, 154.4, 151.5, 147.7, 140.8, 140.7, 140.7, 140.0, 139.6, 137.4, 136.4, 135.1, 131.2, 130.6, 129.7, 129.0, 128.7, 128.6, 128.0, 127.4, 126.8, 126.7, 126.6, 119.7, 110.2, 69.5, 68.9, 58.7, 56.5, 56.4, 49.2, 43.3, 41.7, 40.1, 38.0, 36.5, 35.3, 31.0, 26.4, 24.1, 16.0.

LC/MS: m/z calculated for $C_{61}H_{70}N_8O_6S$ $[M+2H]^{2+}$: 522.3, found: 523.0.

Purity (HPLC): 95%.

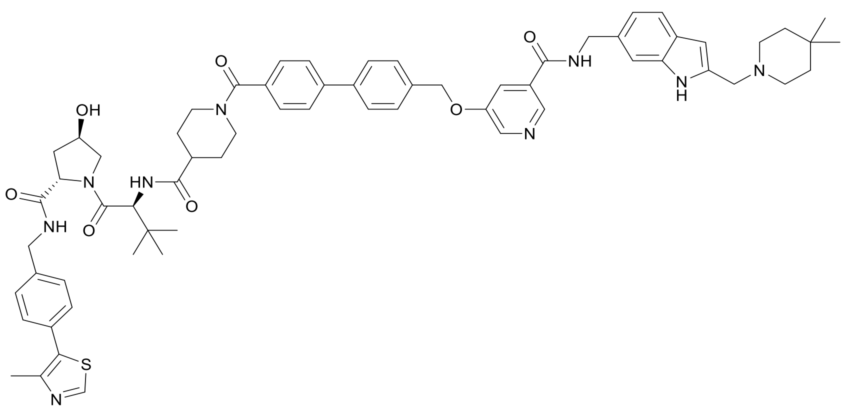

Compound **42**: **66** (6.2 mg, 0.009 mmol, 1.1 eq.), **80** (5 mg, 0.008 mmol, 1.0 eq.), potassium phosphate (3.4 mg, 0.016 mmol, 2.0 eq.), X-Phos (1 mg, 0.002 mmol, 0.2 eq.), and tris(dibenzylidenacetone)dipalladium (1 mg, 0.001 mmol, 0.1 eq.) were dissolved under argon atmosphere in 120 µL degassed water/ dioxane (1:4). The reaction was stirred at 70 °C for 1.5 h. The mixture was diluted with water and purified by RP flash chromatography.

Yield: 6.3 mg (70% of th.).

^{1}H NMR: (600 MHz, DMSO-d_6) δ [ppm] = 9.21 (t, *J* = 6.0 Hz, 2H), 8.98 (s, 1H), 8.69 – 8.67 (m, 1H), 8.59 (t, *J* = 6.1 Hz, 1H), 8.51 (d, *J* = 2.8 Hz, 1H), 7.99 (d, *J* = 9.4 Hz, 1H), 7.90 (dd, *J* = 2.9, 1.8 Hz, 1H), 7.68 – 7.65 (m, 2H), 7.58 – 7.52 (m, 4H), 7.43 – 7.36 (m, 5H), 7.31 (d, *J* = 8.2 Hz, 3H), 6.97 – 6.92 (m, 2H), 5.27 (s, 1H), 5.17 (d, *J* = 3.5 Hz, 1H), 4.55 (dd, *J* = 7.7, 5.1 Hz, 3H), 4.43 (td, *J* = 9.2, 8.4, 5.2 Hz, 3H), 4.35 (d, *J* = 4.4 Hz, 1H), 4.21 (dd, *J* = 15.9, 5.5 Hz, 2H), 3.67 (d, *J* = 3.8 Hz, 2H), 2.91 – 2.80 (m, 4H), 2.66 – 2.60 (m, 3H), 2.44 (s, 3H), 2.06 – 2.00 (m, 2H), 1.90 (ddd, *J* = 12.9, 8.8, 4.6 Hz, 2H), 1.34 (s, 4H), 1.25 – 1.19 (m, 4H), 1.15 (t, *J* = 6.4 Hz, 2H), 0.88 (d, *J* = 6.1 Hz, 14H).

^{13}C NMR: (151 MHz, DMSO) δ [ppm] = 172.0, 171.3, 169.7, 164.3, 163.2, 154.4, 151.5, 147.7, 140.8, 140.7, 140.0, 139.6, 137.4, 136.4, 135.1, 131.2, 130.6, 129.7, 129.0, 128.7, 128.6, 128.0, 127.4, 126.8, 126.7, 126.6, 119.7, 110.2, 69.5, 68.9, 58.7, 56.4, 49.2, 43.3, 41.7, 38.0, 36.5, 35.3, 31.0, 26.4, 24.1, 16.0.

LC/MS: m/z calculated for $C_{65}H_{75}N_9O_7S$ $[M+2H]^{2+}$: 563.8, found: 564.1.

Purity (HPLC): 97%.

Spectral appendix

Compound **35**:

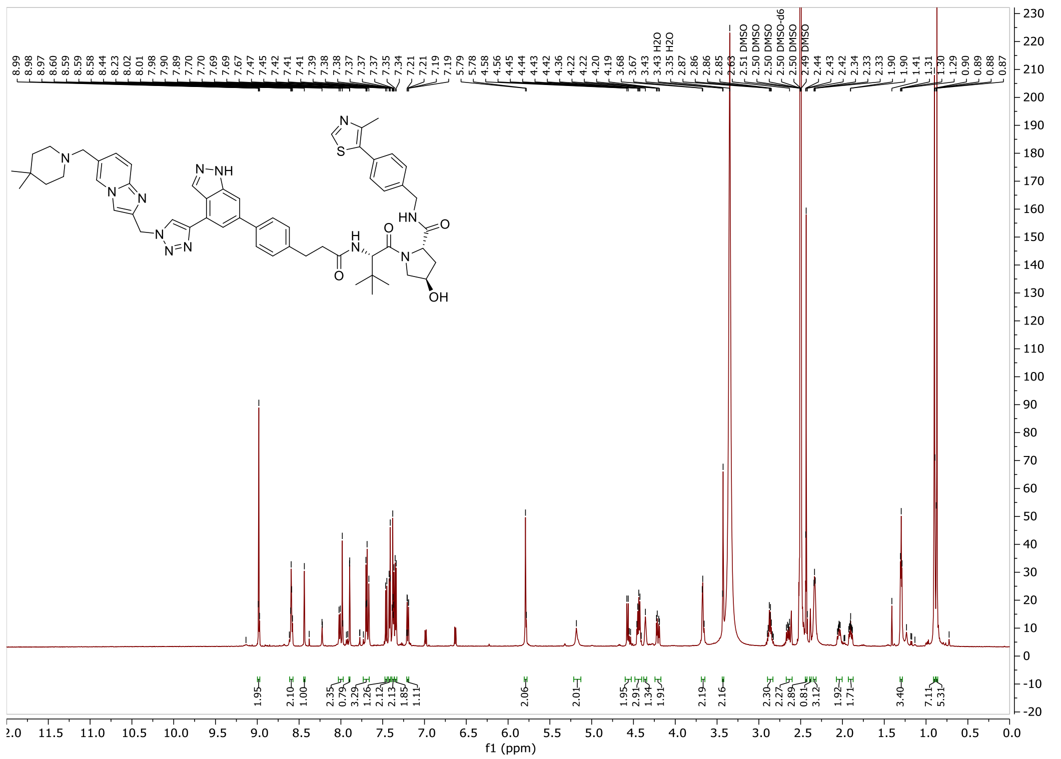

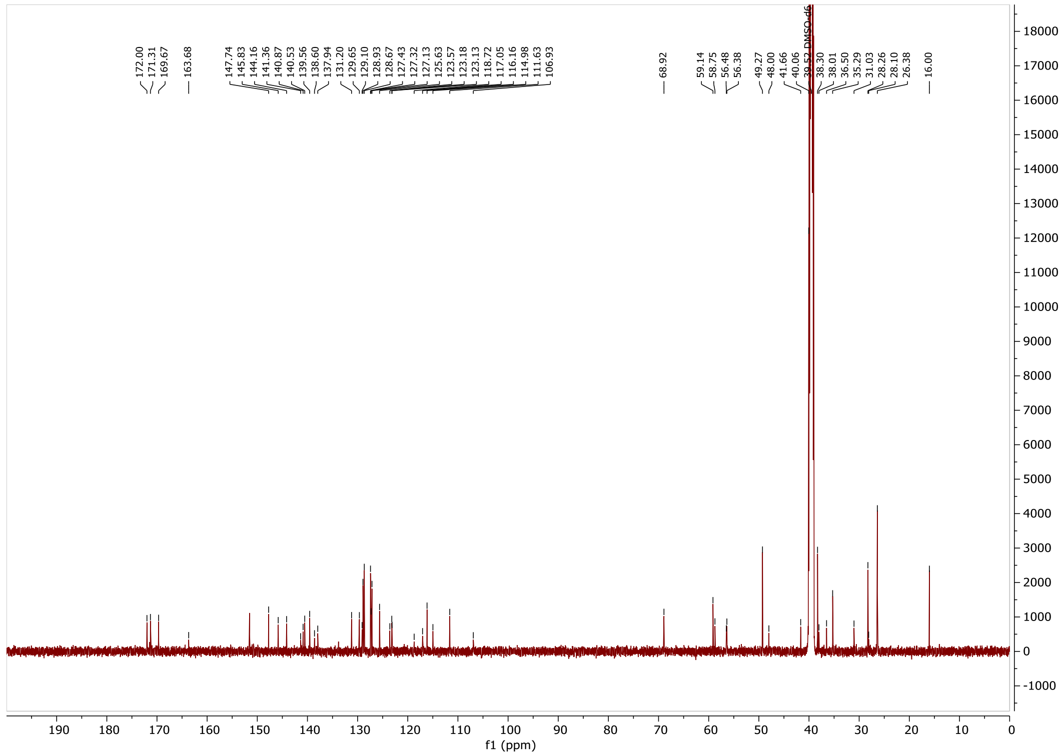

Compound **36**

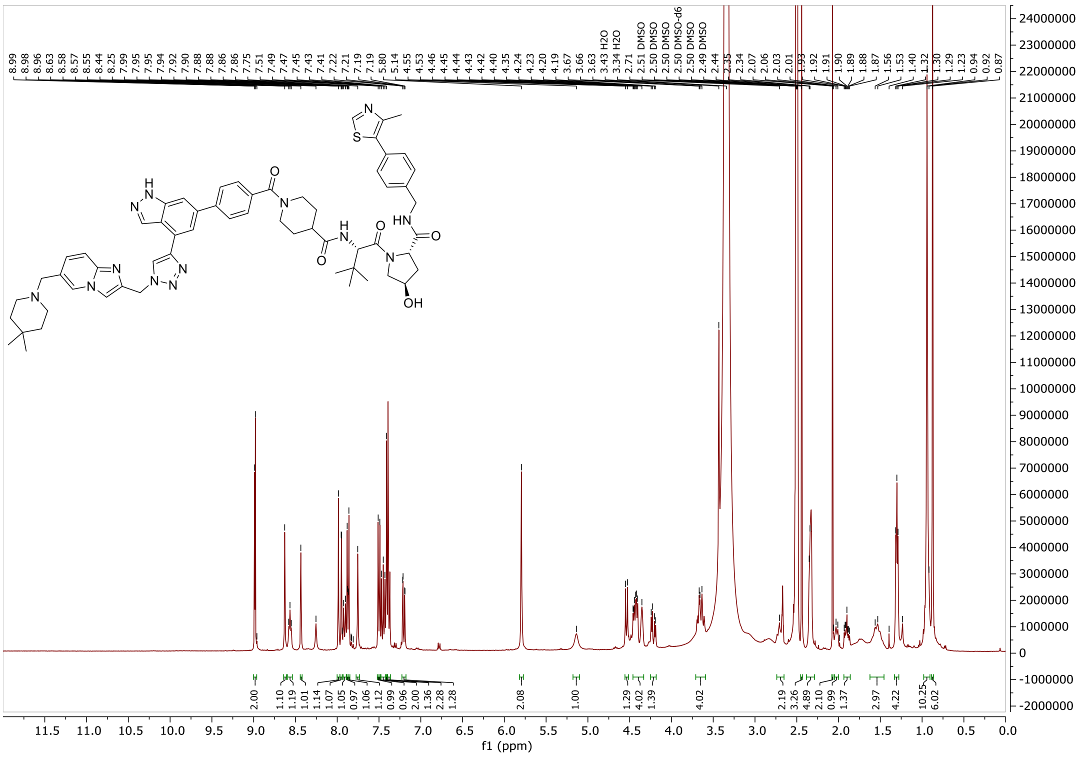
8.99 8.98 8.96 8.63 8.58 8.57 8.55 8.44 8.25 7.99 7.95 7.95 7.94 7.92 7.90 7.88 7.88 7.86 7.86 7.75 7.51 7.49 7.47 7.45 7.43 7.41 7.22 7.21 7.19 7.19 5.80 5.14 4.55 4.53 4.46 4.45 4.44 4.43 4.42 4.40 4.35 4.24 4.23 4.20 4.19 3.67 3.66 3.63
3.43 H2O
3.34 H2O
2.71
2.51 DMSO
2.50 DMSO
2.50 DMSO-d6
2.50 DMSO
2.49 DMSO
2.44 2.35 2.34 2.07 2.06 2.03 2.01 1.93 1.92 1.91 1.90 1.89 1.88 1.87 1.56 1.53 1.40 1.32 1.30 1.29 1.23 0.94 0.92 0.87
2.00 1.10 1.19 1.01 1.14 1.07 1.05 0.97 1.06 1.12 0.99 0.96 2.00 1.36 2.28 1.28 2.08 1.00 1.29 4.02 1.39 4.02 2.19 3.26 4.89 2.10 0.99 1.37 2.97 4.22 10.25 6.02
f1 (ppm)

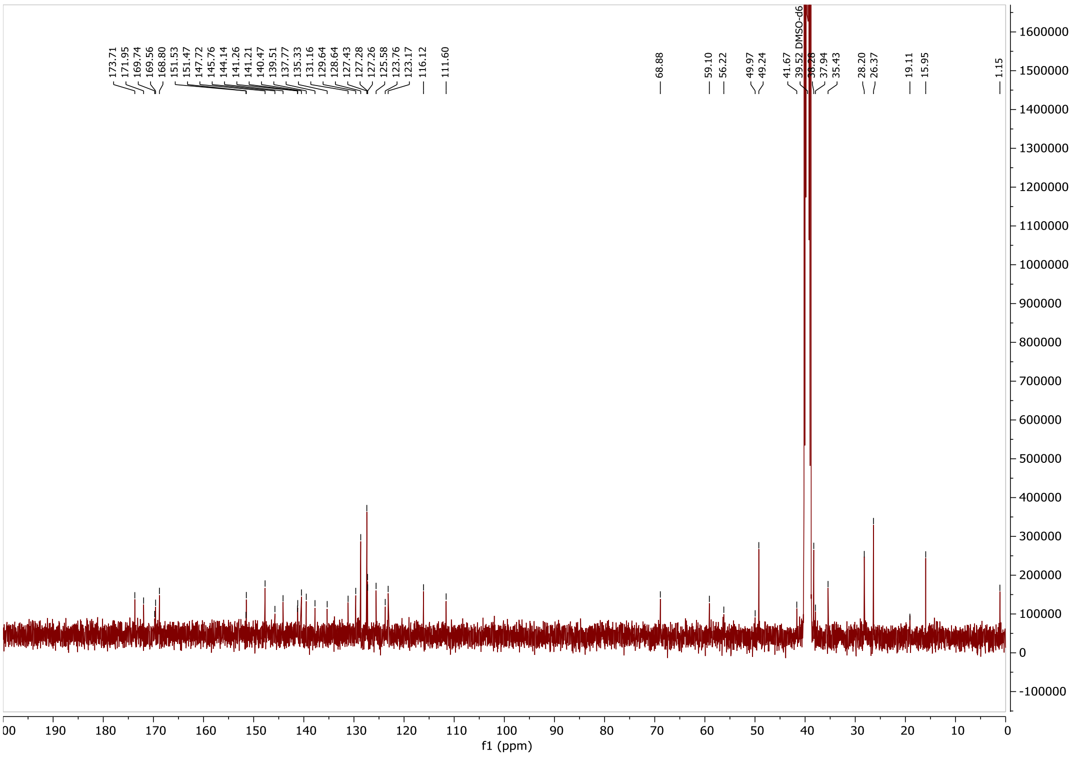
173.71 171.95 169.74 169.56 168.80 151.53 151.47 147.72 145.76 144.14 141.26 141.21 140.47 139.51 137.77 135.33 131.16 129.64 128.64 127.43 127.28 127.26 125.58 123.76 123.17 116.12 111.60 68.88 59.10 56.22 49.97 49.24 41.67
39.52 DMSO-d6
37.94 35.43 28.20 26.37 19.11 15.95 1.15
f1 (ppm)

Compound **38**

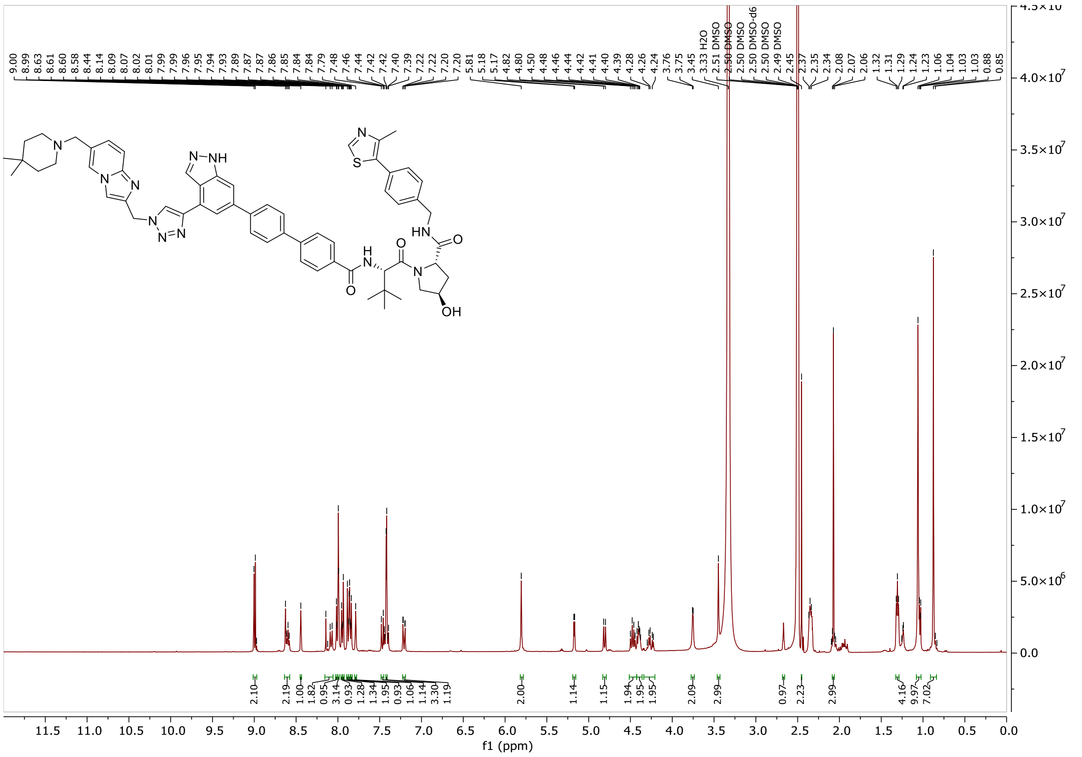

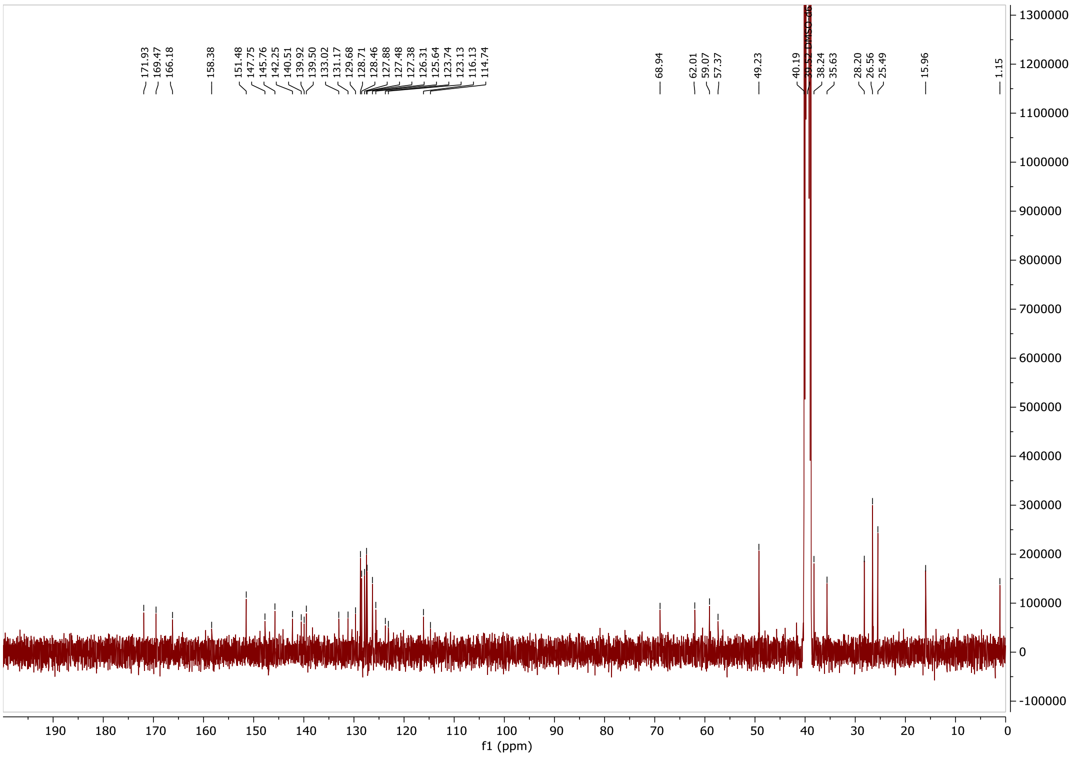

Compound **37**

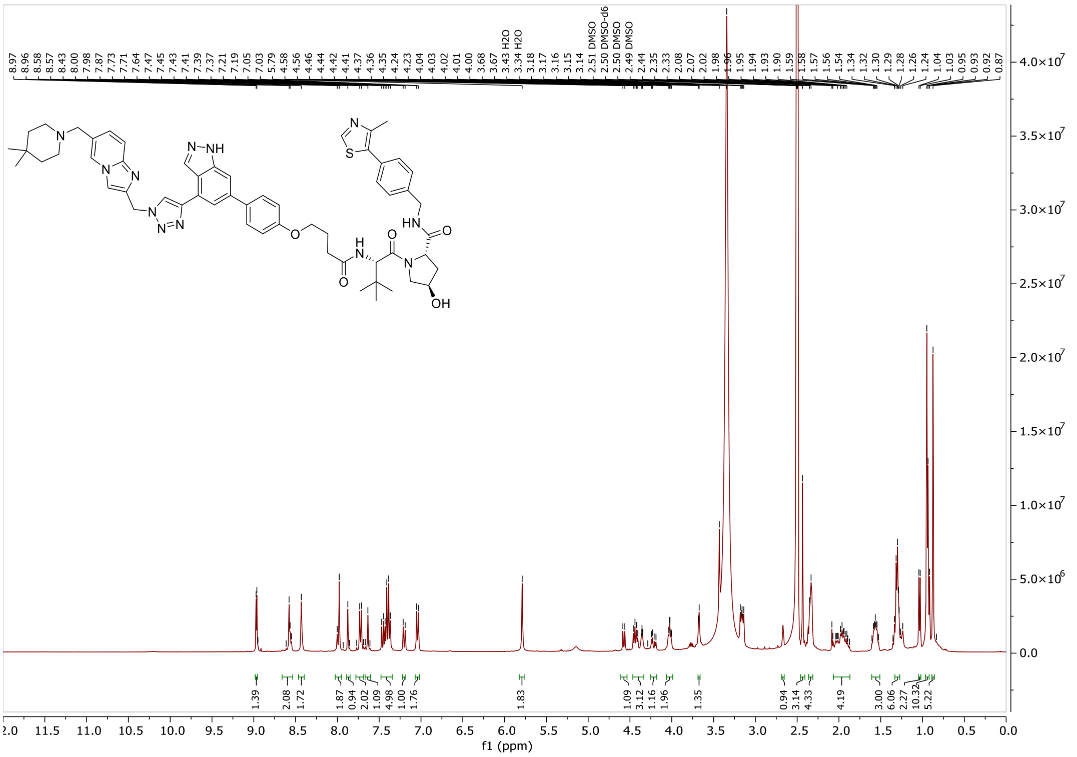
8.97
8.96
8.58
8.57
8.43
8.00
7.98
7.87
7.73
7.71
7.64
7.47
7.45
7.43
7.41
7.39
7.37
7.21
7.19
7.05
7.03
5.79
4.58
4.56
4.46
4.44
4.42
4.41
4.37
4.36
4.35
4.24
4.23
4.04
4.03
4.02
4.01
4.00
3.68
3.67
3.43 H2O
3.34 H2O
3.18
3.17
3.16
3.15
3.14
2.51 DMSO
2.50 DMSO-d6
2.50 DMSO
2.49 DMSO
2.44
2.35
2.33
2.08
2.07
2.02
1.98
1.96
1.95
1.94
1.93
1.90
1.59
1.58
1.57
1.56
1.54
1.34
1.32
1.30
1.29
1.28
1.26
1.24
1.04
1.03
0.95
0.93
0.92
0.87
N
NH
N
N
N
N
N
N
S
O
O
H
N
O
N
HN
O
OH
1.39
2.08
1.72
1.87
0.94
2.02
1.09
4.98
1.00
1.76
1.83
1.09
3.12
1.16
1.96
1.35
0.94
3.14
4.33
4.19
3.00
6.06
2.27
10.32
5.22
f1 (ppm)

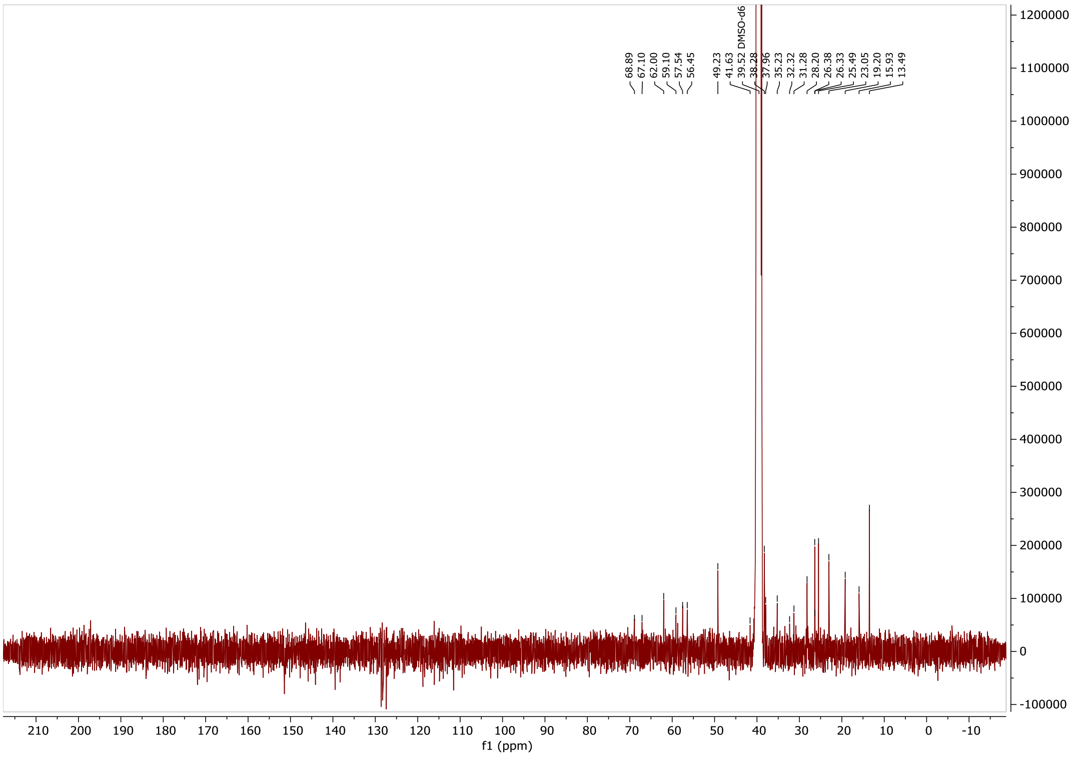
68.89
67.10
62.00
59.10
57.54
56.45
49.23
41.63
39.52 DMSO-d6
38.28
37.96
35.23
32.32
31.28
28.20
26.38
26.33
25.49
23.05
19.20
15.93
13.49
f1 (ppm)

Compound **40**

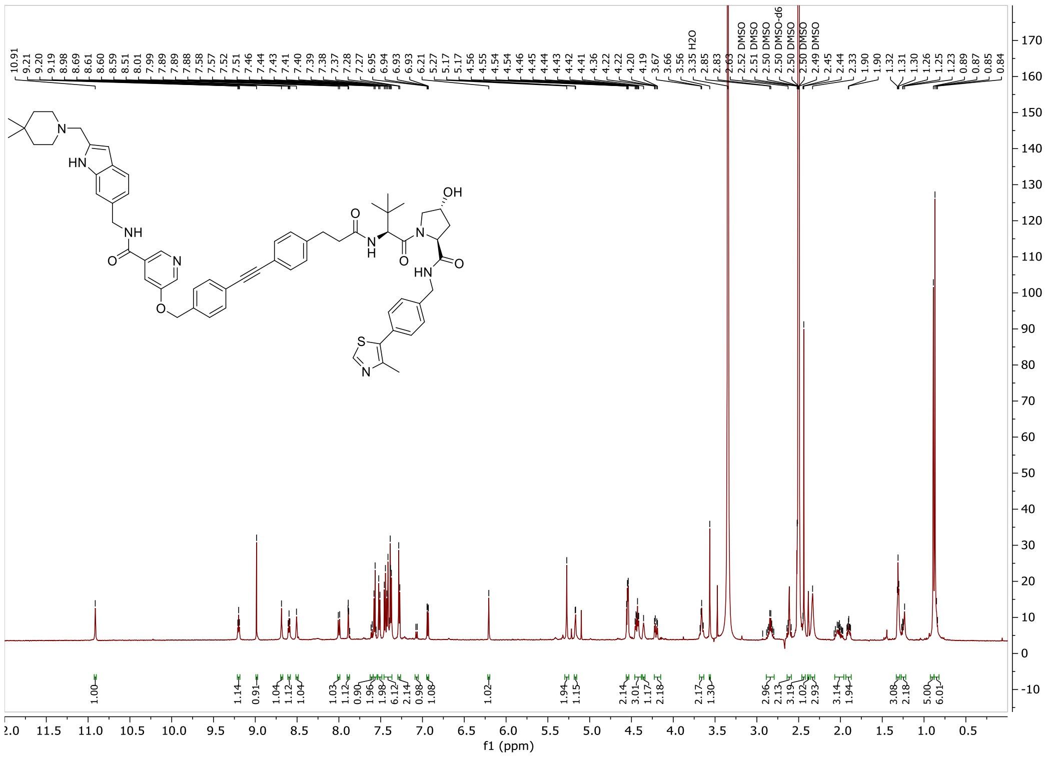

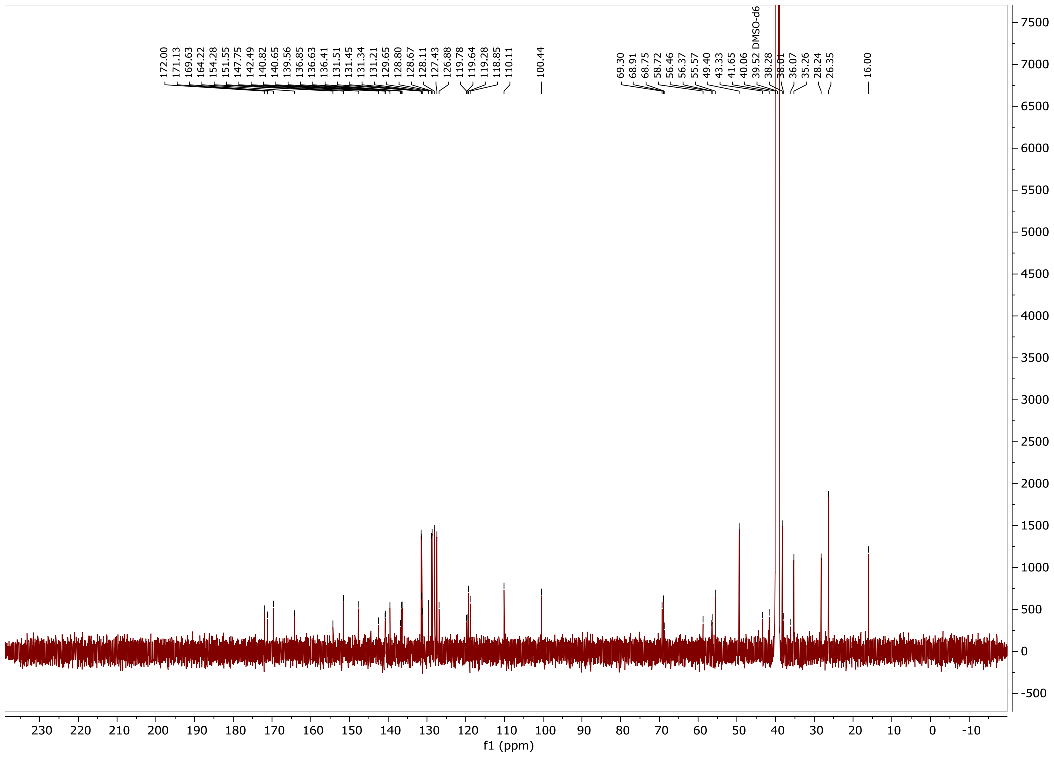

Compound **41 (AF151)**

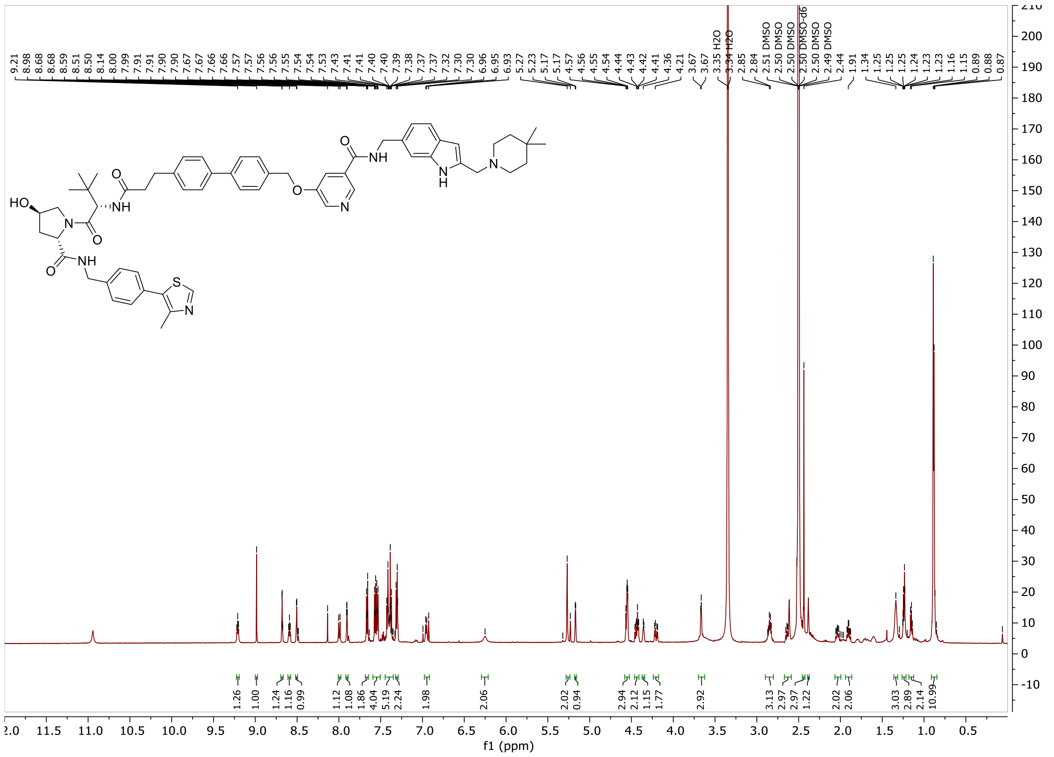

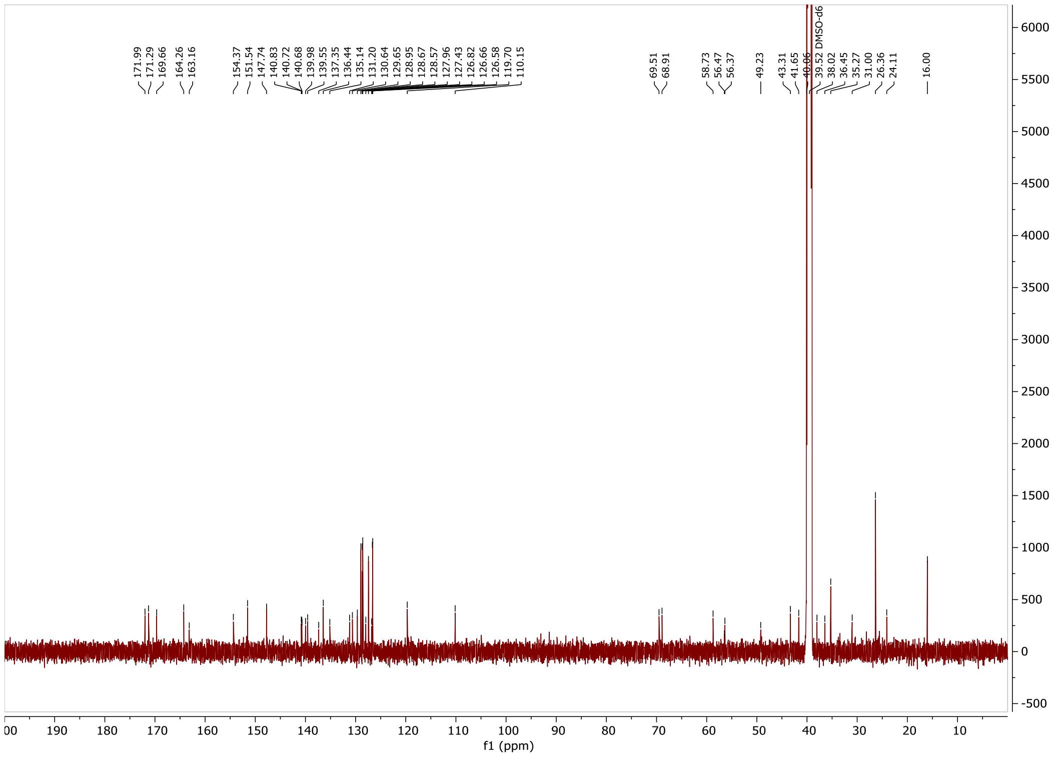

Compound **42**

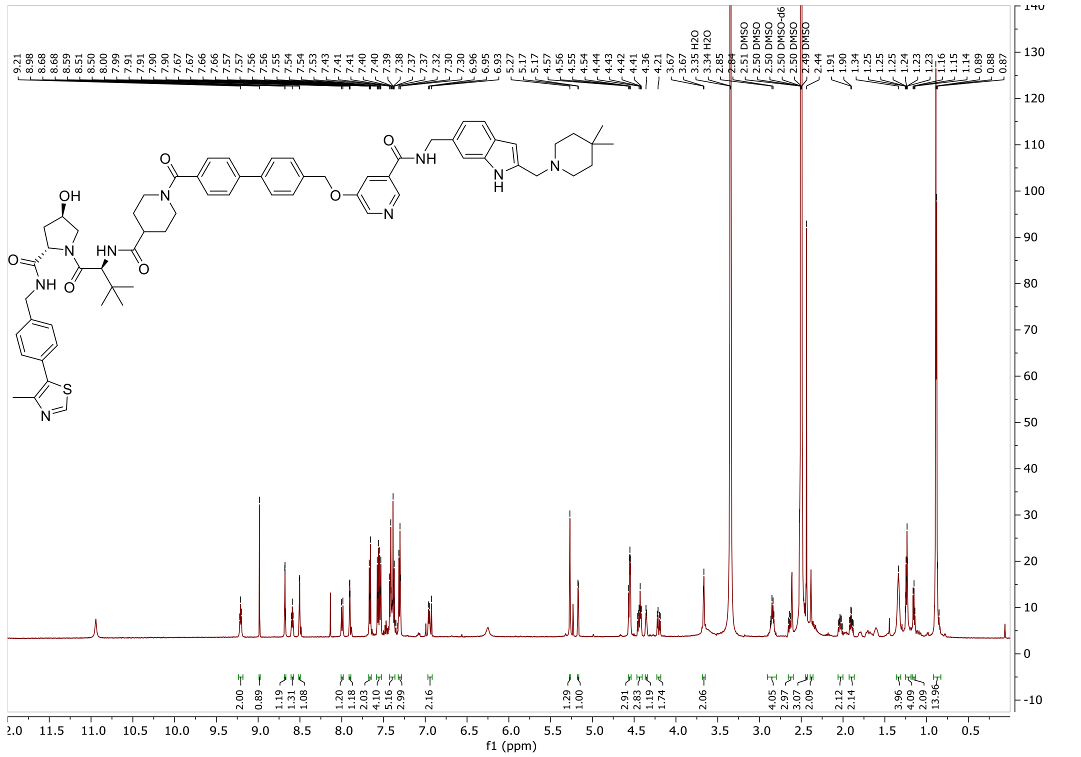

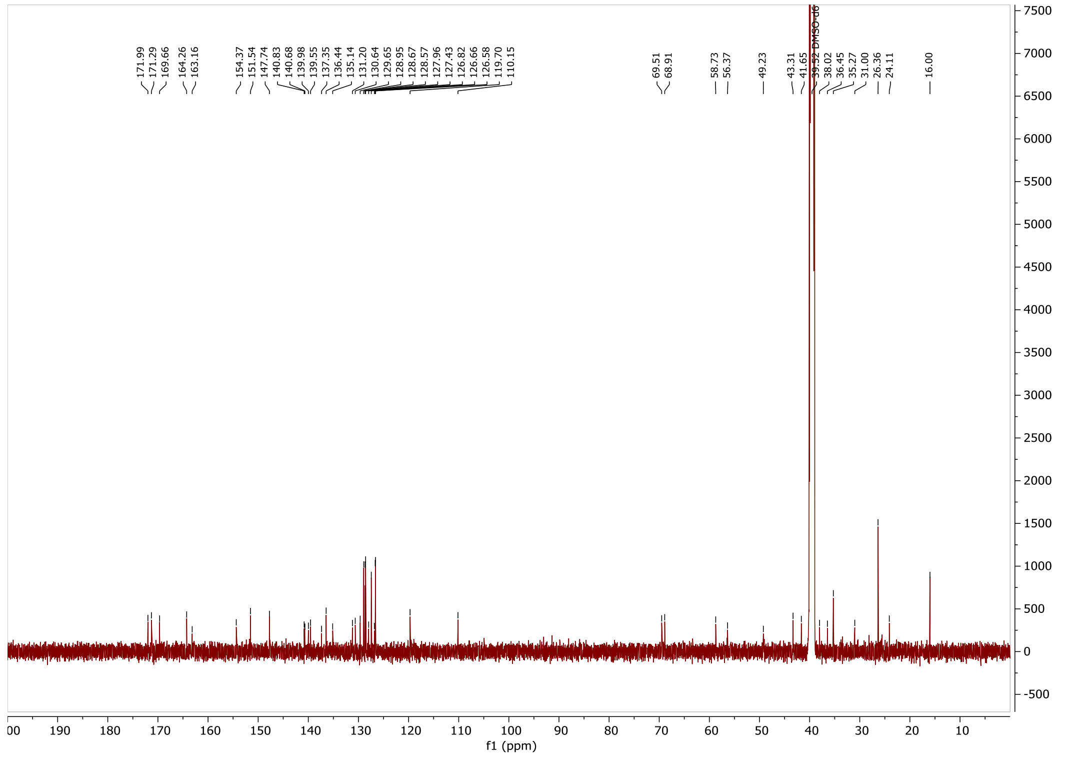

Compound **11**

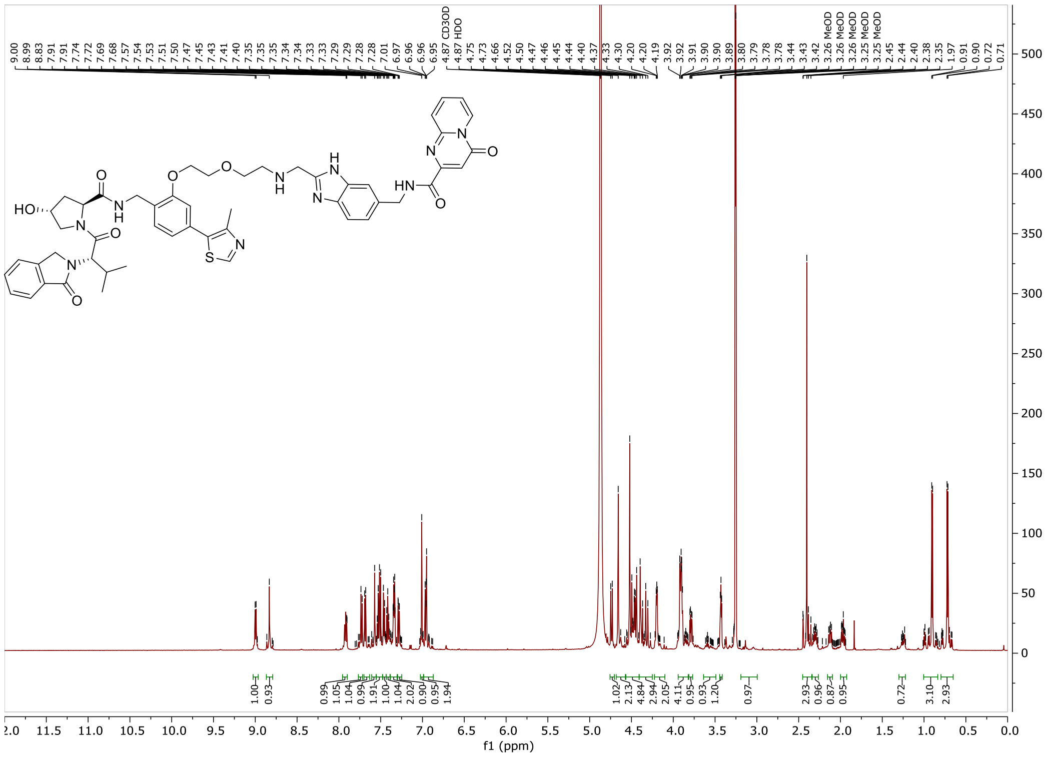

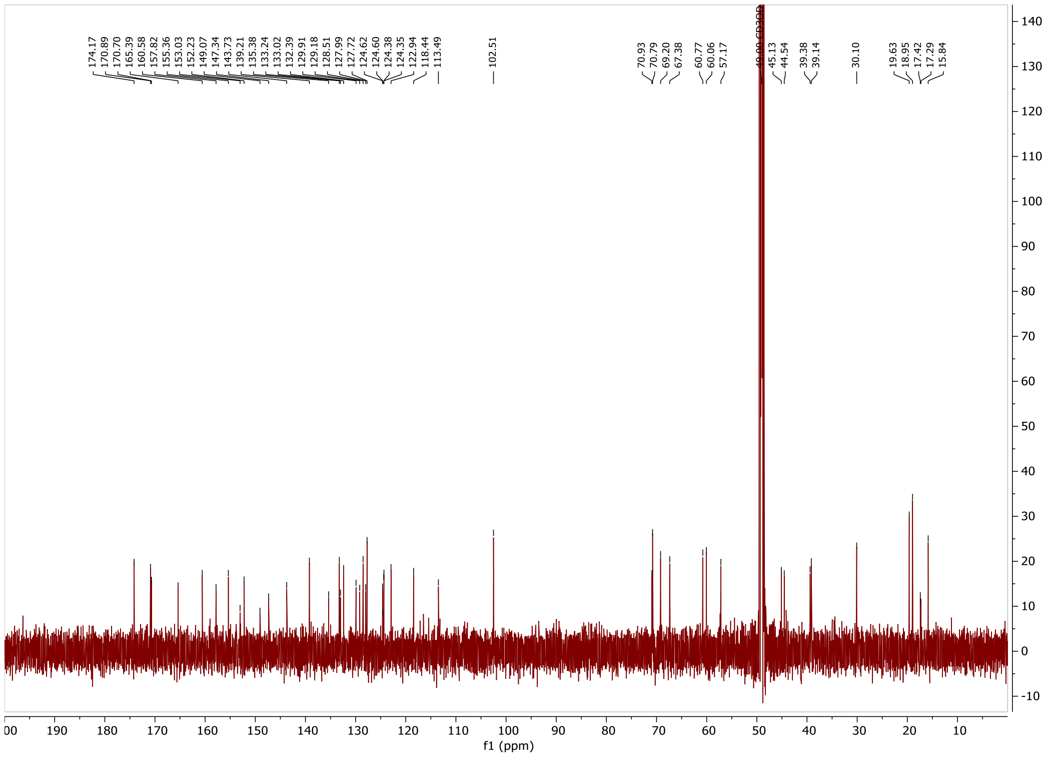

Compound **12**

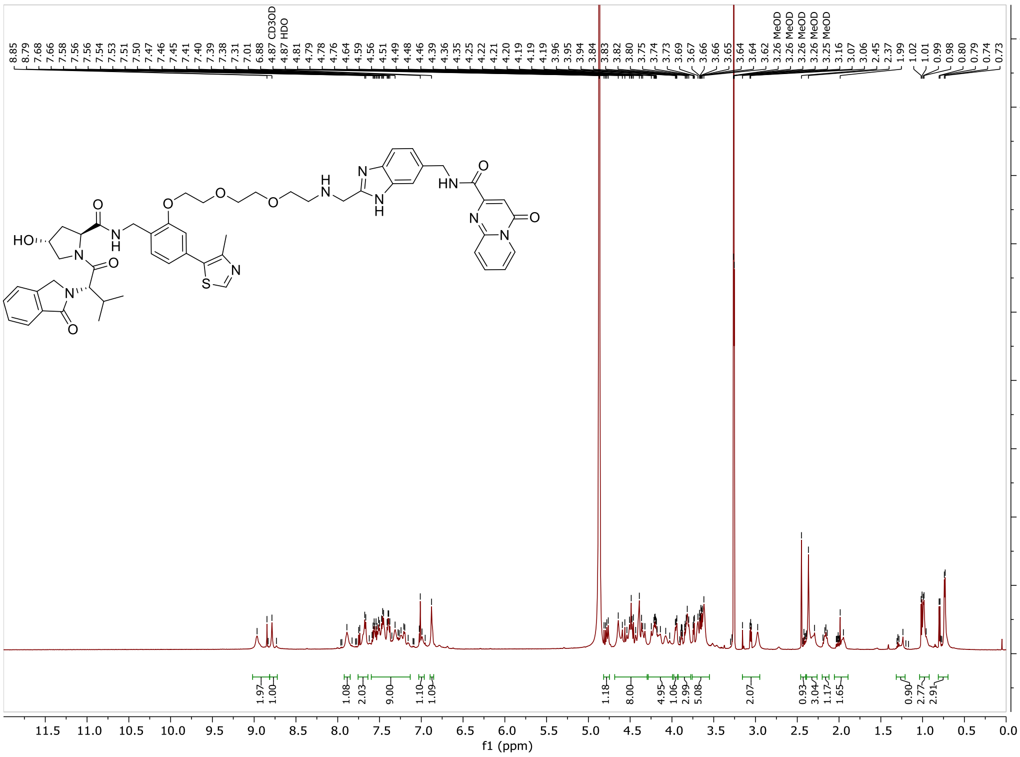
8.85 8.79 7.68 7.66 7.58 7.56 7.56 7.54 7.53 7.51 7.50 7.47 7.46 7.45 7.41 7.40 7.39 7.38 7.31 7.01 6.88 4.87 CD3OD 4.87 HDO 4.81 4.79 4.78 4.76 4.64 4.59 4.56 4.51 4.49 4.48 4.46 4.39 4.36 4.35 4.25 4.22 4.21 4.20 4.19 4.19 4.19 3.96 3.95 3.94 3.84 3.83 3.82 3.80 3.75 3.74 3.73 3.69 3.67 3.66 3.66 3.65 3.64 3.64 3.62 3.26 MeOD 3.26 MeOD 3.26 MeOD 3.26 MeOD 3.25 MeOD 3.16 3.07 3.06 2.45 2.37 1.99 1.02 1.01 0.99 0.98 0.80 0.79 0.74 0.73
1.97 1.00 1.08 2.03 9.00 1.10 1.09 1.18 8.00 4.95 1.06 2.99 5.08 2.07 0.93 3.04 1.17 1.65 0.90 2.77 2.91
f1 (ppm)

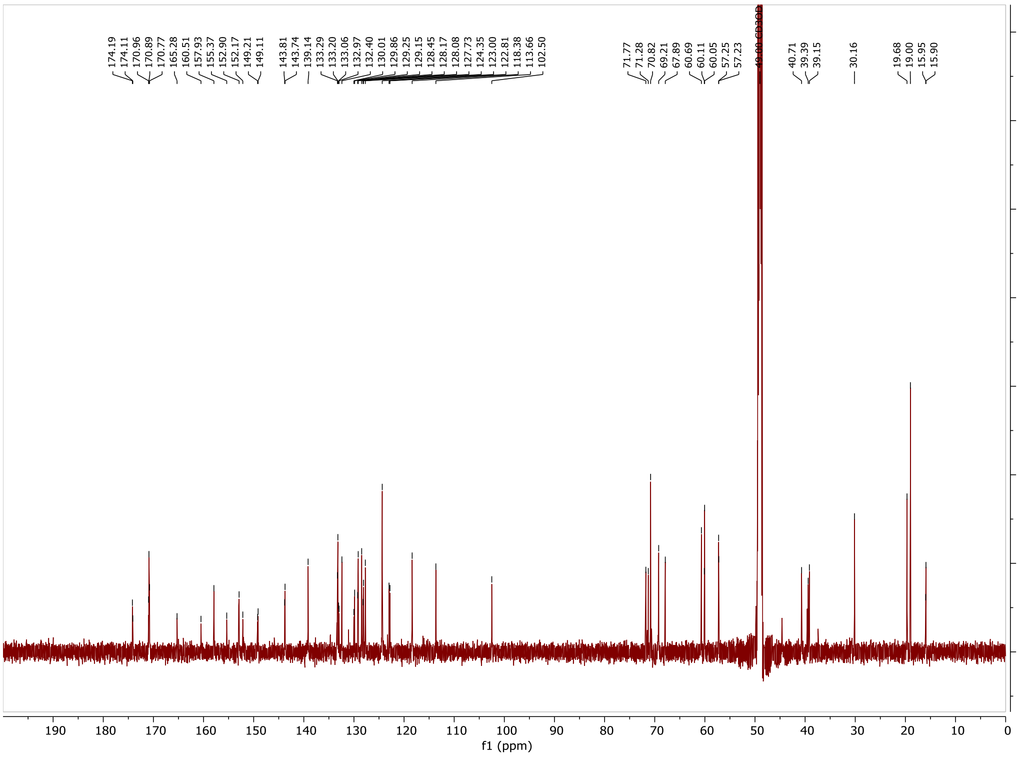
174.19 174.11 170.96 170.89 170.77 165.28 160.51 157.93 155.37 152.90 152.17 149.21 149.11 143.81 143.74 139.14 133.29 133.20 133.06 132.97 132.40 130.01 129.86 129.25 129.15 128.45 128.17 128.08 127.73 124.35 123.00 122.81 118.38 113.66 102.50 71.77 71.28 70.82 69.21 67.89 60.69 60.11 60.05 57.25 57.23 49.00 CD3OD 40.71 39.39 39.15 30.16 19.68 19.00 15.95 15.90
f1 (ppm)

Compound **13**

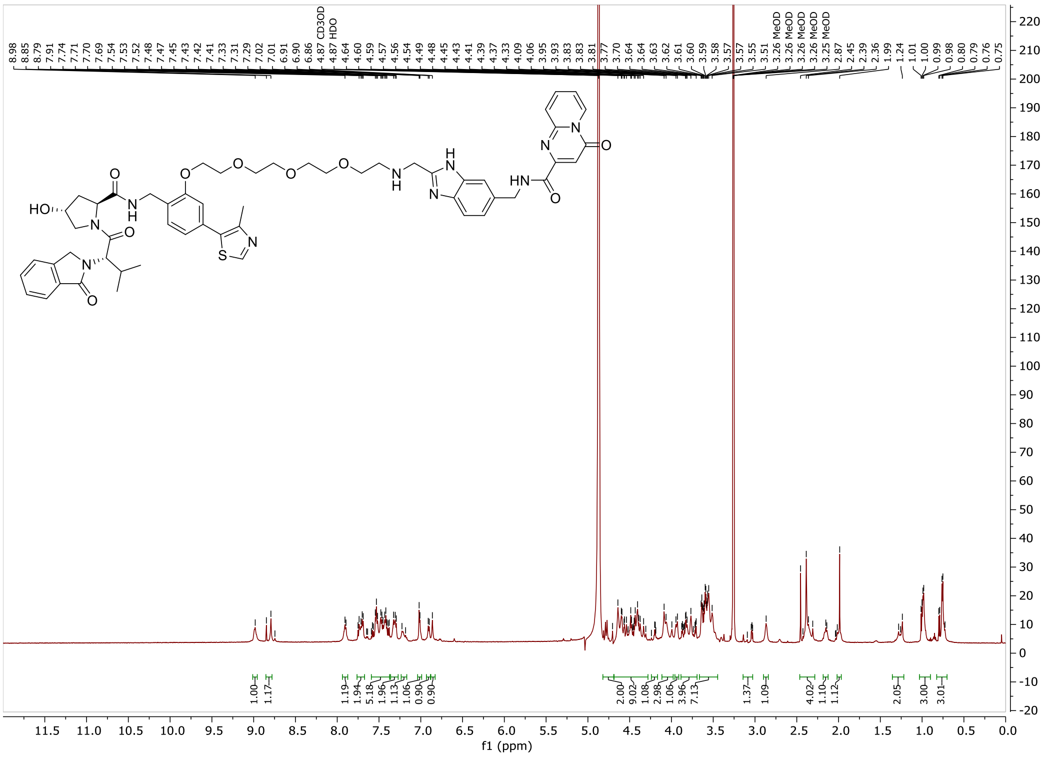
8.98
8.85
8.79
7.91
7.74
7.71
7.70
7.69
7.54
7.53
7.52
7.48
7.47
7.45
7.43
7.42
7.41
7.33
7.31
7.29
7.02
7.01
6.91
6.90
6.86
CD3OD
4.87 HDO
4.64
4.60
4.59
4.57
4.56
4.54
4.49
4.48
4.45
4.43
4.41
4.39
4.37
4.33
4.09
4.06
3.95
3.93
3.83
3.83
3.81
3.77
3.70
3.64
3.64
3.63
3.62
3.61
3.60
3.59
3.58
3.57
3.57
3.55
3.51
3.26 MeOD
3.26 MeOD
3.26 MeOD
3.26 MeOD
3.25 MeOD
2.87
2.45
2.39
2.36
1.99
1.24
1.01
1.00
0.99
0.98
0.80
0.79
0.76
0.75
1.00
1.17
1.19
1.94
5.18
1.96
1.13
1.06
0.90
0.90
2.00
9.02
1.08
2.98
1.06
3.96
7.13
1.37
1.09
4.02
1.10
1.12
2.05
3.00
3.01
HO
N
O
N
H
O
O
O
O
N
H
N
H
N
HN
O
N
N
O
S
N
N
O
O
f1 (ppm)

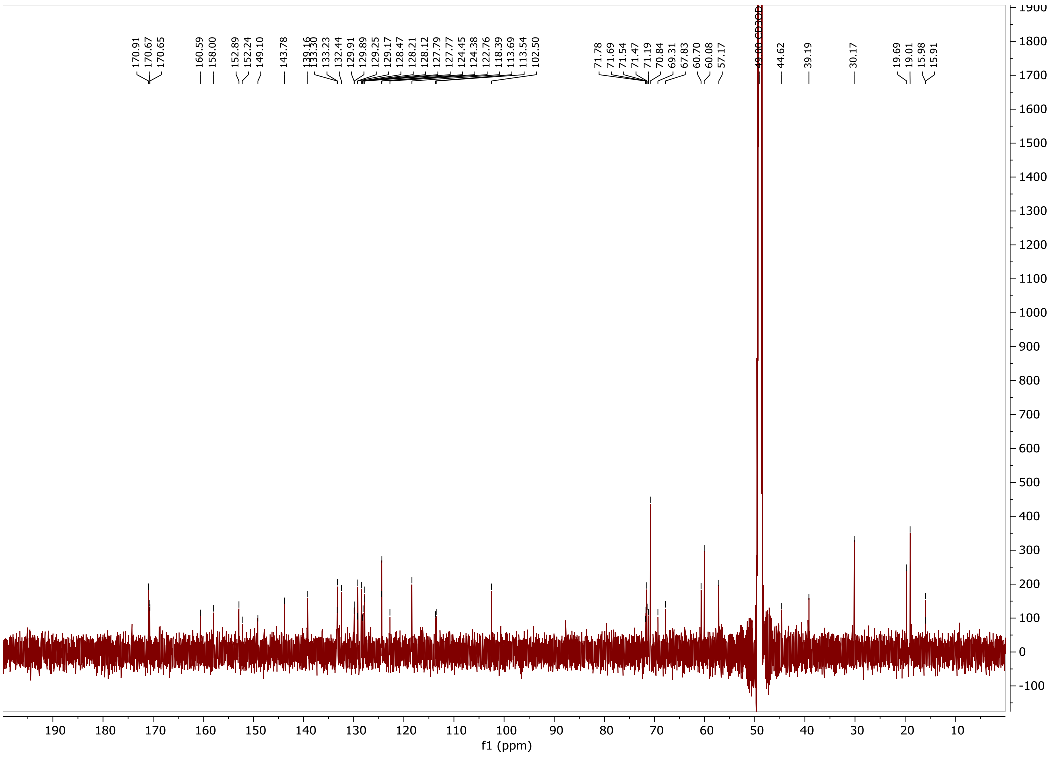
170.91
170.67
170.65
160.59
158.00
152.89
152.24
149.10
143.78
139.16
133.30
133.23
132.44
129.91
129.89
129.25
129.17
128.47
128.21
128.12
127.79
127.77
124.45
124.38
122.76
118.39
113.69
113.54
102.50
71.78
71.69
71.54
71.47
71.19
70.84
69.31
67.83
60.70
60.08
57.17
49.00 CD3OD
44.62
39.19
30.17
19.69
19.01
15.98
15.91
f1 (ppm)

Compound **14**

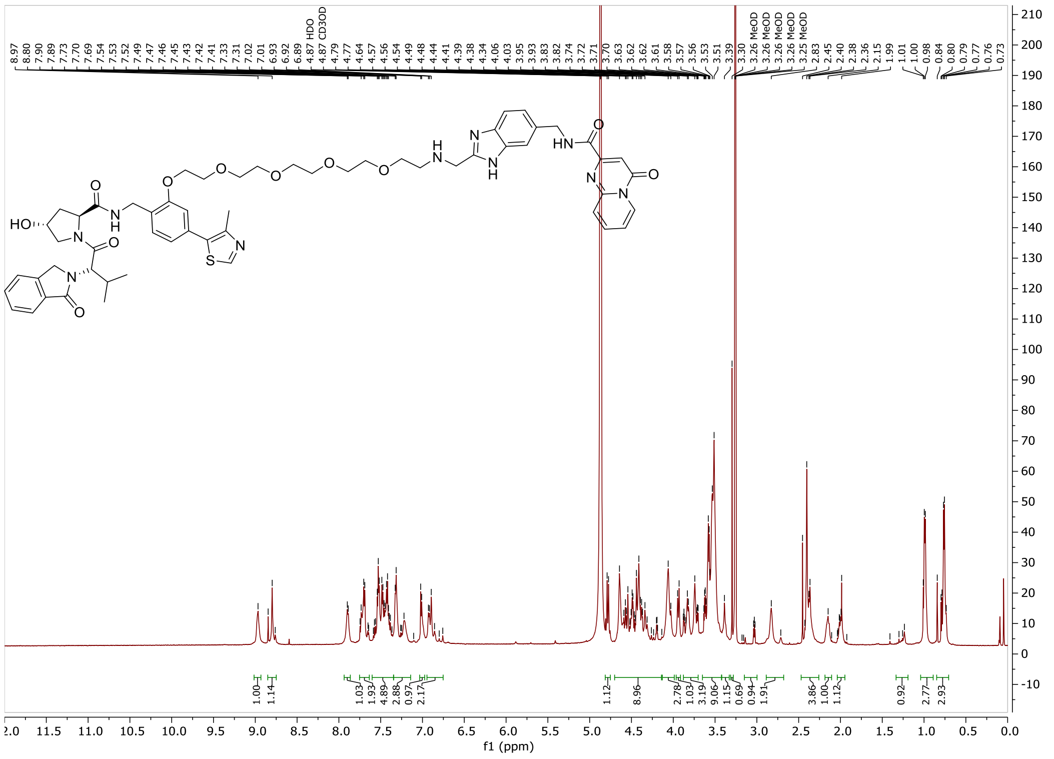

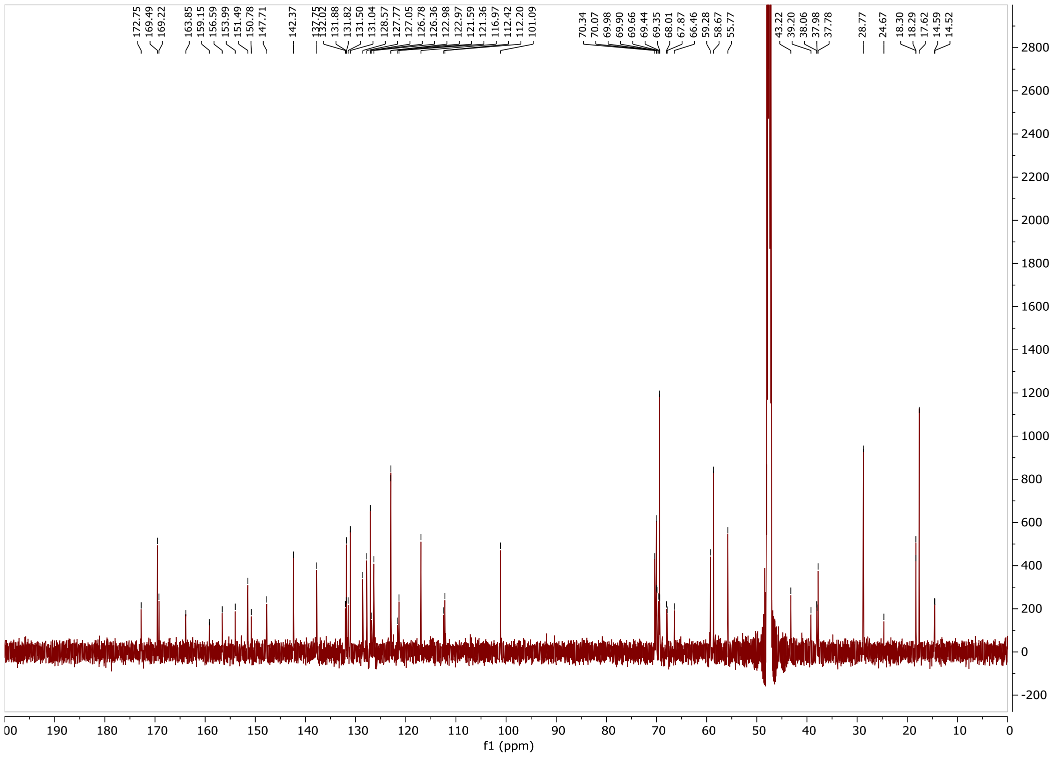

Compound **15**

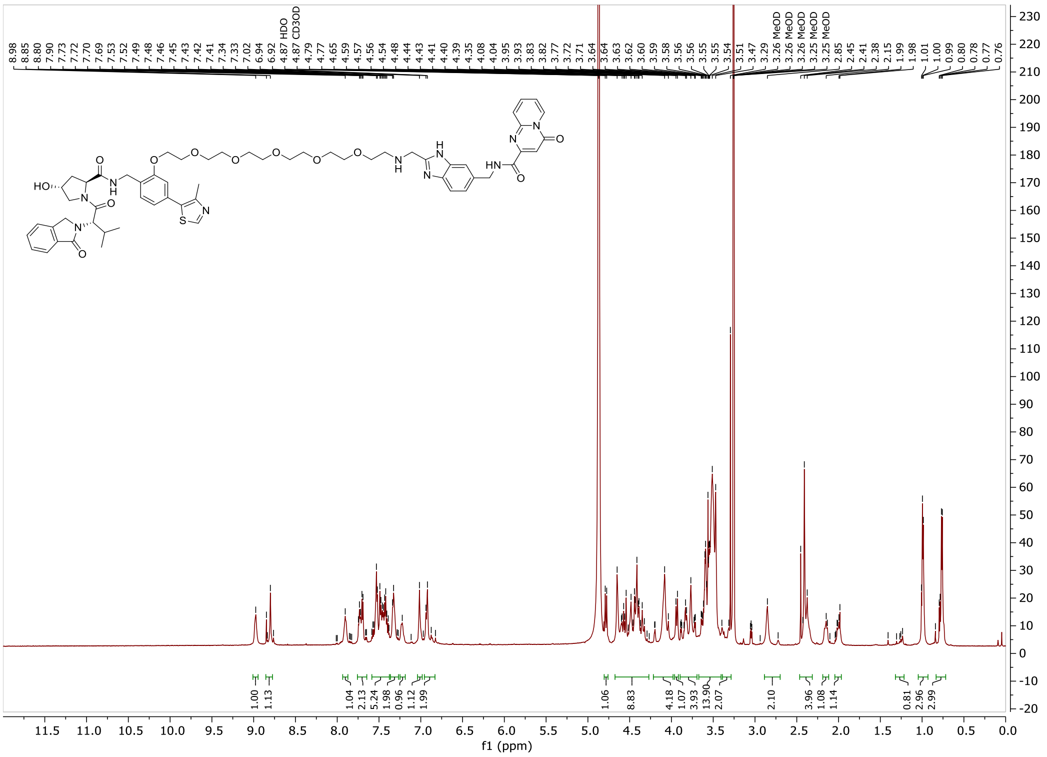

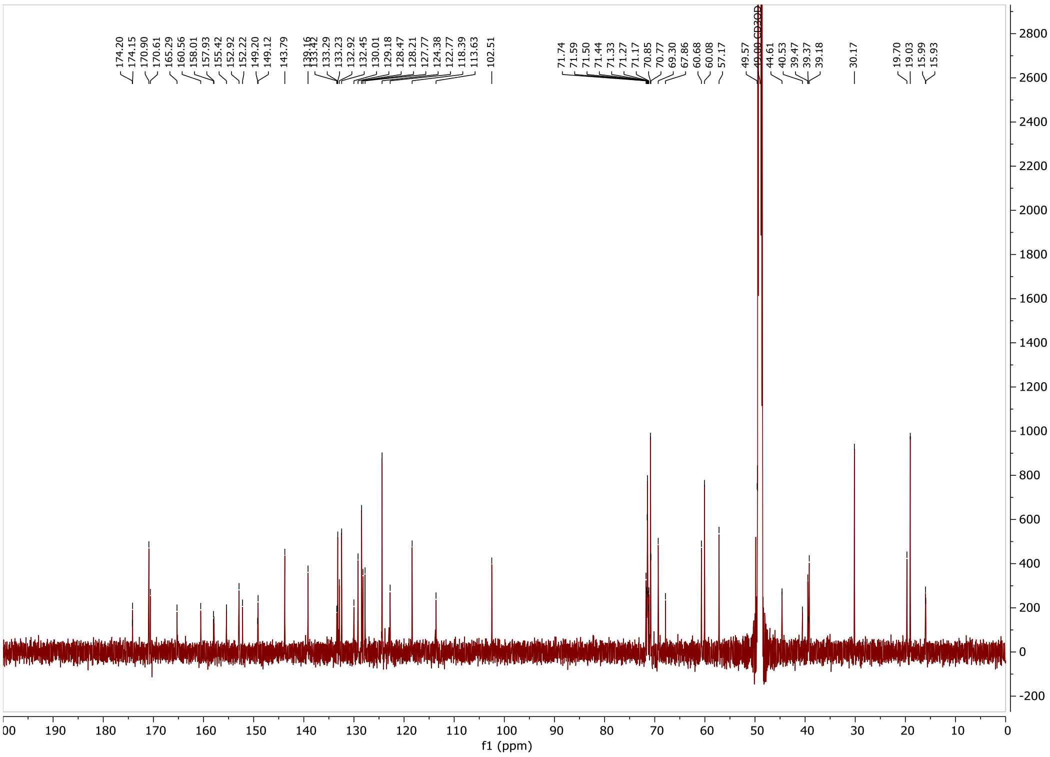

Compound **26**

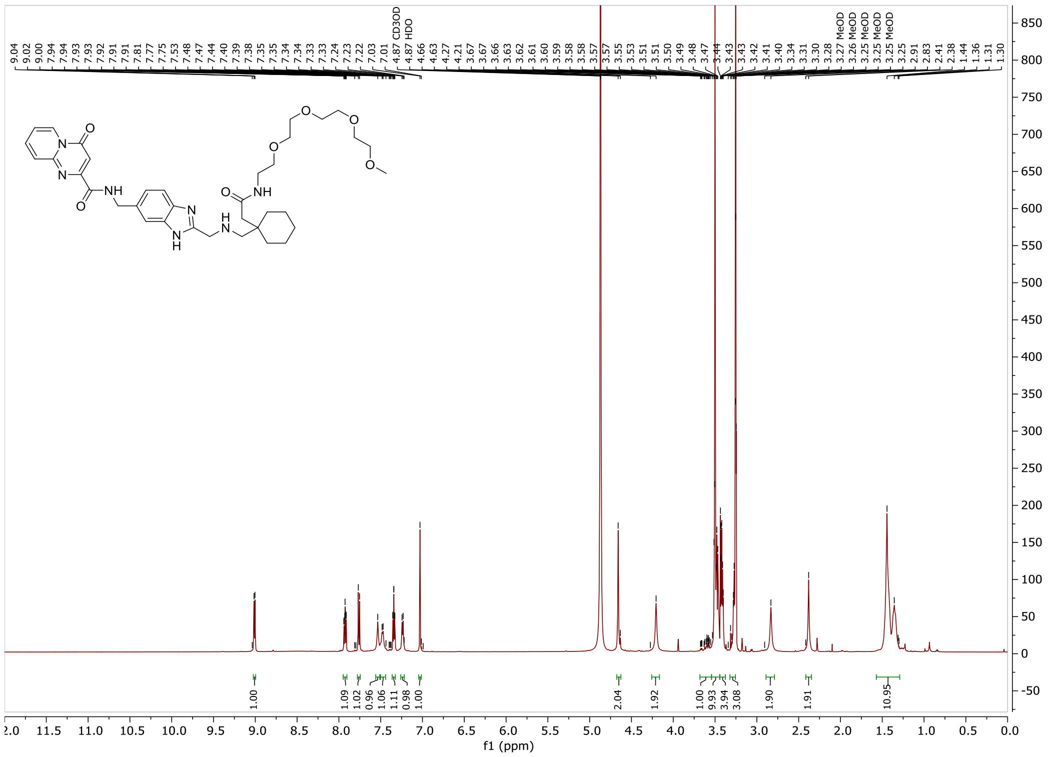

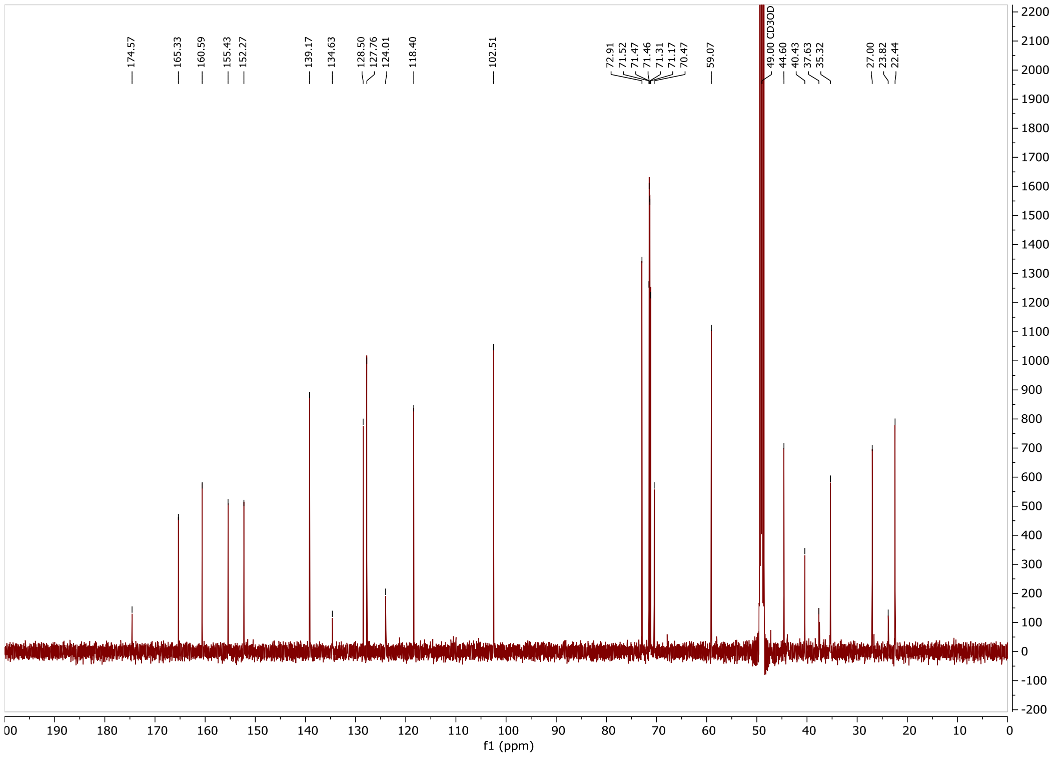

Compound **27**

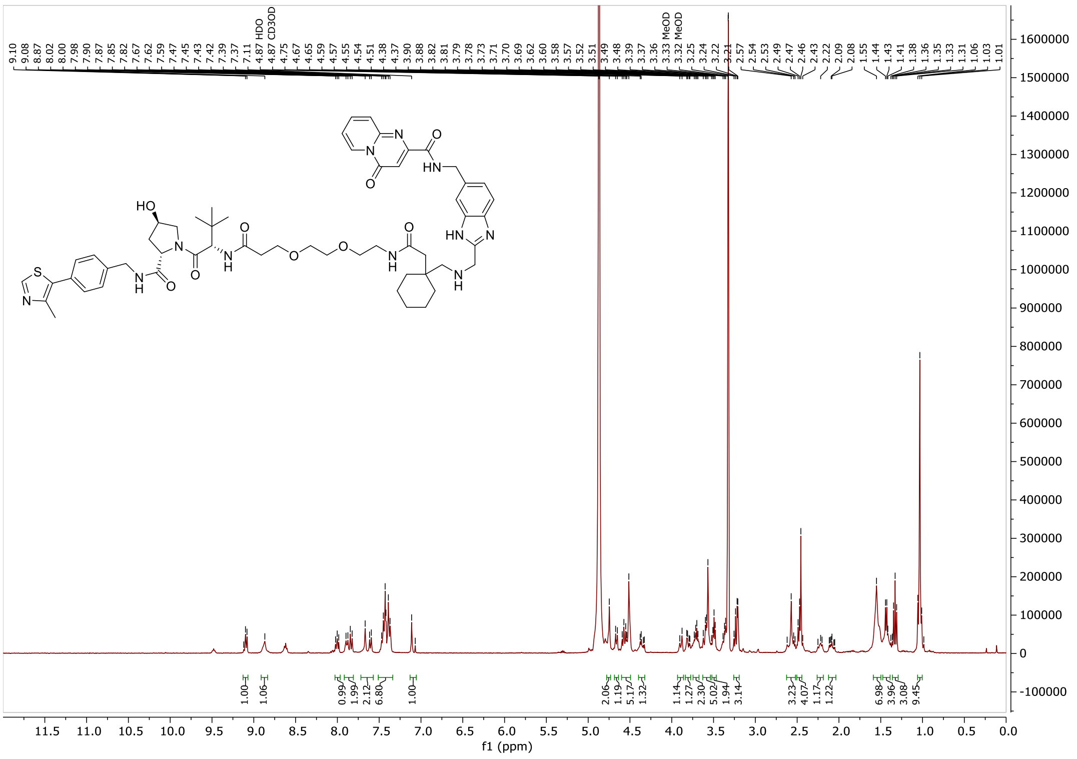

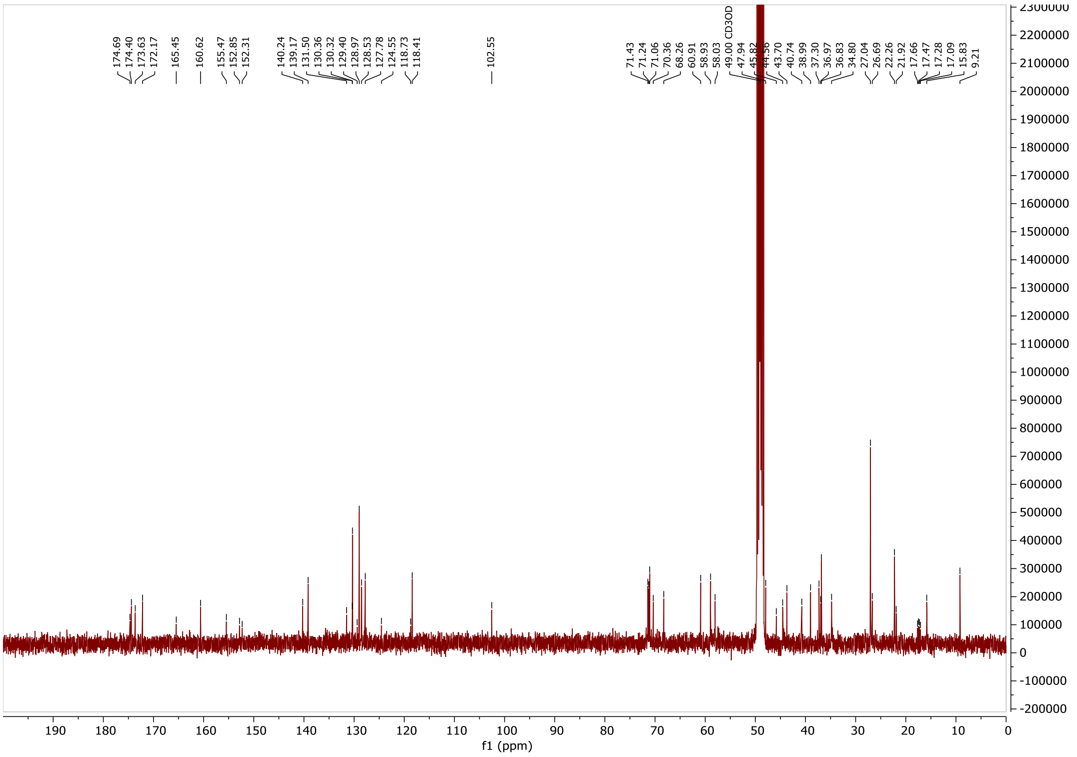

Compound **28**

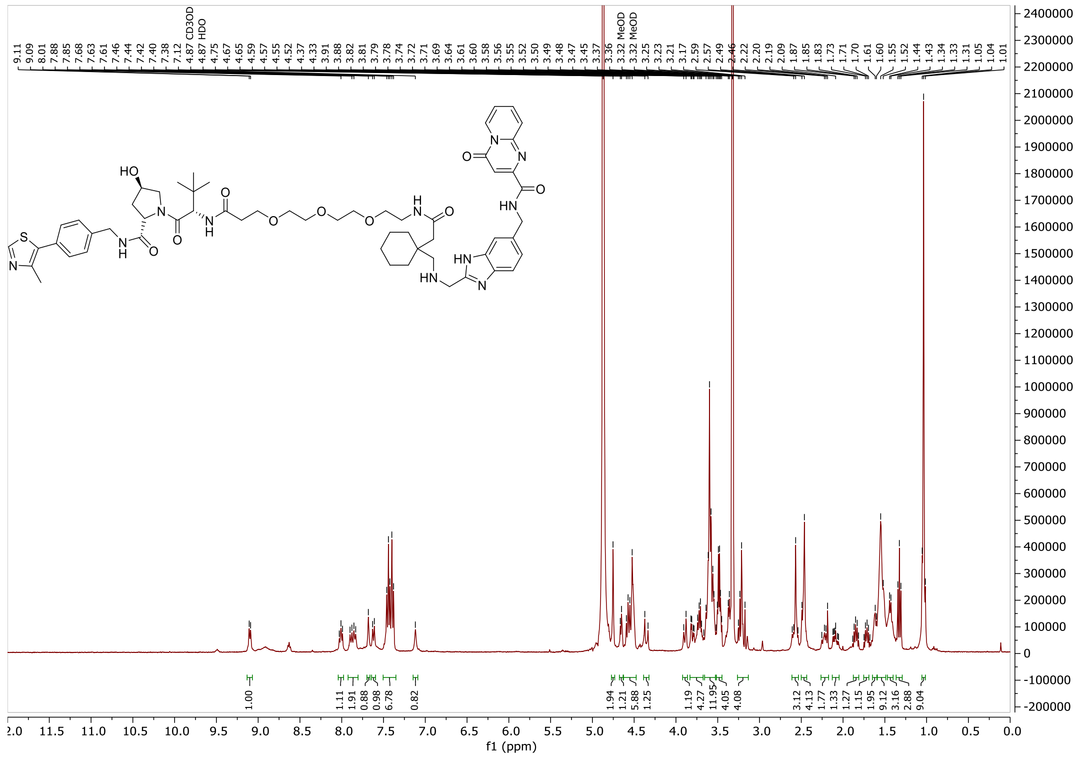

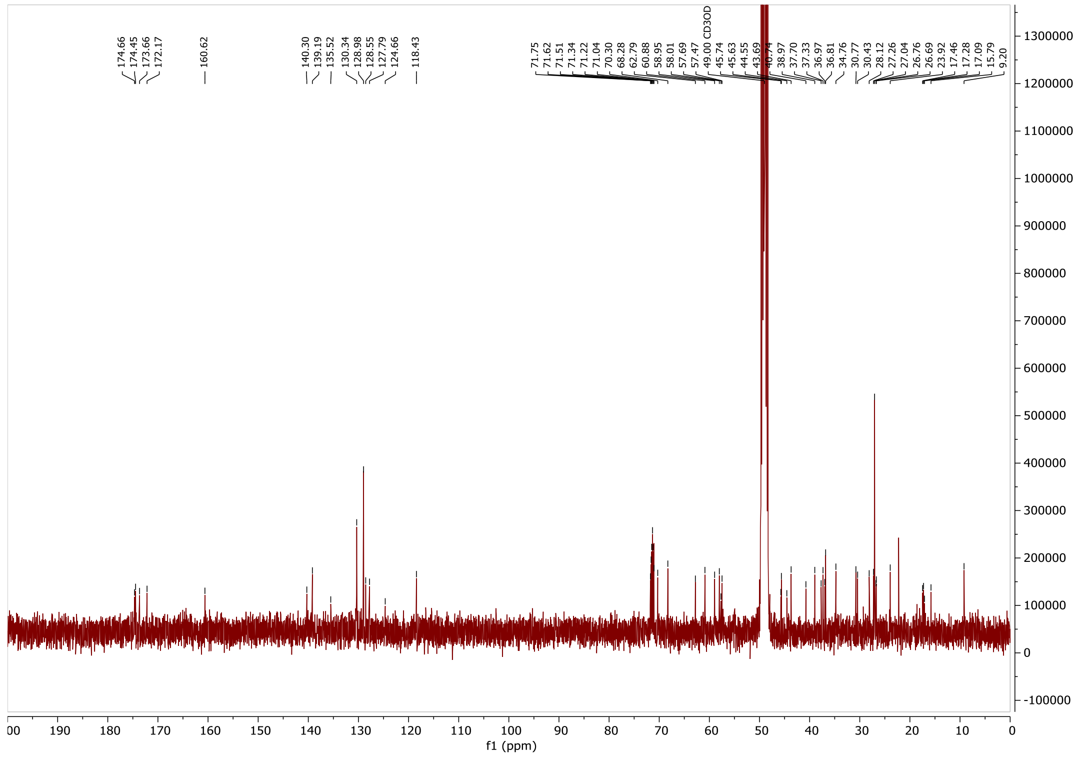

Compound **29**

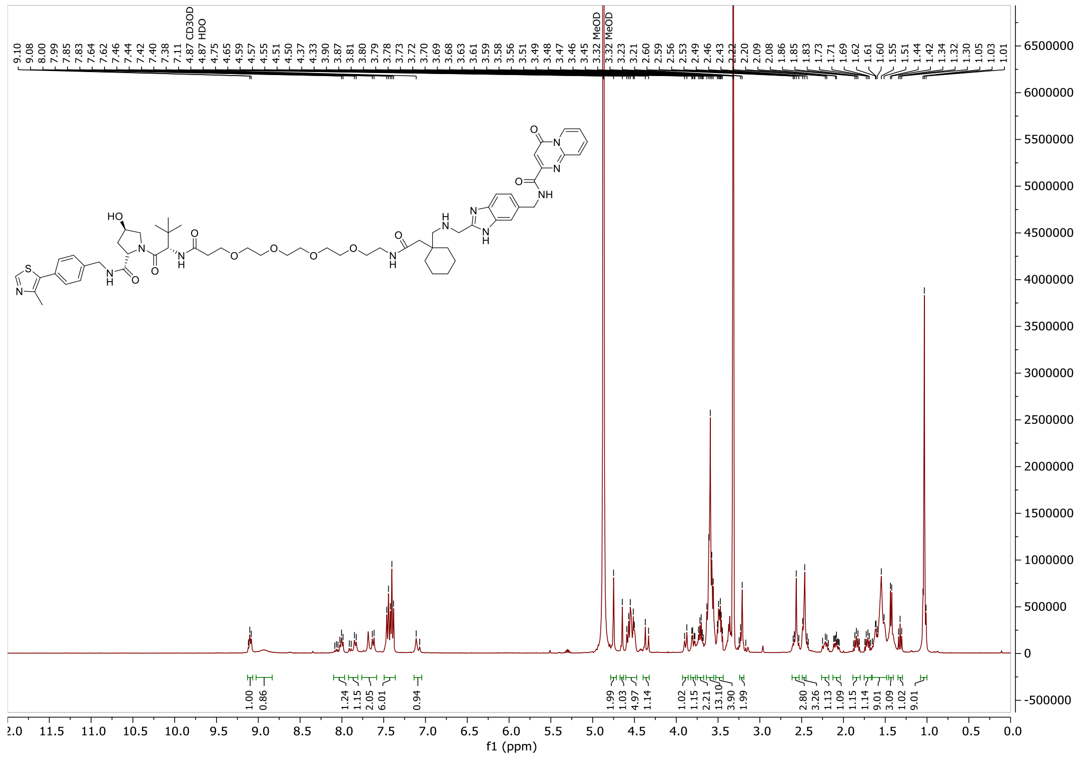

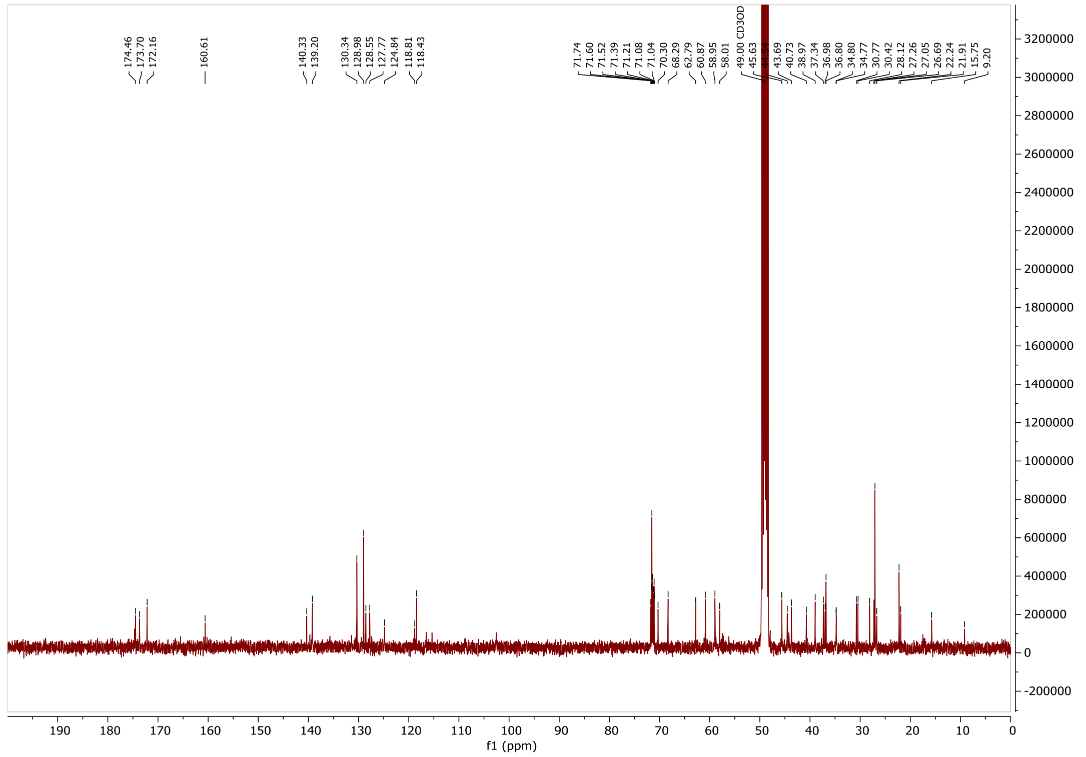

Compound **30**

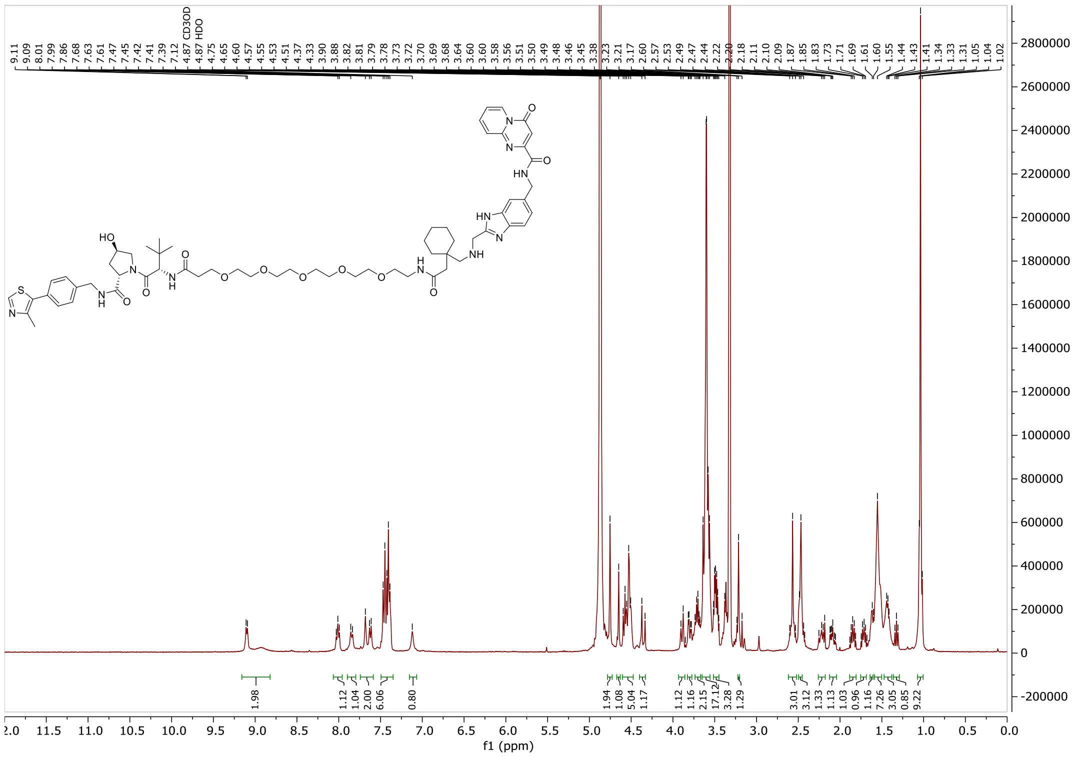

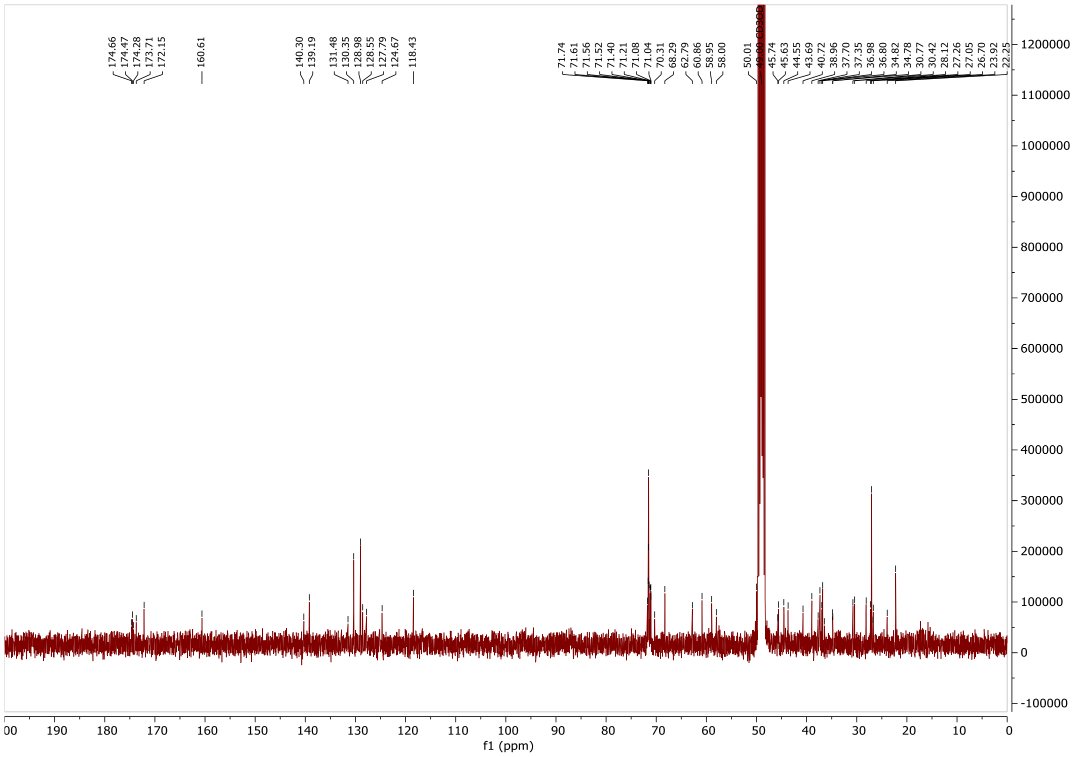

Compound **31**

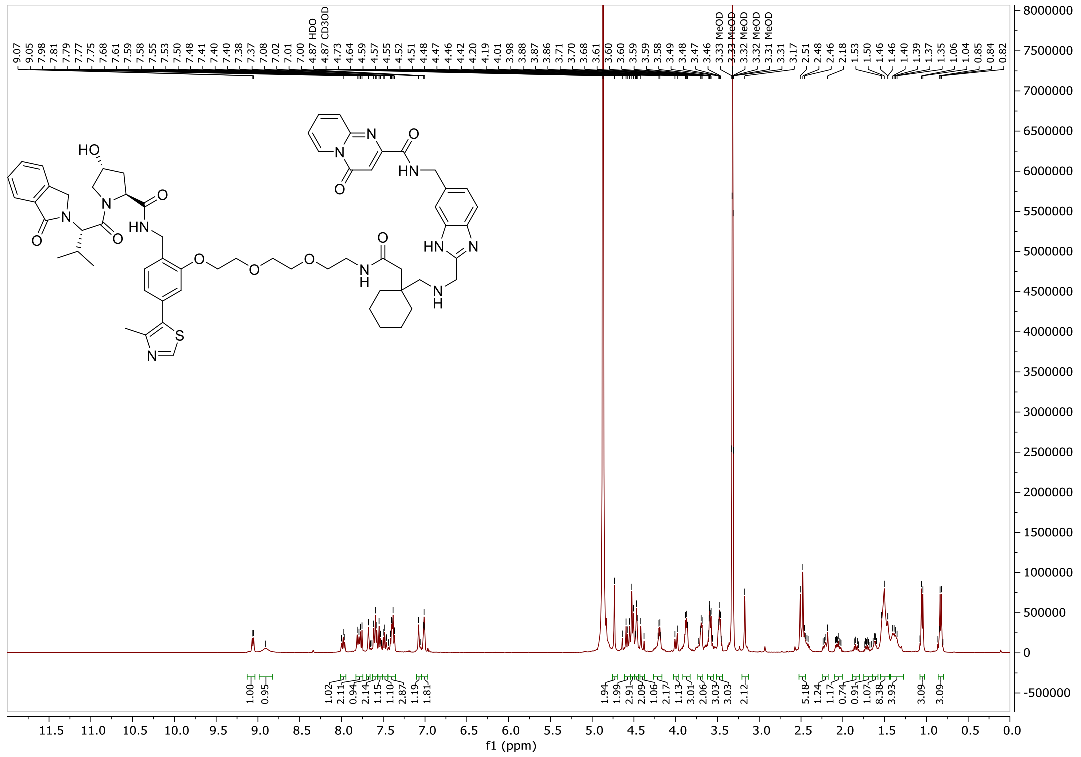

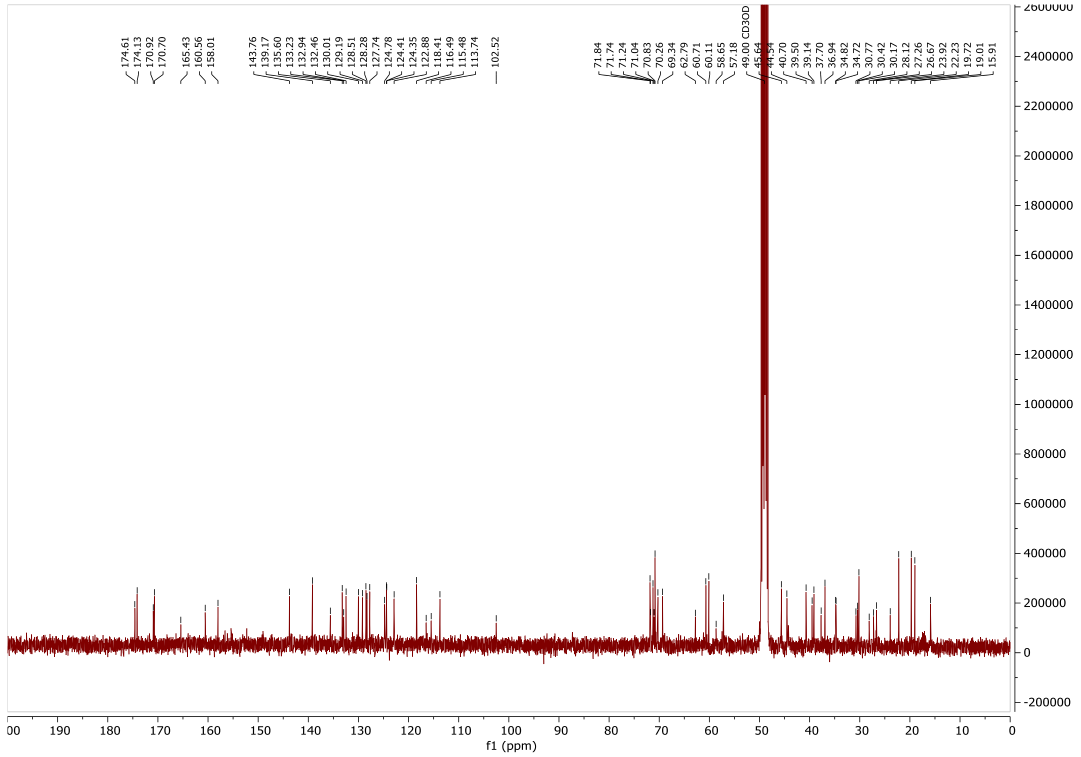

Compound **32**

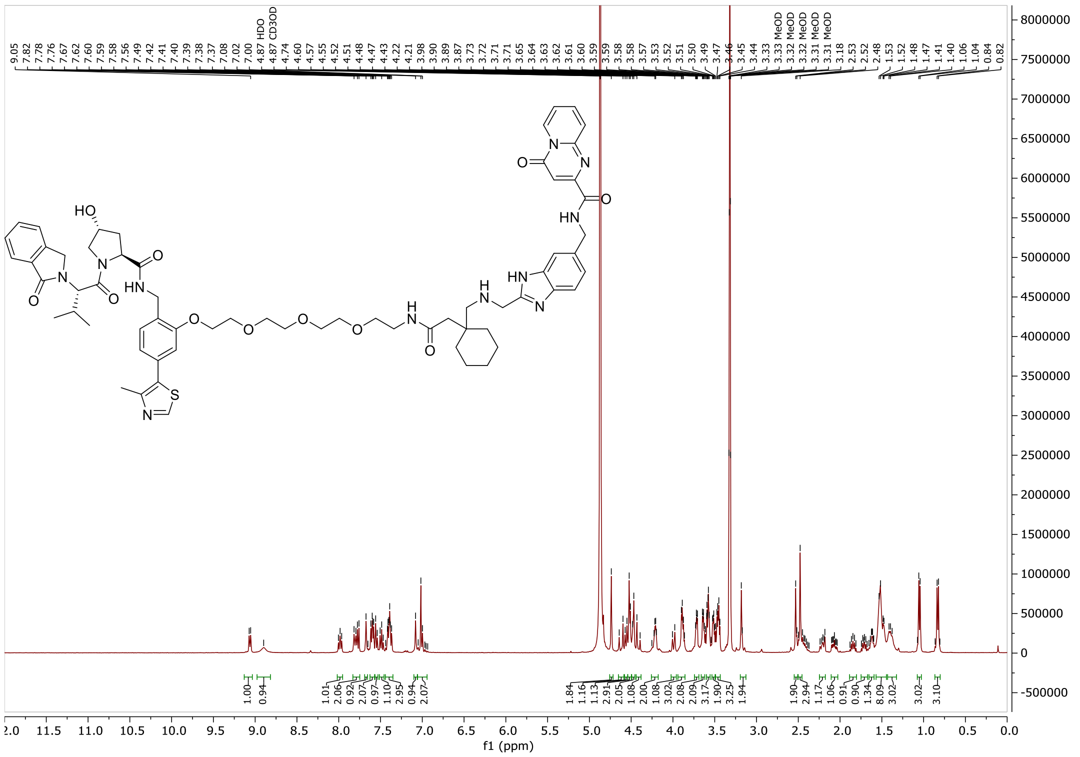
9.05 7.82 7.78 7.76 7.67 7.62 7.60 7.59 7.58 7.56 7.49 7.42 7.41 7.40 7.39 7.38 7.37 7.08 7.02 7.00 4.87 HDO 4.87 CD3OD 4.74 4.60 4.57 4.55 4.52 4.51 4.48 4.47 4.43 4.22 4.21 3.98 3.90 3.89 3.87 3.73 3.72 3.71 3.71 3.65 3.64 3.63 3.62 3.61 3.60 3.59 3.59 3.58 3.58 3.57 3.53 3.52 3.51 3.50 3.49 3.47 3.46 3.45 3.44 3.33 3.33 MeOD 3.32 MeOD 3.32 MeOD 3.31 MeOD 3.31 MeOD 3.18 2.53 2.52 2.48 1.53 1.52 1.48 1.47 1.41 1.40 1.06 1.04 0.84 0.82
HO
O
N
HN
S
N
H
NH
HN
f1 (ppm)

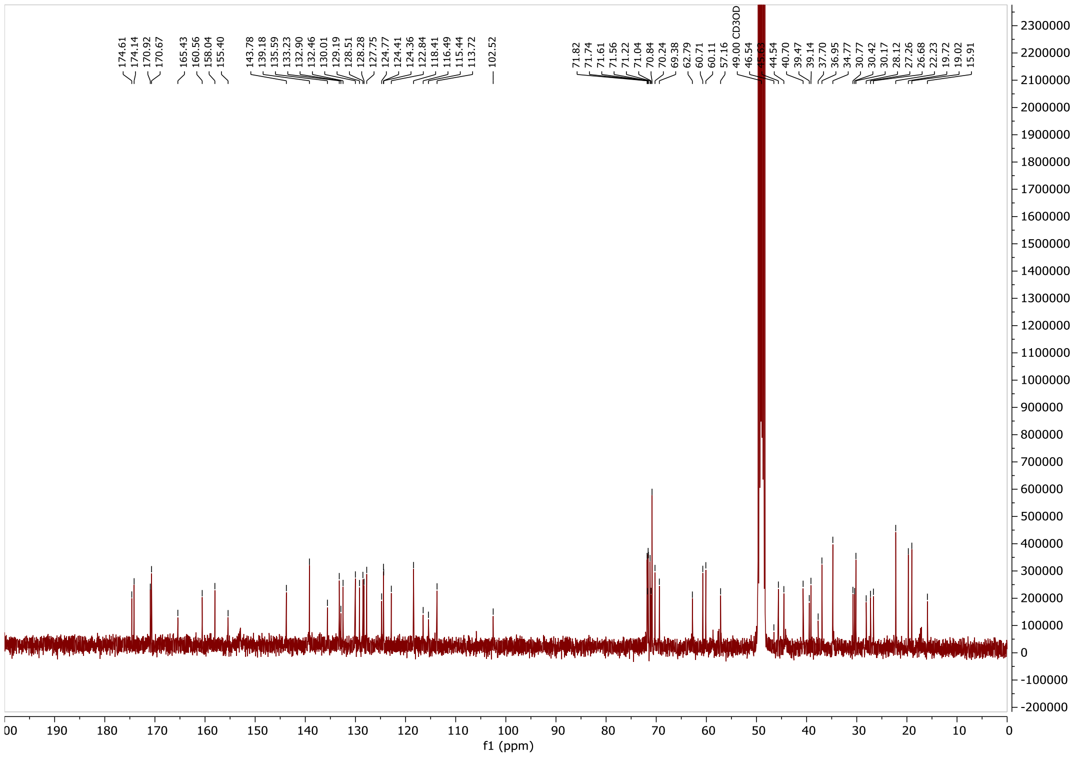
174.61 174.14 170.92 170.67 165.43 160.56 158.04 155.40 143.78 139.18 135.59 133.23 132.90 132.46 130.01 129.19 128.51 128.28 127.75 124.77 124.41 124.36 122.84 118.41 116.49 115.44 113.72 102.52 71.82 71.74 71.61 71.56 71.22 71.04 70.84 70.24 69.38 62.79 60.71 60.11 57.16 49.00 CD3OD 46.54 44.54 40.70 39.47 39.14 37.70 36.95 34.77 30.77 30.42 30.17 28.12 27.26 26.68 22.23 19.72 19.02 15.91
f1 (ppm)

Compound **33**

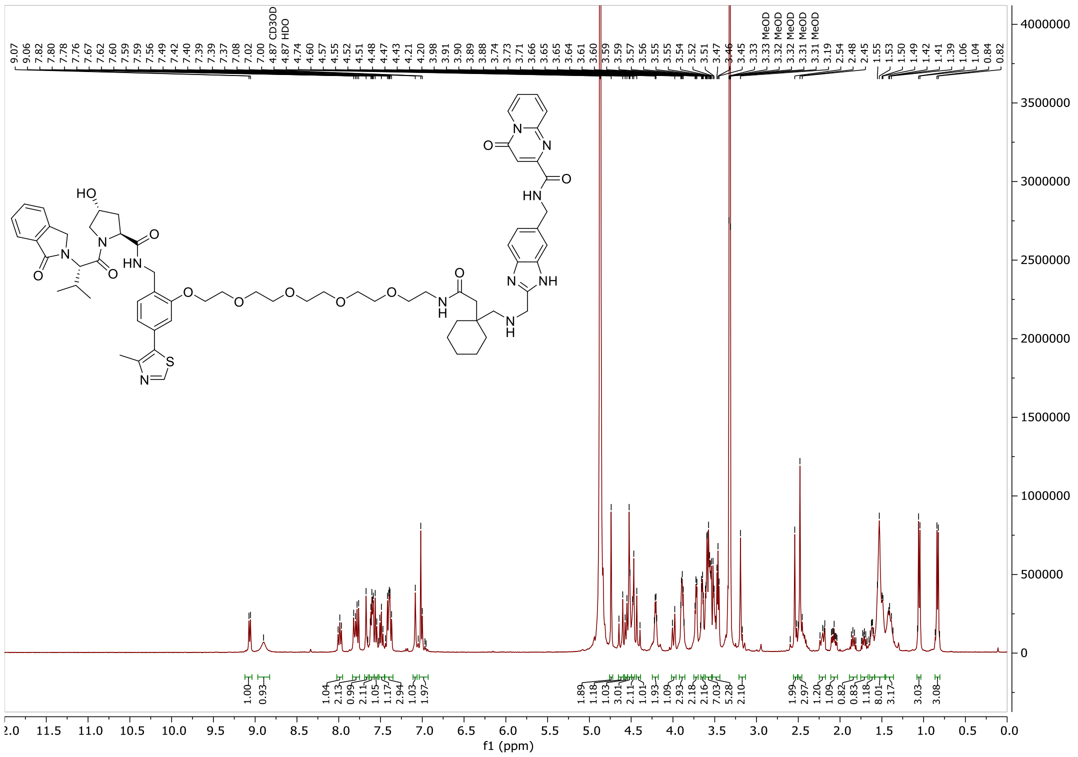

9.07 9.06 7.82 7.80 7.78 7.76 7.67 7.62 7.60 7.59 7.59 7.56 7.49 7.42 7.40 7.39 7.39 7.37 7.08 7.02 7.00 4.87 CD3OD 4.87 HDO 4.74 4.60 4.57 4.55 4.52 4.51 4.48 4.47 4.43 4.21 4.20 3.98 3.91 3.90 3.89 3.88 3.74 3.73 3.71 3.66 3.65 3.65 3.64 3.61 3.60 3.59 3.59 3.57 3.56 3.55 3.55 3.54 3.52 3.51 3.47 3.46 3.45 3.33 3.33 MeOD 3.32 MeOD 3.32 MeOD 3.31 MeOD 3.31 MeOD 3.19 2.54 2.48 2.45 1.55 1.53 1.50 1.49 1.42 1.41 1.39 1.06 1.04 0.84 0.82
1.00 0.93 1.04 2.13 0.99 2.11 1.05 1.17 2.94 1.03 1.97 1.89 1.18 3.01 1.03 2.11 1.01 1.93 1.09 2.93 2.18 2.16 7.03 5.28 2.10 1.99 2.97 1.20 1.09 0.82 0.83 1.18 8.01 3.17 3.03 3.08
f1 (ppm)

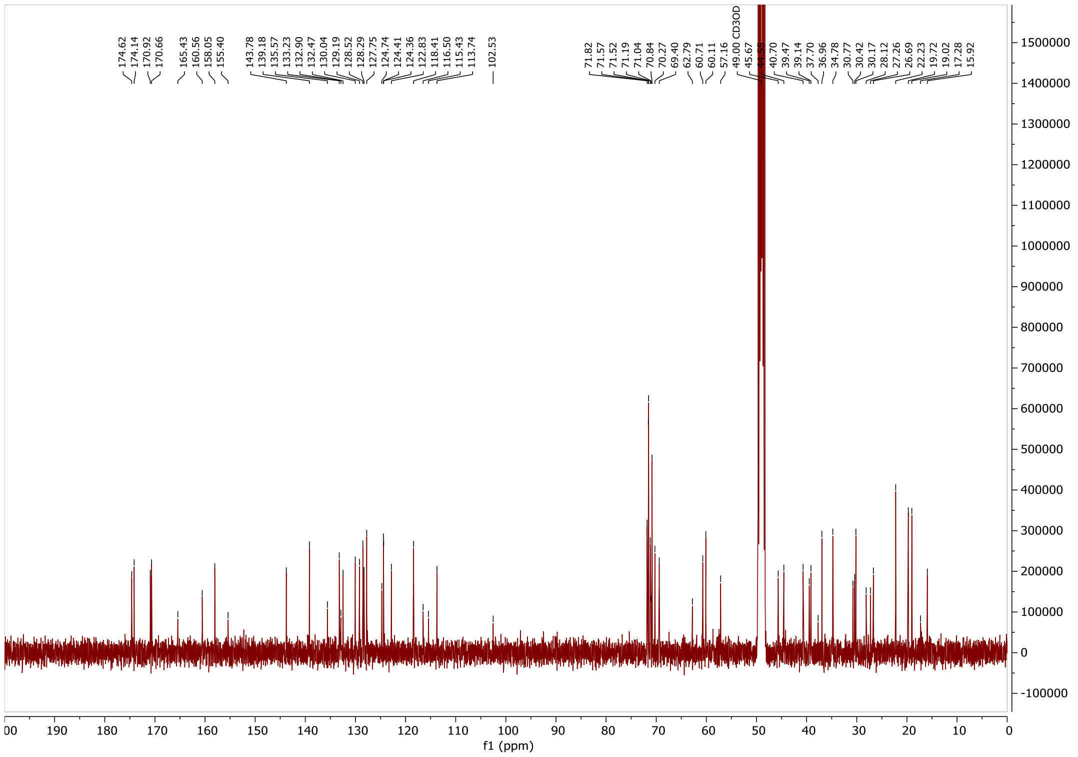

174.62 174.14 170.92 170.66 165.43 160.56 158.05 155.40 143.78 139.18 135.57 133.23 132.90 132.47 130.04 129.19 128.52 128.29 127.75 124.74 124.41 124.36 122.83 118.41 116.50 115.43 113.74 102.53 71.82 71.57 71.52 71.19 71.04 70.84 70.27 69.40 62.79 60.71 60.11 57.16 49.00 CD3OD 45.67 40.70 39.47 39.14 37.70 36.96 34.78 30.77 30.42 30.17 28.12 27.26 26.69 22.23 19.72 19.02 17.28 15.92
f1 (ppm)

Compound **34**

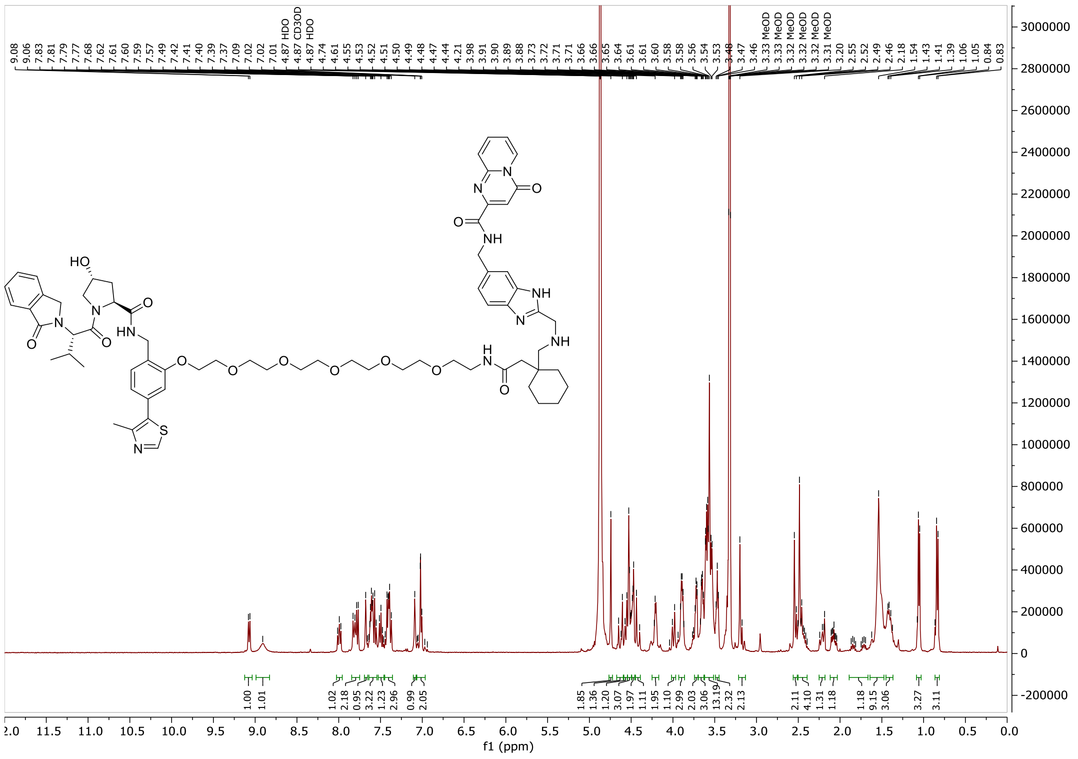

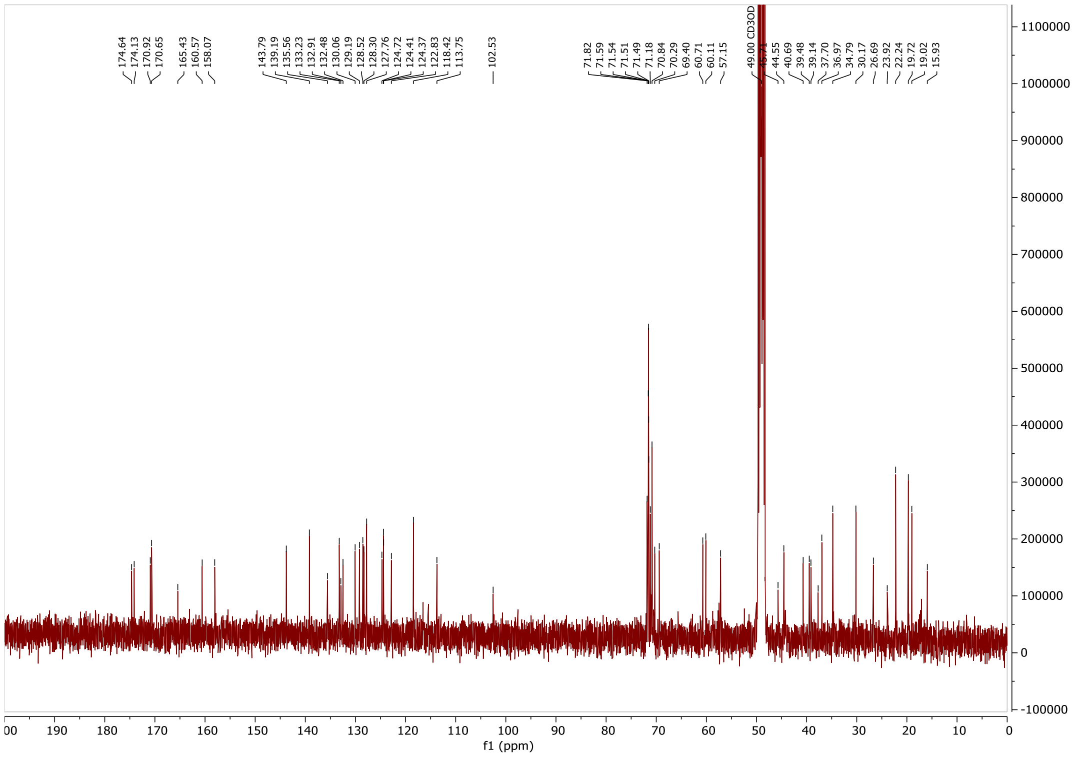

Compound **16**

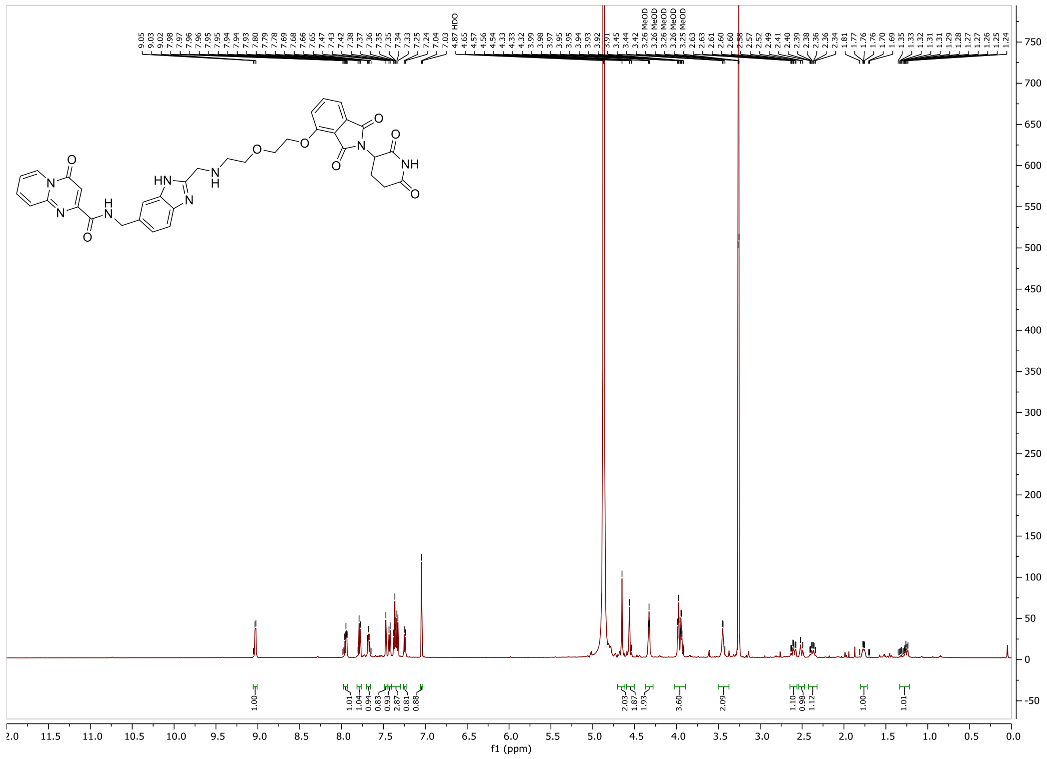
HDO
MeOD
1.00
1.01
1.04
0.94
0.83
0.93
2.87
0.81
0.88
2.03
1.87
1.93
3.60
2.09
1.10
0.98
1.12
1.00
1.01
f1 (ppm)

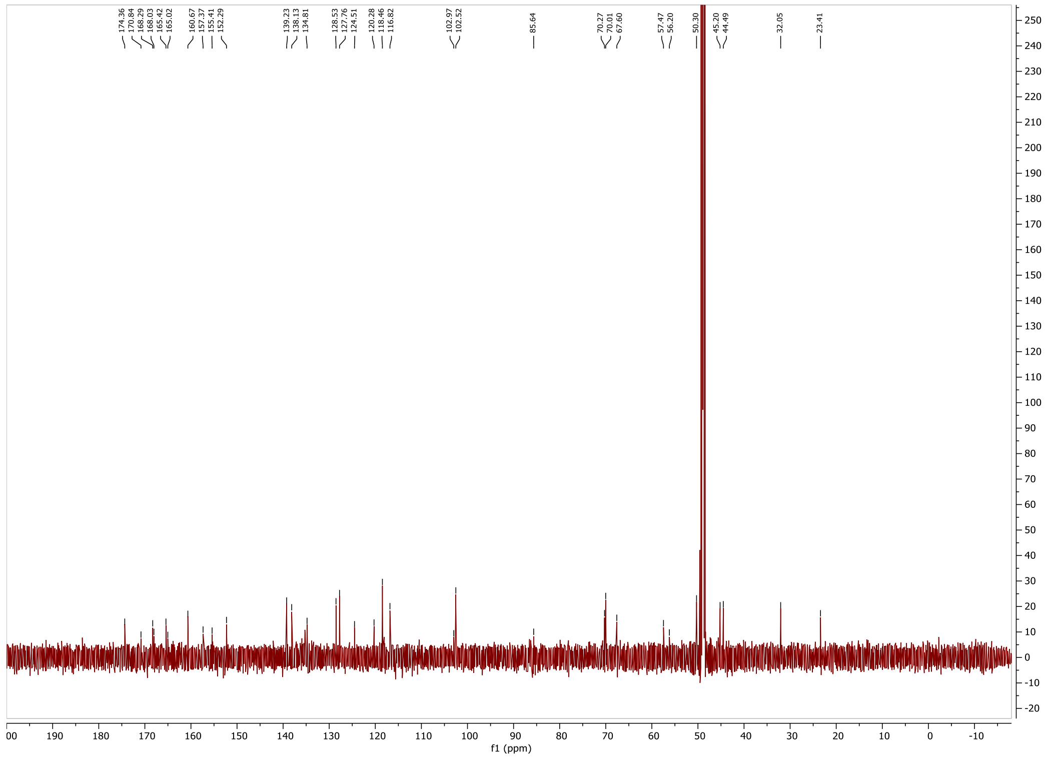
174.36
170.84
168.29
168.03
165.42
165.02
160.67
157.37
155.41
152.29
139.23
138.13
134.81
128.53
127.76
124.51
120.28
118.46
116.82
102.97
102.52
85.64
70.27
70.01
67.60
57.47
56.20
50.30
45.20
44.49
32.05
23.41
f1 (ppm)

Compound **21**

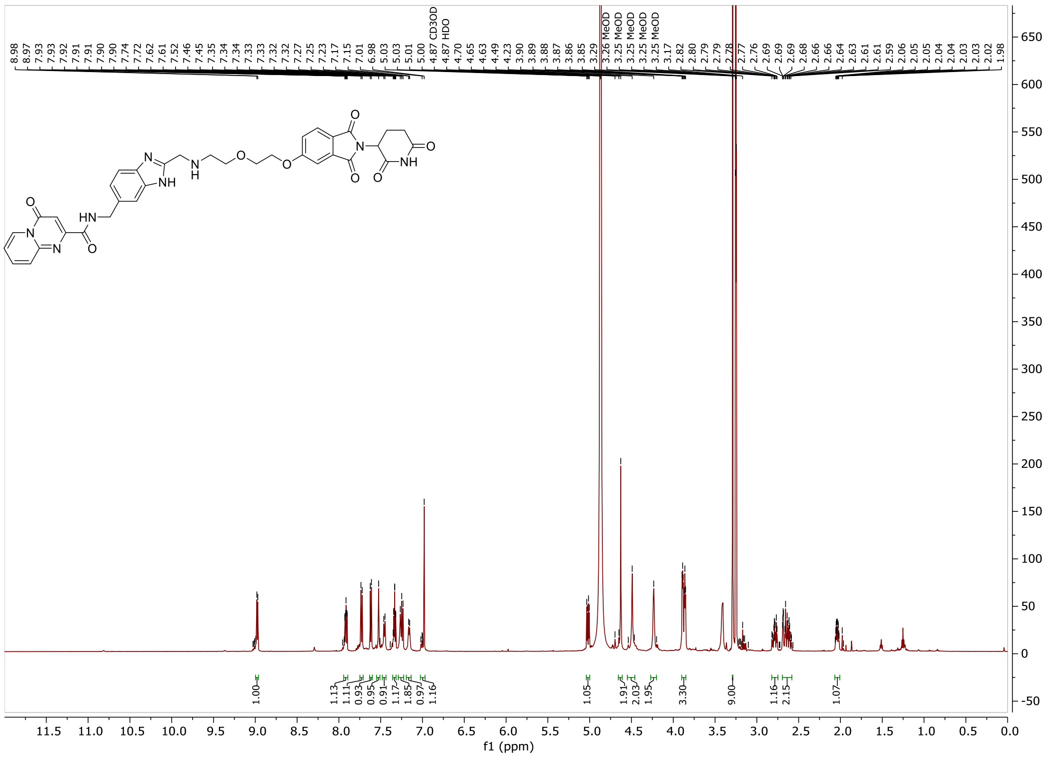

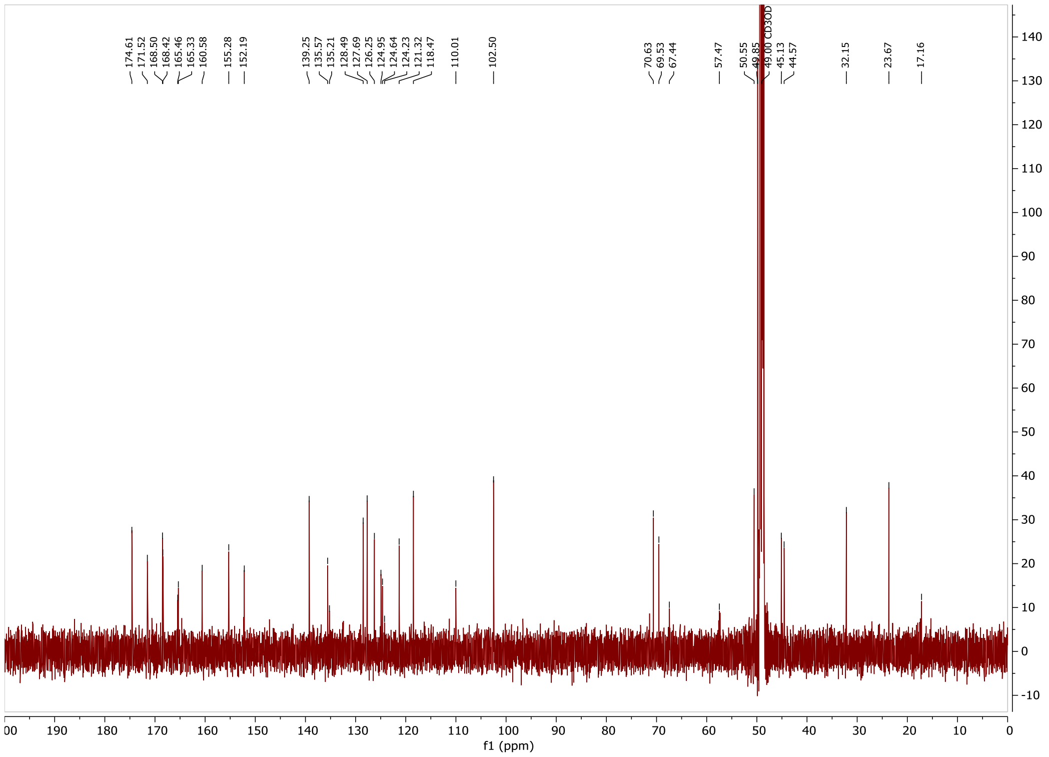

Compound **22**

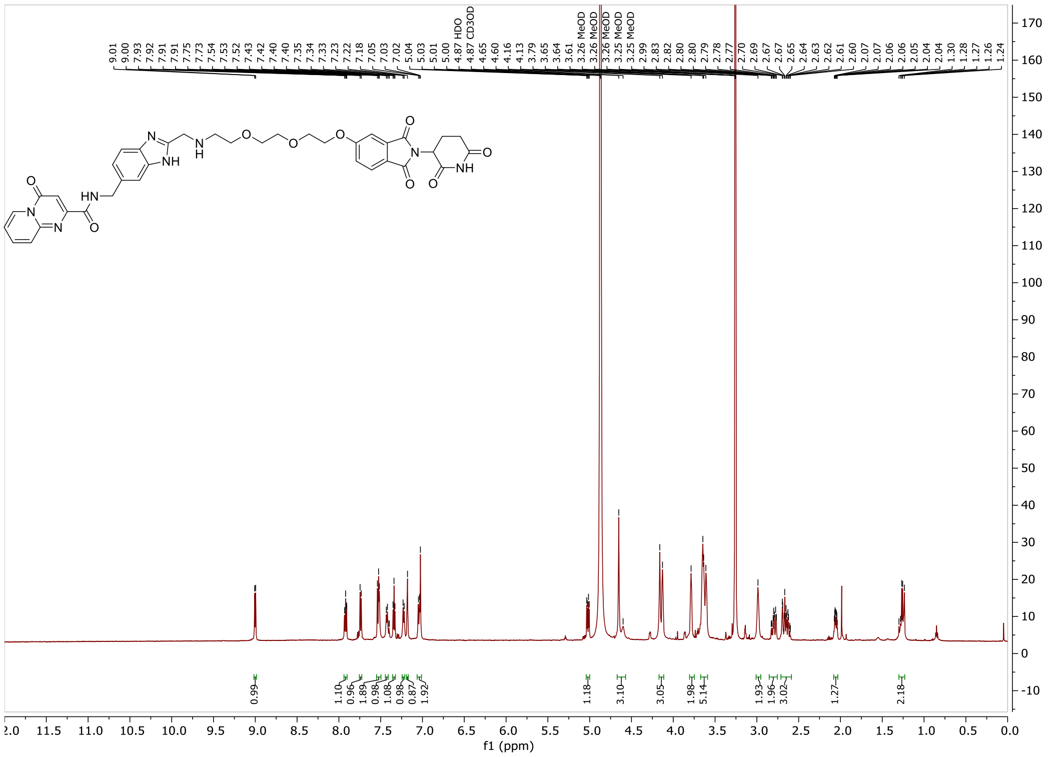

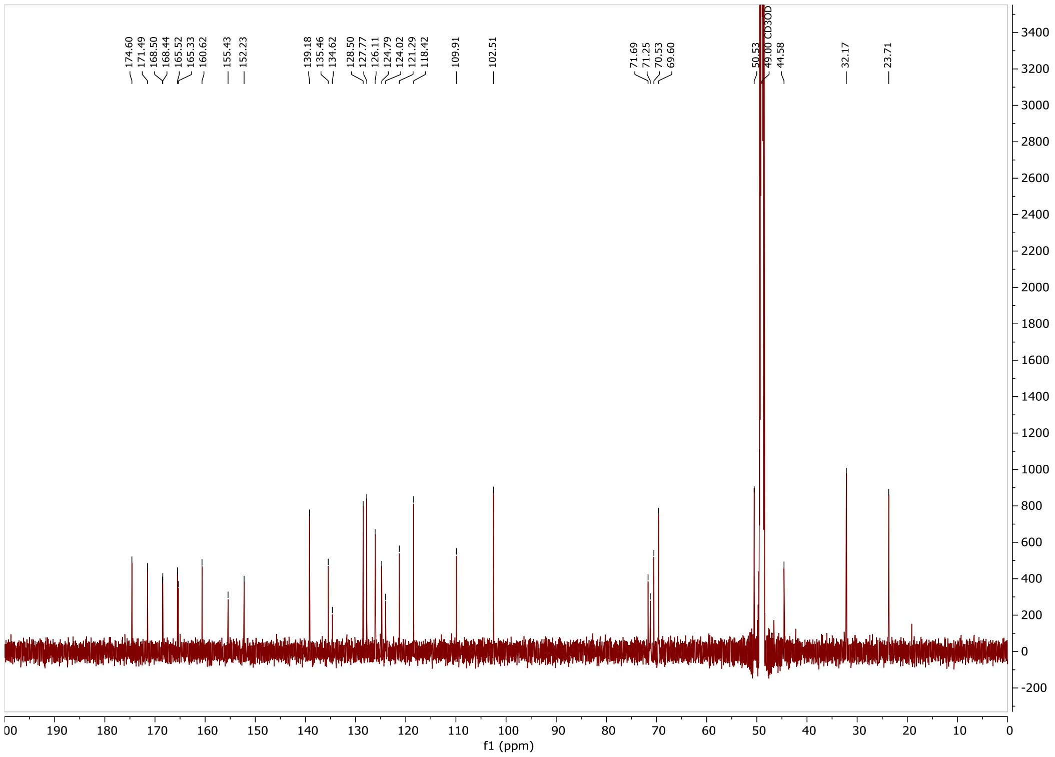

Compound **23**

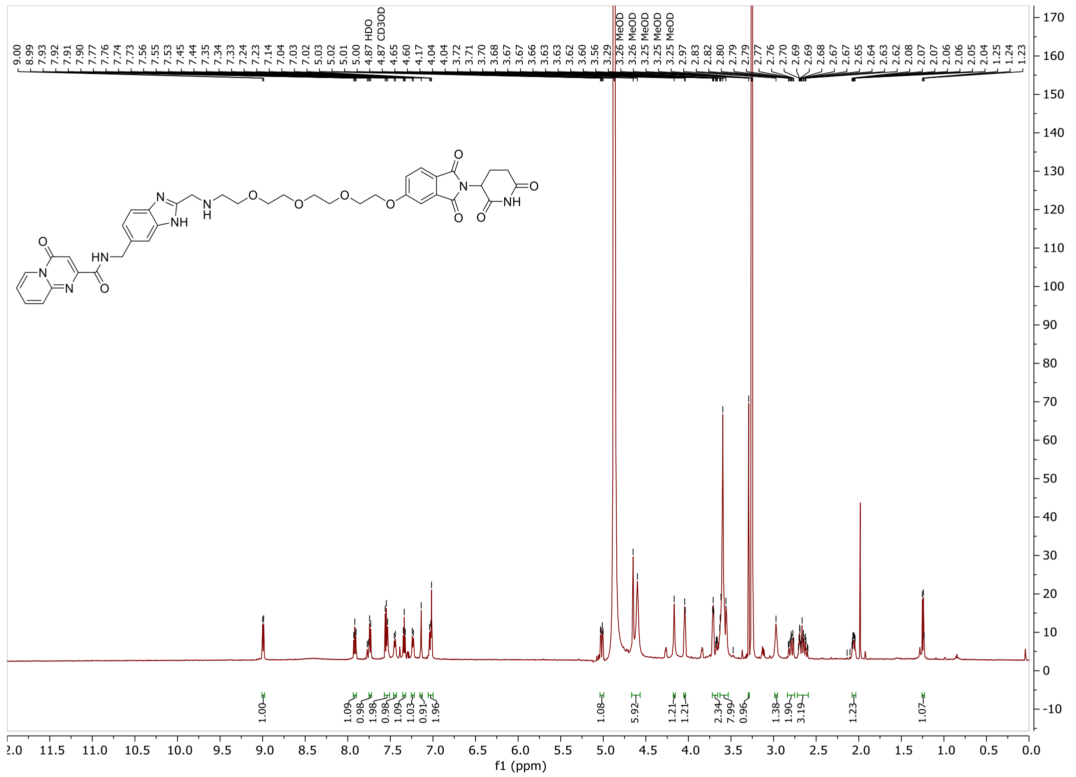

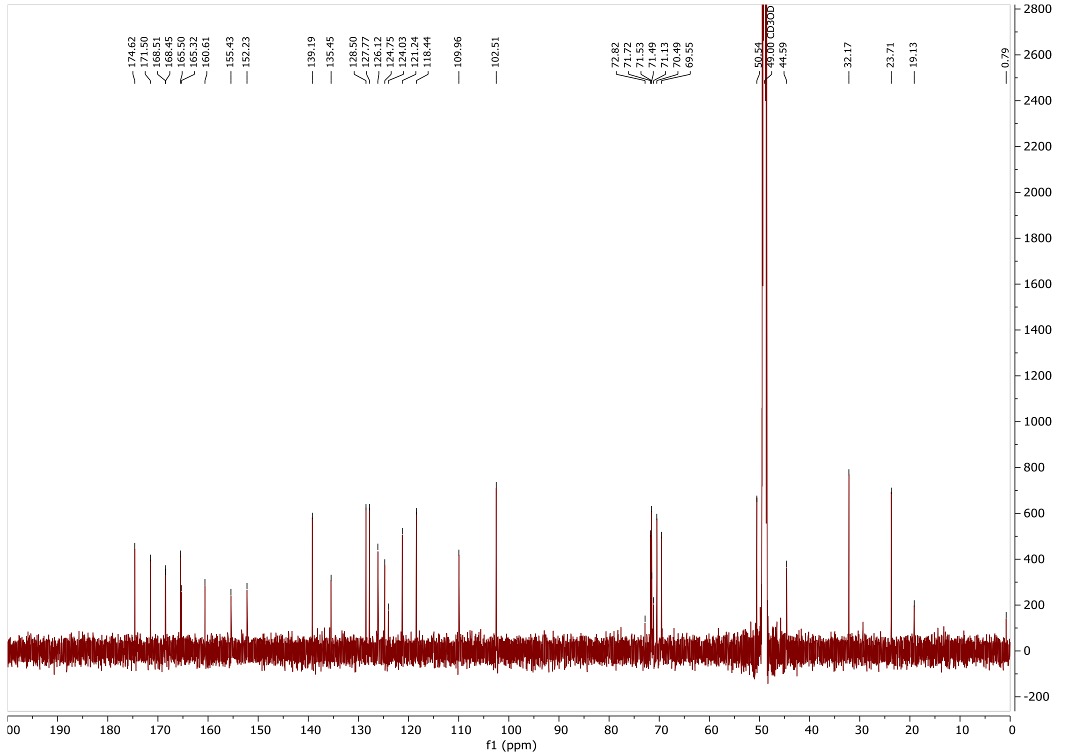

Compound **24**

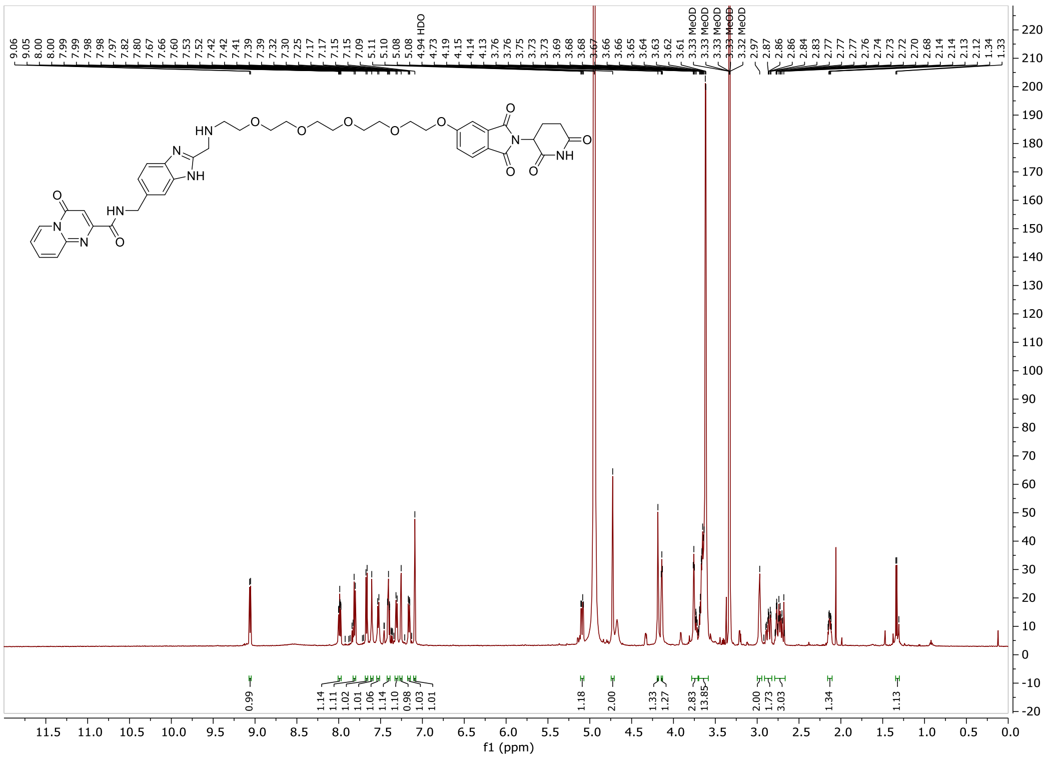

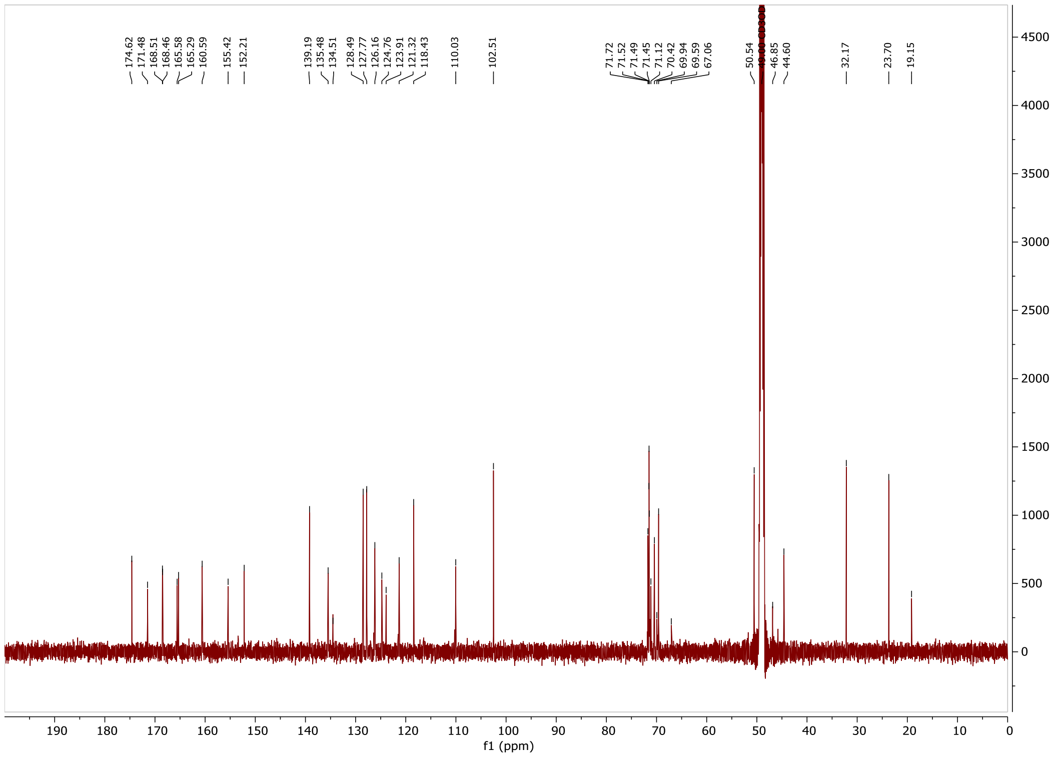

Compound **25**

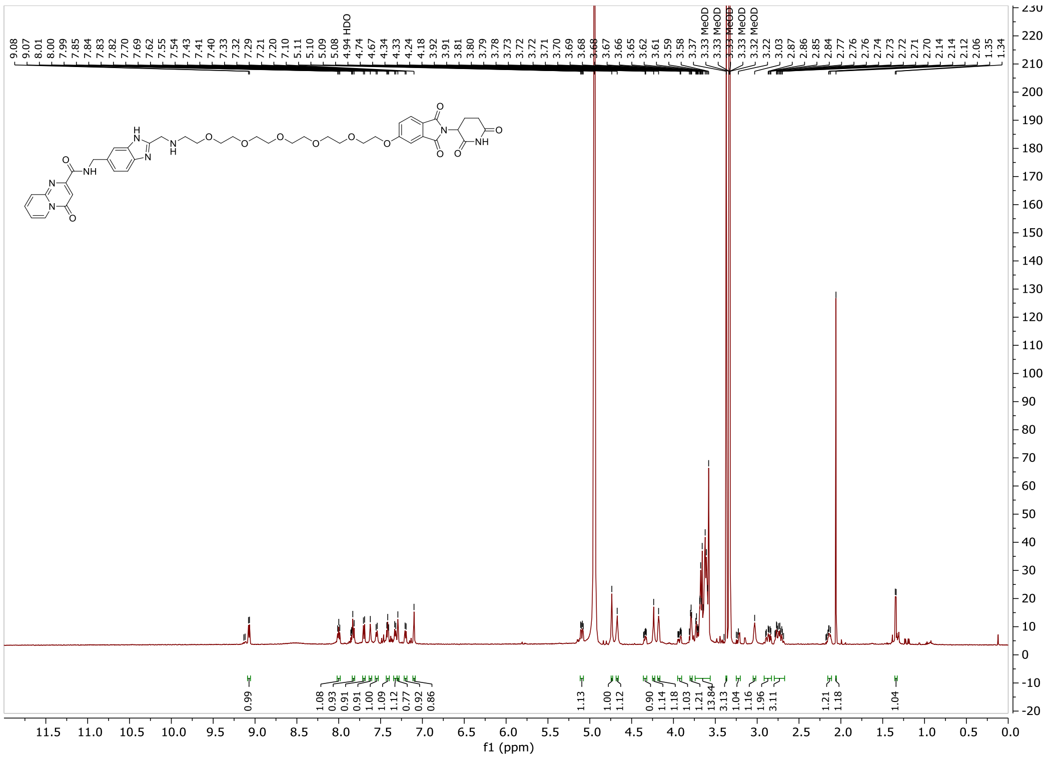
9.08 9.07 8.01 8.00 7.99 7.85 7.84 7.83 7.82 7.70 7.69 7.62 7.55 7.54 7.43 7.41 7.40 7.33 7.32 7.29 7.21 7.20 7.10 5.11 5.10 5.09 5.08 4.94 HDO 4.74 4.67 4.34 4.33 4.24 4.18 3.92 3.91 3.81 3.80 3.79 3.78 3.73 3.72 3.72 3.71 3.70 3.69 3.68 3.68 3.67 3.66 3.65 3.62 3.61 3.59 3.58 3.37 3.33 MeOD 3.33 MeOD 3.33 MeOD 3.33 MeOD 3.32 MeOD 3.22 3.03 2.87 2.86 2.85 2.84 2.77 2.76 2.76 2.74 2.73 2.72 2.71 2.70 2.14 2.12 2.06 1.35 1.34
0.99 1.08 0.93 0.91 0.91 1.00 1.09 1.12 0.77 0.92 0.86 1.13 1.00 1.12 0.90 1.14 1.18 1.03 1.21 13.84 3.13 1.04 1.16 1.96 3.11 1.21 1.18 1.04
f1 (ppm)

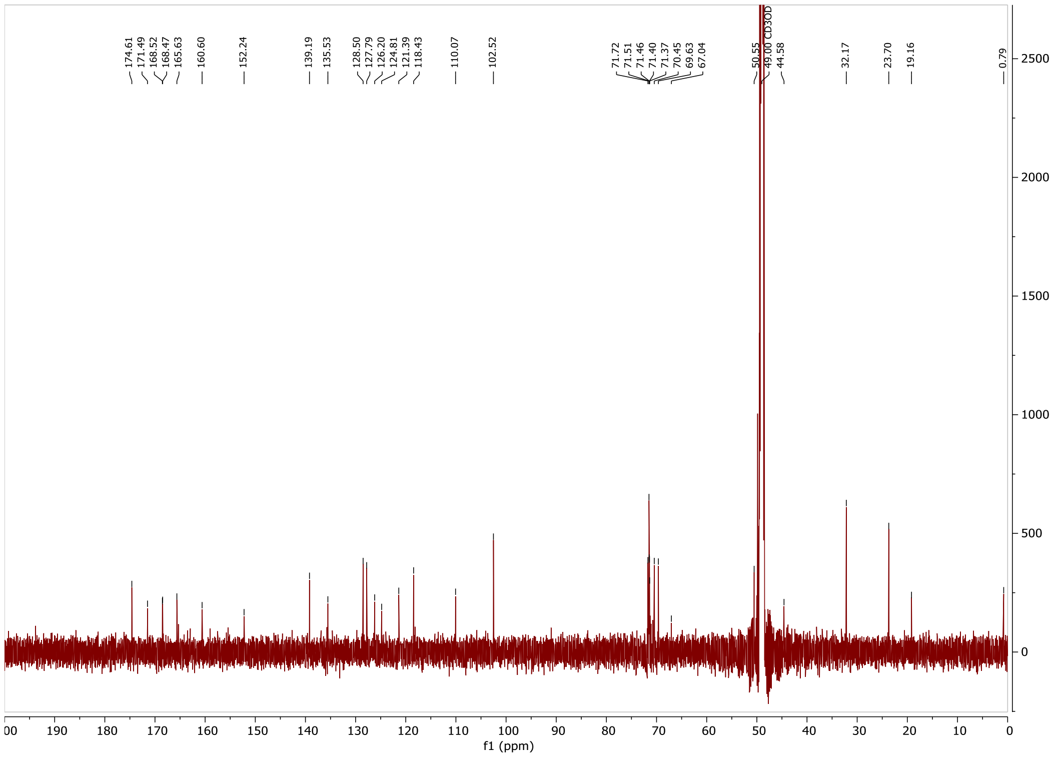
174.61 171.49 168.52 168.47 165.63 160.60 152.24 139.19 135.53 128.50 127.79 126.20 124.81 121.39 118.43 110.07 102.52 71.72 71.51 71.46 71.40 71.37 70.45 69.63 67.04 50.55 49.00 CD3OD 44.58 32.17 23.70 19.16 0.79
f1 (ppm)

Compound **17**

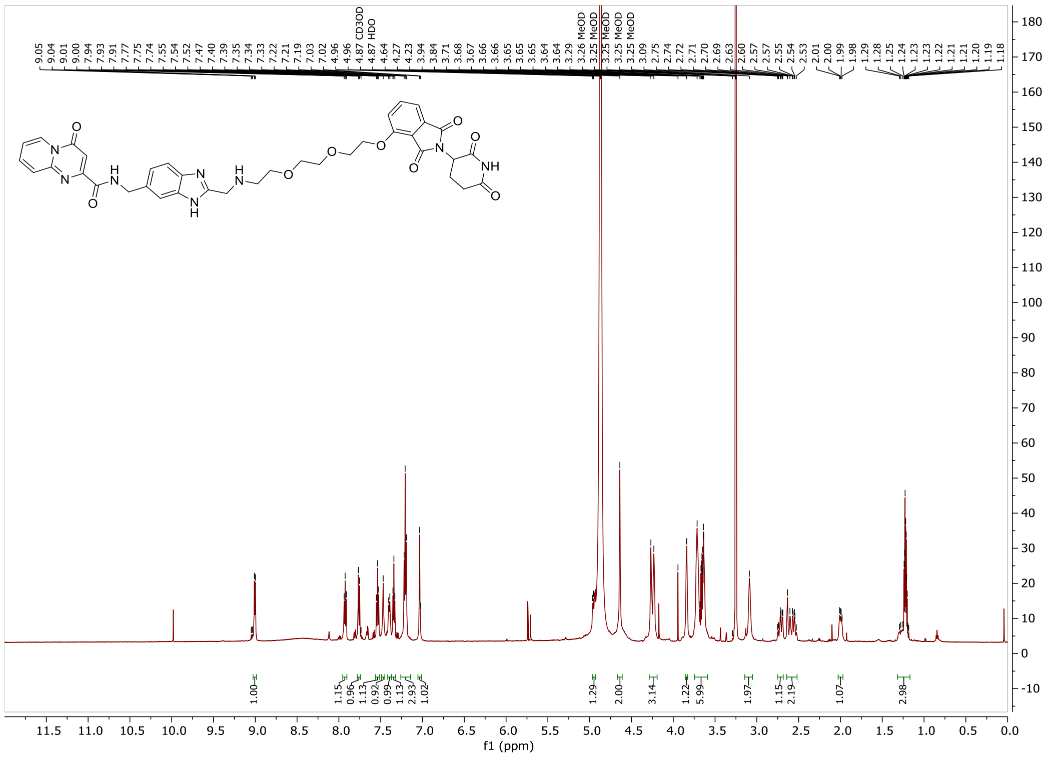

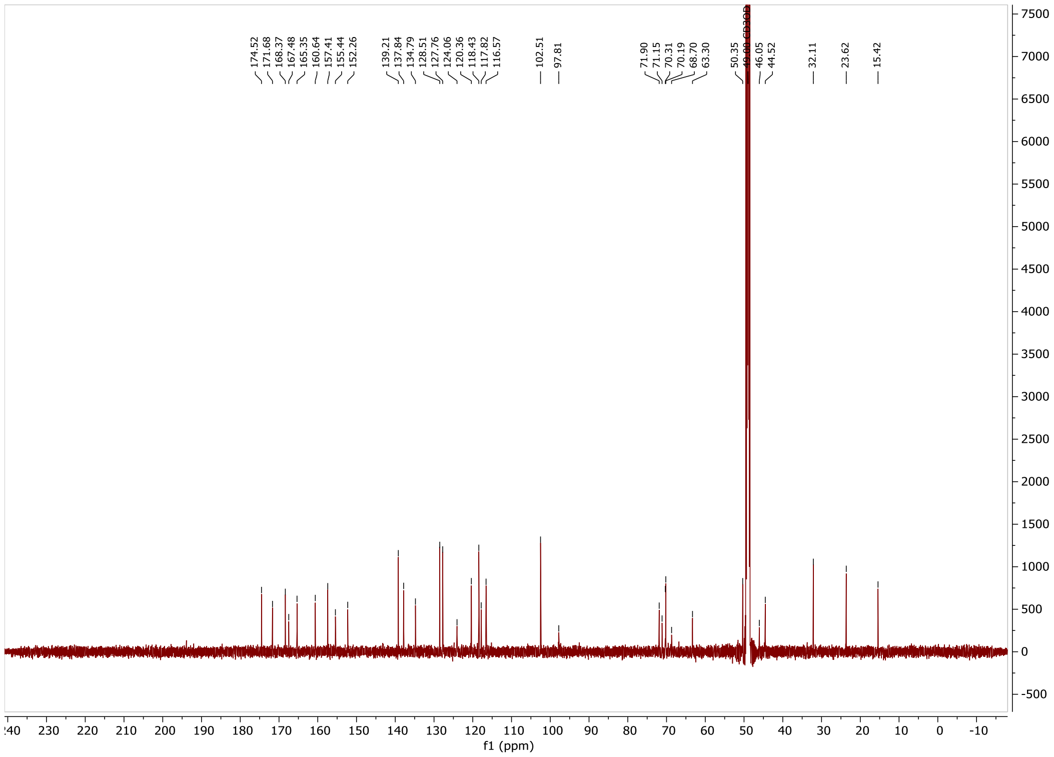

Compound **18**

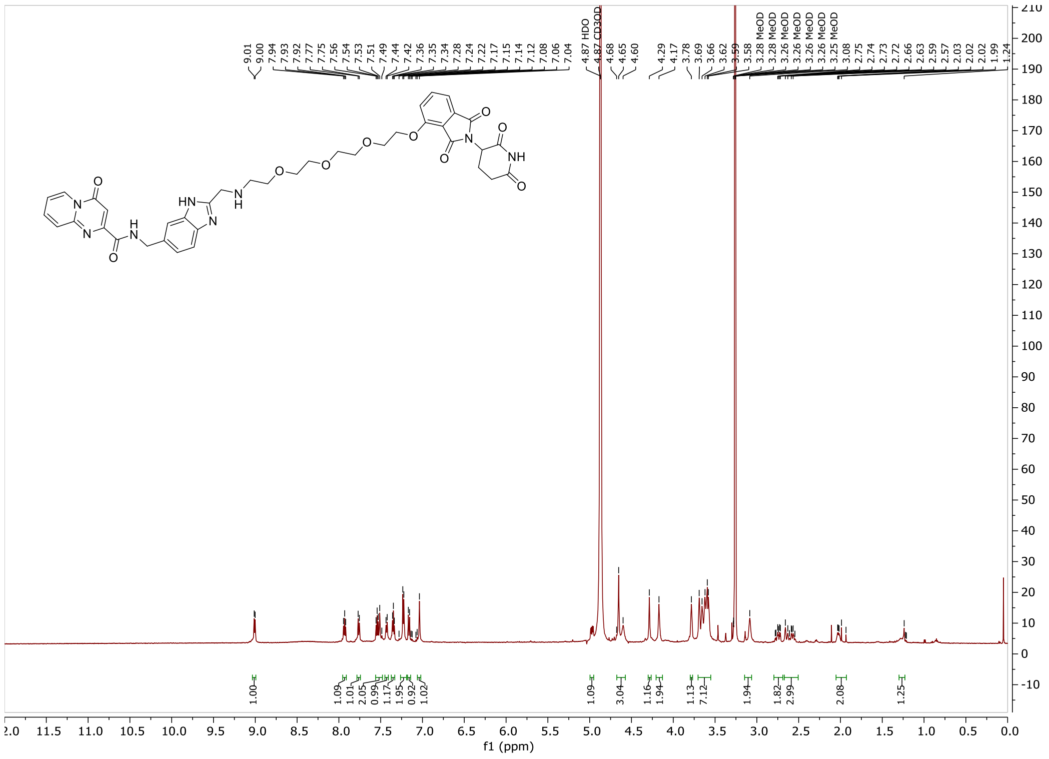
9.01
9.00
7.94
7.93
7.92
7.77
7.75
7.56
7.54
7.53
7.51
7.49
7.44
7.42
7.36
7.35
7.34
7.28
7.24
7.22
7.17
7.15
7.14
7.12
7.08
7.06
7.04
4.87 HDO
4.87 CD3OD
4.68
4.65
4.60
4.29
4.17
3.78
3.69
3.66
3.62
3.59
3.58
3.28 MeOD
3.28 MeOD
3.26 MeOD
3.26 MeOD
3.26 MeOD
3.26 MeOD
3.25 MeOD
3.08
2.75
2.74
2.73
2.72
2.66
2.63
2.59
2.57
2.03
2.02
2.02
1.99
1.24
1.00
1.09
1.01
2.05
0.99
1.17
1.95
0.92
1.02
1.09
3.04
1.16
1.94
1.13
7.12
1.94
1.82
2.99
2.08
1.25
O
N
N
H
N
HN
N
N
H
O
O
O
O
O
O
N
O
O
NH
O
f1 (ppm)

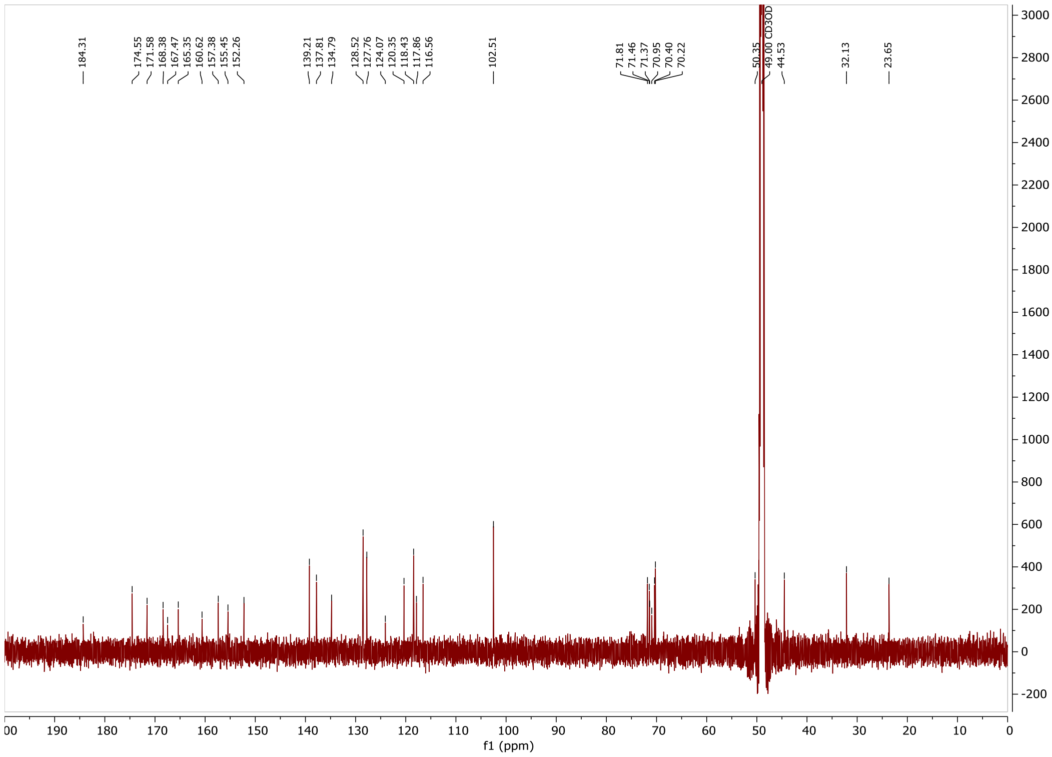
184.31
174.55
171.58
168.38
167.47
165.35
160.62
157.38
155.45
152.26
139.21
137.81
134.79
128.52
127.76
124.07
120.35
118.43
117.86
116.56
102.51
71.81
71.46
71.37
70.95
70.40
70.22
50.35
49.00 CD3OD
44.53
32.13
23.65
f1 (ppm)

Compound **19**

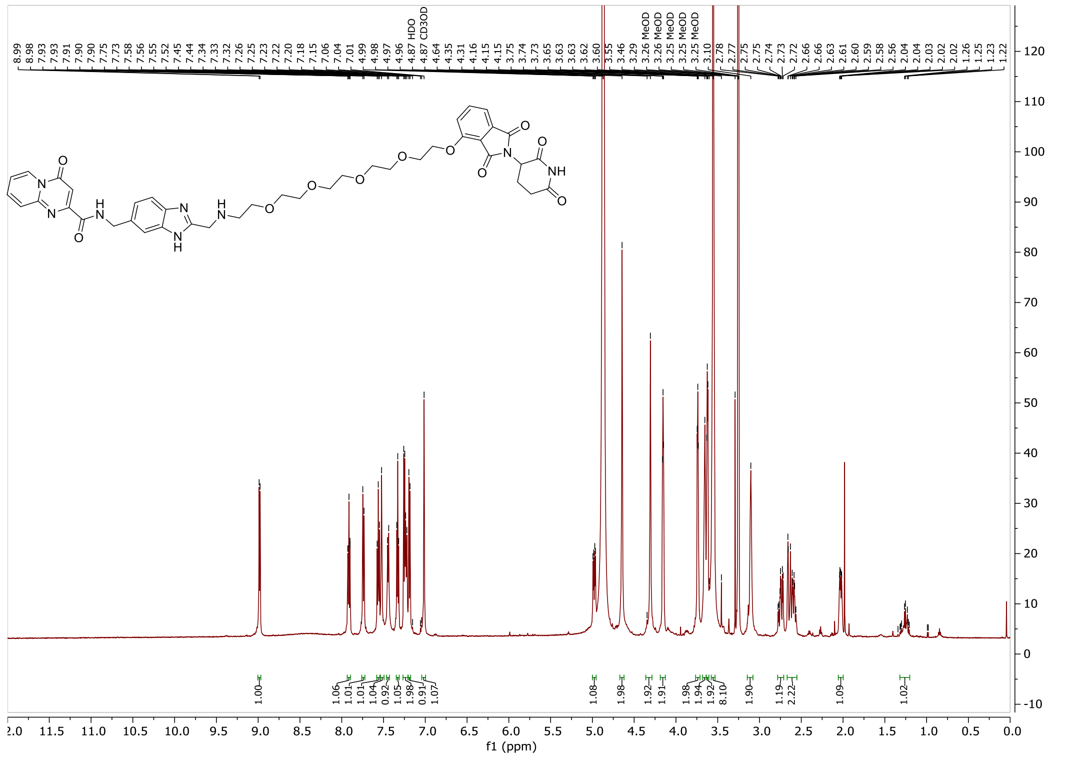

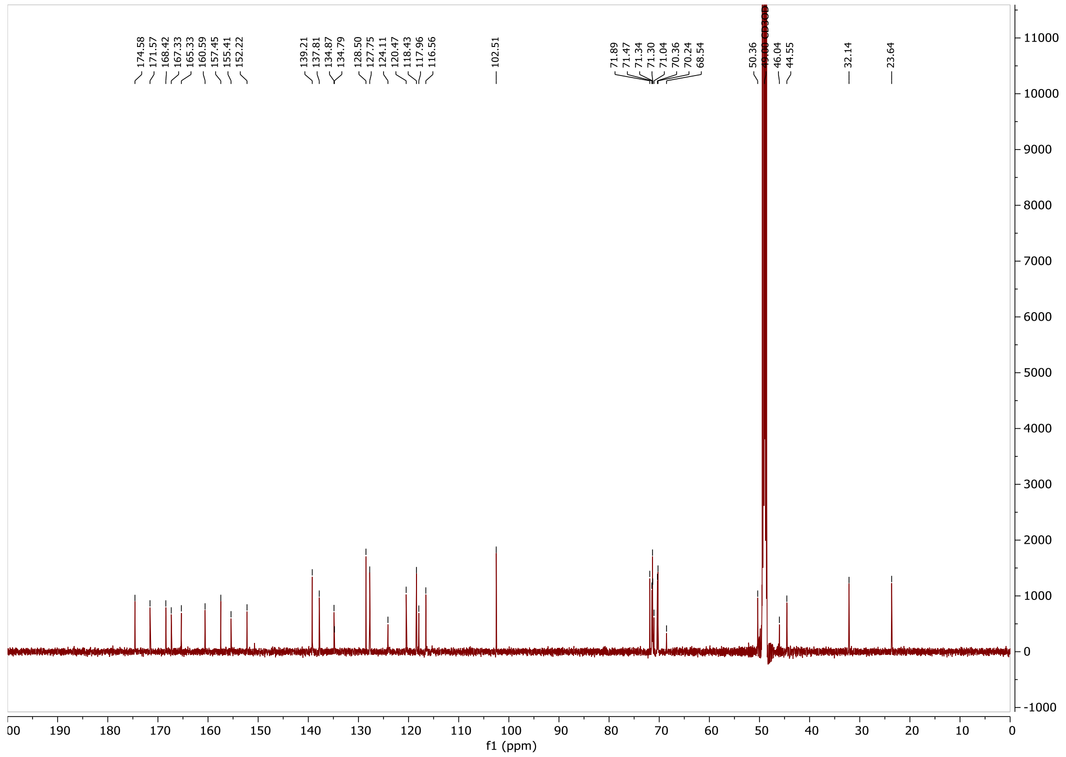

Compound **20**

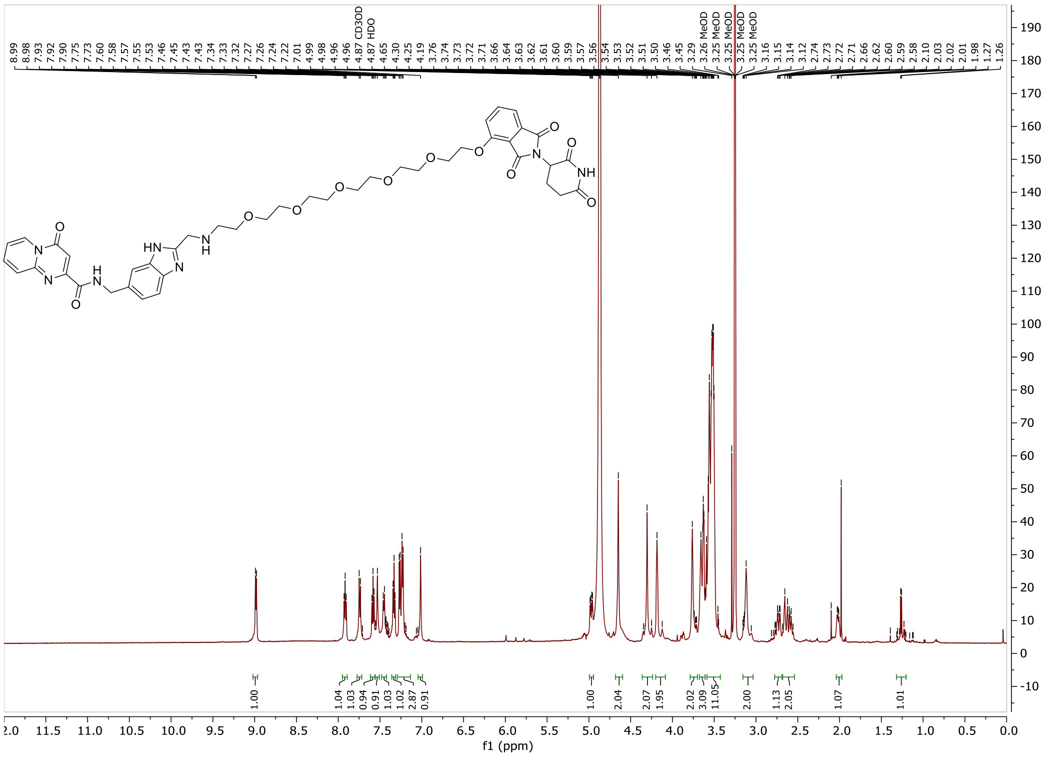
8.99
8.98
7.93
7.92
7.90
7.75
7.73
7.60
7.58
7.57
7.55
7.53
7.46
7.45
7.43
7.43
7.34
7.33
7.32
7.27
7.26
7.24
7.22
7.01
4.99
4.98
4.96
4.96
4.87 CD3OD
4.87 HDO
4.65
4.30
4.25
4.19
3.76
3.74
3.73
3.72
3.71
3.66
3.64
3.63
3.62
3.61
3.60
3.59
3.57
3.56
3.54
3.53
3.52
3.51
3.50
3.46
3.45
3.29
3.26 MeOD
3.25 MeOD
3.25 MeOD
3.25 MeOD
3.25 MeOD
3.16
3.15
3.14
3.12
2.74
2.73
2.72
2.71
2.66
2.62
2.60
2.59
2.58
2.10
2.03
2.02
2.01
1.98
1.27
1.26
1.00
1.04
1.03
0.94
0.91
1.03
1.02
2.87
0.91
1.00
2.04
2.07
1.95
2.02
3.09
11.05
2.00
1.13
2.05
1.07
1.01
f1 (ppm)

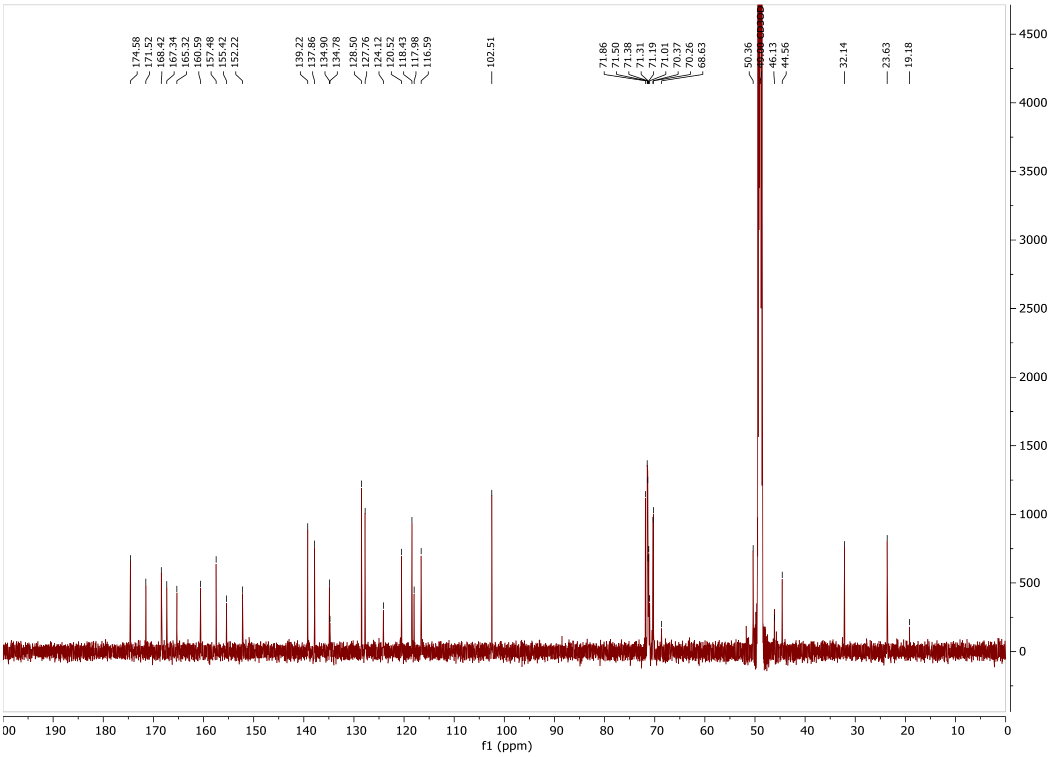
174.58
171.52
168.42
167.34
165.32
160.59
157.48
155.42
152.22
139.22
137.86
134.90
134.78
128.50
127.76
124.12
120.52
118.43
117.98
116.59
102.51
71.86
71.50
71.38
71.31
71.19
71.01
70.37
70.26
68.63
50.36
46.13
44.56
32.14
23.63
19.18
f1 (ppm)

Compound **6**

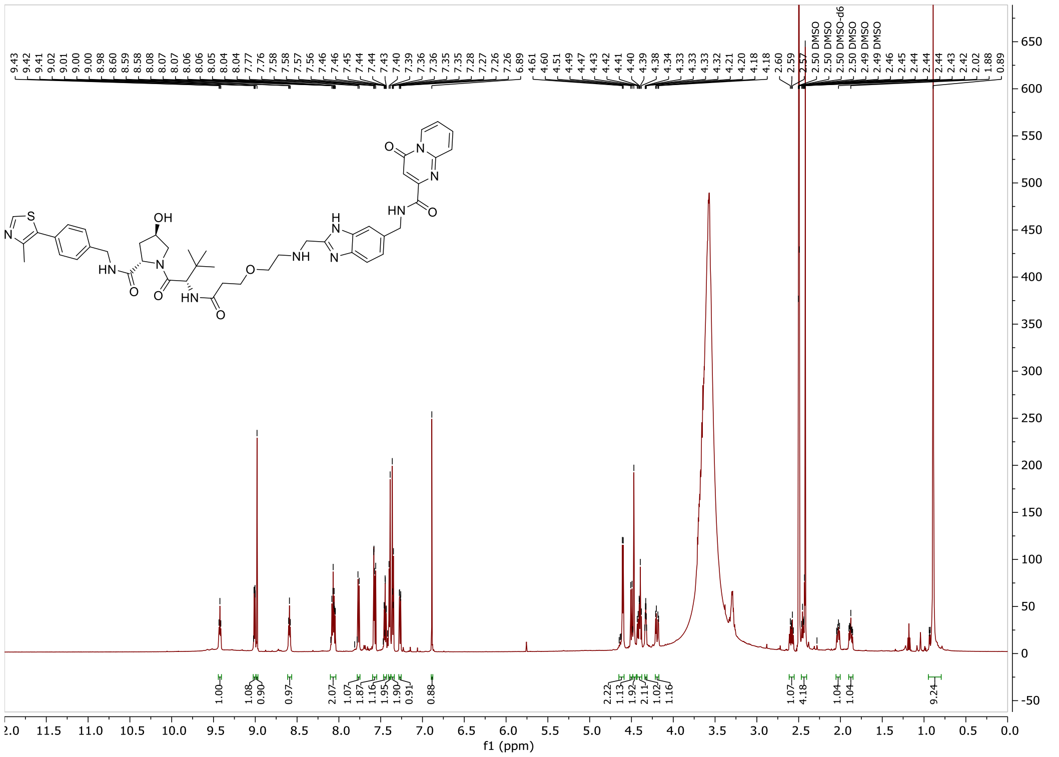

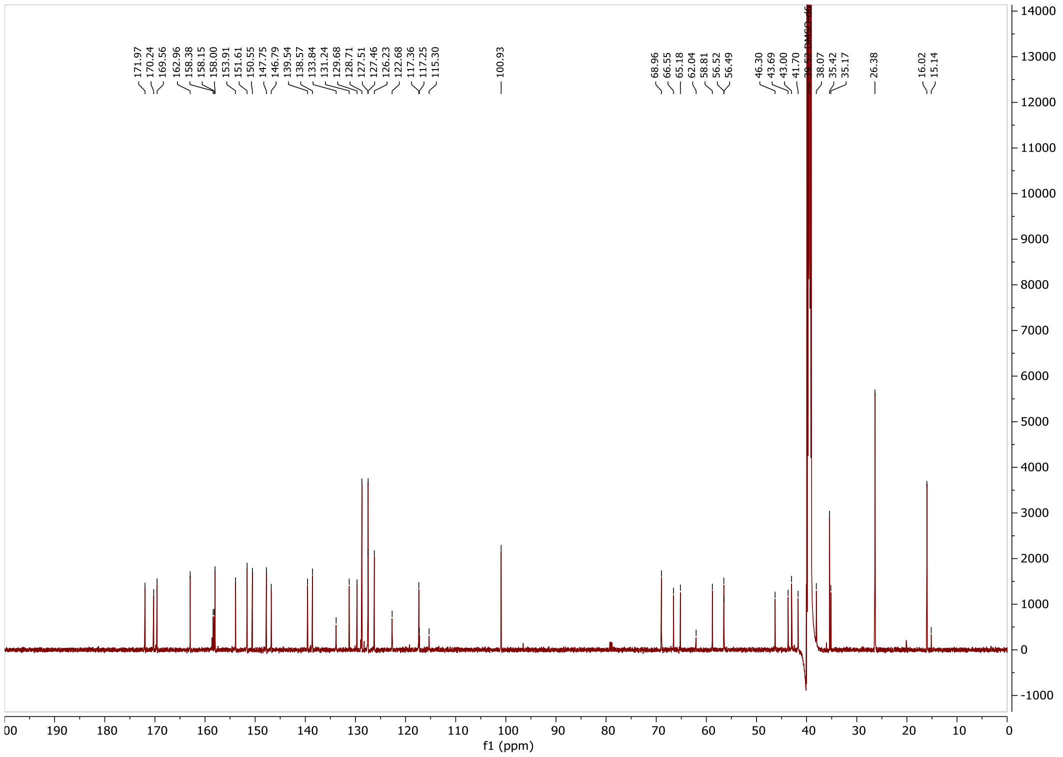

Compound **7**

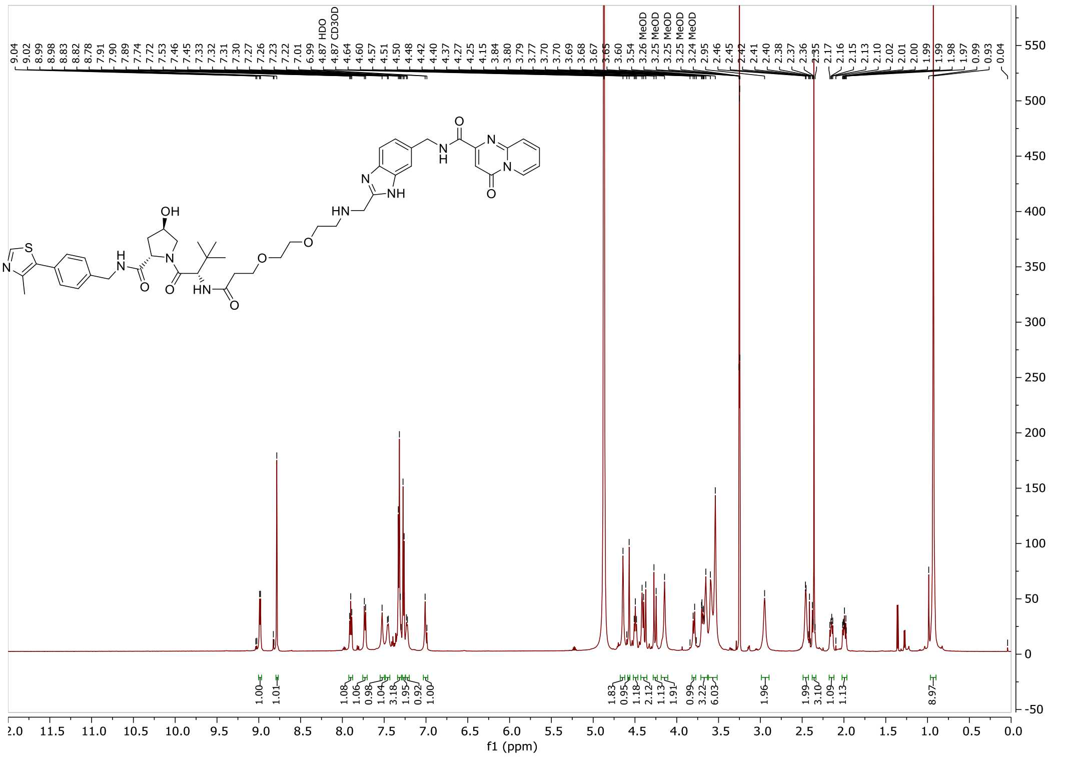

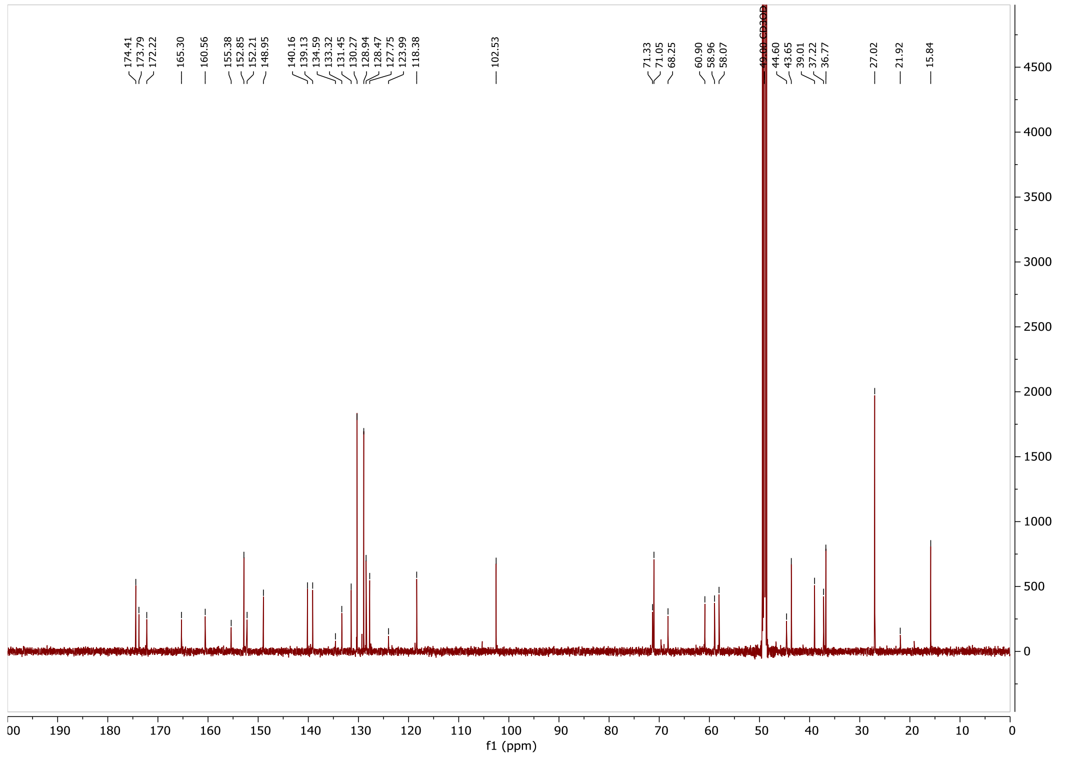

Compound **8**

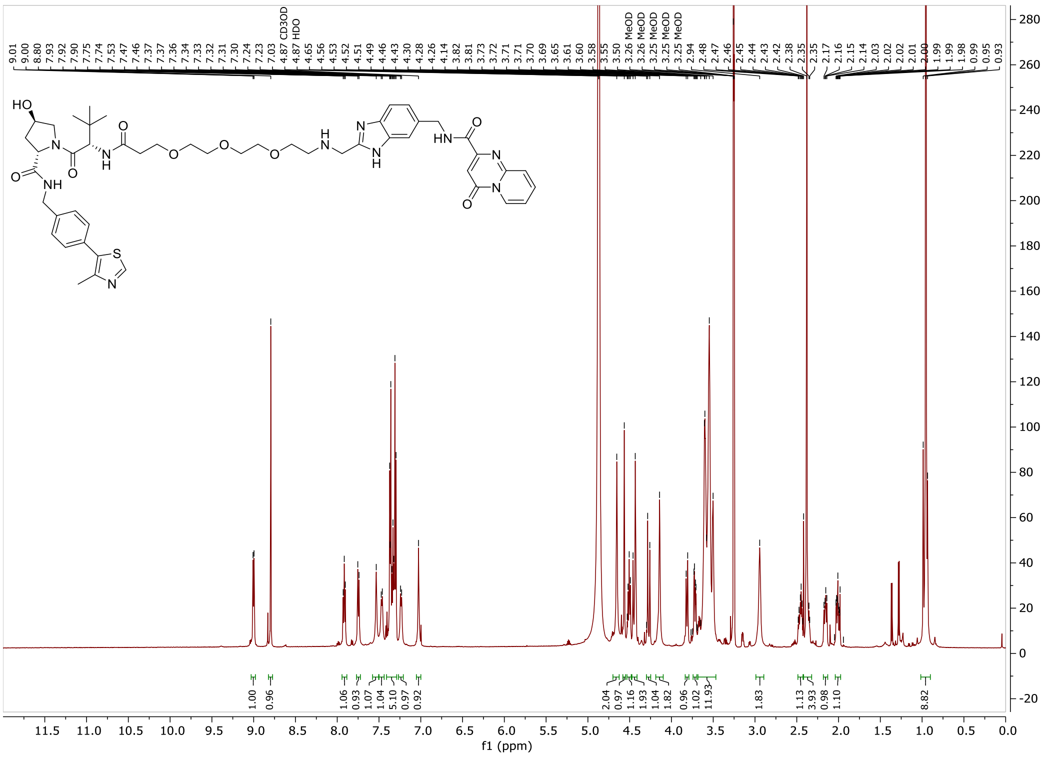
9.01 9.00 8.80 7.93 7.92 7.90 7.75 7.74 7.53 7.47 7.46 7.37 7.37 7.36 7.34 7.33 7.32 7.31 7.30 7.24 7.23 7.03 4.87 CD3OD 4.87 HDO 4.65 4.56 4.53 4.52 4.51 4.49 4.46 4.43 4.30 4.28 4.26 4.14 3.82 3.81 3.73 3.72 3.71 3.71 3.70 3.69 3.65 3.61 3.60 3.58 3.55 3.50 3.26 MeOD 3.26 MeOD 3.25 MeOD 3.25 MeOD 3.25 MeOD 2.94 2.48 2.47 2.46 2.45 2.44 2.43 2.42 2.38 2.35 2.35 2.17 2.16 2.15 2.14 2.03 2.02 2.02 2.01 2.00 1.99 1.99 1.98 0.99 0.95 0.93
1.00 0.96 1.06 0.93 1.07 1.04 5.10 0.97 0.92 2.04 0.97 1.16 1.93 1.04 1.82 0.96 1.02 11.93 1.83 1.13 3.93 0.98 1.10 8.82
f1 (ppm)

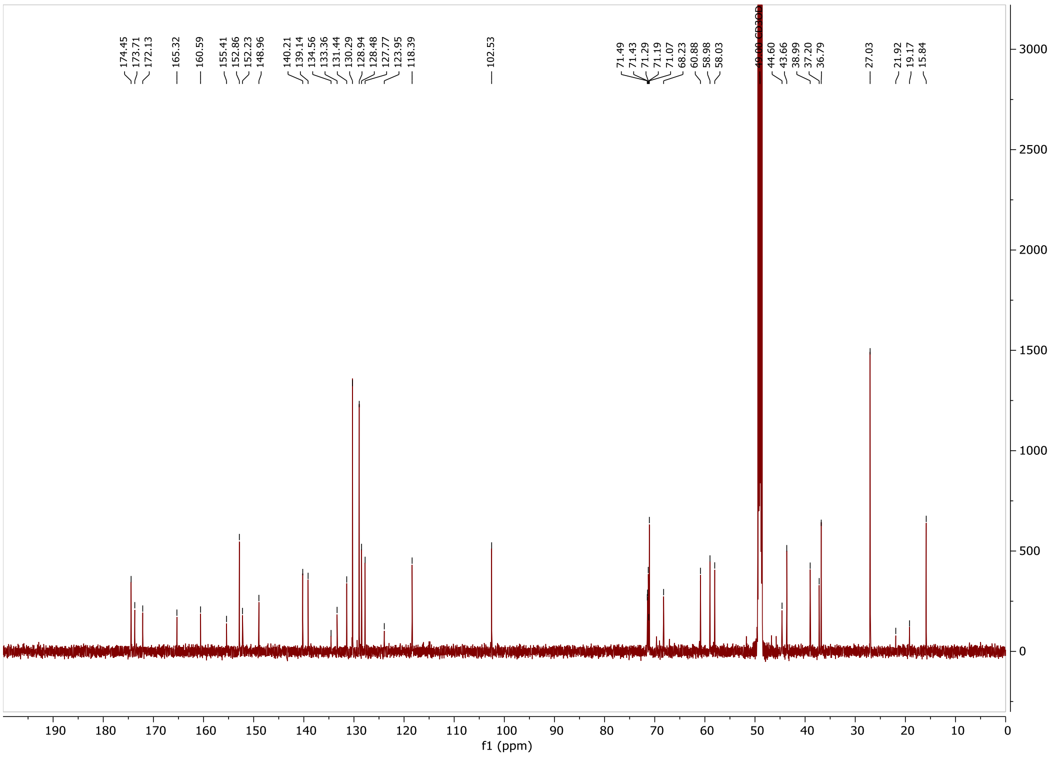
174.45 173.71 172.13 165.32 160.59 155.41 152.86 152.23 148.96 140.21 139.14 134.56 133.36 131.44 130.29 128.94 128.48 127.77 123.95 118.39 102.53 71.49 71.43 71.29 71.19 71.07 68.23 60.88 58.98 58.03 44.60 43.66 38.99 37.20 36.79 27.03 21.92 19.17 15.84
f1 (ppm)

Compound **9**

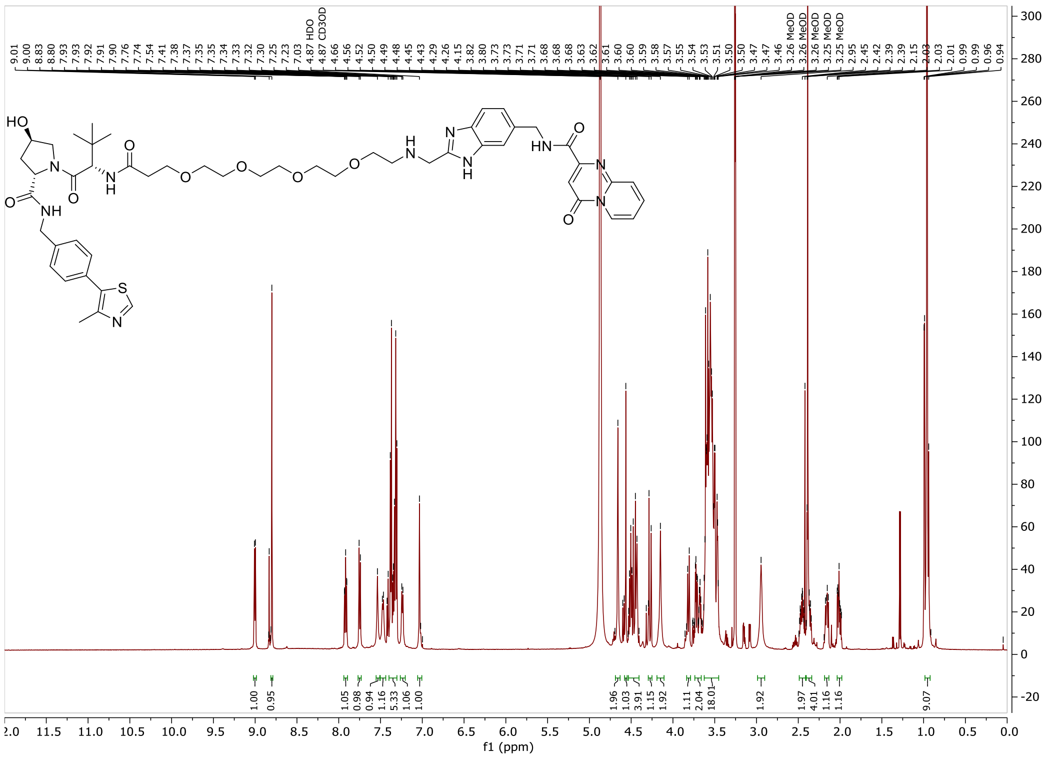
9.01 9.00 8.83 8.80 7.93 7.93 7.92 7.91 7.90 7.76 7.74 7.54 7.41 7.38 7.37 7.35 7.35 7.34 7.33 7.32 7.30 7.25 7.23 7.03 4.87 HDO 4.87 CD3OD 4.66 4.56 4.52 4.50 4.49 4.48 4.45 4.43 4.29 4.26 4.15 3.82 3.80 3.73 3.73 3.71 3.71 3.68 3.68 3.68 3.63 3.62 3.61 3.60 3.60 3.59 3.58 3.57 3.55 3.54 3.53 3.51 3.50 3.50 3.47 3.47 3.46 3.26 MeOD 3.26 MeOD 3.26 MeOD 3.25 MeOD 3.25 MeOD 2.95 2.45 2.42 2.39 2.39 2.15 2.03 2.03 2.01 0.99 0.99 0.96 0.94
1.00 0.95 1.05 0.98 0.94 1.16 5.33 1.06 1.00 1.96 1.03 3.91 1.15 1.92 1.11 2.04 18.01 1.92 1.97 4.01 1.16 1.16 9.07
f1 (ppm)

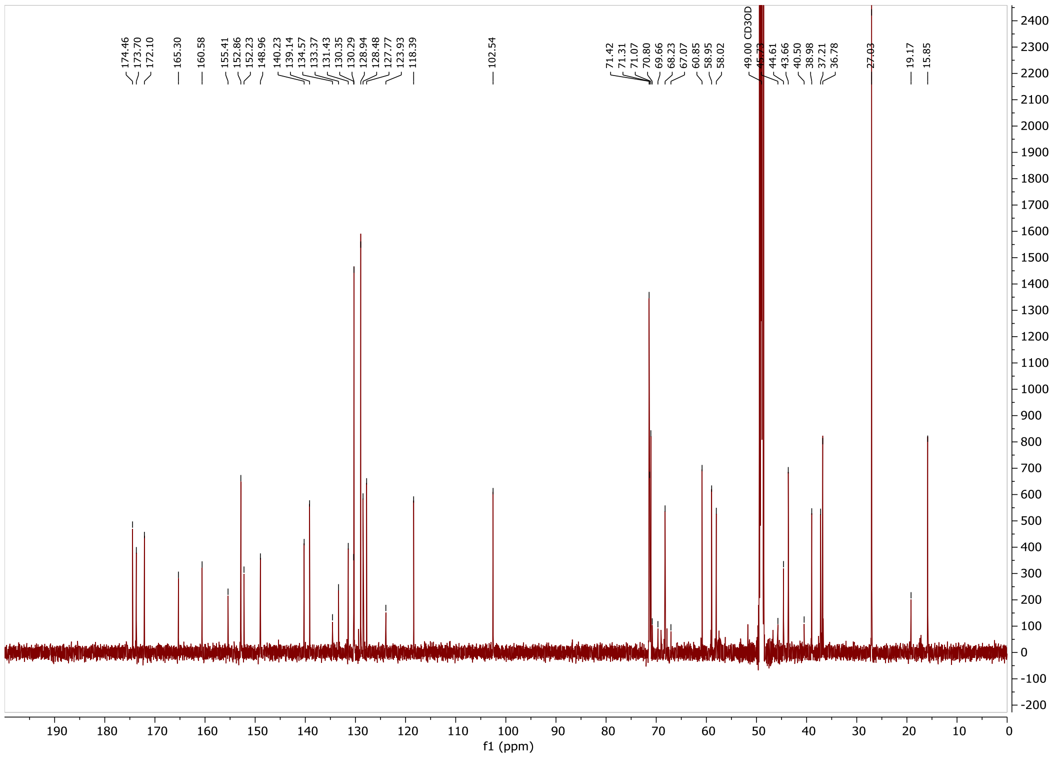
174.46 173.70 172.10 165.30 160.58 155.41 152.86 152.23 148.96 140.23 139.14 134.57 133.37 131.43 130.35 130.29 128.94 128.48 127.77 123.93 118.39 102.54 71.42 71.31 71.07 70.80 69.66 68.23 67.07 60.85 58.95 58.02 49.00 CD3OD 44.61 43.66 40.50 38.98 37.21 36.78 27.03 19.17 15.85
f1 (ppm)

Compound **10**

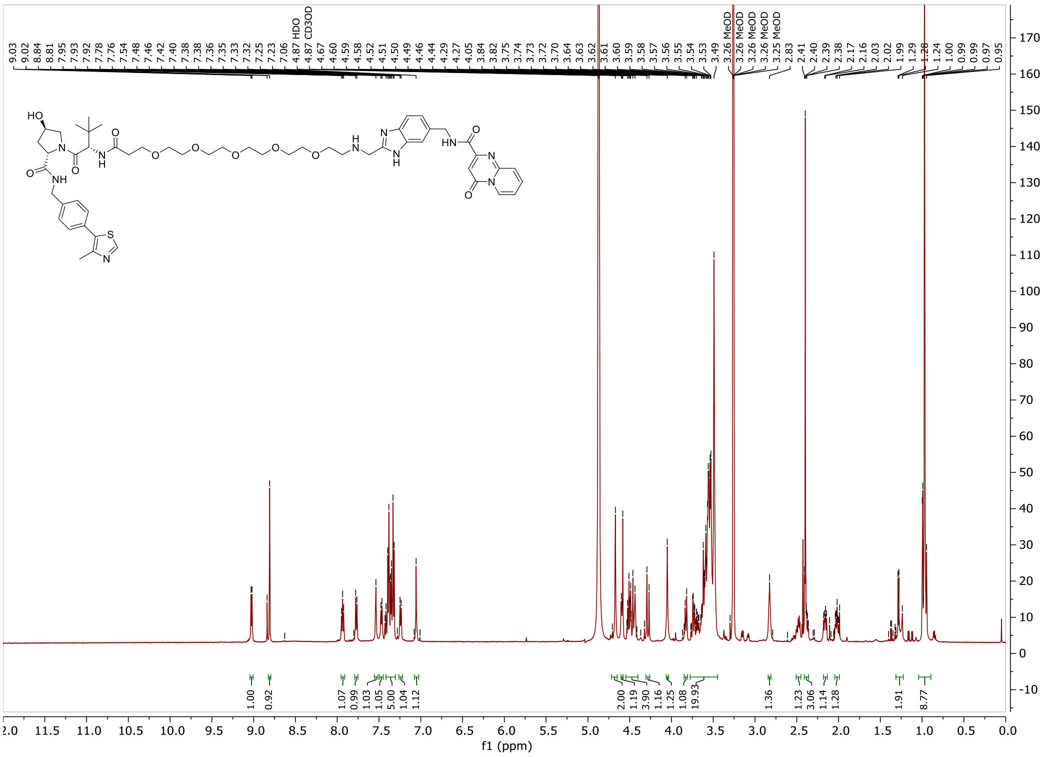
9.03 9.02 8.84 8.81 7.95 7.93 7.92 7.78 7.76 7.54 7.48 7.46 7.42 7.40 7.38 7.38 7.36 7.35 7.33 7.32 7.25 7.23 7.06 4.87 HDO 4.87 CD3OD 4.67 4.60 4.59 4.58 4.52 4.51 4.50 4.49 4.46 4.44 4.29 4.27 4.05 3.84 3.82 3.75 3.74 3.73 3.72 3.70 3.64 3.63 3.62 3.61 3.60 3.59 3.58 3.57 3.56 3.55 3.54 3.53 3.49 3.26 MeOD 3.26 MeOD 3.26 MeOD 3.26 MeOD 3.25 MeOD 2.83 2.41 2.40 2.39 2.38 2.17 2.16 2.03 2.02 1.99 1.29 1.28 1.24 1.00 0.99 0.99 0.97 0.95
f1 (ppm)

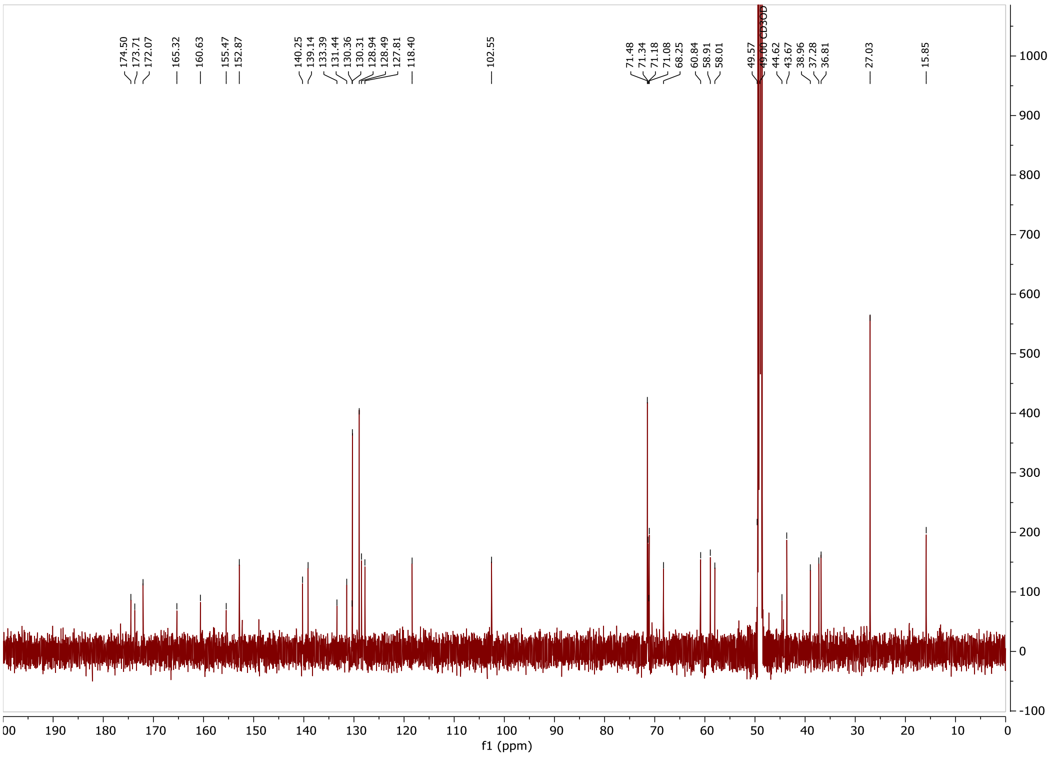
174.50 173.71 172.07 165.32 160.63 155.47 152.87 140.25 139.14 133.39 131.44 130.36 130.31 128.94 128.49 127.81 118.40 102.55 71.48 71.34 71.18 71.08 68.25 60.84 58.91 58.01 49.57 49.00 CD3OD 44.62 43.67 38.96 37.28 36.81 27.03 15.85
f1 (ppm)

Compound **39**

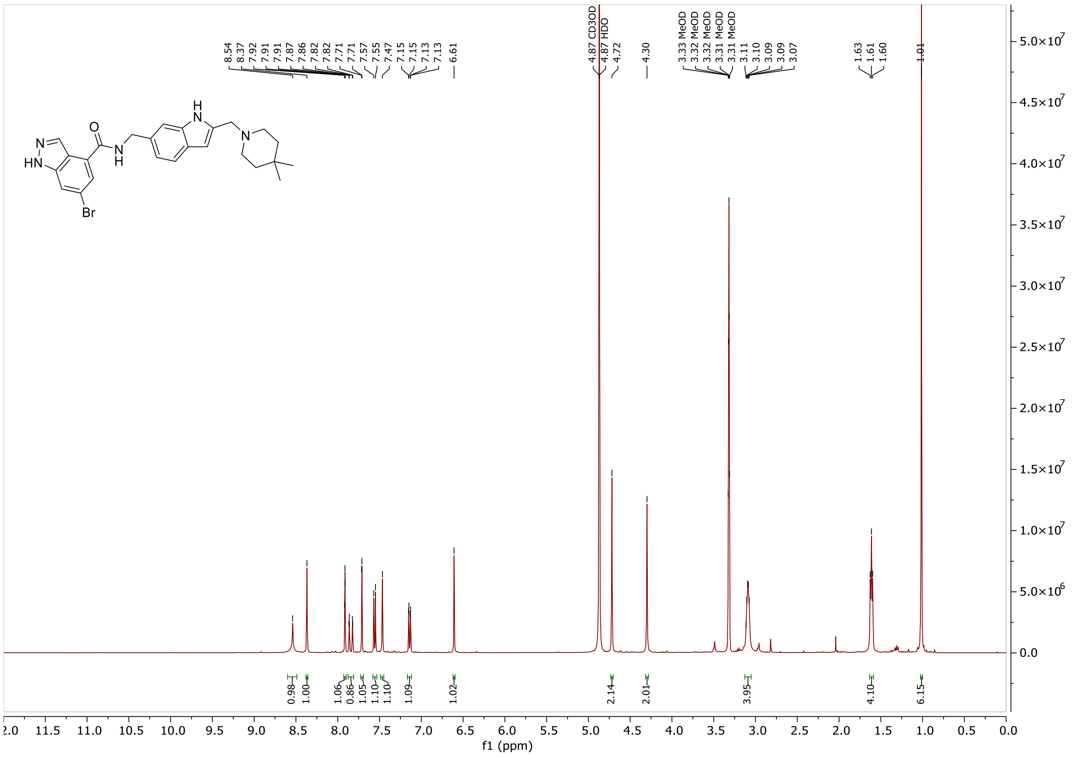
8.54
8.37
7.92
7.91
7.91
7.87
7.86
7.82
7.82
7.71
7.71
7.57
7.55
7.47
7.15
7.15
7.13
7.13
6.61
4.87 CD3OD
4.87 HDO
4.72
4.30
3.33 MeOD
3.32 MeOD
3.32 MeOD
3.31 MeOD
3.31 MeOD
3.11
3.10
3.09
3.09
3.07
1.63
1.61
1.60
1.01
0.98
1.00
1.06
0.86
1.05
1.10
1.10
1.09
1.02
2.14
2.01
3.95
4.10
6.15
N
HN
O
N
H
H
N
N
Br
f1 (ppm)

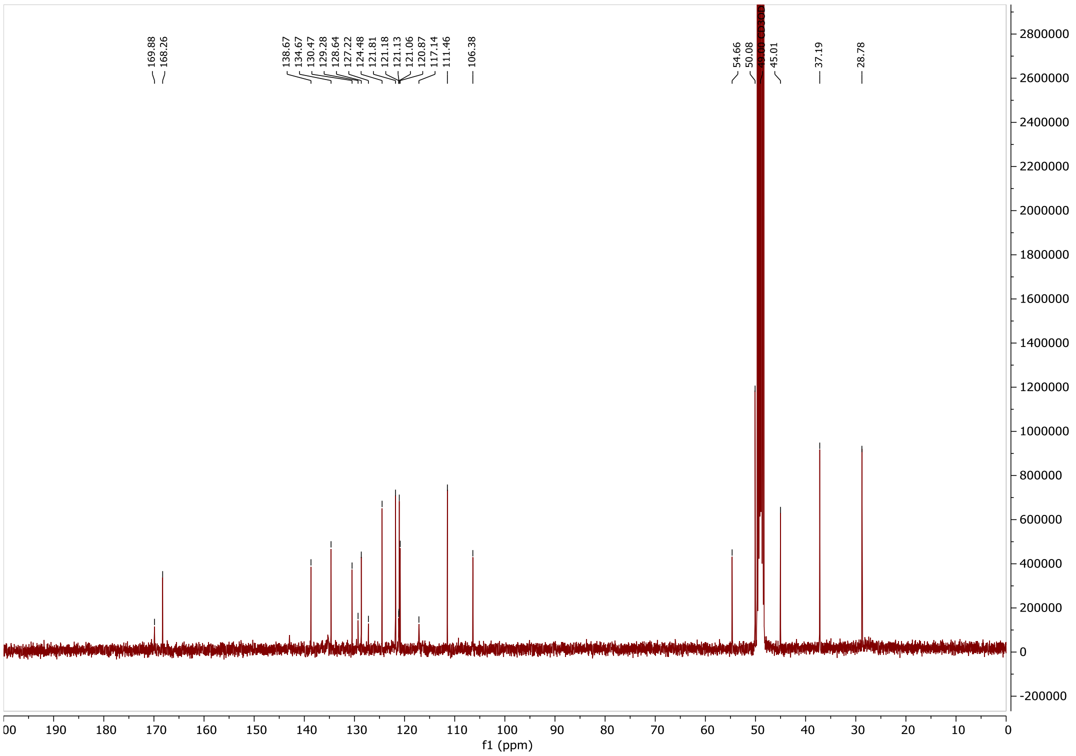
169.88
168.26
138.67
134.67
130.47
129.28
128.64
127.22
124.48
121.81
121.18
121.13
121.06
120.87
117.14
111.46
106.38
54.66
50.08
45.01
37.19
28.78
f1 (ppm)
